# Supplementary material for: Highly scalable photoinduced synthesis of silanols via untraversed pathway for chlorine radical (Cl•) generation
Source: Nat Commun. 2023 Dec 9;14:8173. doi: 10.1038/s41467-023-43286-z (PMC10710510; doi:10.1038/s41467-023-43286-z)
Supplement: Supplementary file 1 — Supplementary information [file 41467_2023_43286_MOESM1_ESM.pdf]

Supplementary Information

Highly Scalable Photoinduced Synthesis of Silanols via Untraversed Pathway for Chlorine Radical ($Cl^{\bullet}$) Generation

Argha Saha,[†1] Wajid Ali,[†1] Daniel B. Werz[2]* & Debabrata Maiti[1]*

[1]Department of Chemistry, Indian Institute of Technology Bombay, Powai, Mumbai 400076, India

*Email: dmaiti@iitb.ac.in

[2]Albert-Ludwigs-Universitat Freiburg, Institute of Organic Chemistry, Albertstr. 21, 79104 Freiburg, Germany.

[†]These authors contributed equally.

Table of Contents

Section		Title	Page No
**1**		General consideration	**S3 – S5**
**2**		Experimental section	
	**2.1**	Preparation of starting materials	**S6 – S7**
	**2.2**	Optimization details for photoinduced silanol synthesis	**S8 – S10**
	**2.3**	General procedure for photoinduced silanol synthesis	**S10 – S11**
	**2.4**	General procedures for applicative protocols	**S11 – S13**
**3**		Mechanistic Studies	
	**3.1**	Control experiments	**S13**
	**3.2**	Evidence of superoxide formation	**S14**
	**3.3**	Evidence of $^1O_2$ by trapping with anthracene	**S14**
	**3.4**	Effect on radical scavenger	**S15**
	**3.5**	Quantum Yield Measurement	**S15 - S17**
	**3.6**	Evidence of Cl anion formation	**S18**
	**3.7**	Powder X-Ray diffraction graph	**S19**
	**3.8**	Source of hydroxyl group in the reaction	**S19 – S20**
**4**		On/Off experiment	**S20-S21**
**5**	**5.1**	Hammet plot	**S21 – S22**
	**5.2**	Kinetic isotope effect experiment	**S22**
	**5.3**	Competition experiment	**S22 - S23**
**6**		Characterization data of silanol products	**S23 – S52**
**7**		NMR Spectra's	**S53 - S117**
**8**		References	**S118**

1. General Consideration:

1.1. Reagent Information. All the reactions were carried out in screw cap reaction tubes under aerobic conditions unless otherwise stated. All the chemicals were purchased from Sigma Aldrich, Alfa Aesar, and TCI-India. Solvents were bought from commercial sources and were used without further purification. Silica gel (100–200 mesh) was used for column chromatography purchased from Merck. Petroleum ether and ethyl acetate mixture were used as a gradient elution for column chromatography. A gradient elution using petroleum ether and ethyl acetate was performed, based on Merck aluminum TLC sheets (silica gel 60F254).

1.2. Analytical Information. All isolated compounds were characterized by ^{1}H NMR, ^{13}C NMR spectroscopy, and HRMS. Unless otherwise stated, all Nuclear Magnetic Resonance spectra were recorded on Bruker 400 MHz and 500 MHz instruments. NMR spectra are reported in parts per million (ppm) and were measured relative to the signals for residual solvent in the deuterated solvent unless otherwise stated. All optimization analysis was performed by ^{1}H NMR analysis with TMB as the internal standard. The description of the light set-up used for this transformation is 440 nm, 34 W Kessil Lamp. All ^{13}C NMR spectra were obtained with ^{1}H decoupling. High-resolution mass spectra (HRMS) were recorded on a micro-mass ESI TOF (time of flight) mass spectrometer.

1.3. Description of Reaction Tube and Photo Setup:

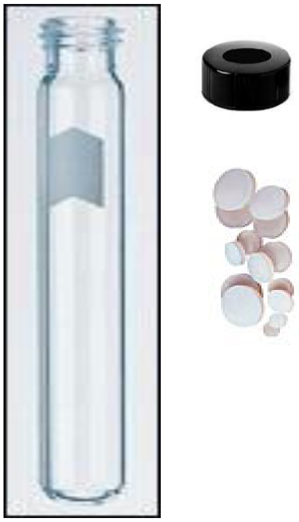

Figure 1. A pictorial description of reaction tube for photoinduced silanol synthesis: Fisher brand Disposable Borosilicate Glass Tubes (16*125mm) with Threaded End (Fisher Scientific, Order No. 1495935A) [left]; Kimble Black Phenolic Screw Thread Closures with Open Tops (Fisher Scientific Order No. 033407E) [left]; Thermo Scientific National PTFE/Silicone Septa for Sample Screw Thread Caps (Fisher Scientific Order No. 03394A) [left].

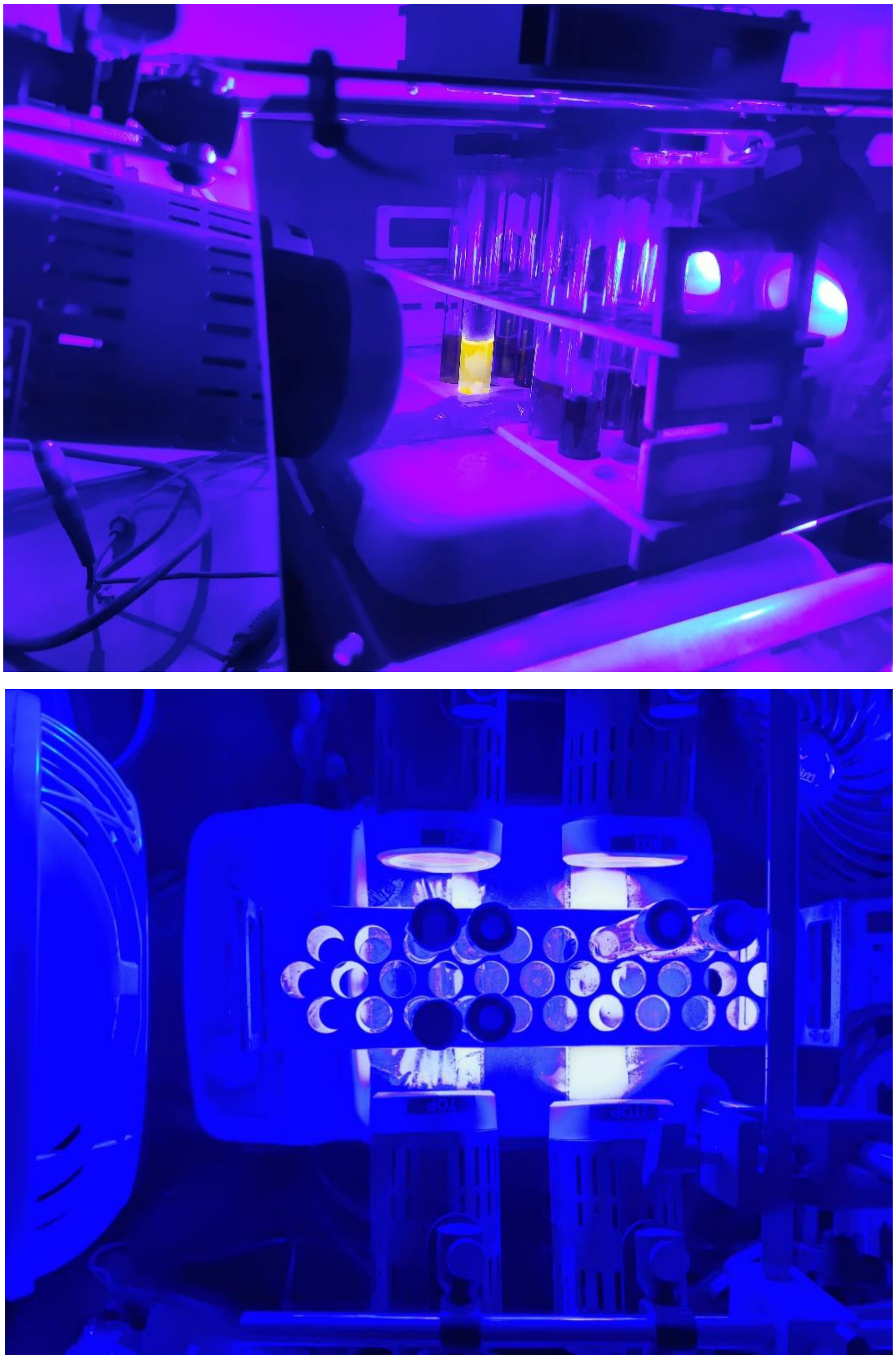

Supplementary Fig 1: Photograph of the light set used for irradiation of the reactions.

Supplementary Fig 2: Photograph of the light set used for flow synthesis.

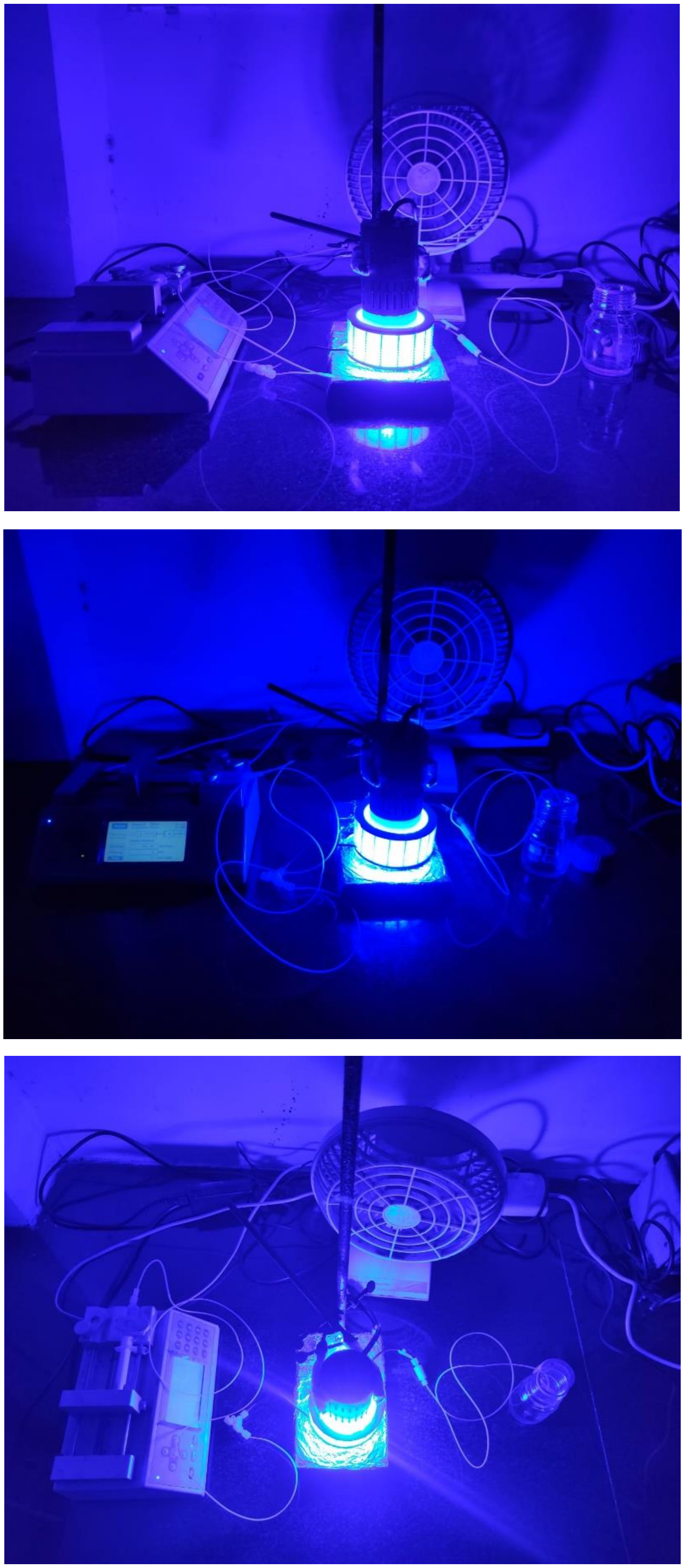

Supplementary Fig 2: Photograph of the light set used for flow synthesis.

2. Experimental Section

2.1.A. Preparation of starting materials:

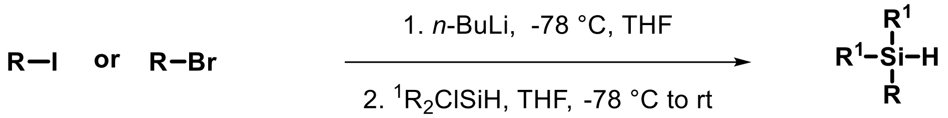

In a clean oven-dried 100 mL round-bottom flask, aryl halide (5.0 mmol, 1.0 *eq*.) in THF (10 mL) was cooled to -78 °C. After that *n*-BuLi (3.2 mL,1.6 M in THF, 6.0 mmol, 1.2 *eq*.) was added dropwise to the reaction mixture over 30 min. The resulting mixture was stirred at -78 °C for 2 h and followed by the dropwise addition of chlorodimethylsilane (6.0 mmol, 1.2 *eq*.) or chlorodi-isopropylsilane (6.0 mmol, 1.2 *eq*.). The reaction was allowed to warm to room temperature and stirred for another 8 h. After completion of the reaction, it was quenched with saturated aqueous NH_4Cl solution and was extracted with ethyl acetate. The combined organic layer was washed with water, and brine, dried over anhydrous Na_2SO_4, and then concentrated under reduced pressure. The residue was purified by column chromatography using petroleum ether/ethyl acetate as the eluent to afford the hydrosilane.

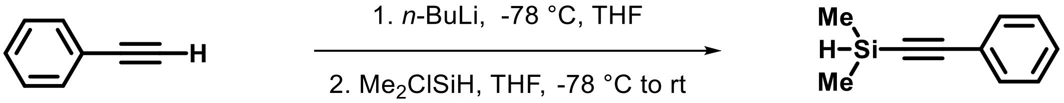

In a clean oven-dried 100 mL round-bottom flask, phenylacetylene (5.0 mmol, 1.0 *eq*.) in THF (10 mL) was cooled to -78 °C. After that *n*-BuLi (3.2 mL,1.6 M in THF, 6.0 mmol, 1.2 *eq*.) was added dropwise slowly to the reaction mixture over 30 min. The resulting mixture was stirred at -78 °C for 2 h and followed by the dropwise addition of chlorodimethylsilane (6.0 mmol, 1.2 *eq*.). The reaction was allowed to warm to room temperature and stirred for 4 h. After completion of the reaction, it was quenched with saturated aqueous NH_4Cl solution and the mixture was extracted with ethyl acetate. The combined organic layer was washed with water, and brine, dried over anhydrous Na_2SO_4, and then concentrated under reduced pressure. The residue was purified by column chromatography using petroleum ether/ethyl acetate as the eluent to afford the hydrosilane.

2.1.B. General route for the synthesis of benzyl silanes:

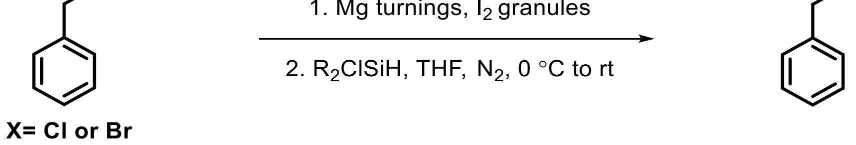

In a clean oven-dried 100 mL round-bottom flask, charged with a magnetic stir–bar, activated magnesium turnings (15.0 mmol, 3.0 *eq.*) and I_2 (one bead) were taken. The reaction vessel was evacuated and backfilled with nitrogen three times. Dry THF (15 mL) was added to it followed by chlorodimethylsilane (6.0 mmol, 1.2 *eq.*) or chlorodi-isopropylsilane (6.0 mmol, 1.2 *eq.*) in dropwise fashion and stirred at room temperature for 15 min. A solution of benzyl chloride/bromide (5.0 mmol) in dry THF (10 mL) was added to the solution dropwise over a period of 15 min. under ice-cold conditions. The mixture was vigorously stirred for 3 hours. Upon completion, the reaction mixture was quenched and washed with brine solution. The aqueous part was extracted three times with ethyl acetate. The combined organic layer was then dried over anhydrous Na_2SO_4. The corresponding silane was purified by column chromatography using petroleum ether/ethyl acetate as the eluent.

2.1.C. General synthesis of diphenyl alkoxy silane:

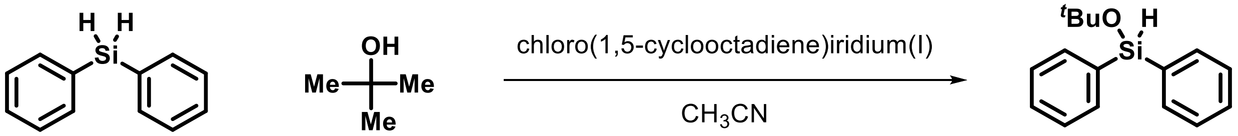

In a clean oven-dried reaction tube, diphenyl silane (3.0 mmol, 1.0 *eq.*) and ^{t}BuOH (4 mmol, 1.33 *eq.*) in acetonitrile (3 mL) were taken. Followed by the addition of catalyst chloro(1,5-cyclooctadiene)iridium(I) dimer (20 mg, 1 mol %) to the resulting reaction mixture. Then the reaction mixture was stirred at rt for 24 h under an air atmosphere. After completion of the reaction the solvent was removed under reduced pressure and the crude product was purified by column chromatography using petroleum ether/ethyl acetate as the eluent to afford the product as a colorless oil. All the synthesized hydrosilanes were characterized by ^{1}H and ^{13}C NMR spectroscopy and characterization data were matched with previous reports.[1-4]

2.2.A. Optimization details for the photoinduced silanol synthesis:

Yield was determined by ^{1}H NMR analysis of the crude product using TMB as the internal standard.

Initial reaction condition:

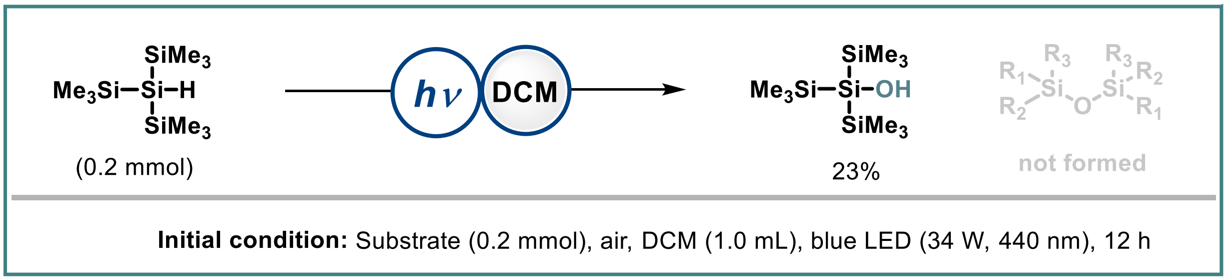

Supplementary Table 1: Reaction under oxygen atmosphere:

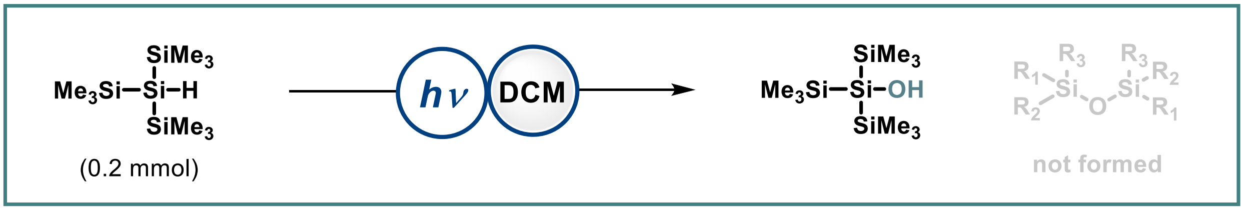

Entry	Condition- DCM (1.0 ml)	NMR Yield (%)
**1**	**12 h, (O_2 purged)**	**52**

Supplementary Table 2: Optimization by varying amounts of water:

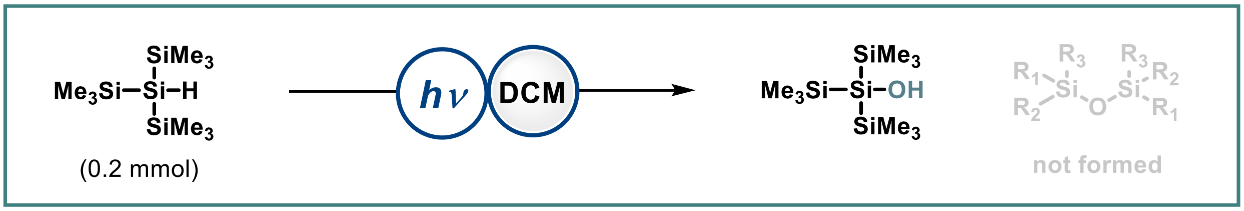

Entry	Condition- (O_2 purged), DCM (1.0 ml), 12 h	NMR Yield (%)
1	H_2O (1 *eq.*)	57
2	H_2O (2 *eq.*)	61
3	H_2O (3 *eq.*)	64
4	H_2O (4 *eq.*)	68
**5**	**H_2O (5 *eq.*)**	**73**
6	H_2O (6 *eq.*)	73

Supplementary Table 3: Time optimization:

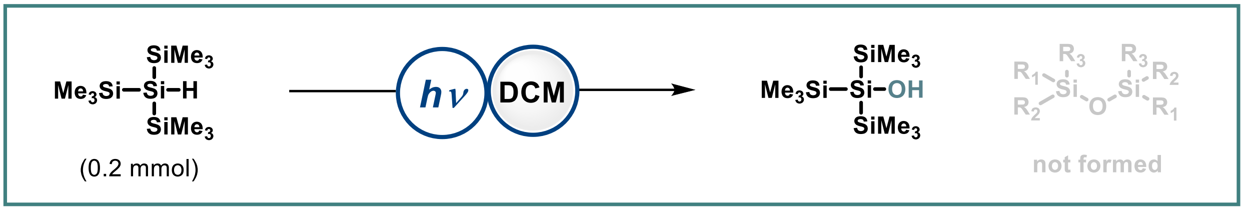

Entry	Condition- (O_2 purged), DCM (1.0 ml), H_2O (5 *eq.*)	NMR Yield (%)
1	16 h	79
2	20 h	86
3	24 h	92
**4**	**28 h**	**95**
5	30 h	94

Supplementary Table 4: Solvent optimization:

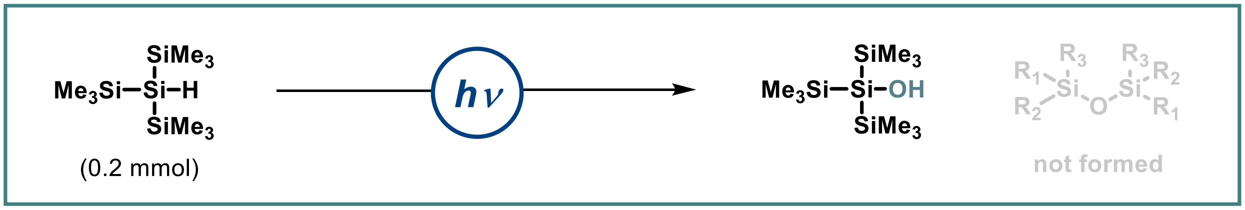

Entry	Condition- (O_2 purged), H_2O (5 *eq.*), 28 h	NMR Yield (%)
1	DCE	85
2	$CHCl_3$	88
3	CCl_4	89
4	Chlorobenzene	26
5	Bromobenzene	5
6	Iodobenzene	trace
7	Fluorobenzene	7
8	CH_3CN	11
9	THF	8

2.2.B. Optimization details for photoinduced silanol synthesis for continuous-flow reactions:

Yield was determined by ^{1}H NMR analysis of the crude product using TMB as the internal standard.

Supplementary Table 5: Flow set-up optimization:

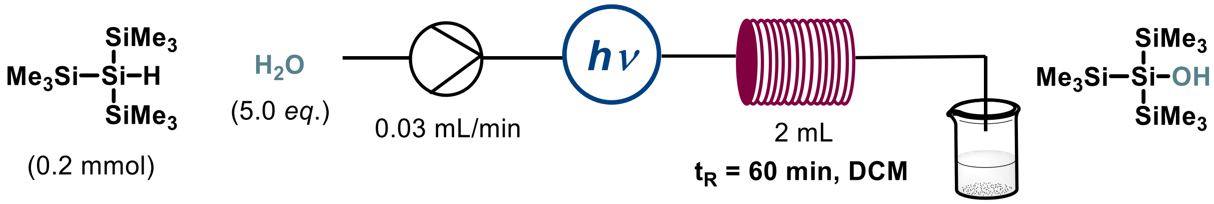

Entry	Time optimization	NMR Yield (%)
1	5 min	33
2	10 min	44
3	20 min	52
4	30 min	60
5	40 min	69
6	50 min	72
**7**	**60 min**	**86**
8	65 min	85

2.3.A. General procedure for photoinduced silanol synthesis:

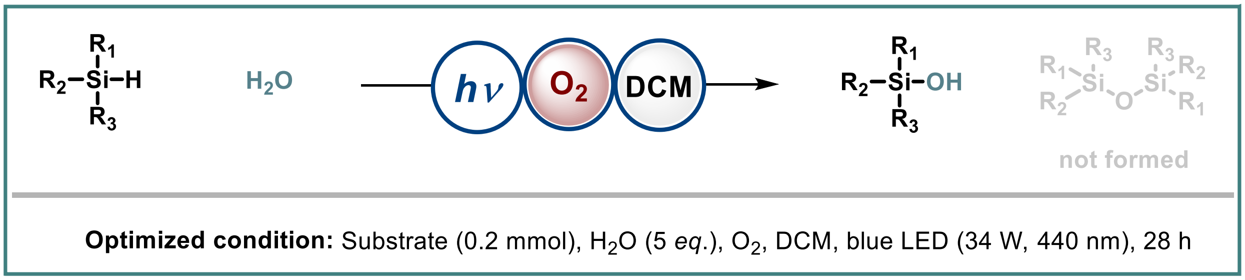

An oven-dried screw capped reaction tube equipped with magnetic stir-bar was charged with corresponding solid silanes (0.2 mmol, 1 *eq*.). Then, the tube was capped with a screw cap with rubber septum and purged with oxygen for three times. Then, the cap was wrapped with a teflon. Subsequently, water (1.0 mmol, 5 *eq*.) and 1 mL of dichloromethane was added to the reaction tube. Then the reaction tube was placed 3 cm away from 34 W Kessil lamp with stirring (1000 rpm) for 28 h. For liquid silanes, the addition of silanes was done after the addition of the reaction

solvent. The temperature was maintained by cooling with two fans. Upon completion of the reaction, the solvent was removed under reduced pressure, and the crude mixture was purified by column chromatography using silica (100-200 mesh size) and petroleum ether/ethyl acetate as the eluent.

Under this reaction condition, $(TMS)_3SiOH$ **35** is produced at 44 g/h/L and up to 1.05 Kg/day/L reactor volume. The product **52**, **53** and **56** were scaled up to 32.4 g/h/L, 37.4 g/h/L and 41.4 g/h/L respectively.

2.3.B. General procedure for photoinduced silanol synthesis from continuous-flow reactions: Following the above-mentioned general procedure, a homogenous solution of silanes in DCM was prepared and filled in a 10 mL syringe. The syringe was then loaded on the syringe pump and the solution was fed into a microreactor (2 mL) which was irradiated with 34 W Kessil lamp at a speed of 0.03 mL/min which is equivalent to 60 min of residence time. When the complete solution was injected into the microreactor, another syringe was refilled with DCM solvent and reloaded on the syringe pump to flush out the product. In order to collect the product, an outlet was connected to a receiver flask, and the product was collected over a period of time. The temperature was maintained by cooling with a fan. Upon completion, the solvent was evaporated under reduced pressure and the crude mixture was purified by column chromatography using silica (100-200 mesh size) and petroleum ether/ethyl acetate as the eluent.

2.4. General procedure for applicative protocols:

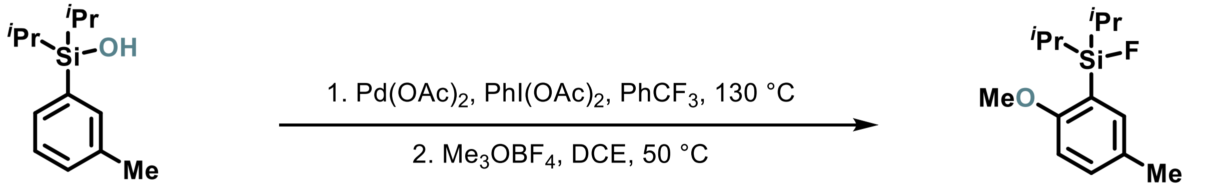

3

60, 42%, over 2 steps

General procedure:[5] An oven-dried reaction tube containing a stirring bar was charged with product **3** (1.0 *eq.*), $Pd(OAc)_2$ (5 mol %), and $PhI(OAc)_2$ (1.5 *eq.*) under N_2 atmosphere. After that dry α,α,α-trifluorotoluene (1 mL) was added *via* syringes, and the reaction tube was capped with a screw cap. The reaction mixture was heated at 100 °C for 7 h. The reaction mixture was then cooled to room temperature and the mixture was concentrated under a reduced pressure. the crude product was purified by column chromatography using silica (100-200 mesh size) and petroleum ether/ethyl acetate as the eluent. Then, $Me_3O^+BF_4^-$ (2.0 *eq.*) and Dry DCE (0.5 mL) were added to

the obtained product and heated at 50 °C for 48 h. After completion of the reaction, the mixture was cooled to room temperature, diluted with DCM, and treated with saturated aqueous solution of sodium bicarbonate. The reaction mixture was stirred for an additional 30 min at RT. The aqueous layer was extracted with DCM and the organic phase was washed with water. The combined organic extracts dried over Na_2SO_4 and evaporated under reduced pressure. The crude mixture was purified by column chromatography using silica (100-200 mesh size) and petroleum ether/ethyl acetate as the eluent.

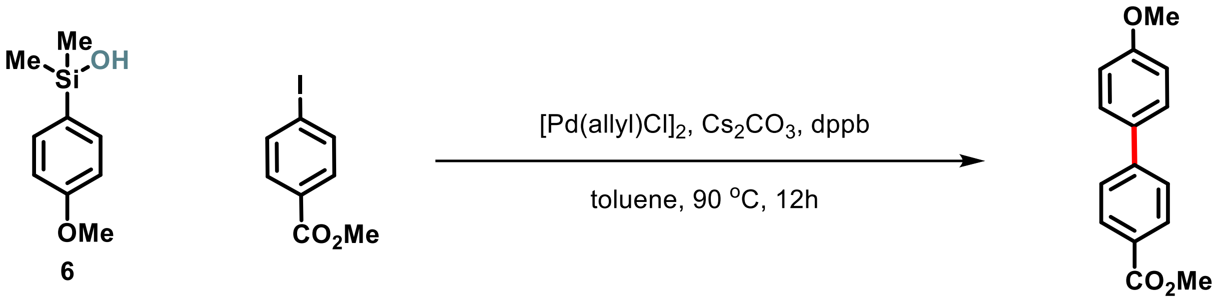

General procedure:[6] An oven dried reaction tube containing a stirring bar, was charged with anhydrous cesium carbonate (2.0 *eq*.) and suspended in dry toluene (1.0 mL) at room temperature. To this solution H_2O (6.0 *eq*.) was added dropwise and was allowed to stir for 10 min. Methyl 4-iodobenzoate (1.0 mmol), and product **6** (1.2 *eq*.) were then added followed by [allylPdCl]$_2$ (5 mol%) and triphenylarsine (30.6 mg, 0.1 mmol, 0.1 *eq*.). Finally, the reaction tube was purged with argon and placed in a preheated 90 °C bath. Upon completion, the reaction was cooled to rt, and extracted with ethyl acetate and washed with water. The combined organic extracts dried over Na_2SO_4 and evaporated under reduced pressure. The crude mixture was purified by column chromatography using silica (100-200 mesh size) and petroleum ether/ethyl acetate as the eluent.

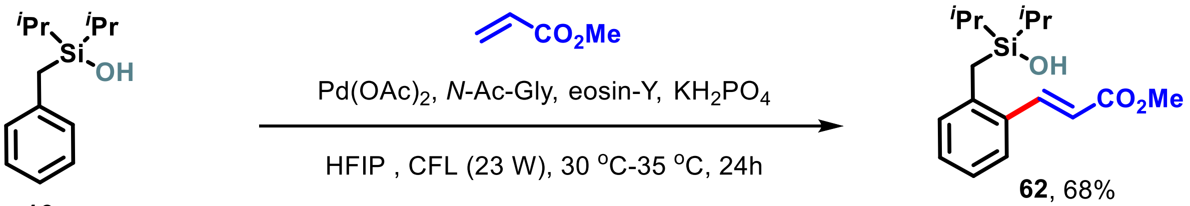

General procedure:[7] In an oven-dried screw capped reaction tube was charged with magnetic stir-bar, product **16** (1.0 mmol), $Pd(OAc)_2$ (10 mol%), *N*-Ac-Gly (20 mol%), KH_2PO_4 (2.0 *eq*.), eosin Y (3 mol%.) and olefin (2.0 *eq*.) in 1 mL of 1,1,1,3,3,3-hexafluoro-2-propanol (HFIP) were added. The reaction tube was capped and placed 3 cm away from four 23 W house hold CFL bulbs

with stirring (1500 rpm) at room temperature for 24 h. The temperature was maintained at approximately (30-35 °C) through cooling with a fan. Upon completion of the reaction, it was diluted with ethyl acetate and filtered through a celite pad. The filtrate was evaporation under reduced pressure and the crude mixture was purified by column chromatography using silica (100-200 mesh size) and petroleum ether/ethyl acetate as the eluent.

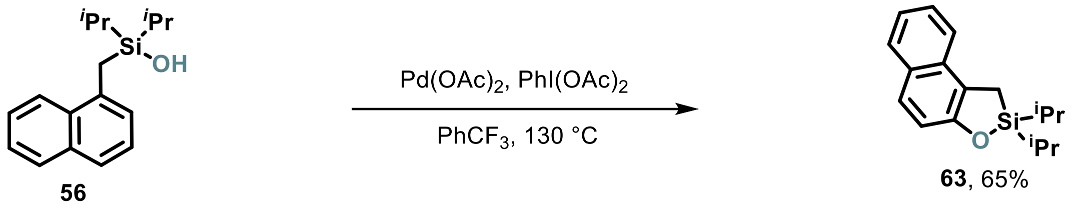

General procedure:[5] An oven dried reaction tube containing a stirring bar, was charged with product **56** (0.1 mmol), $Pd(OAc)_2$ (5 mol%), and $PhI(OAc)_2$ (0.15 mmol) under N_2 atmosphere. After that dry α,α,α-trifluorotoluene (1 mL) was added *via* syringes and the reaction tube was capped with screw cap. The reaction mixture was heated at 100 °C for 7 h. The resulting mixture was cooled to room temperature and the mixture was concentrated under a reduced pressure. The crude mixture was purified by column chromatography using silica (100-200 mesh size) and petroleum ether/ethyl acetate as the eluent.

3. Mechanistic Studies:

3.1. Control experiments:

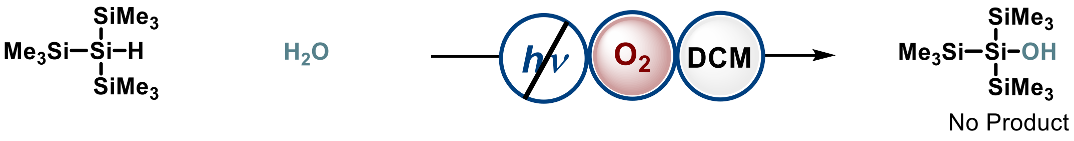

3.1.A. Following the general procedure for silanol synthesis, the above stated control experiment was performed in absence of 34 W Kessil lamp (dark condition) for 28 h.

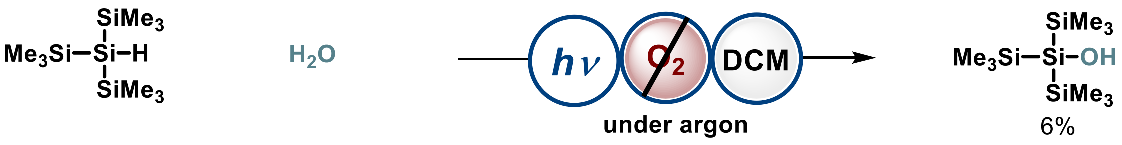

3.1.B. Following the general procedure for silanol synthesis, the above stated control experiment was performed in presence of 34 W Kessil lamp for 28 h under argon atmosphere. The experiment clearly shows the significant decrease in yield, hence oxygen purged condition is the integral part of the reaction condition.

3.2. Evidence of superoxide formation:

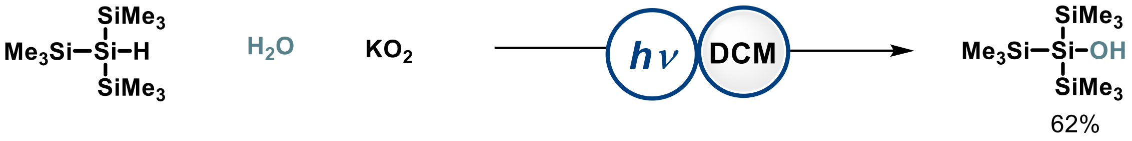

Following the general procedure for silanol synthesis, the control experiment was performed with potassium superoxide KO_2 (1.2 *eq*.) in absence of oxygen with 34 W Kessil lamp for 28 h. It is observed that the product was obtained in 62% yield. So, it can be concluded that the superoxide formation is taking place in the reaction medium.

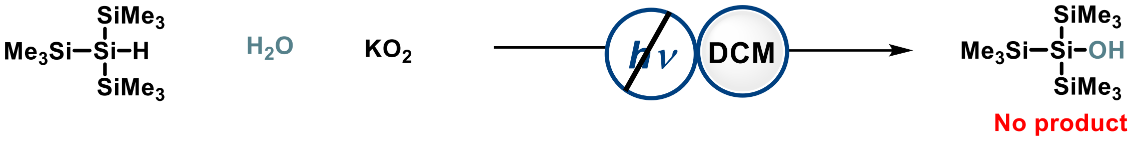

Following the general procedure for silanol synthesis, the control experiment was performed with potassium superoxide KO_2 (1.2 *eq*.) under argon and blue LEDs for 28 h. It is observed that the product was not formed. So, it can be concluded that the light is necessary for the superoxide formation.

3.3. Evidence of $^1O_2$ by trapping with anthracene:

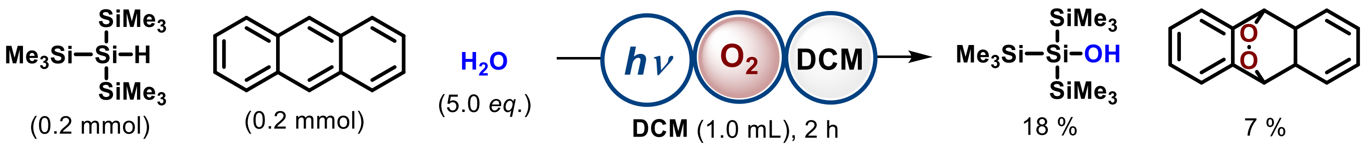

Following the general procedure, the control experiment was performed in presence of silane (0.2 mmol), anthracene (0.2 mmol), H_2O (5 *equiv*.) and dichloromethane as solvent in presence of 34 W Kessil lamp for 2 h. After completion of the reaction, it was analyzed by GC-MS to give the silanol and endoperoxide product in 18 % and 7 % yield respectively. However, the result of the parallel experiment, which was performed under the same reaction condition except avoiding light irradiation showed no endoperoxide product. It confirms the presence of formation of $^1O_2$ in the reaction medium.

3.4. Effect on radical scavenger:

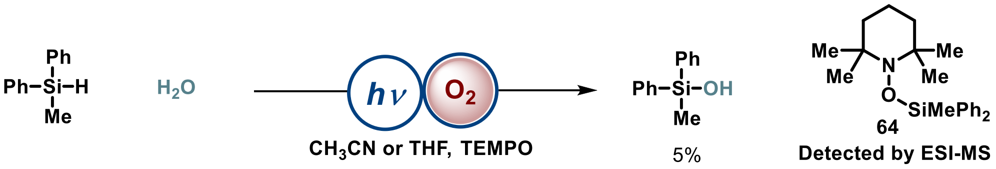

Following the general procedure for silanol synthesis, the control experiment was performed with radical scavenger TEMPO (3.0 *eq*.) with CH_3CN and THF as solvent in presence of 34 W Kessil lamp for 28 h. It is observed that there is significant decrease in product yield also formation of TEMPO-adduct (Fig S3) was observed. This observation further concluded that the reaction is proceeds with the generation of a radical species in the reaction.

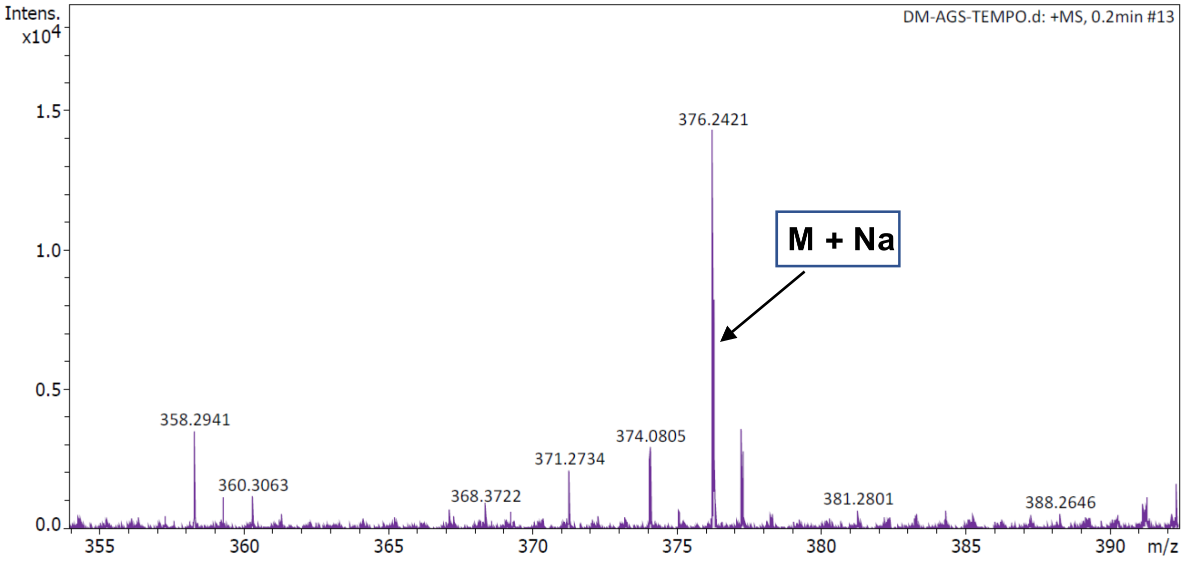

Supplementary Fig 3: TEMPO adduct was observed by ESI-MS mass analysis

3.5. Quantum Yield Measurement:

Determination of the light intensity at 440 nm: Each sample preparation and measurements were repeated two more times.

According to the literature reported proecdure,[8] the photon flux of the LED ($\lambda = 440$ nm) was first determined by standard ferrioxalate actinometry.[9] Initially, a 10 mL 0.15 M solution of ferrioxalate was prepared by dissolving potassium ferrioxalate hydrate (0.737 g) in H_2SO_4 (10 mL of a 0.05 M solution). Then 20 mL buffered solution of 1,10-phenanthroline was prepared by dissolving 1,10-

phenanthroline (20 mg) and sodium acetate (4.5 g) in H_2SO_4 (20 mL of a 0.5 M solution). Both solutions were stored in the dark to avoid decomposition.

In order to determine the photon flux of the blue LED, the ferrioxalate solution (1.0 mL) was placed in a cuvette and irradiated for 90 secs at λ_{max}= 440 nm. After irradiation, the phenanthroline solution (0.2 mL) was added to the cuvette and the mixture was stirred in the dark for 1.0 h. The absorption of the solution was measured at 510 nm. A non-irradiated sample was also prepared identically and the absorption at 510 nm was also measured. The average of the absorption of the irradiated and non-irradiated samples were determined and used for the calculation of photon flux.

$$\text{mol } Fe^{2+} = V \times \Delta A / l \times \varepsilon \qquad \text{..........(eq-1)}$$

where V is the total volume (0.00224 L) of the solution after addition of phenanthroline, ΔA is the difference in absorption at 510 nm between the irradiated and non-irradiated solutions (0.2872452, *l* is the path length (1.00 cm), and ε is the molar absorptivity at 510 nm (11,100 L mol^{-1} cm^{-1}). The photon flux can be calculated based on the following equation:

$$\text{photon flux} = \text{mol } Fe^{2+} / \Phi \times t \times f \qquad \text{...............(eq-2)}$$

where Φ is the quantum yield for the ferrioxalate actinometer (1.01 at λ = 440 nm)[10], t is the irradiation time (90 s), and f is the fraction of light absorbed at λ = 436 nm (0.99798).

mol Fe^{2+}= 0.00224 L x 0.2872452 / 1.000 cm x 11,100 L mol^{-1} cm^{-1} = 5.80×10^{-8} mol

photon flux = 5.80×10^{-8} mol / 1.01 x 90.0 s x 0.99798 = 6.39×10^{-10} einstein s^{-1}

The average photon flux was 6.39×10^{-10} einstein s^{-1}.

Determination of fraction of light absorbed at 436 nm for the ferrioxalate solution:

The absorbance of the above ferrioxalate solution at 436 nm was measured to be 2.764533. The fraction of light absorbed (f) by this solution was calculated using eq 3, where A is the measured absorbance at 440 nm.

$$f = 1 - 10^{-A} \qquad \text{...............(eq-3)}$$

$$f = 1.00.$$

Determination of the reaction quantum yield:

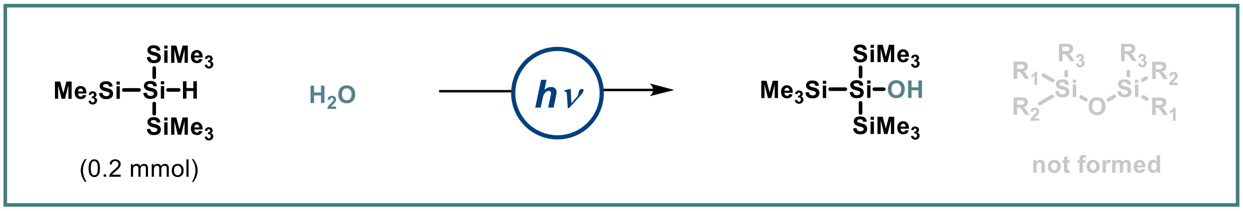

An oven-dried screw capped reaction tube equipped with magnetic stir-bar was capped with a screw cap with rubber septum and purged with oxygen for three times. Then, the cap was wrapped with a teflon. Subsequently, water (1.0 mmol, 5 *eq*.) and 1 mL of dichloromethane was added to the reaction tube. Then silane (0.2 mmol, 1 *eq*.) was added to the reaction solvent. Finally, the reaction tube was placed 3 cm away from 34 W Kessil lamp with stirring (1000 rpm) for 1 h. The temperature was maintained by cooling with two fans. After irradiation, the yield was determined to be 7% (1.4×10^{-5} mol) by ^{1}H NMR using TMB as an internal standard.

$$\Phi = \text{mol product} / \text{flux} \times t \times f \quad ....(\text{eq-4})$$

The reaction quantum yield (Φ) was determined using eq-4 where the photon flux is 6.39×10^{-10} einstein s^{-1} (determined from eq-2 as described above), *t* is the reaction time (3600 s) and f is the fraction of incident light absorbed by the reaction mixture is 1.00 determined using eq 3.

$$\Phi = 1.4 \times 10^{-5} / 6.39 \times 10^{-10} \times 3600 \times 1.00 = 6.08$$

The reaction quantum yield (Φ) was thus determined to be $\Phi = 6.08$.

3.6. Evidence of Cl anion formation:

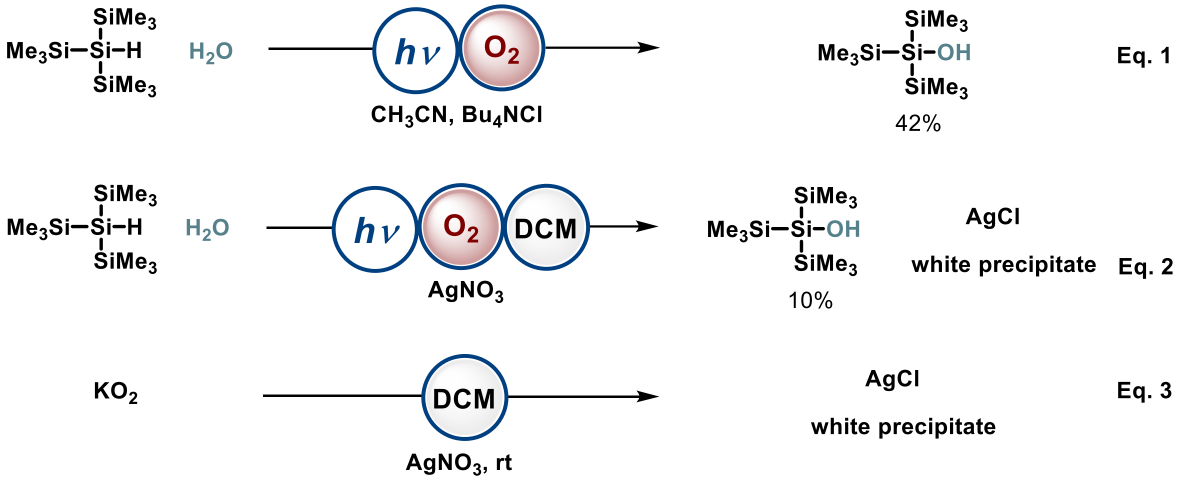

Eq. 1. Following the general procedure for silanol synthesis, the control experiment was performed with Bu_4NCl (1.5 *eq*.) in CH_3CN solvent in presence of 34 W Kessil lamp for 28 h. It was observed that the reaction proceeds with 42% yield indicates the formation of Cl anion in the catalytic cycle.

Eq. 2. Following the general procedure for silanol synthesis, the control experiment was performed with $AgNO_3$ (2.0 *eq*.) with DCM as the solvent in presence of 34 W Kessil lamp for 28 h. It was observed that the reaction proceeds with 10% yield indicates that the Cl anion forms in the medium reacts with silver nitrate to give white precipitate of silver chloride.

Eq. 3. Following the general procedure for silanol synthesis, the control experiment was performed with KO_2 (1.5 *eq*.) and $AgNO_3$ (2.0 *eq*.) with DCM as solvent in room temperature for 28 h. The outcome of the reaction also shows a characteristic white precipitate of silver chloride.

All three above-mentioned control experiments suggest the formation of Cl anion in the reaction. It is worthy to mention that when we performed the reaction with tetrabutylammonium bromide, tetrabutylammonium iodide and tetrabutylammonium fluoride shows yield of 7, 8 and 3% respectively.

3.7. AgCl detection by powder X-Ray diffraction:

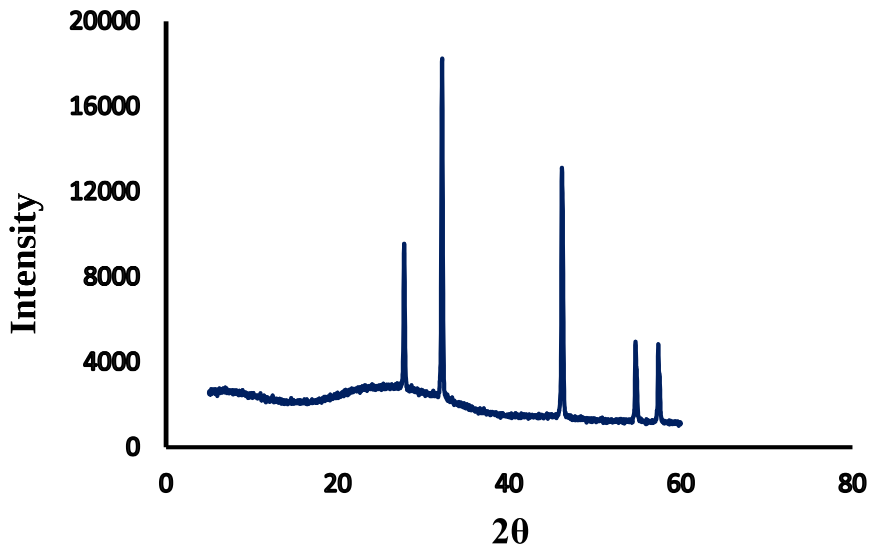

Supplementary Fig 4: AgCl formation analyzed by powder X-Ray diffraction

The silver chloride precipitate was further characterized by powder X-Ray diffraction (Fig S4).

3.8. Source of hydroxyl group in the reaction:

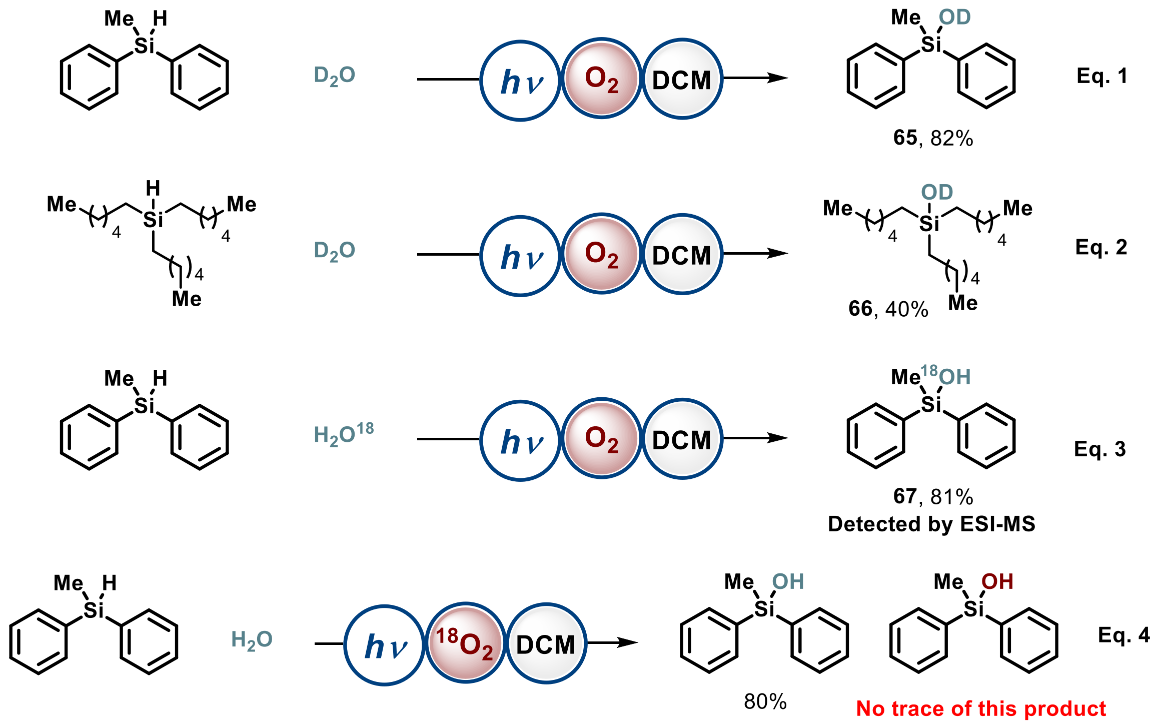

Eqn. 1 and **Eqn. 2**. Following the general procedure for silanol synthesis, the control experiment was performed with deuterated water in presence of 34 W Kessil lamp for 28 h. The deuterated

(**66-67**) silanol was observed and confirming that H_2O is the source of the hydroxyl group in the reaction.

Eqn. 3. Another experiment was done with H_2O^{18} in the presence of a 34 W Kessil lamp for 28 h. The ^{18}O-labelled (**68**) product again confirmed that H_2O is the source of the hydroxyl group in the silanol products. The ^{18}O-labeled (**68**) product was further detected by mass analysis (Fig S5).

Eqn. 4. A similar experiment was done with $^{18}O_2$ in the presence of a 34 W Kessil lamp for 28 h. The outcome of the result shows normal silanol product. There is no formation of the ^{18}O-labelled (**68**) product. This further consolidate that H_2O is the source of the hydroxyl group in the silanol products, unlikely from the oxygen present in the reaction medium.

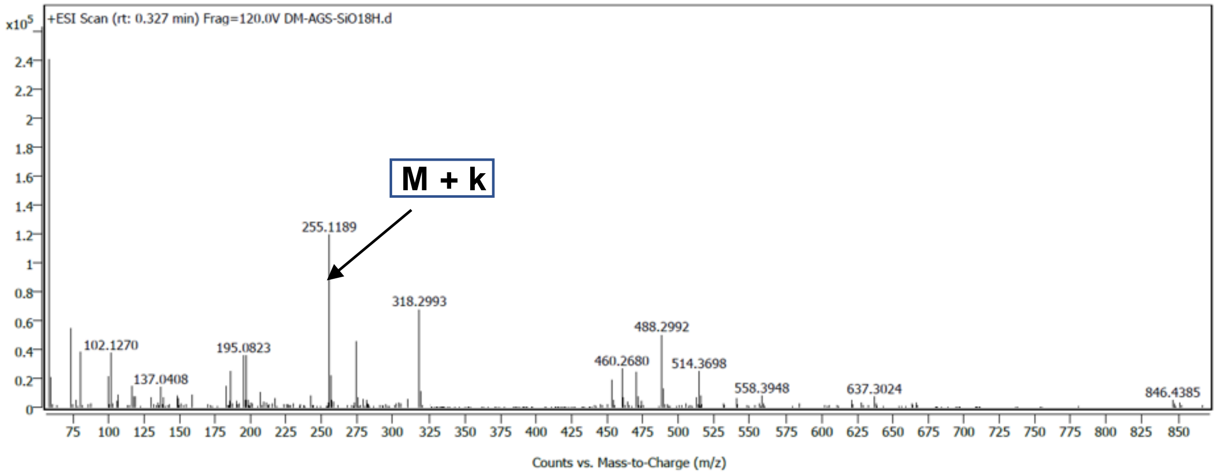

Supplementary Fig 5: ESI-MS analysis for ^{18}O incorporation experiment

4. On/Off experiment:

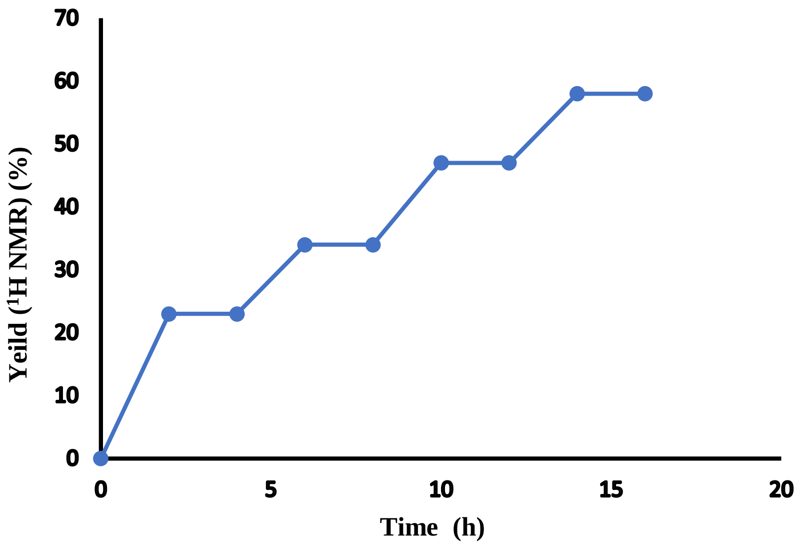

Supplementary Fig 6: Effect of visible light irradiation

According to the general procedure for silanol synthesis, a reaction containing TMB (Trimethoxy-benzene) as internal standard was set up and placed in presence of 34 W Kessil lamp for 12 h. The reaction was sequentially stirred under visible light irradiation and in the absence of light. Every two hours an aliquot of 50 µL was taken *via* syringe and analyzed by ^{1}H NMR spectroscopy using TMB as internal standard. After a total of 12 h the determined yields were plotted against the reaction time.

5.1. Hammett plot to show the electronic effects on the silanol synthesis:

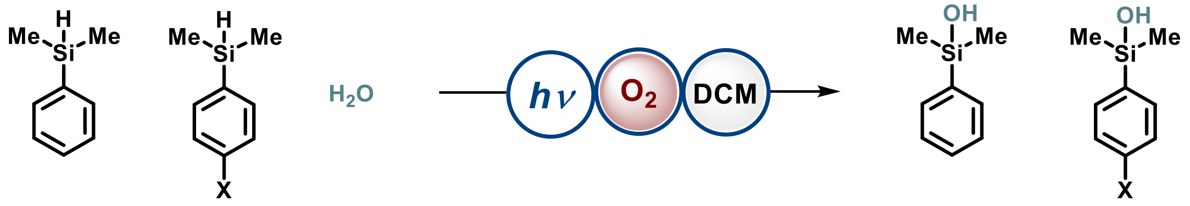

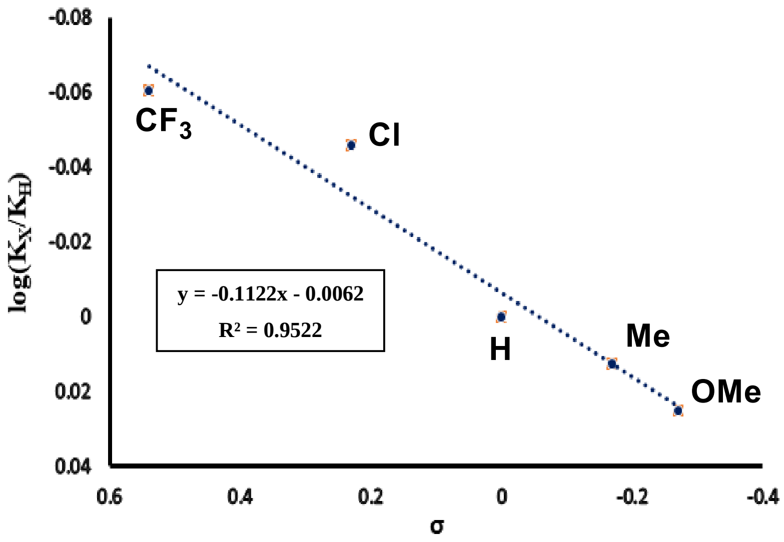

Supplementary Fig 7: Hammett Plot of log(K_X/K_H) vs. σ

Following the general procedure for silanol synthesis, the reaction of dimethylphenylsilane (1.0 *eq*.) and *p*-substituted dimethylphenylsilane (1.0 *eq*.) was performed in presence of 34 W Kessil lamp for 28 h. Upon completion of the reaction the crude product analyzed by ^{1}H NMR spectroscopy using TMB as internal standard. The result is obtained based on the average for 3 trials. The K_X/K_H was estimated based on the remaining substrates.

The Hammett plot (log(k_X/k_H) versus σ) exhibited linear correlation plot and good linearity implies that oxidation proceeds through a single radical mechanism.

Data's for Plotting Hammett Plot:[11-12]

Substituents	σ	Log (k_X/k_H)
OMe	-0.27	0.0253
Me	-0.17	0.0128
H	0	0
Cl	0.23	-0.0457
CF_3	0.54	-0.0604

5.2. Parallel kinetic isotope effect experiment:

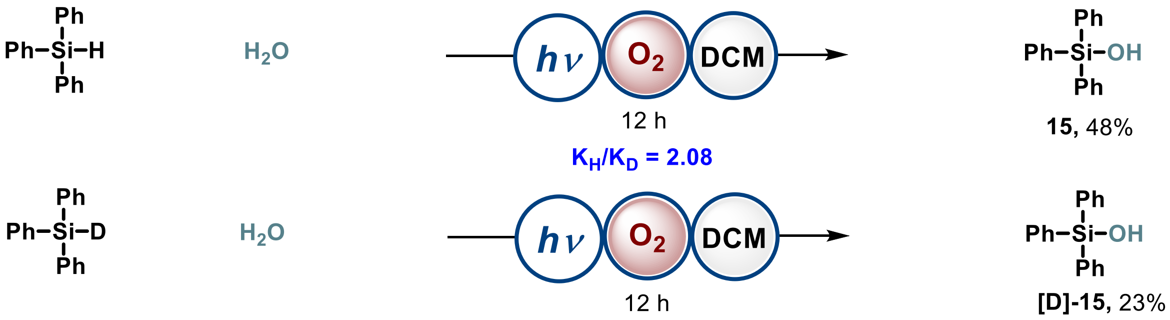

Following the general procedure for silanol synthesis, two independent reactions was performed parallelly with normal triphenyl silane (1.0 *eq*.) and deuterated triphenyl silane (1.0 *eq*.) in presence of 34 W Kessil lamp for 28 h. Upon completion of the reaction the crude products were isolated by column chromatography using silica (100-200 mesh size) and petroleum ether/ethyl acetate as the eluent. The ratio was determined from the yield and it was found in 2.08:1 ratio.

The value of k_H/k_D = 2.08 from two parallel reactions indicate that Si–H bond cleavage is involved in the rate-determining step of this photochemical.

5.3. Competition experiment:

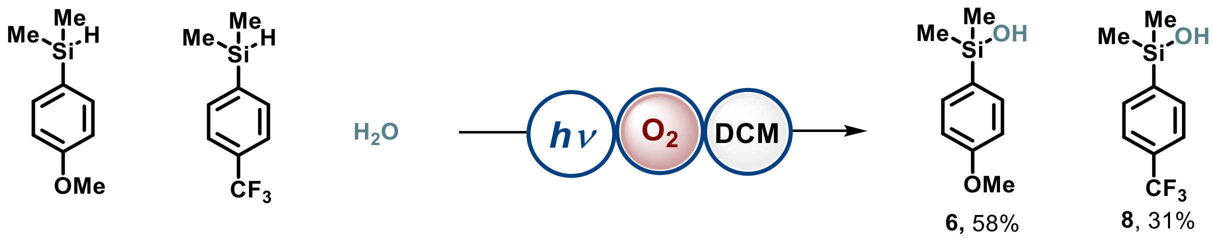

Following the general procedure for silanol synthesis, equimolar mixture of (4-Methoxyphenyl)dimethylsilane (1.0 *eq*.) and (4-trifluorophenyl)dimethylsilane (1.0 *eq*.) was taken

and reaction was performed in presence of 34 W Kessil lamp for 28 h. Upon completion of the reaction the crude products were isolated by column chromatography using silica (100-200 mesh size) and petroleum ether/ethyl acetate as the eluent. The yield was determined for both the substrates and it indicates that electron-rich silanes to be preferentially converted to the corresponding silanol products.

6. Characterization data of silanol products:

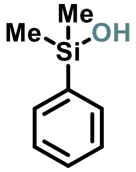

Dimethyl(phenyl)silanol (1):

Physical appearance: Colorless oil.

Column material: 100-200 mesh silica

Eluent: petroleum ether/ethyl acetate (95/5).

Yield: 72%

^{1}H NMR (400 MHz, $CDCl_3$) δ 7.61 (dd, *J* = 7.5, 1.9 Hz, 2H), 7.40 (qd, *J* = 5.7, 3.6 Hz, 3H), 2.81 (s, 1H), 0.40 (s, 6H). **^{13}C NMR (101 MHz, $CDCl_3$)** δ 139.30, 133.25, 129.77, 128.05, 0.12. **LRMS**: Calculated mass for $C_8H_{12}NaOSi$ $[M+Na]^+$ 175.0550, observed mass: 175.0543. **IR**: 3283 (OH).

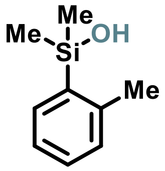

Dimethyl(*o*-tolyl)-silanol (2):[11]

Physical appearance: Colorless oil.

Column material: 100-200 mesh silica

Eluent: petroleum ether/ethyl acetate (97/3).

Yield: 81%

^{1}H NMR (400 MHz, $CDCl_3$) δ 7.54–7.56 (m, 1H), 7.29–7.33 (m, 1H), 7.18–7.22 (m, 2H), 2.52 (s, 3H), 2.04 (s,1H), 0.46 (s, 6H). **^{13}CNMR (101 MHz, $CDCl_3$)** δ 143.26, 137.32, 134.06, 129.90, 129.84, 124.94, 22.75, 0.19. **LRMS**: Calculated mass for $C_9H_{15}OSi[M+H]^+$: 167.0892., observed mass: 167.0890. **IR**: 3275 (OH).

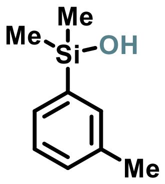

Dimethyl(*m*-tolyl)silanol (3):[11]

Physical appearance: Colorless oil.

Column material: 100-200 mesh silica

Eluent: petroleum ether/ethyl acetate (97/3).

Yield: 78%

^{1}H NMR (400 MHz, $CDCl_3$) δ 7.39–7.42 (m, 2H), 7.28–7.32 (m, 1H), 7.22–7.24 (m, 1H), 2.29 (s, 3H), 2.01 (s,1H), 0.41 (s, 6H). **^{13}CNMR (101 MHz, $CDCl_3$)** δ 138.61, 136.95, 133.36, 130.10, 129.69, 127.51, 21.16, 0.20. **LRMS**: Calculated mass for $C_9H_{15}OSi[M+H]^+$: 167.0892., observed mass: 167.0887. **IR**: 3281 (OH).

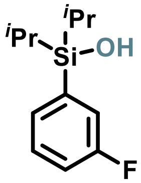

(3-fluorophenyl)diisopropylsilanol (4):

Physical appearance: Colorless oil.

Column material: 100-200 mesh silica

Eluent: petroleum ether/ethyl acetate (95/5).

Yield: 70%

^{1}H NMR (400 MHz, $CDCl_3$) δ 7.38 – 7.29 (m, 2H), 7.24 (dd, *J* = 4.7, 4.3 Hz, 1H), 7.07 (dddd, *J* = 9.2, 7.8, 2.7, 1.4 Hz, 1H), 1.86 (s, 1H), 1.26 – 1.14 (m, 2H), 1.05 (d, *J* = 7.3 Hz, 6H), 0.97 (d, *J* = 7.4 Hz, 6H). **^{13}C NMR (101 MHz, $CDCl_3$)** δ 164.01 (d, *J* = 244.5 Hz), 138.81 (d, *J* = 7.8 Hz), 129.79, 129.76, 129.65 (d, *J* = 3.8 Hz), 120.68, 120.49, 116.48 (d, *J* = 19.5 Hz), 17.27, 17.03, 12.57. **^{19}F NMR (471 MHz, $CDCl_3$)** δ -113.77. **HRMS (ESI)**: Calculated mass for $C_{12}H_{20}FOSi$ $[M+H]^+$: 227.1267., observed mass: 227.1264. **IR**: 3290 (OH).

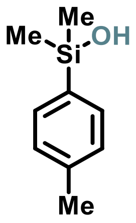

Dimethyl(*p*-tolyl)silanol (5):

Physical appearance: Sticky oil.

Column material: 100-200 mesh silica

Eluent: petroleum ether/ethyl acetate (94/6).

Yield: 74%

^{1}H NMR (400 MHz, $CDCl_3$) δ 7.51 (d, *J* = 7.8 Hz, 2H), 7.23 (d, *J* = 7.5 Hz, 2H), 2.39 (s, 3H), 2.23 (s, 1H), 0.41 (d, *J* = 3.3 Hz, 6H).**^{13}C NMR (101 MHz, $CDCl_3$)** δ 139.77, 135.76, 133.32, 128.91, 21.69, 0.21. **LRMS**: Calculated mass for $C_9H_{15}OSi$ $[M+H]^+$: 167.0892; observed mass: 167.0890. **IR**: 3422 (OH).

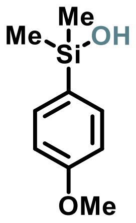

(4-methoxyphenyl)dimethylsilanol (6):

Physical appearance: Light yellow oil.

Column material: 100-200 mesh silica

Eluent: petroleum ether/ethyl acetate (92/8).

Yield: 76%

^{1}H NMR (400 MHz, $CDCl_3$) δ 7.46 (d, *J* = 8.6 Hz, 2H), 6.90 (d, *J* = 8.6 Hz, 2H), 3.82 (s, 3H), 0.30 (s, 6H). **^{13}C NMR (101 MHz, $CDCl_3$)** δ 160.74, 134.76, 131.25, 113.61, 55.23, 1.20. **HRMS (ESI)**: Calculated mass for $C_9H_{15}O_2Si$ $[M+H]^+$: 183.0841; observed mass: 183.0840. **IR**: 3452 (OH).

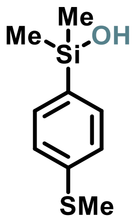

Dimethyl(4-(methylthio)phenyl)silanol (7):

Physical appearance: Yellow oil.

Column material: 100-200 mesh silica

Eluent: petroleum ether/ethyl acetate (93/7).

Yield: 77%

^{1}H NMR (500 MHz, $CDCl_3$) δ 7.46 (d, *J* = 8.1 Hz, 2H), 7.23 (t, *J* = 11.7 Hz, 2H), 2.52 (s, 1H), 2.46 (s, 3H), 0.35 (s, 6H). **^{13}C NMR (126 MHz, $CDCl_3$)** δ 140.65, 135.23, 133.65, 125.74, 15.42, 0.17. **HRMS (ESI)**: Calculated mass for $C_9H_{15}OSSi$ $[M+H]^+$: 199.0613; observed mass: 199.0610. **IR**: 3289 (OH).

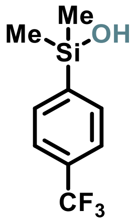

Dimethyl(4-(trifluoromethyl)phenyl)silanol (8):

Physical appearance: Colorless oil.

Column material: 100-200 mesh silica

Eluent: petroleum ether/ethyl acetate (94/6).

Yield: 62%

^{1}H NMR (500 MHz, $CDCl_3$) δ 7.71 (d, *J* = 7.8 Hz, 2H), 7.62 (d, *J* = 7.9 Hz, 2H), 2.04 (s, 1H), 0.43 (s, 6H). **^{13}C NMR (126 MHz, $CDCl_3$)** δ 144.10, 133.57, 131.61 (q, *J* = 32.1 Hz), 125.46 (q, *J* = 3.8 Hz), 124.66, 124.63, 123.29 (q, *J* = 270.5 Hz), 0.19. **^{19}F NMR (471 MHz, $CDCl_3$)** δ -63.00. **HRMS (ESI)**: Calculated mass for $C_9H_{12}F_3OSi$ $[M+H]^+$: 221.0610., observed mass: 221.0604. **IR**: 3458 (OH).

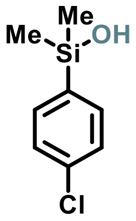

(4-Chlorophenyl)dimethylsilanol (9):[3]

Physical appearance: Colorless oil.

Column material: 100-200 mesh silica

Eluent: petroleum ether/ethyl acetate (90/10).

Yield: 65%

^{1}H NMR (500 MHz, CDCl$_3$) δ 7.50 (d, *J* = 8.0 Hz, 2H), 7.35 (d, *J* = 8.0 Hz, 2H), 2.39 (s, 1H), 0.38 (s, 6H). **^{13}C NMR (126 MHz, CDCl$_3$)** δ 137.62, 136.20, 134.72, 128.39, 0.26. **HRMS (ESI)**: Calculated mass for $C_8H_{11}ClNaOSi$ $[M+Na]^+$: 209.0165., observed mass: 209.0162. **IR**: 3446 (OH).

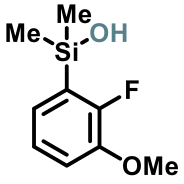

(2-Fluoro-3-methoxyphenyl)dimethylsilanol (10):

Physical appearance: Sticky oil.

Column material: 100-200 mesh silica

Eluent: petroleum ether/ethyl acetate (90/10).

Yield: 74%

^{1}H NMR (500 MHz, CDCl$_3$) δ 7.09 (t, *J* = 7.6 Hz, 1H), 7.04 – 6.98 (m, 2H), 3.88 (s, 3H), 2.45 (s, 1H), 0.44 (s, 6H). **^{13}C NMR (126 MHz, CDCl$_3$)** δ 157.28 (d, *J* = 244.5 Hz), 155.38, 147.30, 147.19 (d, *J* = 7.8 Hz), 130.51, 126.41, 126.19, 125.66, 125.58, 124.64, 124.61, 115.34 (d, *J* = 19.5 Hz), 56.33, 0.71, 0.70. **^{19}F NMR (471 MHz, CDCl$_3$)** δ -124.96. **HRMS (ESI)**: Calculated mass for $C_9H_{14}FO_2Si$ $[M+H]^+$: 201.0747., observed mass: 201.0741. **IR**: 3590 (OH).

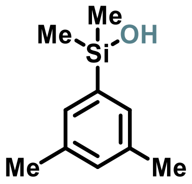

(3,5-Dimethylphenyl)dimethylsilanol (11):[1]

Physical appearance: Colorless oil.

Column material: 100-200 mesh silica

Eluent: petroleum ether/ethyl acetate (94/6).

Yield: 82%

^{1}H NMR (400 MHz, CDCl$_3$) δ 7.28 (s, 2H), 7.14 (s, 1H), 2.40 (s, 6H); 0.45 (s, 6H). **^{13}C NMR (101 MHz, CDCl$_3$)** δ 139.20, 137.42, 131.55, 130.90, 21.54, 0.25. **HRMS (ESI)**: Calculated mass for $C_{10}H_{17}OSi$ $[M+H]^+$: 181.1049., observed mass: 181.1047. **IR**: 3275 (OH).

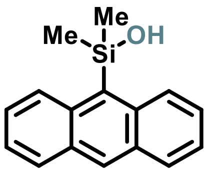

Anthracen-9-yldimethylsilanol (12):

Physical appearance: Yellow oil.

Column material: 100-200 mesh silica

Eluent: petroleum ether/ethyl acetate (88/12).

Yield: 80%

^{1}H NMR (400 MHz, CDCl$_3$) δ 8.72 – 8.65 (m, 2H), 8.49 (s, 1H), 8.05 – 7.98 (m, 2H), 7.51 – 7.44 (m, 4H), 2.41 (s, 1H), 0.80 (s, 6H). **^{13}C NMR (101 MHz, CDCl$_3$)** δ 137.01, 131.88, 130.69, 129.67, 128.36, 126.42, 125.54, 124.84, 0.50. **HRMS (ESI)**: Calculated mass for $C_{16}H_{17}OSi$ $[M+H]^+$: 253.1049., observed mass: 253.1040. **IR**: 3235 (OH).

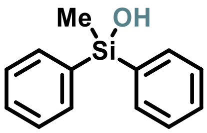

Methyldiphenylsilanol (13):

Physical appearance: Colorless liquid.

Column material: 100-200 mesh silica

Eluent: petroleum ether/ethyl acetate (95/5).

Yield: 86%

^{1}H NMR (500 MHz, CDCl$_3$) δ 7.68 – 7.64 (m, 4H), 7.48 (dd, *J* = 8.5, 6.2 Hz, 2H), 7.42 (t, *J* = 7.3 Hz, 4H), 3.59 (s, 1H), 0.68 (s, 3H). **^{13}C NMR (126 MHz, CDCl$_3$)** δ 137.21, 134.17, 129.96, 128.03, -1.19. **HRMS (ESI)**: Calculated mass for $C_{13}H_{15}OSi$ $[M+H]^+$: 215.0892., observed mass: 215.0890. **IR**: 3389 (OH).

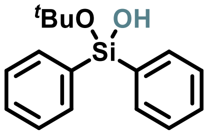

Tert-butoxydiphenylsilanol (14):

Physical appearance: Colorless liquid.

Column material: 100-200 mesh silica

Eluent: petroleum ether/ethyl acetate (85/15).

Yield: 68%

^{1}H NMR (500 MHz, CDCl$_3$) δ 7.73 – 7.69 (m, 4H), 7.42 (t, *J* = 7.3 Hz, 2H), 7.37 (t, *J* = 7.3 Hz, 4H), 2.82 (s, 1H), 1.36 (s, 9H). **^{13}C NMR (126 MHz, CDCl$_3$)** δ 136.26, 134.75, 130.11, 127.91, 74.39, 32.30. **HRMS (ESI)**: Calculated mass for $C_{16}H_{21}OSi$ [M+H]$^+$: 273.1311., observed mass: 273.1304. **IR**: 3398 (OH).

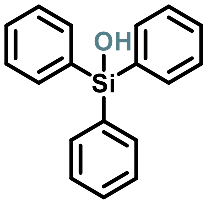

Triphenylsilanol (15):

Physical appearance: Colorless liquid.

Column material: 100-200 mesh silica

Eluent: petroleum ether/ethyl acetate (90/10).

Yield: 92%

^{1}H NMR (400 MHz, CDCl$_3$) δ 7.67 – 7.63 (m, 6H), 7.49 – 7.44 (m, 3H), 7.40 (t, *J* = 7.2 Hz, 6H), 2.53 (s, 1H). **^{13}C NMR (101 MHz, CDCl$_3$)** δ 135.33, 135.19, 130.30, 128.12. **HRMS (ESI)**: Calculated mass for $C_{18}H_{17}OSi$ [M+H]$^+$: 277.1049., observed mass: 277.1046. **IR**: 3292 (OH).

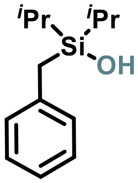

Benzyldiisopropylsilanol (16):

Physical appearance: Colorless liquid.

Column material: 100-200 mesh silica

Eluent: petroleum ether/ethyl acetate (95/5).

Yield: 72%

^{1}H NMR (500 MHz, CDCl$_3$) δ 7.23 (t, *J* = 7.6 Hz, 2H), 7.10 (dd, *J* = 16.3, 7.6 Hz, 3H), 2.21 (s, 2H), 1.54 (s, 1H), 1.15 – 0.83 (m, 14H). **^{13}C NMR (126 MHz, CDCl$_3$)** δ 139.49, 128.65, 128.58, 124.47, 21.57, 17.48, 17.42, 12.90. **HRMS (ESI)**: Calculated mass for $C_{13}H_{23}OSi$ [M+H]$^+$: 223.1518., observed mass: 223.1517. **IR**: 3403 (OH).

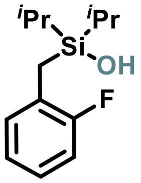

(2-Fluorobenzyl)diisopropylsilanol (17):

Physical appearance: Sticky oil.

Column material: 100-200 mesh silica

Eluent: petroleum ether/ethyl acetate (92/8)

Yield: 61%

^{1}H NMR (400 MHz, CDCl$_3$) 7.18 – 7.12 (m, 1H), 7.11 – 7.04 (m, 1H), 7.00 (dtd, *J* = 9.6, 7.7, 1.6 Hz, 2H), 2.20 (d, *J* = 1.8 Hz, 2H), 1.74 (s, 1H), 1.02 (d, *J* = 5.2 Hz, 14H). **^{13}C NMR (101 MHz, CDCl$_3$)** δ 161.83, 159.43, 131.00, 130.95, 126.78, 126.61, 126.11, 126.03, 124.21, 124.18, 115.46, 115.24, 17.32, 17.27, 14.53, 14.51, 13.08. **^{19}F NMR (471 MHz, CDCl$_3$)** δ -116.98. **HRMS (ESI)**: Calculated mass for $C_{13}H_{22}FOSi$ $[M+H]^+$: 241.1424., observed mass: 241.1422. **IR**: 3445 (OH).

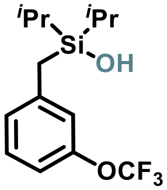

Diisopropyl(3-(trifluoromethoxy)benzyl)silanol (18):

Physical appearance: Yellow solid.

Column material: 100-200 mesh silica

Eluent: petroleum ether/ethyl acetate (90:10)

Yield: 65%

^{1}H NMR (400 MHz, CDCl$_3$) δ 7.23 (t, *J* = 7.9 Hz, 1H), 7.04 (d, *J* = 7.7 Hz, 1H), 6.99 (s, 1H), 6.95 (dd, *J* = 8.2, 1.0 Hz, 1H), 2.23 (s, 2H), 1.60 (s, 1H), 0.99 (d, *J* = 1.8 Hz, 14H). **^{13}C NMR (101 MHz, CDCl$_3$)** δ 149.58, 142.14, 129.71, 127.06, 122.01, 121.14, 119.46, 116.91, 21.85, 17.37, 17.33, 12.87. **^{19}F NMR (471 MHz, CDCl$_3$)** δ -57.72. **HRMS (ESI)**: Calculated mass for $C_{14}H_{22}F_3O_2Si$ $[M+H]^+$: 307.1341., observed mass: 307.1338. **IR**: 3580 (OH).

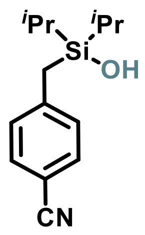

4-((Hydroxydiisopropylsilyl)methyl)benzonitrile (19):

Physical appearance: Sticky oil.

Column material: 100-200 mesh silica

Eluent: petroleum ether/ethyl acetate (90:10)

Yield: 70%

^{1}H NMR (500 MHz, $CDCl_3$) δ 7.46 (d, J = 8.1 Hz, 2H), 7.21 (d, J = 8.1 Hz, 2H), 2.27 (s, 2H), 2.21 (s, 1H), 1.11 – 0.85 (m, 14H). **^{13}C NMR (126 MHz, $CDCl_3$)** δ 146.56, 132.15, 129.34, 119.43, 107.65, 23.07, 17.34, 17.30, 12.87. **HRMS (ESI)**: Calculated mass for $C_{14}H_{22}NOSi$ $[M+H]^+$: 248.1471., observed mass: 248.1468. **IR**: 3386 (OH).

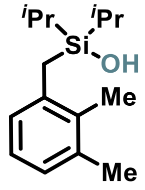

(2,3-dimethylbenzyl)diisopropylsilanol (20):

Physical appearance: Sticky liquid.

Column material: 100-200 mesh silica

Eluent: petroleum ether/ethyl acetate (92:8)

Yield: 81%

^{1}H NMR (500 MHz, $CDCl_3$) δ 6.94 (tt, J = 14.9, 7.3 Hz, 3H), 2.27 (s, 3H), 2.23 (s, 2H), 2.20 (s, 3H), 1.39 (s, 1H), 1.10 – 0.98 (m, 14H). **^{13}C NMR (101 MHz, $CDCl_3$)** δ 137.80, 137.19, 133.87, 127.26, 126.53, 125.45, 21.23, 19.26, 17.62, 17.48, 16.29, 13.26. **HRMS (ESI)**: Calculated mass for $C_{15}H_{26}NaOSi$ $[M+Na]^+$: 273.1651., observed mass: 273.1650. **IR**: 3210 (OH).

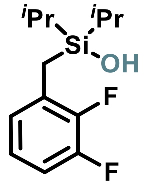

(2,3-Difluorobenzyl)diisopropylsilanol (21):

Physical appearance: Colorless oil.

Column material: 100-200 mesh silica

Eluent: petroleum ether/ethyl acetate (90:10)

Yield: 55%

^{1}H NMR (400 MHz, CDCl$_3$) δ 7.00 – 6.82 (m, 3H), 2.22 (d, *J* = 2.1 Hz, 2H), 1.77 (s, 1H), 1.02 (dd, *J* = 7.4, 2.7 Hz, 14H). **^{13}C NMR (101 MHz, CDCl$_3$)** δ 152.32, 152.19, 149.87, 149.80, 149.73, 149.67, 147.39, 147.26, 129.56, 129.43, 125.58, 125.55, 125.51, 123.88, 123.84, 123.81, 123.77, 113.40, 113.23, 17.28, 17.23, 14.59, 14.57, 14.55, 13.06. **^{19}F NMR (471 MHz, CDCl$_3$)** δ -138.80, -138.84, -142.53, -142.58. **HRMS (ESI)**: Calculated mass for $C_{13}H_{20}NaF_2OSi$ $[M+Na]^+$: 281.1149., observed mass: 281.1142. **IR**: 3520 (OH).

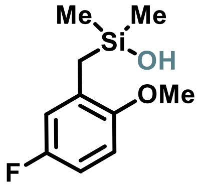

(5-Fluoro-2-methoxybenzyl)dimethylsilanol (22):

Physical appearance: Colorless oil.

Column material: 100-200 mesh silica

Eluent: petroleum ether/ethyl acetate (86:14)

Yield: 62%

^{1}H NMR (500 MHz, CDCl$_3$) δ 6.79 – 6.74 (m, 1H), 6.74 – 6.68 (m, 2H), 3.77 (s, 3H), 2.14 (d, *J* = 30.3 Hz, 2H), 0.06 (dd, *J* = 10.3, 2.4 Hz, 3H), 0.01 (s, 3H), -0.00 (s, 1H). **^{13}C NMR (101 MHz, CDCl$_3$)** δ 158.00, 156.11, 152.80, 152.78, 130.46, 130.40, 116.53, 116.34, 110.90, 110.72, 110.55, 110.48, 55.60, 22.57, 22.50, 1.30, 0.36. **^{19}F NMR (471 MHz, CDCl$_3$)** δ -124.95, -124.98. **HRMS (ESI)**: Calculated mass for $C_{10}H_{16}FO_2Si$ $[M+H]^+$: 215.0904., observed mass: 215.0897. **IR**: 3414 (OH).

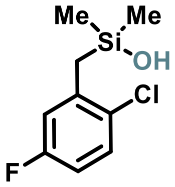

(2-Chloro-5-fluorobenzyl)dimethylsilanol (23):

Physical appearance: Sticky oil.

Column material: 100-200 mesh silica

Eluent: petroleum ether/ethyl acetate (90:10)

Yield: 59%

^{1}H NMR (500 MHz, CDCl$_3$) δ 7.28 (d, *J* = 8.6 Hz, 1H), 6.85 (dd, *J* = 9.4, 2.2 Hz, 1H), 6.76 (td, *J* = 8.5, 2.4 Hz, 1H), 2.36 (s, 2H), 2.22 (s, 1H), 0.18 (s, 6H). **^{13}C NMR (126 MHz, CDCl$_3$)** δ 162.37, 160.42, 139.96, 139.90, 130.70, 130.63, 127.68, 127.66, 116.70, 116.52, 113.06, 112.87, 26.42, -0.11. **^{19}F NMR (471 MHz, CDCl$_3$)** δ -116.01. **HRMS (ESI)**: Calculated mass for $C_9H_{13}ClFOSi$ $[M+H]^+$: 219.0408., observed mass: 219.0408. **IR**: 3500 (OH).

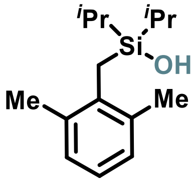

(2,6-Dimethylbenzyl)diisopropylsilanol (24):

Physical appearance: Colourless oil.

Column material: 100-200 mesh silica

Eluent: petroleum ether/ethyl acetate (93:7)

Yield: 79%

^{1}H NMR (400 MHz, CDCl$_3$) δ 6.99 (d, *J* = 7.4 Hz, 2H), 6.92 (dd, *J* = 8.5, 6.3 Hz, 1H), 2.32 (s, 6H), 2.23 (s, 2H), 1.47 (s, 1H), 1.06 (d, *J* = 5.7 Hz, 6H), 1.03 – 0.96 (m, 2H), 0.95 (d, *J* = 5.5 Hz, 6H). **^{13}C NMR (101 MHz, CDCl$_3$)** δ 137.08, 135.45, 128.23, 124.23, 21.53, 17.54, 17.36, 16.31, 14.03. **HRMS (ESI)**: Calculated mass for $C_{15}H_{27}OSi$ $[M+H]^+$: 251.1831., observed mass: 251.1829. **IR**: 3240 (OH).

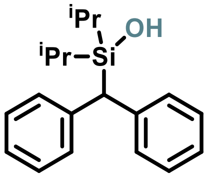

Benzhydryldiisopropylsilanol (25):

Physical appearance: Colorless oil.

Column material: 100-200 mesh silica

Eluent: petroleum ether/ethyl acetate (90:10)

Yield: 56%

^{1}H NMR (500 MHz, CDCl$_3$) δ 7.40 (d, *J* = 7.6 Hz, 4H), 7.30 (t, *J* = 7.6 Hz, 4H), 7.18 (t, *J* = 7.3 Hz, 2H), 4.11 (s, 1H), 0.98 (ddd, *J* = 17.5, 13.1, 7.2 Hz, 14H). **^{13}C NMR (126 MHz, CDCl$_3$)** δ 143.43, 129.06, 128.70, 125.61, 40.53, 19.31, 19.11, 10.97. **HRMS (ESI)**: Calculated mass for $C_{19}H_{27}OSi$ [M+H]$^+$: 299.1831., observed mass: 299.1830. **IR**: 3287 (OH).

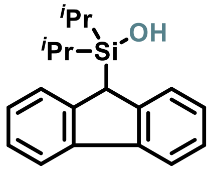

(9H-fluoren-9-yl)diisopropylsilanol (26):

Physical appearance: Colorless oil.

Column material: 100-200 mesh silica

Eluent: petroleum ether/ethyl acetate (90:10)

Yield: 58%

^{1}H NMR (500 MHz, CDCl$_3$) δ 7.89 (d, *J* = 7.5 Hz, 2H), 7.64 (d, *J* = 7.5 Hz, 2H), 7.38 (t, *J* = 7.4 Hz, 2H), 7.32 (td, *J* = 7.5, 1.1 Hz, 2H), 4.16 (s, 1H), 1.83 (s, 1H), 0.94 – 0.89 (m, 2H), 0.87 (d, *J* = 6.5 Hz, 6H), 0.70 (d, *J* = 7.0 Hz, 6H). **^{13}C NMR (126 MHz, CDCl$_3$)** δ 144.87, 141.06, 126.44, 125.73, 124.59, 120.23, 40.49, 17.52, 17.20, 12.21. **HRMS (ESI)**: Calculated mass for $C_{19}H_{24}NaOSi$ [M+Na]$^+$: 319.1494., observed mass: 319.1494. **IR**: 3283 (OH).

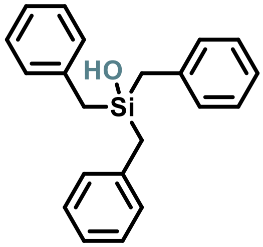

Tribenzylsilanol (27):

Physical appearance: Colorless oil.

Column material: 100-200 mesh silica

Eluent: petroleum ether/ethyl acetate (88:12)

Yield: 75%

^{1}H NMR (400 MHz, CDCl$_3$) δ 7.32 – 7.29 (m, 6H), 7.23 – 7.15 (m, 3H), 7.12 – 7.00 (m, 6H), 2.25 (s, 6H), 1.87 (s, 1H). **^{13}C NMR (126 MHz, CDCl$_3$)** δ 138.13, 128.75, 128.71, 124.79, 24.23. **HRMS (ESI)**: Calculated mass for $C_{21}H_{23}OSi$ $[M+H]^+$: 319.1518., observed mass: 319.1515. **IR**: 3210 (OH).

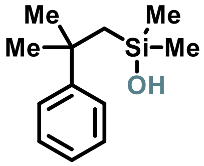

Dimethyl(2-methyl-2-phenylpropyl)silanol (28):

Physical appearance: Sticky liquid.

Column material: 100-200 mesh silica

Eluent: petroleum ether/ethyl acetate (85:15)

Yield: 48%

^{1}H NMR (500 MHz, CDCl$_3$) δ 7.42 (d, *J* = 7.8 Hz, 2H), 7.32 (t, *J* = 7.7 Hz, 2H), 7.19 (t, *J* = 7.3 Hz, 1H), 1.44 (s, 6H), 1.19 (d, *J* = 37.8 Hz, 2H), 1.15 (s, 1H), -0.06 (s, 6H). **^{13}C NMR (126 MHz, CDCl$_3$)** δ 150.91, 128.45, 125.91, 125.61, 37.01, 35.84, 32.59, 1.89. **HRMS (ESI)**: Calculated mass for $C_{12}H_{21}OSi$ $[M+H]^+$: 201.1362., observed mass: 201.1360. **IR**: 3210 (OH).

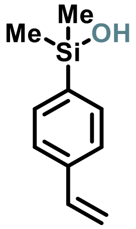

Dimethyl(4-vinylphenyl)silanol (29):

Physical appearance: Colorless liquid.

Column material: 100-200 mesh silica

Eluent: petroleum ether/ethyl acetate (93:7)

Yield: 60%

^{1}H NMR (500 MHz, CDCl$_3$) δ 7.56 (d, *J* = 7.8 Hz, 2H), 7.43 (d, *J* = 7.8 Hz, 2H), 6.73 (dd, *J* = 17.6, 10.9 Hz, 1H), 5.80 (d, *J* = 17.6 Hz, 1H), 5.29 (d, *J* = 10.9 Hz, 1H), 2.02 (s, 1H), 0.41 (s, 6H). **^{13}C NMR (126 MHz, CDCl$_3$)** δ 138.95, 138.84, 136.94, 133.53, 133.40, 127.75, 125.88, 114.77,

0.22. **HRMS (ESI)**: Calculated mass for $C_{10}H_{15}OSi$ $[M+H]^+$: 179.0892., observed mass: 179.0892. **IR**: 3276 (OH).

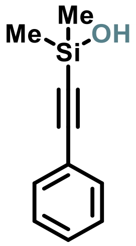

Dimethyl(phenylethynyl)silanol (30):[1]

Physical appearance: Colorless liquid.

Column material: 100-200 mesh silica

Eluent: petroleum ether/ethyl acetate (95:5)

Yield: 70%

^{1}H NMR (400 MHz, CDCl$_3$) δ 7.47-7.45 (m, 2H), 7.33-7.25 (m, 3H), 2.08 (s, 1H), 0.36 (s, 6H). **^{13}C NMR (100 MHz, CDCl$_3$)** δ 132.34, 129.19, 128.55, 122.74, 105.03, 92.87, 1.72. **HRMS (ESI)**: Calculated mass for $C_{10}H_{12}OSi$ $[M+H]^+$: 176.0892., observed mass: 176.0892. **IR**: 3448 (OH

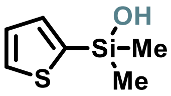

Dimethyl(thiophen-2-yl)silanol (31):[12]

Physical appearance: Colorless liquid.

Column material: 100-200 mesh silica

Eluent: petroleum ether/ethyl acetate (88:12)

Yield: 63%

^{1}H NMR (500 MHz, CDCl$_3$) δ 7.63 (d, J = 4.5 Hz, 1H), 7.34 (d, J = 3.0 Hz, 1H), 7.23 (dd, J = 4.2, 3.6 Hz, 1H), 2.43 (s, 1H), 0.42 (s, 6H). **^{13}C NMR (126 MHz, CDCl$_3$)** δ 138.84, 134.85, 131.26, 128.39, 1.12. **HRMS (ESI)**: Calculated mass for $C_6H_{11}OSSi$ $[M+H]^+$: 159.0300., observed mass: 159.0300. **IR**: 3230 (OH).

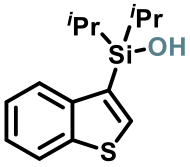

Benzo[b]thiophen-3-yldiisopropylsilanol (32):

Physical appearance: Yellowish oil.

Column material: 100-200 mesh silica

Eluent: petroleum ether/ethyl acetate (86:14)

Yield: 60%

^{1}H NMR (500 MHz, CDCl$_3$) δ 7.61 (d, *J* = 7.7 Hz, 1H), 7.53 (d, *J* = 8.2 Hz, 1H), 7.30 (t, *J* = 7.7 Hz, 1H), 7.23 (t, *J* = 7.4 Hz, 1H), 7.12 (s, 1H), 2.19 (s, 1H), 1.27 (dq, *J* = 14.3, 7.3 Hz, 2H), 1.12 (dd, *J* = 18.5, 7.4 Hz, 12H). **^{13}C NMR (126 MHz, CDCl$_3$)** δ 141.15, 138.62, 137.32, 125.05, 124.89, 122.81, 122.46, 105.91, 32.66, 29.86, 22.45, 14.03. **HRMS (ESI):** Calculated mass for $C_{14}H_{21}OSSi$ [M+H]$^+$: 265.1082; observed mass: 265.1076. **IR**: 3260 (OH).

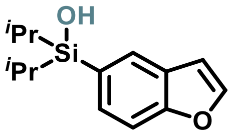

Benzofuran-5-yldiisopropylsilanol (33):

Physical appearance: Colorless liquid.

Column material: 100-200 mesh silica

Eluent: petroleum ether/ethyl acetate (85:15)

Yield: 68%

^{1}H NMR (500 MHz, CDCl$_3$) δ 7.61 (d, *J* = 7.7 Hz, 1H), 7.53 (d, *J* = 8.2 Hz, 1H), 7.30 (t, *J* = 7.7 Hz, 1H), 7.23 (t, *J* = 7.4 Hz, 1H), 7.12 (s, 1H), 2.19 (s, 1H), 1.28 (dt, *J* = 14.7, 7.4 Hz, 2H), 1.12 (dd, *J* = 18.5, 7.4 Hz, 12H). **^{13}C NMR (126 MHz, CDCl$_3$)** δ 159.30, 158.16, 127.83, 124.80, 122.64, 121.40, 118.23, 111.65, 17.12, 16.99, 12.83. **HRMS (ESI):** Calculated mass for $C_{14}H_{20}NaO_2Si$ [M+Na]$^+$: 271.1130; observed mass: 271.1121. **IR**: 3213 (OH).

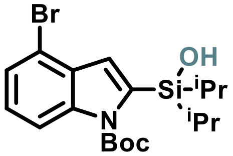

Tert-butyl 4-bromo-2-(hydroxydiisopropylsilyl)-1H-indole-1-carboxylate (34):

Physical appearance: Brown oil.

Column material: 100-200 mesh silica

Eluent: petroleum ether/ethyl acetate (85:15)

Yield: 58%

^{1}H NMR (500 MHz, CDCl$_3$) δ 7.83 (d, *J* = 8.9 Hz, 1H), 7.67 (d, *J* = 1.9 Hz, 1H), 7.37 (dd, *J* = 8.9, 2.0 Hz, 1H), 6.85 (s, 1H), 1.72 (s, 1H), 1.71 (s, 9H), 1.41 (dtd, *J* = 14.8, 7.4, 4.0 Hz, 2H), 1.13 (d, *J* = 7.4 Hz, 6H), 0.92 (d, *J* = 7.4 Hz, 6H). **^{13}C NMR (126 MHz, CDCl$_3$)** δ 151.41, 139.56, 136.40, 133.21, 127.24, 123.51, 121.24, 116.90, 115.97, 84.83, 28.39, 19.83, 19.67, 11.87. **HRMS (ESI):** $C_{19}H_{28}NaBrNO_3Si$ $[M+Na]^+$: 448.0920; observed mass: 448.0913. **IR**: 3346 (OH).

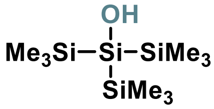

1,1,1,3,3,3-hexamethyl-2-(trimethylsilyl)trisilan-2-ol (35):

Physical appearance: Colorless oil.

Column material: 100-200 mesh silica

Eluent: petroleum ether/ethyl acetate (95:5)

Yield: 93%

^{1}H NMR (500 MHz, CD$_3$CN) δ 1.86 (s, 1H), 0.16 (s, 27H). **^{13}C NMR (126 MHz, CD$_3$CN)** δ -0.11. **HRMS (ESI):** $C_9H_{29}OSi_4$ $[M+H]^+$: 265.1295; observed mass: 265.1294. **IR**: 3390 (OH).

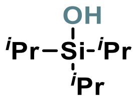

Triisopropylsilanol (36):

Physical appearance: Colorless oil.

Column material: 100-200 mesh silica

Eluent: petroleum ether/ethyl acetate (92:8)

Yield: 52%

^{1}H NMR (500 MHz, CDCl$_3$) δ 1.64 (s, 1H), 1.03 (s, 21H). **^{13}C NMR (126 MHz, CDCl$_3$)** 17.88, 12.51. **LRMS:** $C_9H_{23}OSi$ $[M+H]^+$: 175.1518; observed mass: 175.1509. **IR**: 3426 (OH).

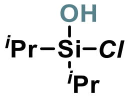

Chlorodiisopropylsilanol (37):

Physical appearance: Colorless liquid.

Column material: 100-200 mesh silica

Eluent: petroleum ether/ethyl acetate (95:5)

Yield: 40%

^{1}H NMR (500 MHz, CDCl$_3$) δ 3.77 (s, 1H), 1.02 (d, *J* = 7.7 Hz, 12H), 0.97 – 0.87 (m, 2H). **^{13}C NMR (126 MHz, CDCl$_3$)** δ 17.30, 17.27, 13.32. **LRMS**: Calculated mass for $C_6H_{16}ClOSi$ $[M+H]^+$: 167.0659; observed mass: 167.0641. **IR**: 3496 (OH).

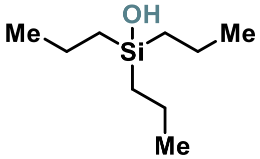

Tripropylsilanol (38):

Physical appearance: Colorless liquid.

Column material: 100-200 mesh silica

Eluent: petroleum ether/ethyl acetate (93:7)

Yield: 51%

^{1}H NMR (400 MHz, CDCl$_3$) δ 2.30 (s, 1H), 1.44 – 1.31 (m, 6H), 0.95 (t, *J* = 7.3 Hz, 9H), 0.62 – 0.48 (m, 6H). **^{13}C NMR (101 MHz, CDCl$_3$)** δ 18.49, 18.01, 16.83. **LRMS**: Calculated mass for $C_9H_{23}OSi$ $[M+H]^+$: 175.1518; observed mass: 175.1503. **IR**: 3433 (OH).

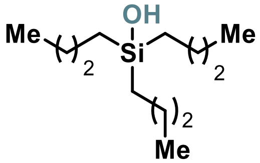

Tributylsilanol (39):

Physical appearance: Colorless liquid.

Column material: 100-200 mesh silica

Eluent: petroleum ether/ethyl acetate (92:8)

Yield: 48%

^{1}H NMR (500 MHz, CDCl$_3$) δ 2.03 (s, 1H), 1.33 (dd, *J* = 7.7, 3.7 Hz, 12H), 0.89 (t, *J* = 6.9 Hz, 9H), 0.75 – 0.45 (m, 6H). **^{13}C NMR (126 MHz, CDCl$_3$)** δ 26.77, 25.51, 14.97, 13.96. **LRMS:** Calculated mass for $C_{12}H_{29}OSi$ $[M+H]^+$: 217.1988; observed mass: 217.1980. **IR**: 3476 (OH).

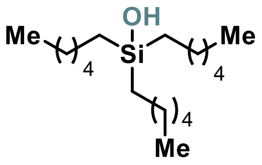

Trihexylsilanol (40):

Physical appearance: Colorless liquid.

Column material: 100-200 mesh silica

Eluent: petroleum ether/ethyl acetate (93:7)

Yield: 45%

^{1}H NMR (500 MHz, $CDCl_3$) δ 2.33 (s, 1H), 1.42 – 1.19 (m, 24H), 0.89 (t, J = 6.9 Hz, 9H), 0.69 – 0.41 (m, 6H). **^{13}C NMR (101 MHz, $CDCl_3$)** δ 33.56, 31.81, 23.28, 22.84, 15.31, 14.30. **HRMS (ESI):** Calculated mass for $C_{18}H_{41}OSi$ $[M+H]^+$: 301.2927; observed mass: 301.2925. **IR**: 3482 (OH).

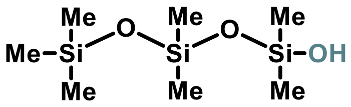

1,1,3,3,5,5,5-Heptamethyltrisiloxan-1-ol (41):

Physical appearance: Sticky oil.

Column material: 100-200 mesh silica

Eluent: petroleum ether/ethyl acetate (85:15)

Yield: 40%

^{1}H NMR (400 MHz, $CDCl_3$) δ 0.14 (s, 6H), 0.10 (s, 9H), 0.07 (s, 6H), 0.04 (s, 1H). **^{13}C NMR (101 MHz, $CDCl_3$)** 2.00, 1.35, 0.55. **HRMS (ESI)**: Calculated mass for $C_7H_{23}O_3Si_3$ $[M+H]^+$: 239.0955; observed mass: 239.0949. **IR**: 3520 (OH).

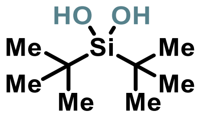

Di-tert-butylsilanediol (42):

Physical appearance: Colourless oil.

Column material: 100-200 mesh silica

Eluent: petroleum ether/ethyl acetate (85:15)

Yield: 42%

^{1}H NMR (500 MHz, $CDCl_3$) δ 2.19 (s, 2H), 1.04 (s, 18H). **^{13}C NMR (126 MHz, $CDCl_3$)** δ 27.44, 19.99. **LRMS**: Calculated mass for $C_8H_{21}O_2Si$ $[M+H]^+$: 177.1311; observed mass: 177.1303. **IR**: 3254 (OH).

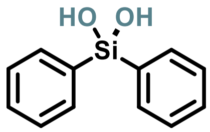

Diphenylsilanediol (43):

Physical appearance: Colourless liquid.

Column material: 100-200 mesh silica

Eluent: petroleum ether/ethyl acetate (88:12)

Yield: 69%

^{1}H NMR (400 MHz, DMSO) δ 7.64 – 7.51 (m, 4H), 7.40 – 7.25 (m, 6H), 6.95 (s, 2H). **^{13}C NMR (101 MHz, DMSO)** δ 143.01, 139.28, 134.54, 132.68. **LRMS**: Calculated mass for $C_{12}H_{13}O_2Si$ $[M+H]^+$: 217.0685; observed mass: 217.0680.

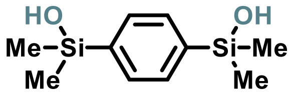

1,4-Phenylenebis(dimethylsilanol) (44):

Physical appearance: Colorless Liquid.

Column material: 100-200 mesh silica

Eluent: petroleum ether/ethyl acetate (80:20)

Yield: 60%

^{1}H NMR (400 MHz, $CDCl_3$) δ 7.60 (s, 4H), 2.13 (s, 1H), 1.67 (s, 1H), 0.40 (s, 12H). **^{13}C NMR (101 MHz, $CDCl_3$)** δ 140.85, 132.65, 0.16. **HRMS (ESI)**: Calculated mass for $C_{10}H_{19}O_2Si_2$ $[M+H]^+$: 227.0924., observed mass: 227.0920. **IR**: 3429 (OH).

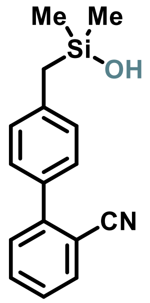

4'-((Hydroxydimethylsilyl)methyl)-[1,1'-biphenyl]-2-carbonitrile (45):

Physical appearance: Yellow sticky liquid.

Column material: 100-200 mesh silica

Eluent: petroleum ether/ethyl acetate (85:15)

Yield: 61%

^{1}H NMR (400 MHz, $CDCl_3$) δ 7.72 (dd, *J* = 7.7, 0.8 Hz, 1H), 7.60 (td, *J* = 7.8, 1.3 Hz, 1H), 7.48 (d, *J* = 7.8 Hz, 1H), 7.43 (d, *J* = 8.1 Hz, 2H), 7.38 (td, *J* = 7.7, 1.1 Hz, 1H), 7.18 (d, *J* = 8.1 Hz, 2H), 2.65 (s, 1H), 2.24 (s, 2H), 0.16 (s, 6H). **^{13}C NMR (101 MHz, $CDCl_3$)** δ 145.66, 140.37, 134.13, 133.84, 132.95, 130.04, 128.81, 128.65, 127.27, 119.07, 110.99, 28.23, -0.54. **HRMS (ESI)**: Calculated mass for $C_{16}H_{18}NOSi$ $[M+H]^+$: 268.1158; observed mass: 268.1158. **IR**: 3387 (OH).

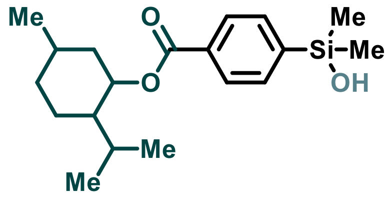

2-Isopropyl-5-methylcyclohexyl 4-(hydroxydimethylsilyl)benzoate (46):[12]

Physical appearance: Colorless liquid.

Column material: 100-200 mesh silica

Eluent: petroleum ether/ethyl acetate (83:17)

Yield: 56%

^{1}H NMR (500 MHz, $CDCl_3$) δ 8.04 (d, *J* = 8.1 Hz, 2H), 7.67 (d, *J* = 8.0 Hz, 2H), 4.95 (td, *J* = 10.9, 4.4 Hz, 1H), 2.14 (m, 1H), 1.96 – 1.92 (m, 1H), 1.77 – 1.71 (m, 2H), 1.65 – 1.53 (m, 3H), 1.12 (m, 2H), 0.94 – 0.91 (m, 6H), 0.79 (d, *J* = 7.0 Hz, 3H), 0.44 (s, 6H). **^{13}C NMR (126 MHz, $CDCl_3$)** δ 166.6, 145.0, 144.7, 133.2, 128.9, 75.3, 47.5, 41.4, 34.4, 31.3, 26.3, 23.9, 23.4, 21.0,

20.8, 16.2, 0.2. **HRMS (ESI)**: Calculated mass for $C_{19}H_{31}O_3Si$ $[M+H]^+$: 335.2042; observed mass: 335.2040. **IR**: 3510 (OH).

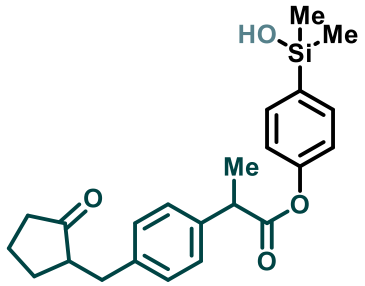

4-(Hydroxydimethylsilyl)phenyl 2-(4-((2-oxocyclopentyl)methyl)phenyl)propanoate (47):

Physical appearance: Brownish oil.

Column material: 100-200 mesh silica

Eluent: petroleum ether/ethyl acetate (75:25)

Yield: 50%

^{1}H NMR (500 MHz, $CDCl_3$) δ 7.15 (d, *J* = 7.9 Hz, 4H), 7.08 (d, *J* = 8.0 Hz, 4H), 3.12 (dd, *J* = 13.9, 3.9 Hz, 2H), 2.86 – 2.78 (m, 2H), 1.70 (s, 1H), 1.61 – 1.45 (m, 6H), 1.27 (s, 3H), 1.25 (s, 6H). **^{13}C NMR (126 MHz, $CDCl_3$)** δ 200.49, 169.94, 146.81, 135.71, 128.56, 128.20, 127.35, 126.37, 126.16, 78.41, 48.72, 41.74, 40.97, 38.53, 26.71, 26.02, 23.26, 22.70, 21.06, 14.23. **HRMS (ESI)**: Calculated mass for $C_{23}H_{29}O_4Si$ $[M+H]^+$: 397.1835; observed mass: 397.1832. **IR**: 3555 (OH).

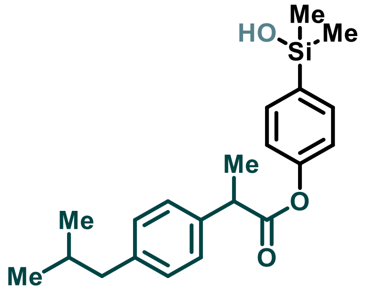

4-(hydroxydimethylsilyl)phenyl 2-(4-isobutylphenyl)propanoate (48):[12]

Physical appearance: Yellow sticky liquid.

Column material: 100-200 mesh silica

Eluent: petroleum ether/ethyl acetate (86:14)

Yield: 46%

^{1}H NMR (500 MHz, CDCl$_3$) δ 7.54 (d, *J* = 8.3 Hz, 2H), 7.28 (d, *J* = 7.9 Hz, 2H), 7.16 (d, *J* = 7.9 Hz, 2H), 7.00 (d, *J* = 8.4 Hz, 2H), 3.96 (q, *J* = 7.1 Hz, 1H), 2.50 (d, *J* = 7.2 Hz, 2H), 1.91 (s, 1H), 1.89 – 1.78 (m, 1H), 1.61 (d, *J* = 7.1 Hz, 3H), 0.90 (d, *J* = 7.1 Hz, 6H), 0.41 (s, 6H). **^{13}C NMR (126 MHz, CDCl$_3$)** δ 173.2, 152.1, 140.8, 137.1, 136.5, 134.3, 129.5, 127.2, 120.9, 45.2, 45.0, 30.2, 22.4, 18.5, 0.00. **HRMS (ESI)**: Calculated mass for $C_{21}H_{29}O_3Si$ $[M+H]^+$: 357.1886; observed mass: 357.1882. **IR**: 3512 (OH).

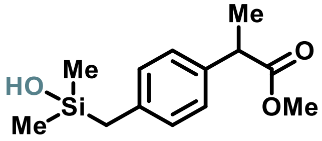

Methyl 2-(4-((hydroxydimethylsilyl)methyl)phenyl)propanoate (49):

Physical appearance: Colorless liquid.

Column material: 100-200 mesh silica

Eluent: petroleum ether/ethyl acetate (70:20)

Yield: 66%

^{1}H NMR (500 MHz, CDCl$_3$) δ 7.24 (d, *J* = 8.0 Hz, 2H), 7.07 (d, *J* = 8.0 Hz, 2H), 3.75 (dd, *J* = 15.1, 7.8 Hz, 1H), 3.71 (s, 3H), 2.40 (s, 1H), 2.21 (s, 2H), 1.55 (d, *J* = 7.2 Hz, 3H), 0.15 (s, 6H), 0.14 (s, 6H). **^{13}C NMR (126 MHz, CDCl$_3$)** δ 175.29, 138.89, 136.42, 128.43, 127.46, 51.95, 45.00, 23.89, 18.68, -4.58. **HRMS (ESI)**: Calculated mass for $C_{13}H_{20}NaO_3Si$ $[M+Na]^+$: 275.1079; observed mass: 275.1077. **IR**: 3342 (OH).

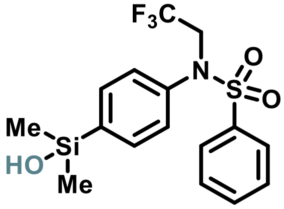

***N*-(4-(Hydroxydimethylsilyl)phenyl)-N-(2,2,2-trifluoroethyl)benzenesulfonamide (50):**[4]

Physical appearance: White semi-solid.

Column material: 100-200 mesh silica

Eluent: petroleum ether/ethyl acetate (88:12)

Yield: 60%

^{1}H NMR (400 MHz, CDCl$_3$) δ 7.60 (d, *J* = 7.3 Hz, 3H), 7.52 (d, *J* = 7.7 Hz, 2H), 7.49 (t, *J* = 7.4

Hz, 2H), 7.04 (d, J = 7.7 Hz, 2H), 4.22 (q, J = 8.2 Hz, 2H), 2.60 (s, 1H), 0.37 (s, 6H). **^{13}C NMR (101 MHz, $CDCl_3$)** δ 140.44, 140.00, 138.18, 134.15, 133.28, 128.99, 128.36, 127.66, 125.05, 122.26, 52.56, 52.22, 51.87, 51.52, -0.07. **HRMS (ESI)**: Calculated mass for $C_{16}H_{19}F_3NO_3SSi$ $[M+H]^+$: 390.0807; observed mass: 390.0805. **IR**: 3510 (OH).

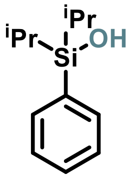

Diisopropyl(phenyl)silanol (51):

Physical appearance: Colorless liquid.

Column material: 100-200 mesh silica

Eluent: petroleum ether/ethyl acetate (95:5)

Yield: 70%

^{1}H NMR (400 MHz, $CDCl_3$) δ 7.58 – 7.54 (m, 2H), 7.41 – 7.36 (m, 3H), 1.85 (s, 1H), 1.28 – 1.16 (m, 2H), 1.06 (d, J = 7.3 Hz, 6H), 0.98 (d, J = 7.4 Hz, 6H). **^{13}C NMR (101 MHz, $CDCl_3$)** δ 135.57, 134.28, 129.54, 127.85, 17.35, 17.11, 12.59. **LRMS**: Calculated mass for $C_{12}H_{21}OSi$ $[M+H]^+$: 209.1362; observed mass: 209.1354. **IR**: 3231 (OH).

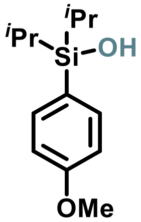

Diisopropyl(4-methoxyphenyl)silanol (52):

Physical appearance: Colorless liquid.

Column material: 100-200 mesh silica

Eluent: petroleum ether/ethyl acetate (92:8)

Yield: 68%

^{1}H NMR (400 MHz, $CDCl_3$) δ 7.50 (d, J = 8.6 Hz, 2H), 6.93 (d, J = 8.5 Hz, 2H), 3.82 (s, 3H), 2.18 (s, 1H), 1.29 – 1.09 (m, 2H), 1.06 (d, J = 7.3 Hz, 6H), 0.98 (d, J = 7.4 Hz, 6H). **^{13}C NMR (101 MHz, $CDCl_3$)** δ 160.76, 135.77, 126.40, 113.61, 55.11, 17.34, 17.11, 12.65. **HRMS (ESI)**: Calculated mass for $C_{13}H_{23}O_2Si$ $[M+H]^+$: 239.1467; observed mass: 239.1465. **IR**: 3231 (OH).

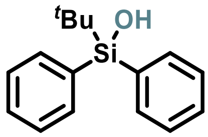

Tert-butyldiphenylsilanol (53):

Physical appearance: Yellow oil.

Column material: 100-200 mesh silica

Eluent: petroleum ether/ethyl acetate (90/10).

Yield: 73%

^{1}H NMR (500 MHz, CDCl$_3$) δ 7.77 (dd, *J* = 7.9, 1.5 Hz, 4H), 7.56 – 7.37 (m, 6H), 2.55 (s, 1H), 1.13 (s, 9H). **^{13}C NMR (126 MHz, CDCl$_3$)** δ 135.37, 135.02, 129.82, 127.90, 26.76, 19.19. **HRMS (ESI)**: Calculated mass for $C_{16}H_{20}NaOSi$ [M+Na]$^+$: 279.1181.; observed mass: 279.1181. **IR**: 3312 (OH).

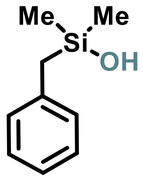

Benzyldimethylsilanol (54):

Physical appearance: Colourless liquid.

Column material: 100-200 mesh silica

Eluent: petroleum ether/ethyl acetate (80/20, v/v).

Yield: 65%

^{1}H NMR (400 MHz, CDCl$_3$) δ 7.31 (t, *J* = 7.6 Hz, 2H), 7.16 (dd, *J* = 14.2, 7.2 Hz, 3H), 3.23 (s, 1H), 2.24 (s, 2H), 0.19 (s, 6H). **^{13}C NMR (126 MHz, CDCl$_3$)** δ 139.21, 128.46, 128.36, 124.34, 28.18, -0.66. **HRMS (ESI)**: Calculated mass for $C_9H_{15}OSi$ [M+H]$^+$: 167.0892.; observed mass: 167.0890. **IR**: 3312 (OH).

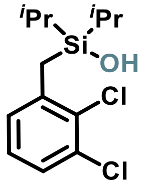

(2,3-dichlorobenzyl)diisopropylsilanol (55):

Physical appearance: Sticky oil.

Column material: 100-200 mesh silica

Eluent: petroleum ether/ethyl acetate (90/10).

Yield: 53%

^{1}H NMR (400 MHz, CDCl$_3$) δ 7.21 (dd, *J* = 7.8, 2.0 Hz, 1H), 7.10 (dd, *J* = 7.9, 1.9 Hz, 1H), 7.05 (t, *J* = 7.8 Hz, 1H), 2.43 (s, 2H), 1.77 (s, 1H), 1.13 – 0.87 (m, 14H). **^{13}C NMR (101 MHz, CDCl$_3$)** δ 140.70, 133.33, 130.98, 128.53, 127.06, 126.77, 20.92, 17.42, 17.31, 13.23. **HRMS (ESI)**: Calculated mass for $C_{13}H_{21}Cl_2OSi$ [M+H]$^+$: 291.0739.; observed mass: 291.0738. **IR**: 3434 (OH).

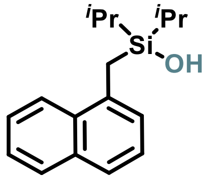

Diisopropyl(naphthalen-1-ylmethyl)silanol (56):

Physical appearance: Yellow oily liquid.

Column material: 100-200 mesh silica

Eluent: petroleum ether/ethyl acetate (90/10).

Yield: 76%

^{1}H NMR (400 MHz, CDCl$_3$) δ 8.07 (d, *J* = 8.3 Hz, 1H), 7.87 – 7.81 (m, 1H), 7.64 (d, *J* = 8.1 Hz, 1H), 7.54 – 7.44 (m, 2H), 7.40 – 7.34 (m, 1H), 7.29 (d, *J* = 6.8 Hz, 1H), 2.68 (s, 2H), 1.42 (s, 1H), 1.22 – 0.87 (m, 14H). **^{13}C NMR (101 MHz, CDCl$_3$)** δ 136.27, 134.23, 132.15, 128.99, 126.12, 125.84, 125.70, 125.30, 124.57, 18.65, 17.60, 17.49, 13.18. **HRMS (ESI)**: Calculated mass for $C_{17}H_{25}OSi$ [M+H]$^+$: 273.1675.; observed mass: 273.1670. **IR**: 3278 (OH).

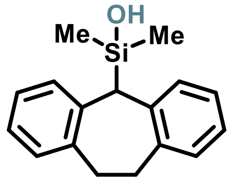

(10,11-Dihydro-5H-dibenzo[a,d][7]annulen-5-yl)dimethylsilanol (57):

Physical appearance: Colourless liquid.

Column material: 100-200 mesh silica

Eluent: petroleum ether/ethyl acetate (85/15).

Yield: 60%

^{1}H NMR (500 MHz, $CDCl_3$) δ 7.18 – 7.05 (m, 8H), 3.76 (s, 1H), 3.51 – 3.35 (m, 2H), 2.98 – 2.87 (m, 2H), 2.00 (s, 1H), 0.11 (s, 6H). **^{13}C NMR (126 MHz, $CDCl_3$)** δ 139.76, 139.45, 130.40, 129.96, 126.17, 125.47, 50.41, 33.81, 0.14. **HRMS (ESI)**: Calculated mass for $C_{17}H_{21}OSi$ $[M+H]^+$: 269.1238; observed mass: 269.1246. **IR**: 3376 (OH).

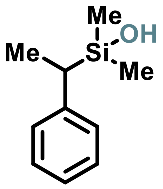

Dimethyl(1-phenylethyl)silanol (58):

Physical appearance: Colourless liquid.

Column material: 100-200 mesh silica

Eluent: petroleum ether/ethyl acetate (88/12).

Yield: 56%

^{1}H NMR (500 MHz, $CDCl_3$) δ 7.28 (d, J = 8.2 Hz, 2H), 7.12 (t, J = 7.1 Hz, 3H), 2.26 (q, J = 7.5 Hz, 1H), 1.86 (s, 1H), 1.41 (d, J = 7.6 Hz, 3H), 0.10 (d, J = 9.4 Hz, 6H). **^{13}C NMR (126 MHz, $CDCl_3$)** δ 144.85, 128.48, 127.30, 124.82, 31.13, 14.35, -2.07, -2.35. **HRMS (ESI)**: Calculated mass for $C_{10}H_{17}OSi$ $[M+H]^+$: 181.1049.; observed mass: 181.1041. **IR**: 3234 (OH).

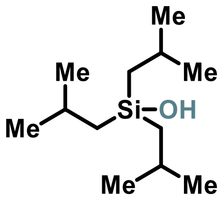

Triisobutylsilanol (59):

Physical appearance: Colorless oil

Column material: 100-200 mesh silica

Eluent: petroleum ether/ethyl acetate (90/10).

Yield: 62%

^{1}H NMR (400 MHz, $CDCl_3$) δ 1.84 (dp, J = 13.3, 6.7 Hz, 3H), 1.50 (s, 1H), 0.96 (d, J = 6.7 Hz, 18H), 0.64 – 0.56 (m, 6H). **^{13}C NMR (101 MHz, $CDCl_3$)** 27.22, 26.63, 24.53. **HRMS (ESI)**: Calculated mass for $C_{12}H_{28}NaOSi$ $[M+Na]^+$: 239.1807.; observed mass: 239.1800. **IR**: 3398 (OH).

For entry 2, 3, 9, 11, 30, 31, 46, 48, 50 the characterization data are consistent with that reported in the literature.

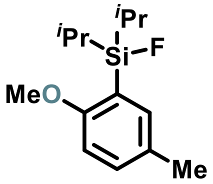

Fluorodiisopropyl(2-methoxy-5-methylphenyl)silane (60):[5]

Physical appearance: Colorless oil.

Column material: 100-200 mesh silica

Eluent: petroleum ether/ethyl acetate (90/10).

Yield: 42%

^{1}H NMR (500 MHz, CDCl$_3$) δ 6.91 (s, 1H), 6.88 (dd, J = 8.4, 1.8 Hz, 1H), 6.69 (d, J = 8.1, 1H), 3.78 (s, 3H), 2.22-2.24 (m, 5 H), 1.01-1.07 (m, 14H). **^{13}C NMR (126 MHz, CDCl$_3$)** δ 155.0, 131.1, 129.7, 126.4, 126.2, 110.1, 55.2, 20.7, 16.9, 14.4, 14.3 (d, J = 12.9 Hz), 12.6 (d, J = 12.9 Hz), 12.7.

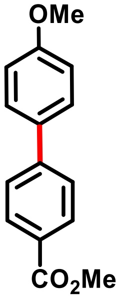

Methyl 4'-methoxy-[1,1'-biphenyl]-4-carboxylate (61):[6]

Physical appearance: Colorless oil.

Column material: 100-200 mesh silica

Eluent: petroleum ether/ethyl acetate (92/8).

Yield: 88%

^{1}H NMR (400 MHz, CDCl$_3$) δ 8.08 (d, J = 8.6 Hz, 2H), 7.61 (d, J = 8.5 Hz, 2H), 7.57 (d, J = 8.8 Hz, 2H), 6.99 (d, J = 8.8 Hz, 2H), 3.93 (s, 3H), 3.85 (s, 3H). **^{13}C NMR (101 MHz, CDCl$_3$)** δ 167.24, 160.03, 145.38, 132.56, 130.28, 128.52, 128.42, 126.62, 114.56, 55.53, 52.22.

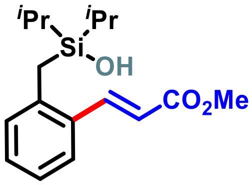

Methyl (*E*)-3-(2-((hydroxydiisopropylsilyl)methyl)phenyl)acrylate (62):[7]

Physical appearance: Colorless oil

Column material: 100-200 mesh silica

Eluent: petroleum ether/ethyl acetate (92/8).

Yield: 68%

^{1}H NMR (400 MHz, $CDCl_3$) δ 7.22 (t, *J* = 9.3 Hz, 2H), 7.13 (dd, *J* = 14.8, 7.3 Hz, 2H), 7.09 – 7.03 (m, 1H), 6.06 (d, *J* = 11.8 Hz, 1H), 3.67 (s, 3H), 2.19 (s, 2H), 1.09 – 0.94 (m, 9H), 0.89 – 0.80 (m, 5H).**^{13}C NMR (101 MHz, $CDCl_3$)** δ 168.09, 144.94, 138.07, 134.52, 129.45, 129.29, 128.95, 124.53, 121.20, 51.93, 19.70, 17.68, 17.50, 13.23.

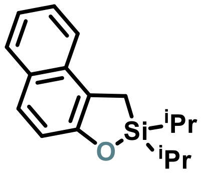

2,2-diisopropyl-1,2-dihydronaphtho[1,2-d][1,2]oxasilole (63):[5]

Physical appearance: Colorless oil.

Column material: 100-200 mesh silica

Eluent: petroleum ether/ethyl acetate (90/10).

Yield: 65%

^{1}H NMR (500 MHz, $CDCl_3$) δ 7.87 (d, *J* =8.44 Hz, 1 H), 7.83 (d, *J* =8.25 Hz, 1 H), 7.69 (d, *J* =8.80 Hz, 1 H), 7.54 (ddd, *J* =8.21, 6.92, 1.19 Hz, 1 H), 7.36 (ddd, *J* =8.07, 6.97, 1.10 Hz, 1 H), 7.25 (d, *J* =8.80 Hz, 1 H), 2.30 (s, 2 H), 1.27 - 1.38 (m, 2 H), 1.15 (dd, *J* =7.43, 1.74 Hz, 12 H). **^{13}C NMR (126 MHz, $CDCl_3$)** δ 158.12, 133.74, 129.05, 128.54, 128.27, 126.30, 123.58, 122.91, 119.90, 116.71, 16.83, 16.62, 12.44, 7.60.

For entry 60,63, the characterization data are consistent with that reported in the literature.

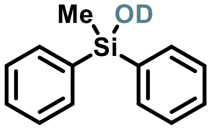

Methyldiphenylsilanol-O*D* (65):

Physical appearance: Colorless liquid.

Column material: 100-200 mesh silica

Eluent: petroleum ether/ethyl acetate (95/5).

Yield: 82%

^{1}H NMR (500 MHz, $CDCl_3$) δ 7.65 (dd, *J* = 6.7, 1.3 Hz, 4H), 7.52 – 7.46 (m, 2H), 7.41 (dd, *J* = 11.0, 4.3 Hz, 4H), 0.68 (s, 3H). **^{13}C NMR (101 MHz, $CDCl_3$)** δ 137.23, 134.16, 129.96, 128.03, -1.19.

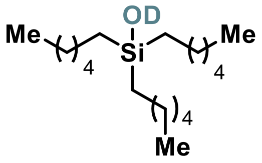

Trihexylsilanol-O*D* (66):

Physical appearance: Colorless liquid.

Column material: 100-200 mesh silica

Eluent: petroleum ether/ethyl acetate (94/6).

Yield: 40%

^{1}H NMR (500 MHz, $CDCl_3$) δ 1.39 – 1.18 (m, 24H), 0.88 (t, *J* = 7.0 Hz, 9H), 0.65 – 0.50 (m, 6H). **^{13}C NMR (101 MHz, $CDCl_3$)** δ 33.52, 31.79, 23.27, 22.82, 15.30, 14.32.

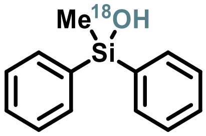

Methyldiphenylsilanol-^{18}O (67):

Physical appearance: Colorless liquid.

Column material: 100-200 mesh silica

Eluent: petroleum ether/ethyl acetate (95/5).

Yield: 81%

^{1}H NMR (400 MHz, $CDCl_3$) δ 7.66 – 7.61 (m, 4H), 7.49 – 7.44 (m, 2H), 7.43 – 7.37 (m, 4H), 3.31 (s, 1H), 0.67 (s, 3H). **^{13}C NMR (101 MHz, $CDCl_3$)** δ 137.19, 134.16, 129.99, 128.04, -1.17.

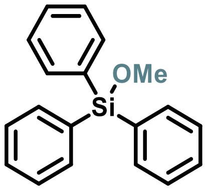

Methoxytriphenylsilane (68):

Physical appearance: Colorless liquid.

Column material: 100-200 mesh silica

Eluent: petroleum ether/ethyl acetate (94/6).

Yield: 27%

^{1}H NMR (500 MHz, $CDCl_3$) δ 7.65 – 7.63 (m, 6H), 7.47 – 7.44 (m, 3H), 7.40 (t, *J* = 7.1 Hz, 6H), 3.66 (s, 3H). **^{13}C NMR (126 MHz, $CDCl_3$)** δ 135.59, 134.08, 130.28, 128.11, 52.08.

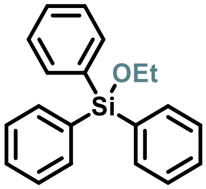

Ethoxytriphenylsilane (69):

Physical appearance: Colorless liquid.

Column material: 100-200 mesh silica

Eluent: petroleum ether/ethyl acetate (96/4).

Yield: 13%

^{1}H NMR (500 MHz, $CDCl_3$) δ 7.64 – 7.62 (m, 6H), 7.45 – 7.42 (m, 3H), 7.38 (t, *J* = 7.2 Hz, 6H), 3.87 (q, *J* = 7.0 Hz, 2H), 1.24 (t, *J* = 7.0 Hz, 3H). **^{13}C NMR (126 MHz, $CDCl_3$)** δ 135.59, 134.64, 130.17, 128.05, 59.96, 18.59.

7. NMR Spectra:

Dimethyl(phenyl)silanol (1):

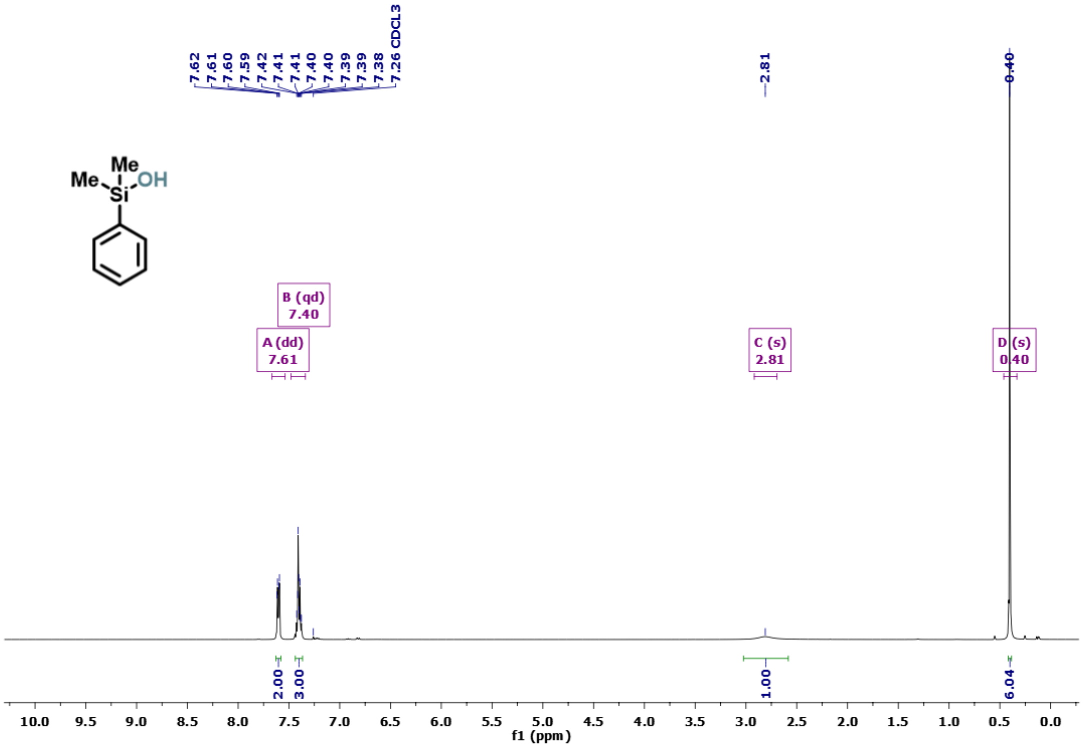

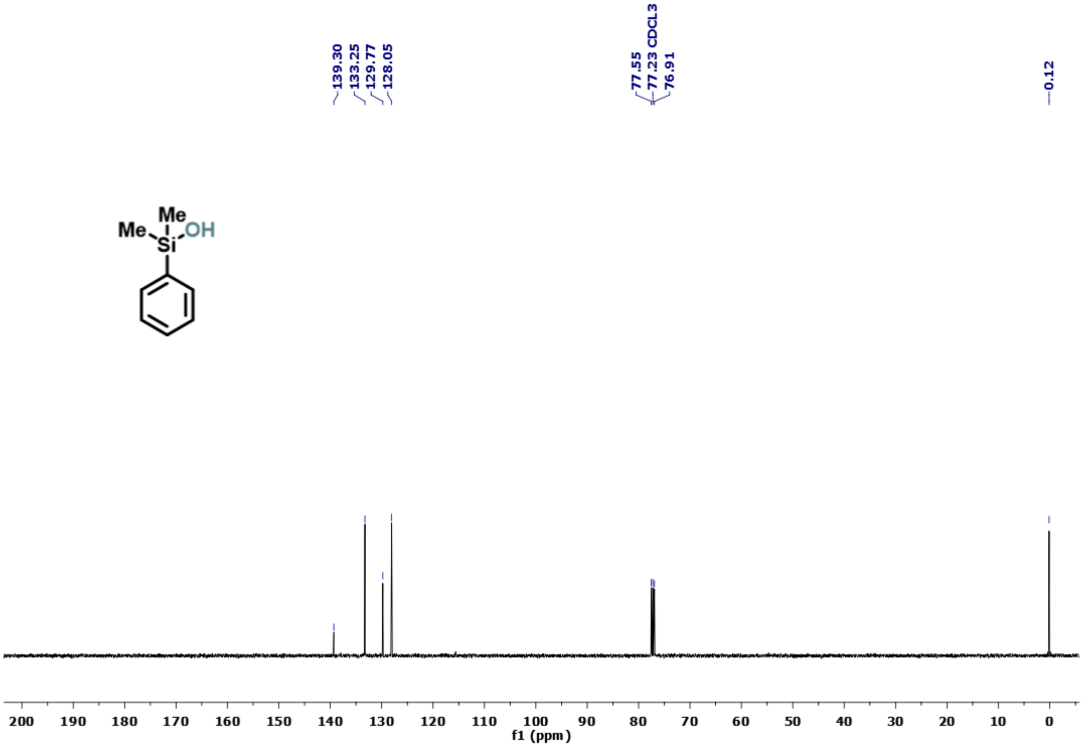

(3-fluorophenyl)diisopropylsilanol (4):

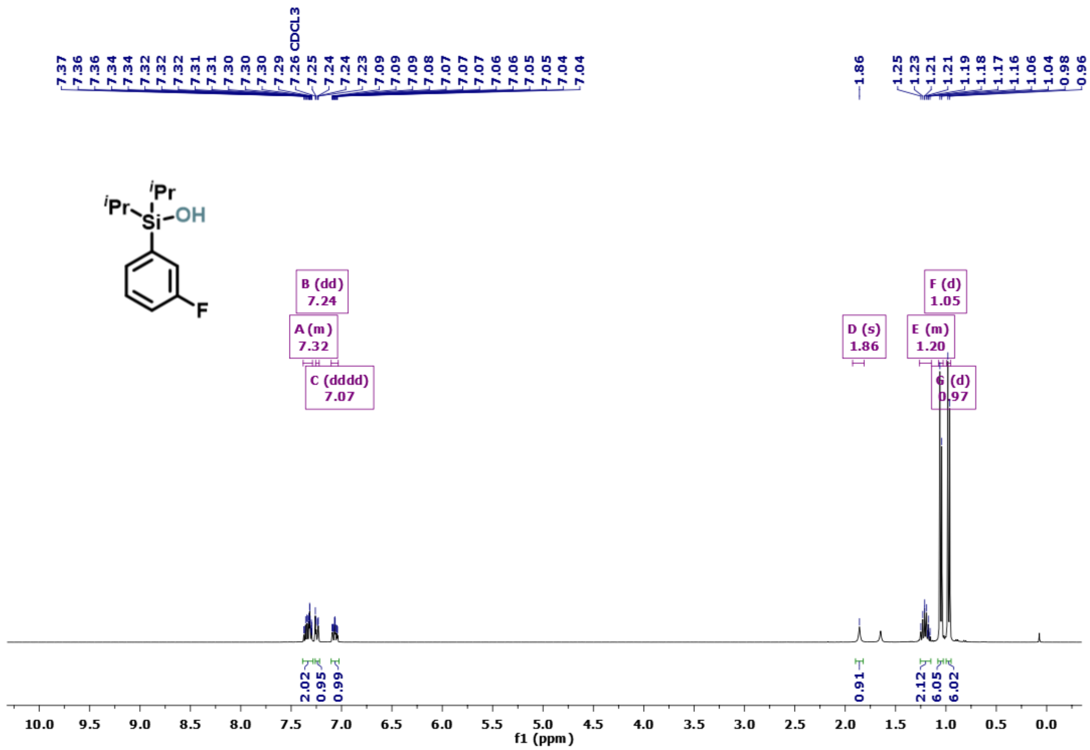

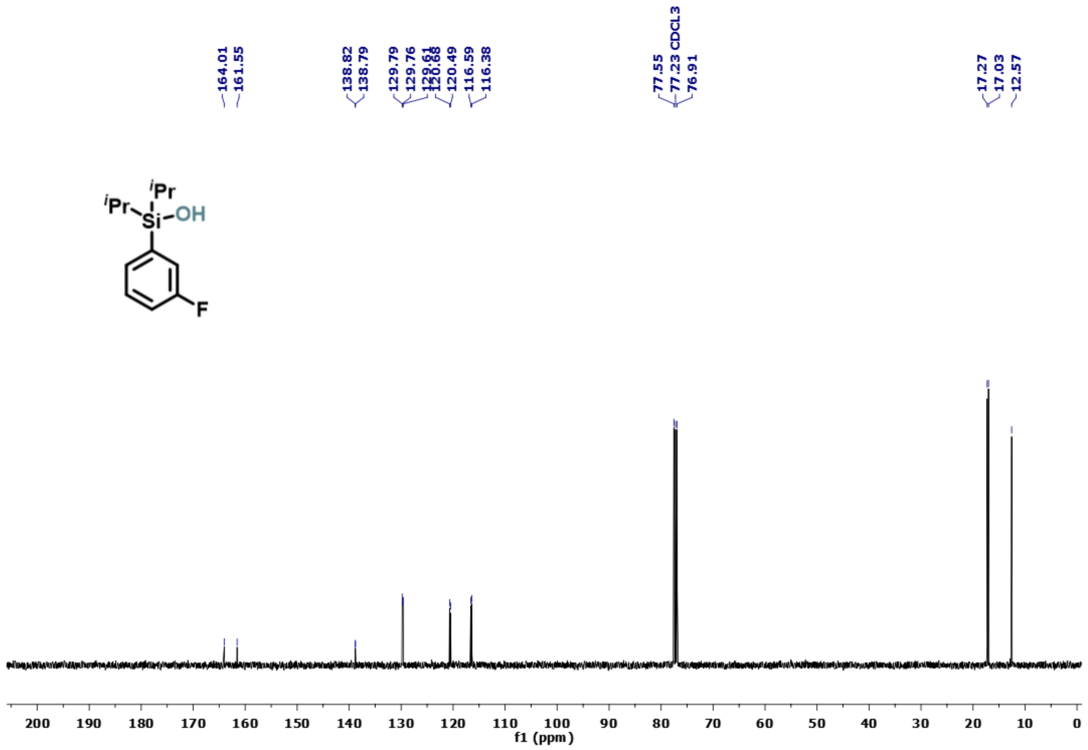

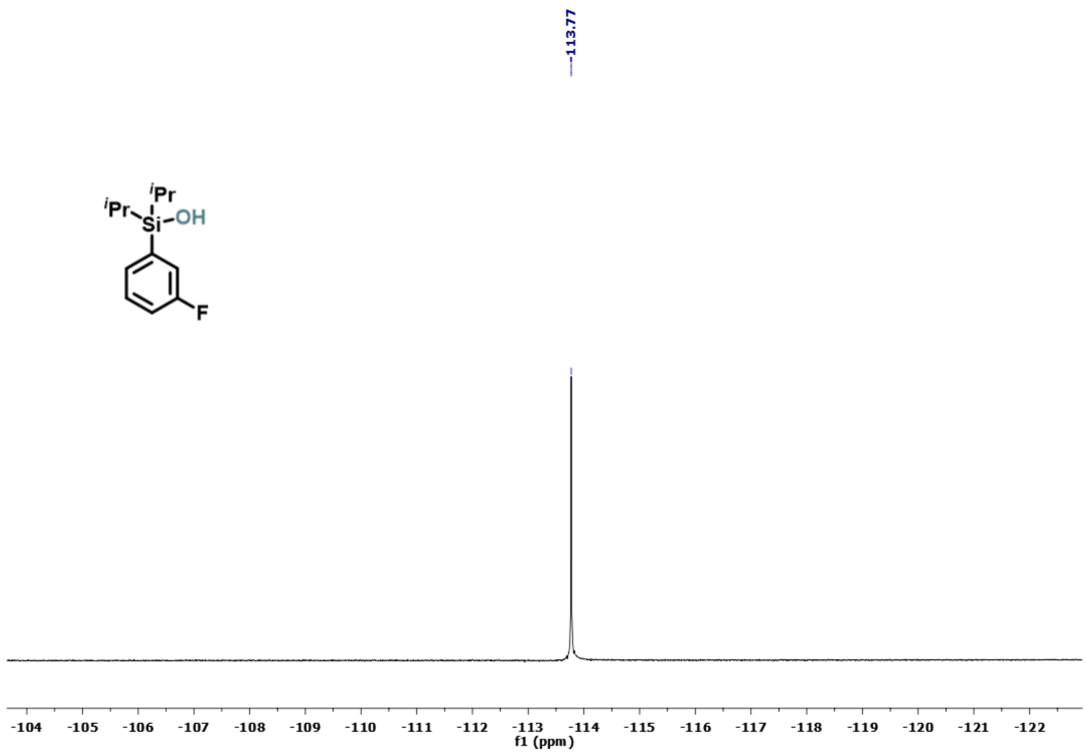

-113.77
^{i}Pr
^{i}Pr
Si
OH
F
-104
-105
-106
-107
-108
-109
-110
-111
-112
-113
-114
-115
-116
-117
-118
-119
-120
-121
-122
f1 (ppm)

Dimethyl(*p*-tolyl)silanol (5):

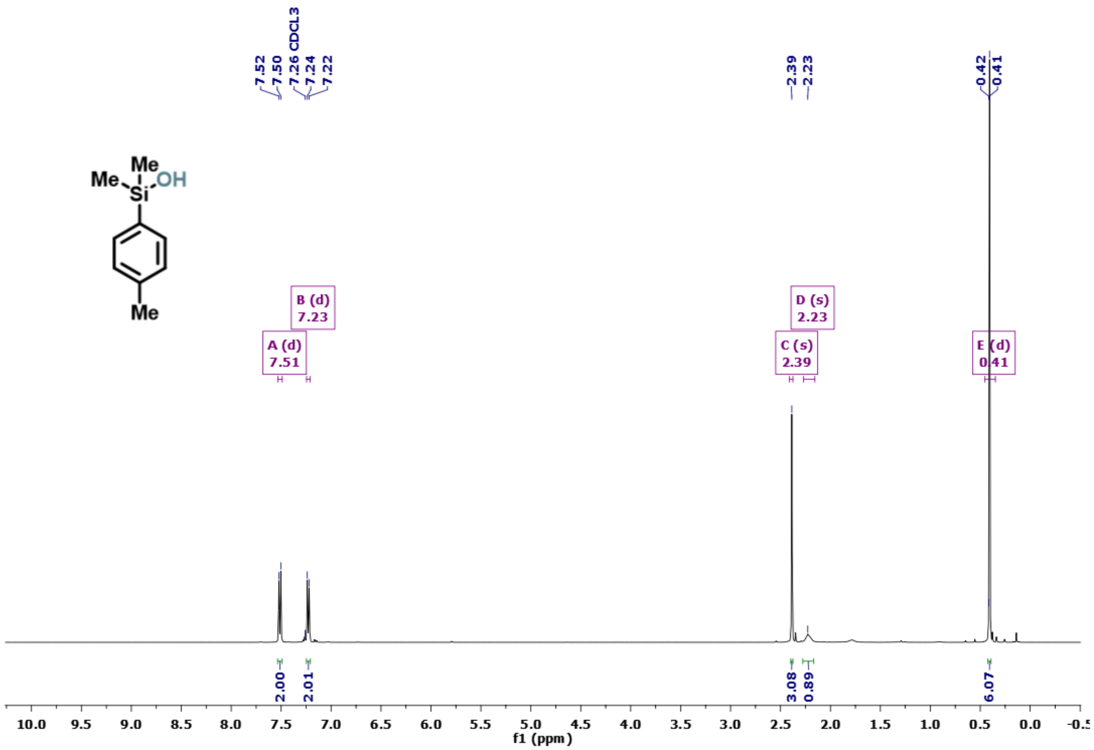

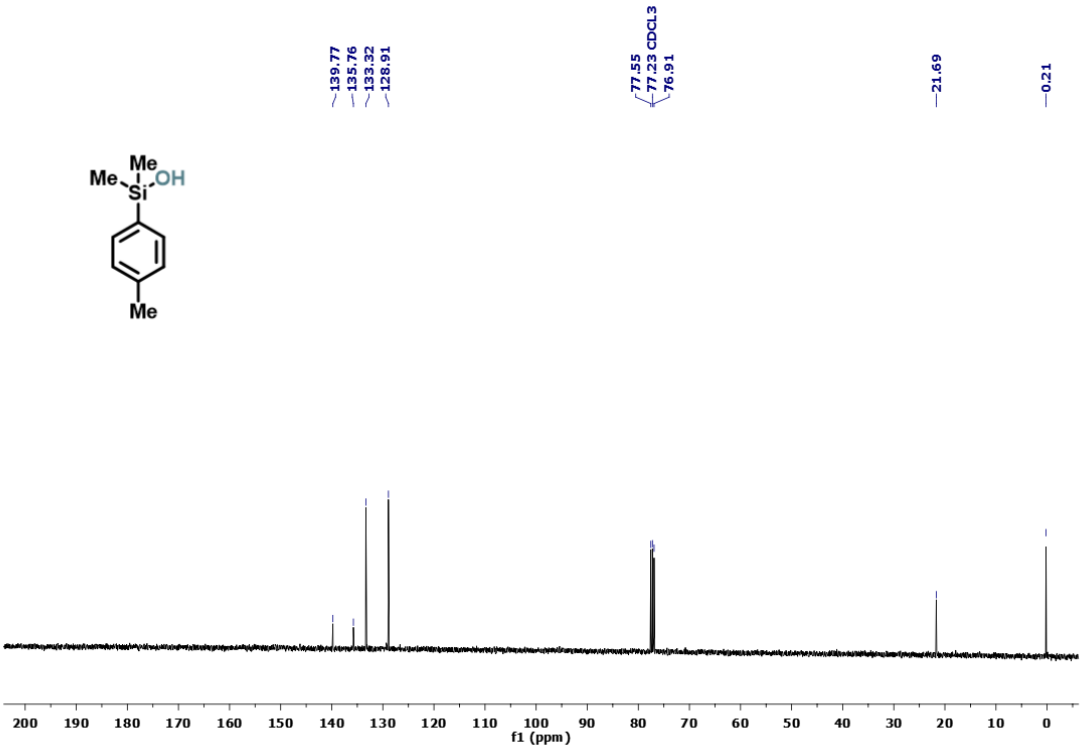

(4-Methoxyphenyl)dimethylsilanol (6):

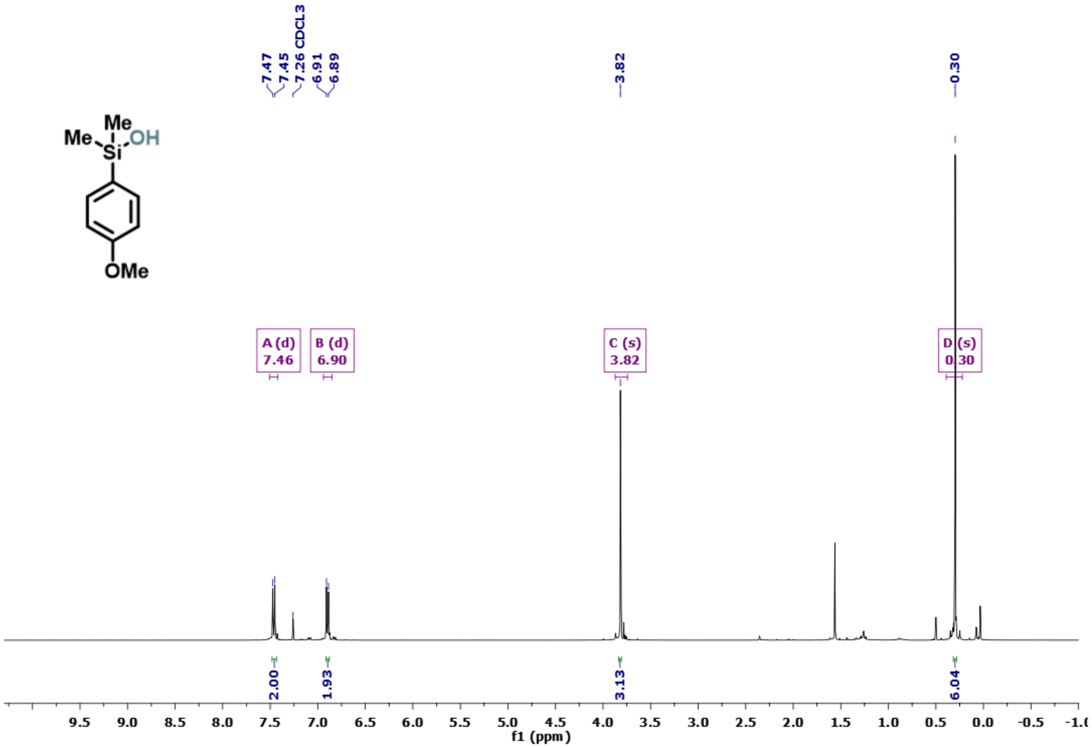

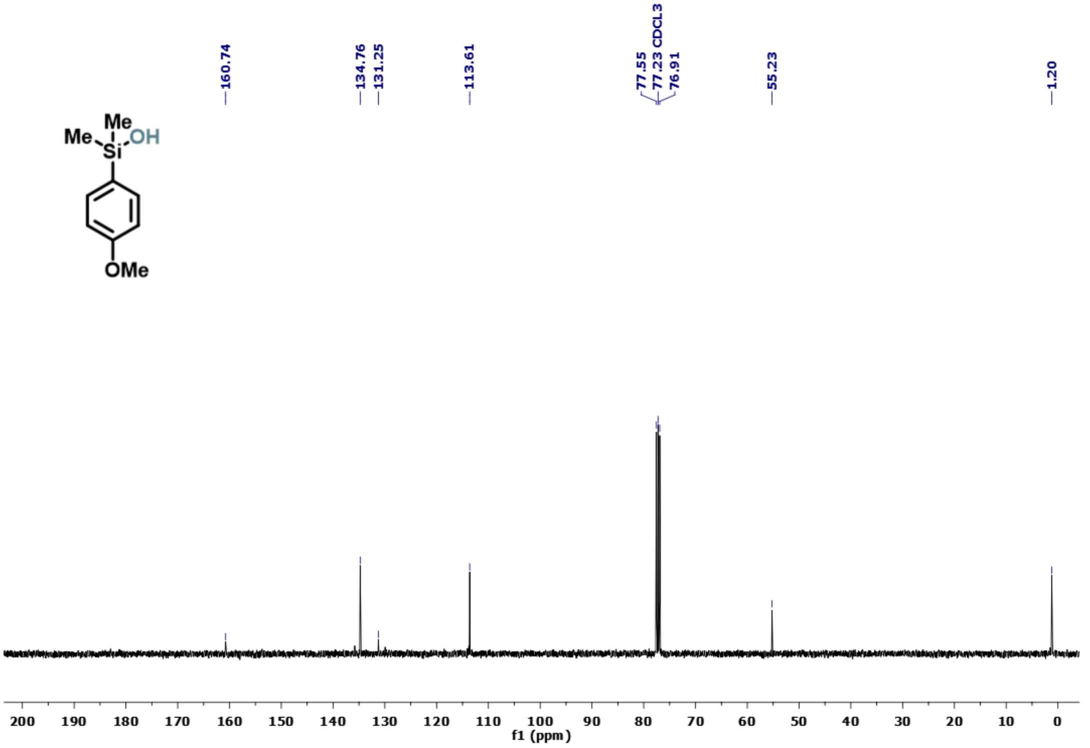

Dimethyl(4-(methylthio)phenyl)silanol (7):

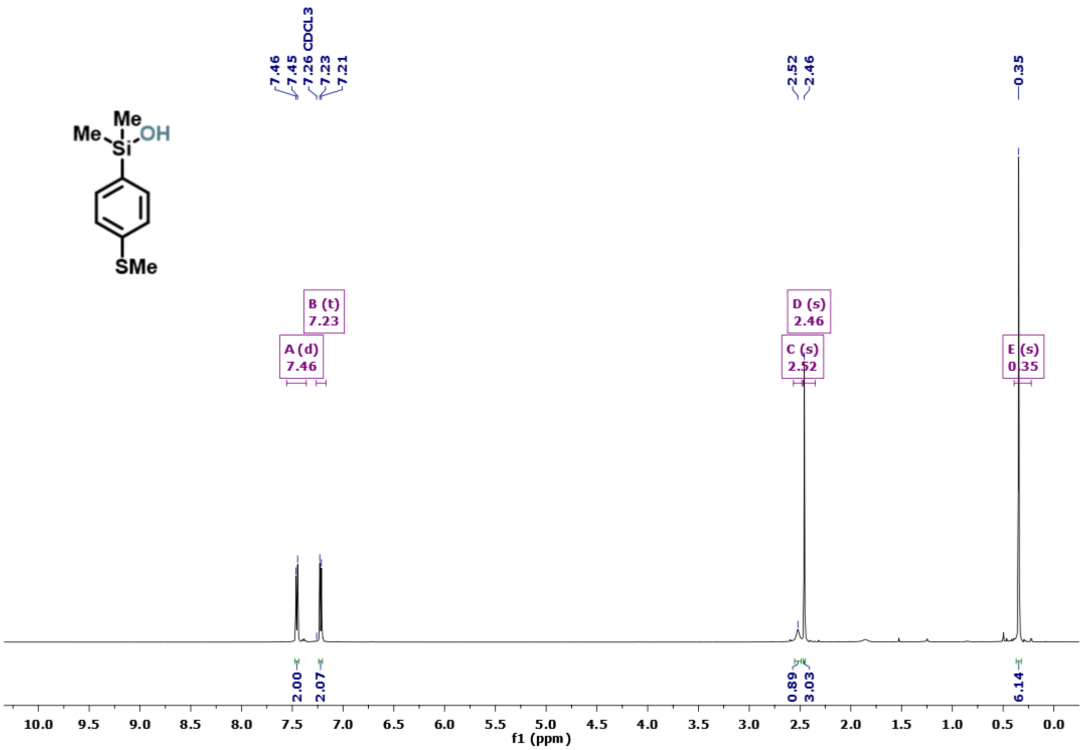

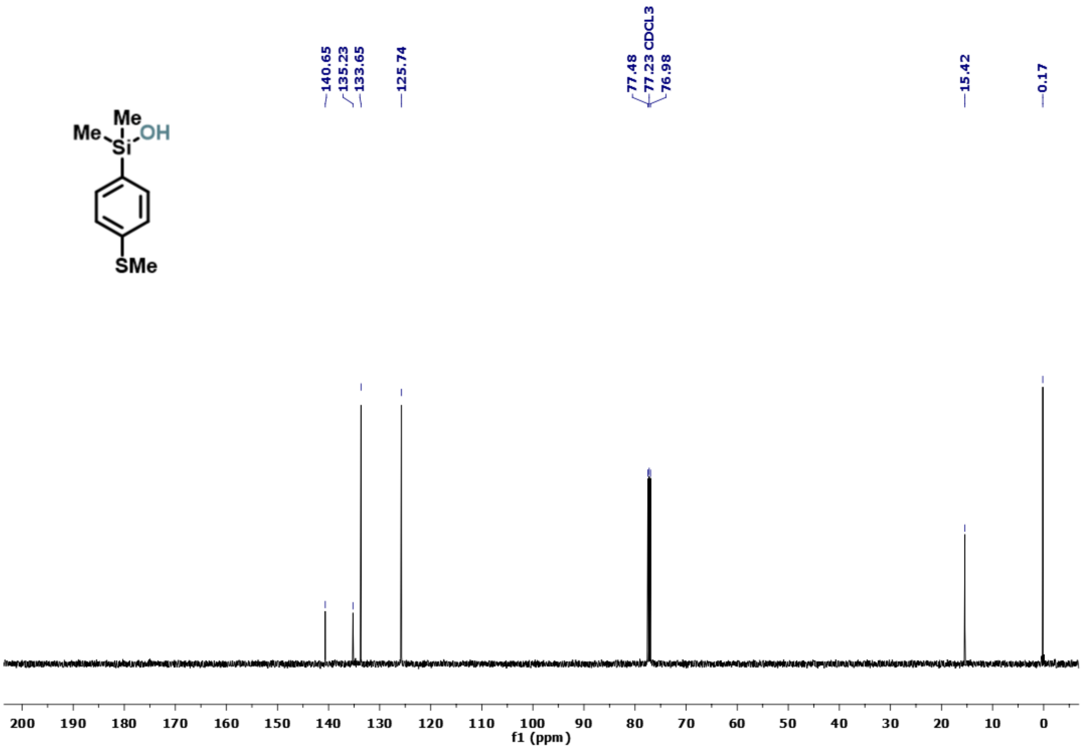

Dimethyl(4-(trifluoromethyl)phenyl)silanol (8):

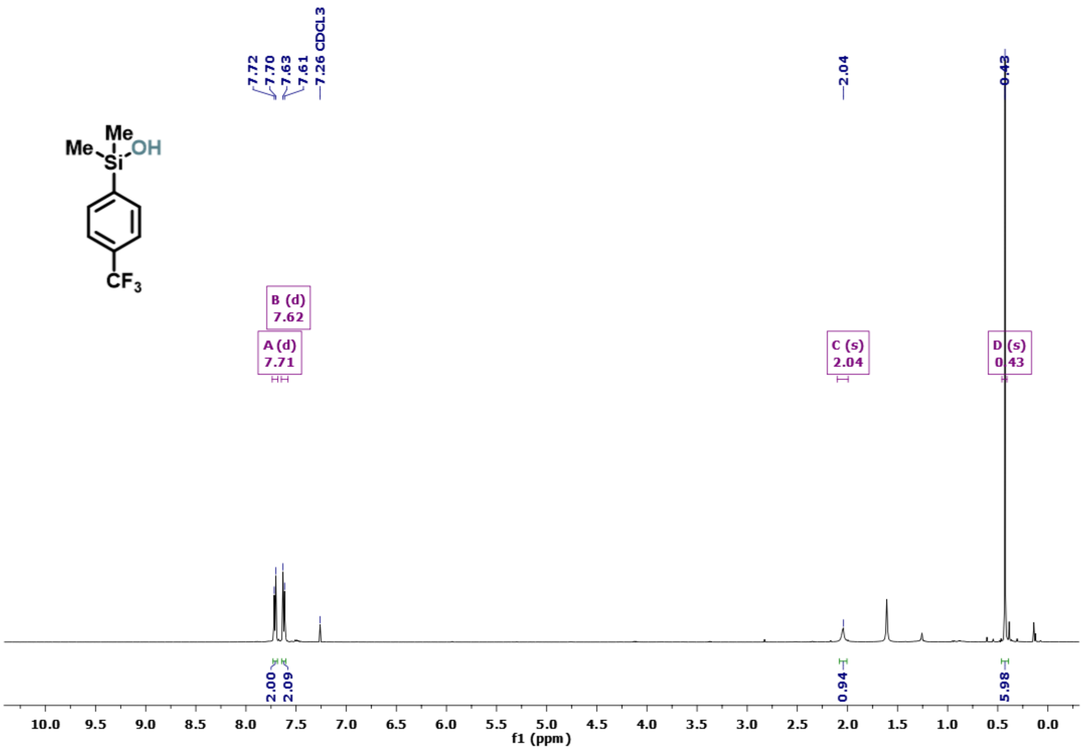

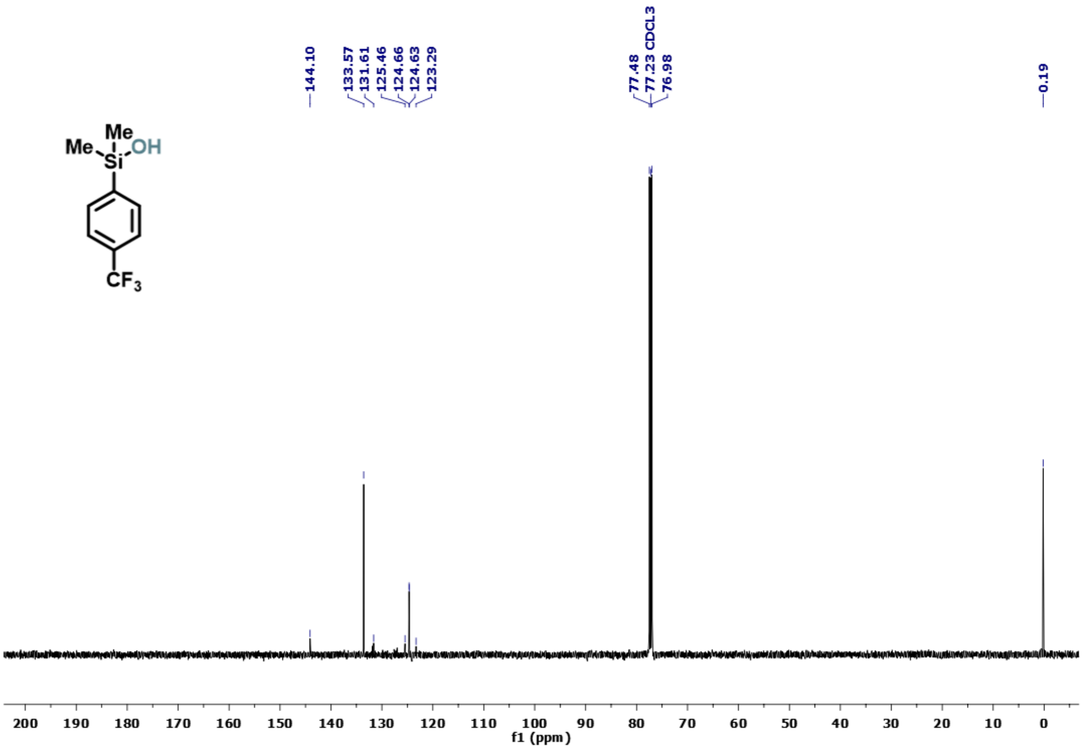

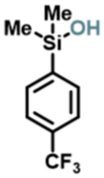

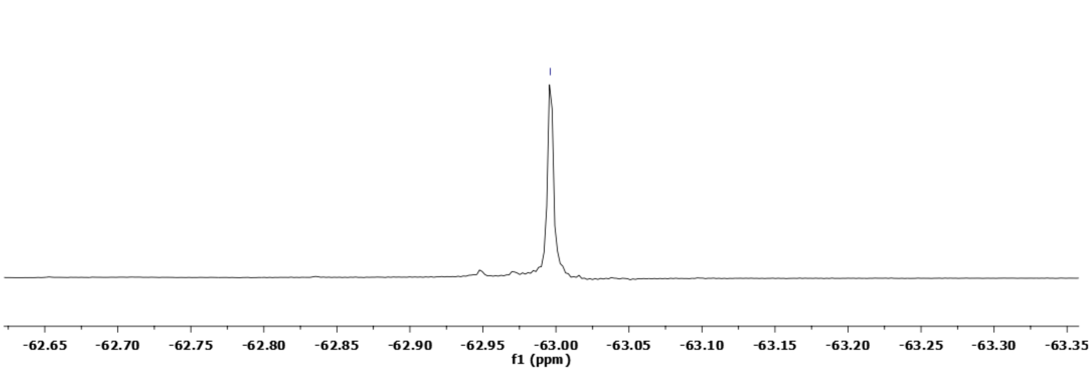
-63.00
-62.65
-62.70
-62.75
-62.80
-62.85
-62.90
-62.95
-63.00
-63.05
-63.10
-63.15
-63.20
-63.25
-63.30
-63.35
f1 (ppm)

(2-Fluoro-3-methoxyphenyl)dimethylsilanol (10):

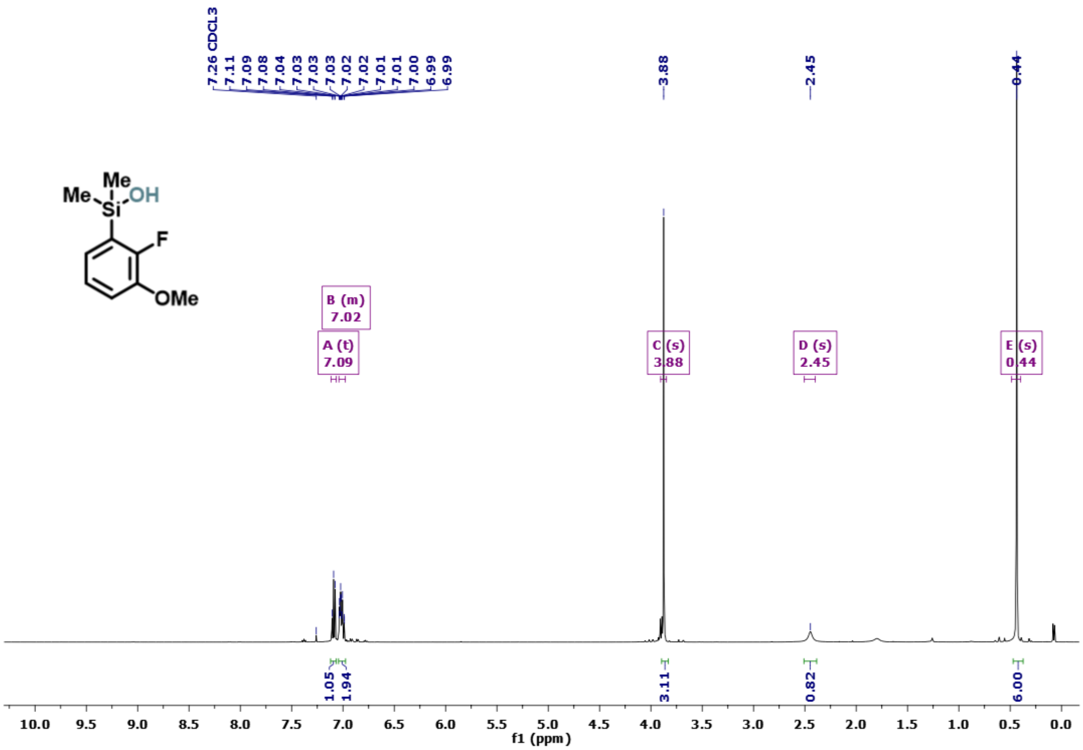

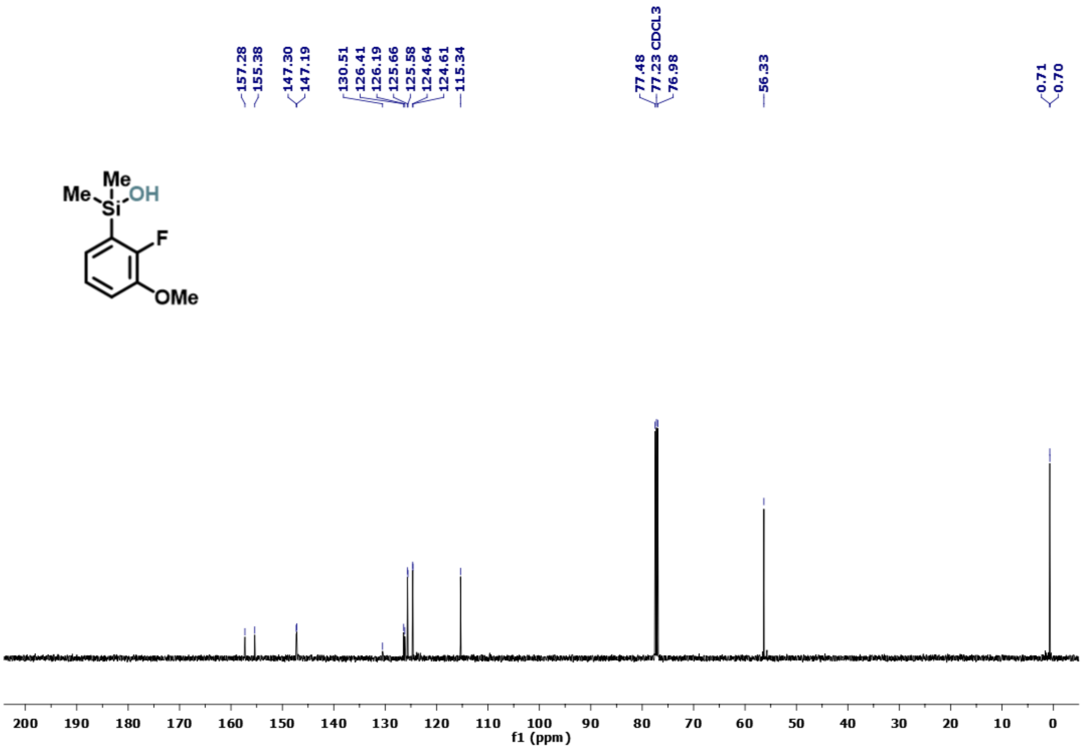

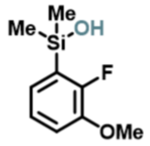

Me
Me
Si
OH
F
OMe

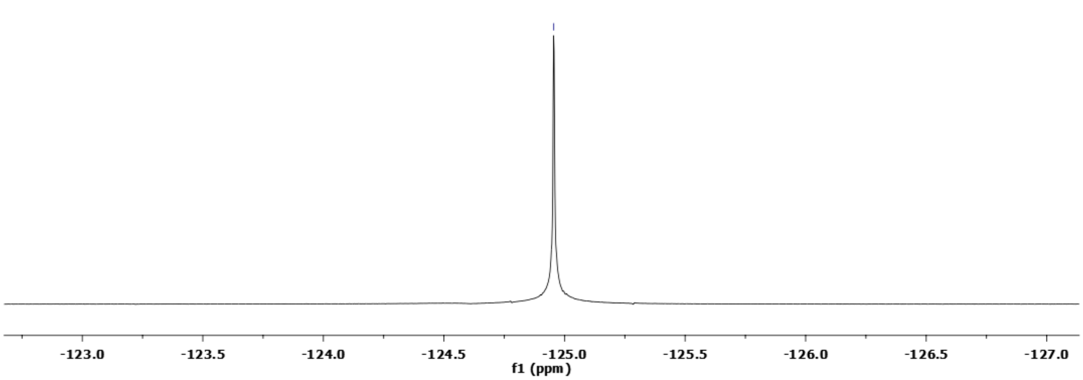

-124.96
-123.0
-123.5
-124.0
-124.5
-125.0
-125.5
-126.0
-126.5
-127.0
f1 (ppm)

Anthracen-9-yldimethylsilanol (12):

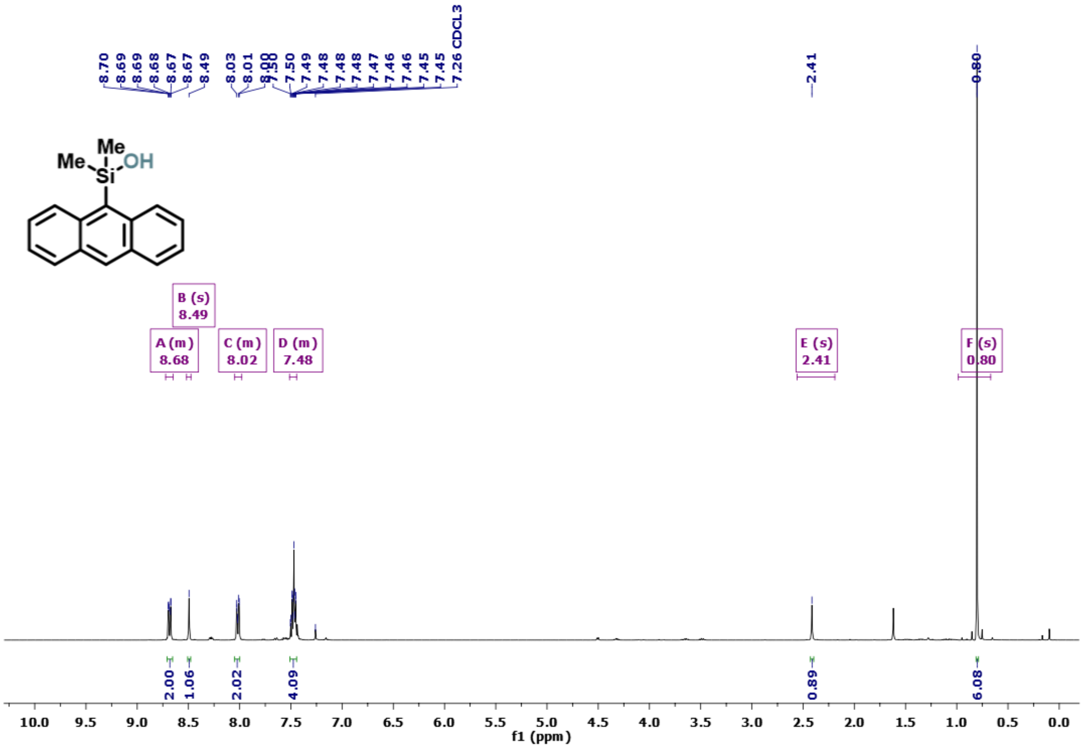

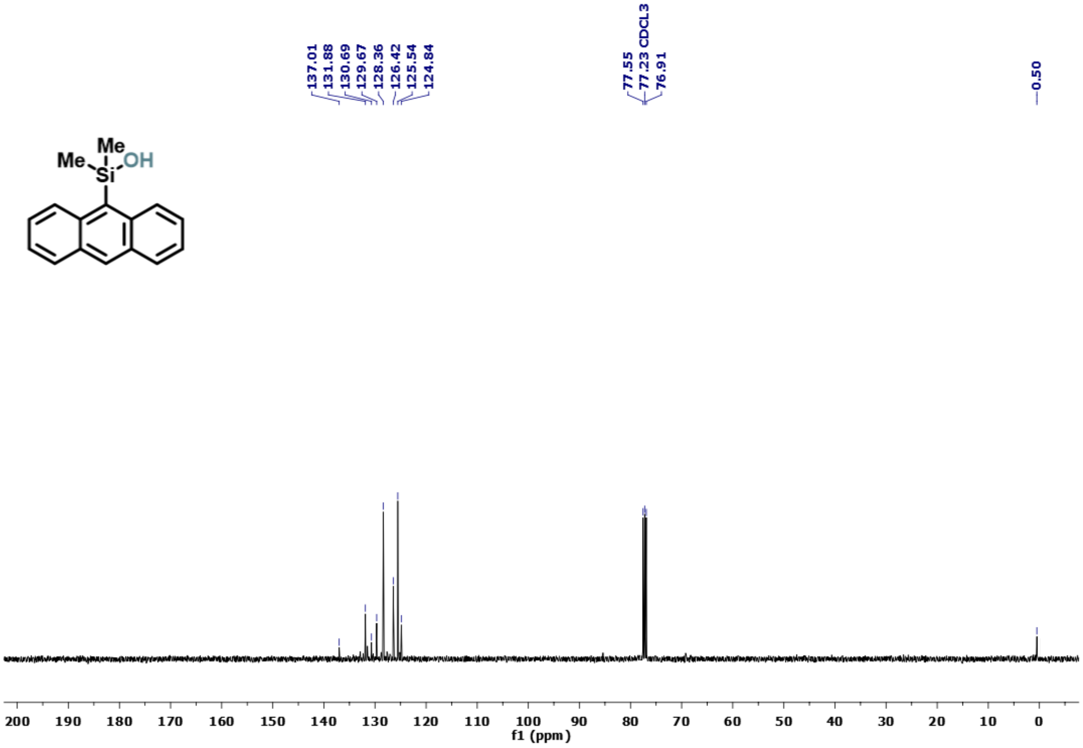

Methyldiphenylsilanol (13):

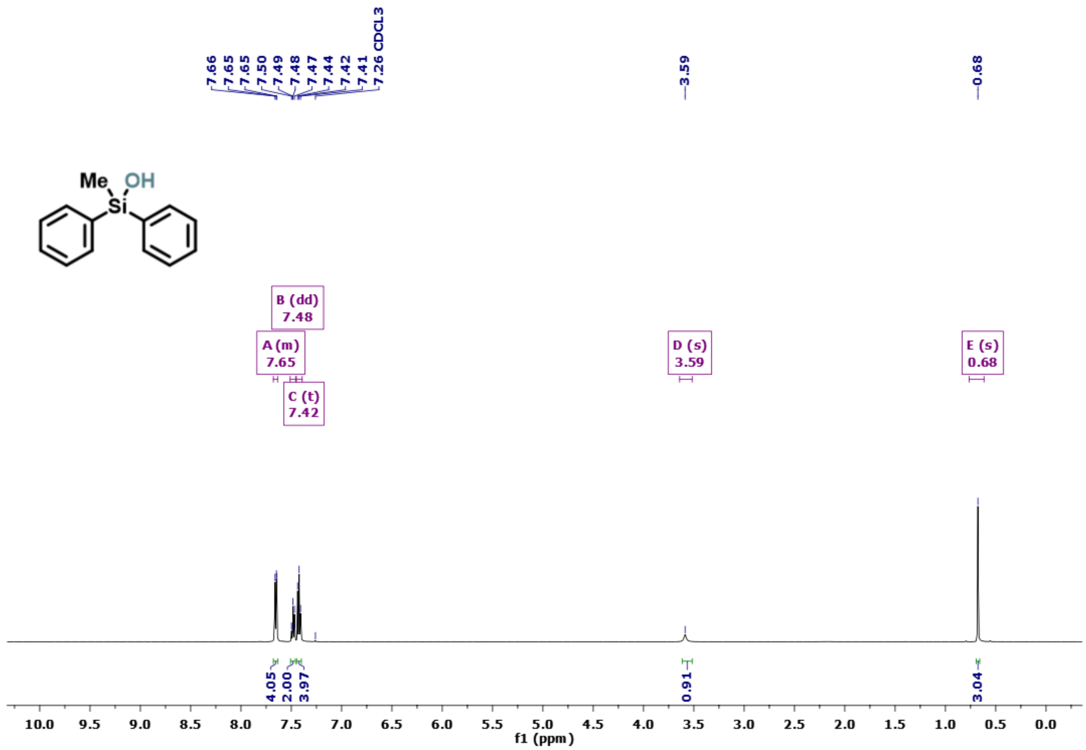

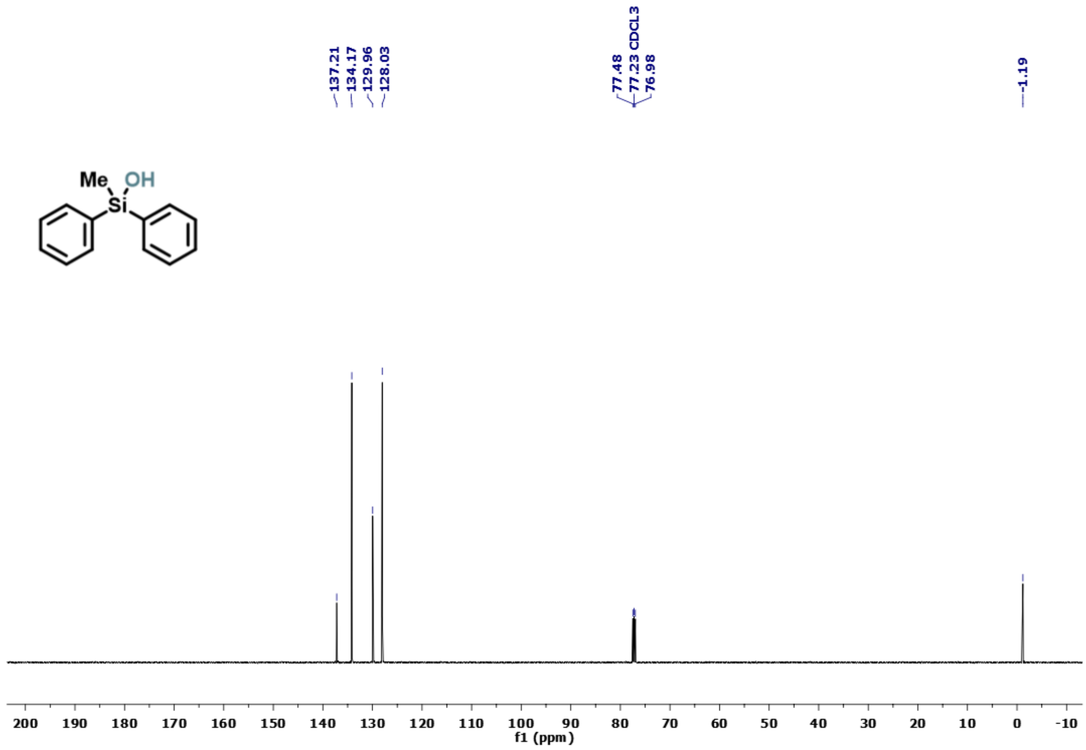

Tert-butoxydiphenylsilanol (14):

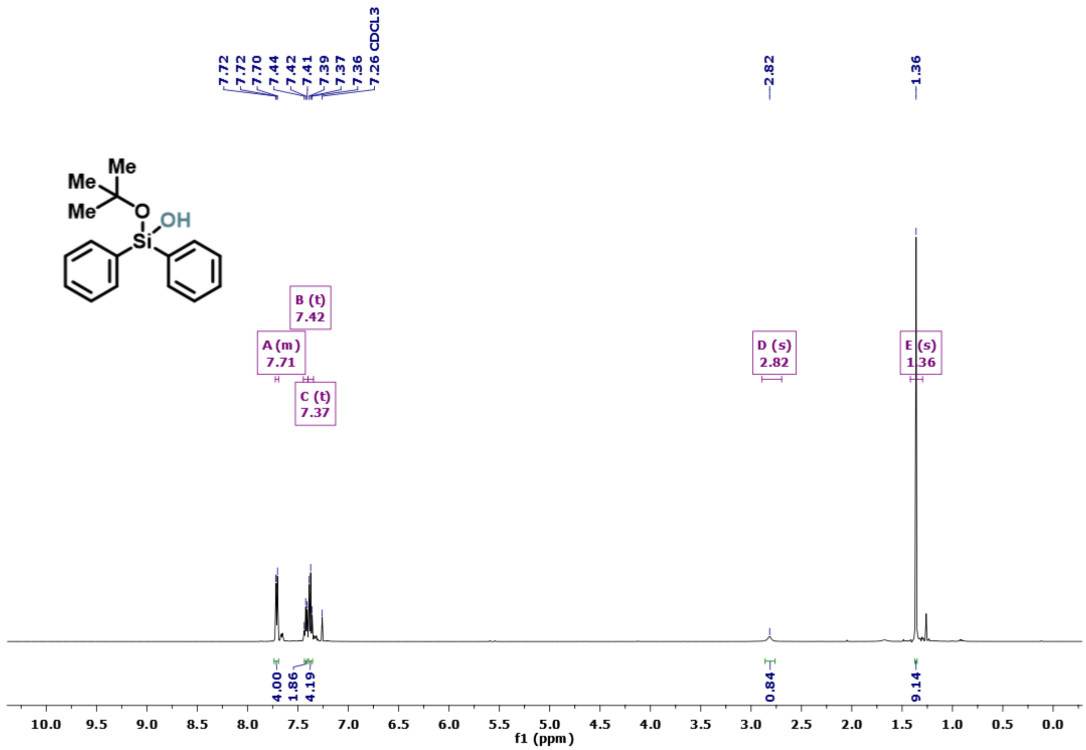

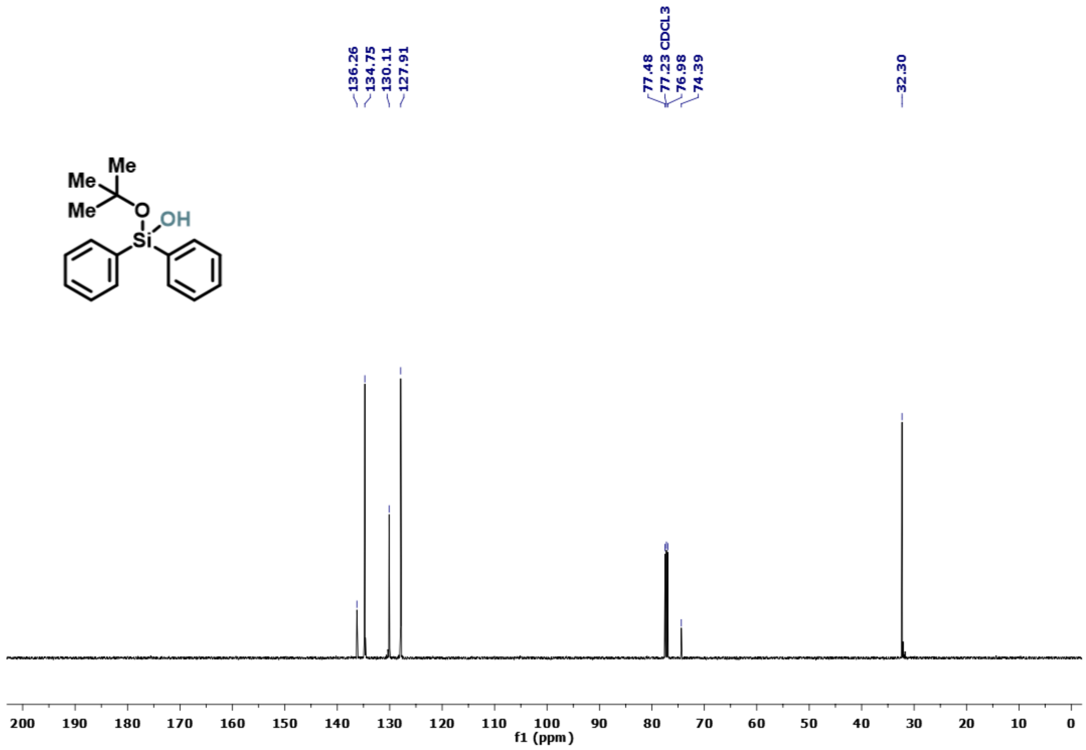

Triphenylsilanol (15):

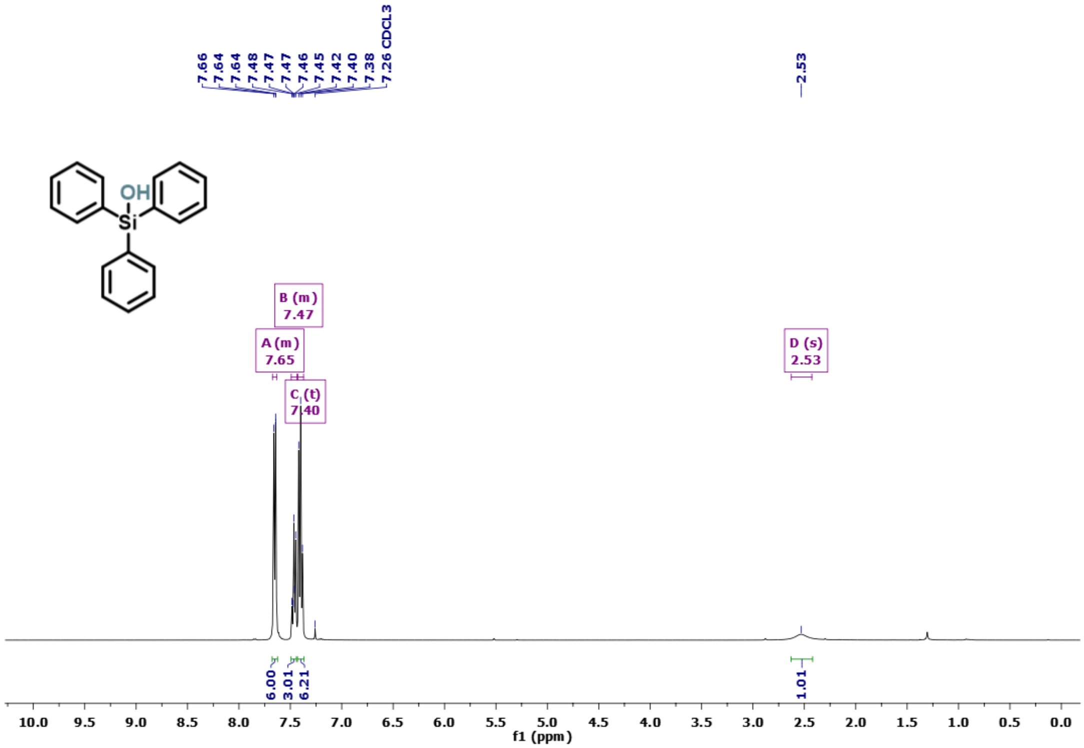

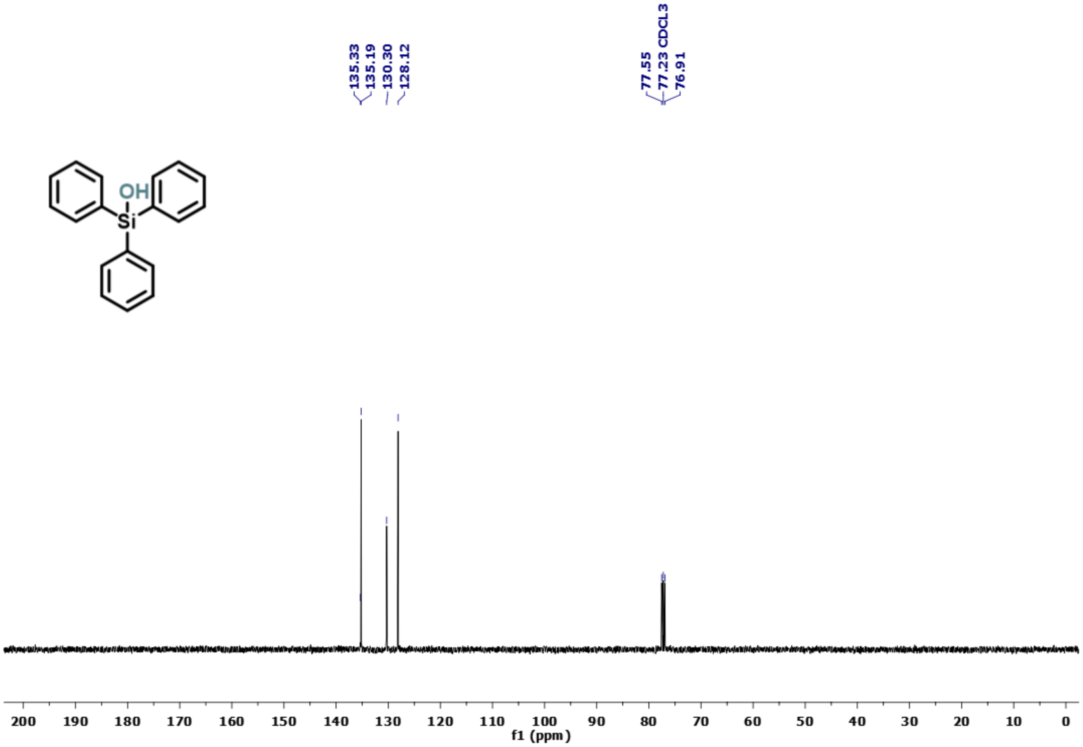

Benzyldiisopropylsilanol (16):

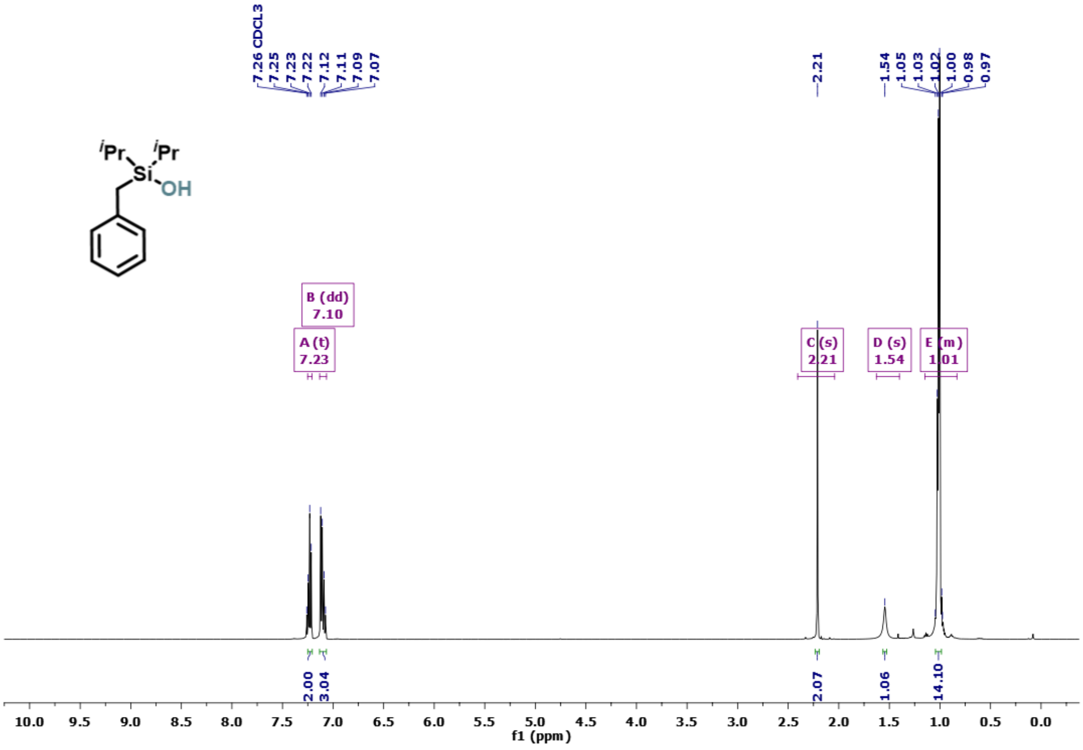

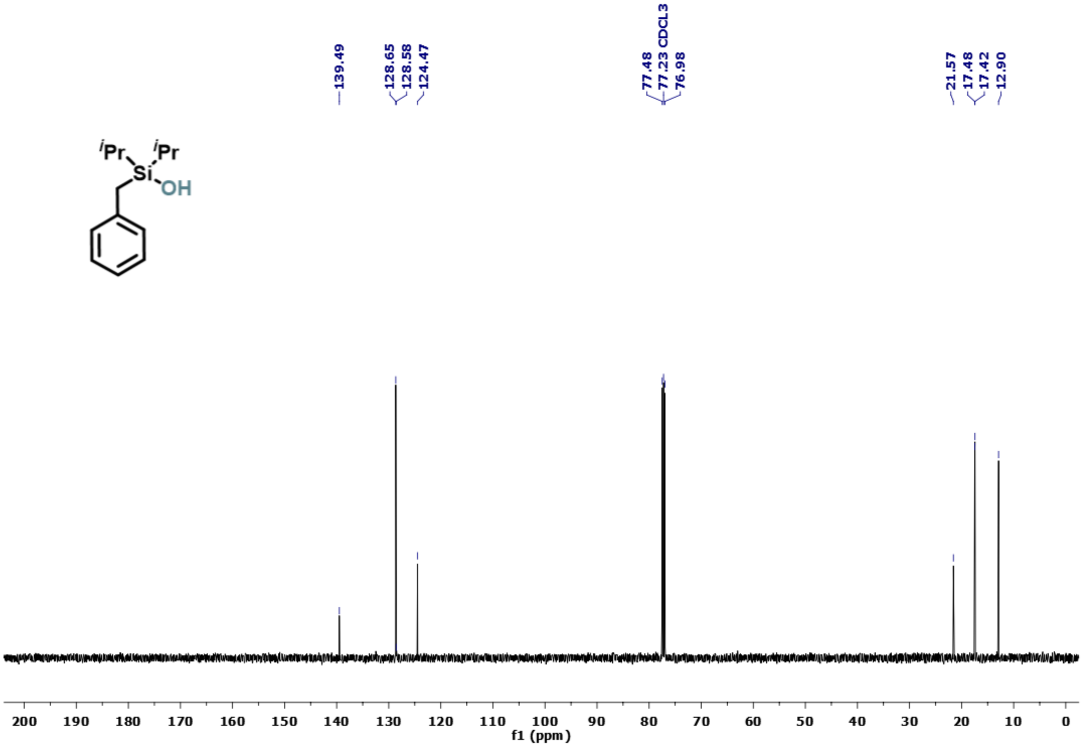

(2-fluorobenzyl)diisopropylsilanol (17):

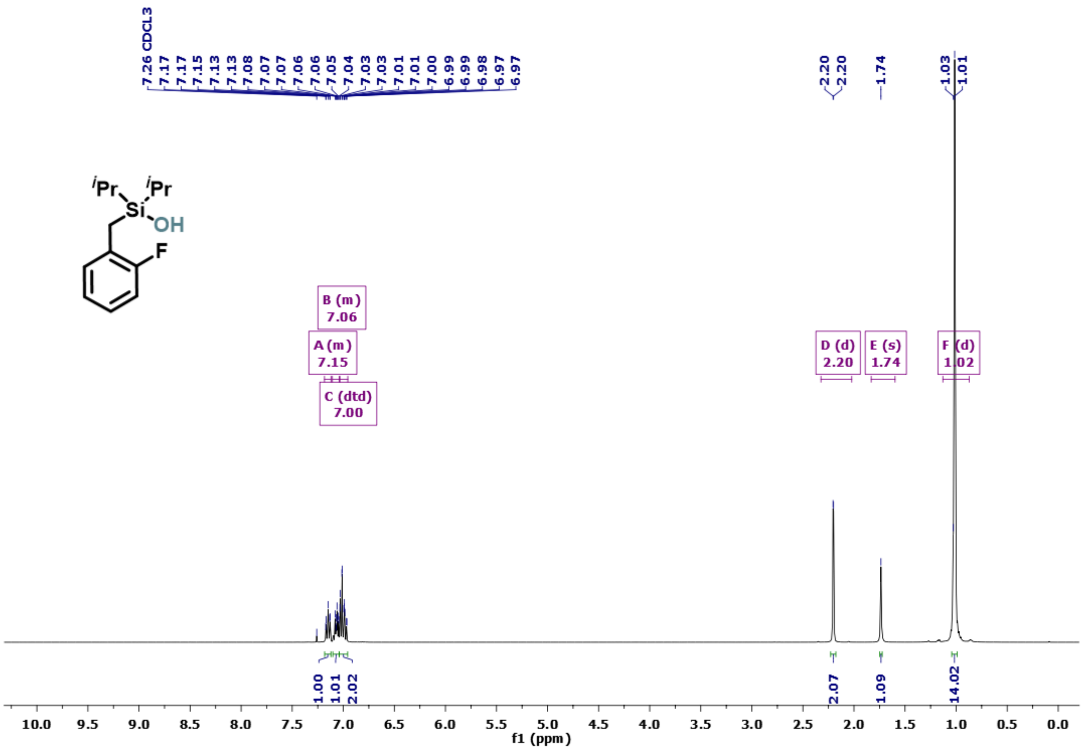

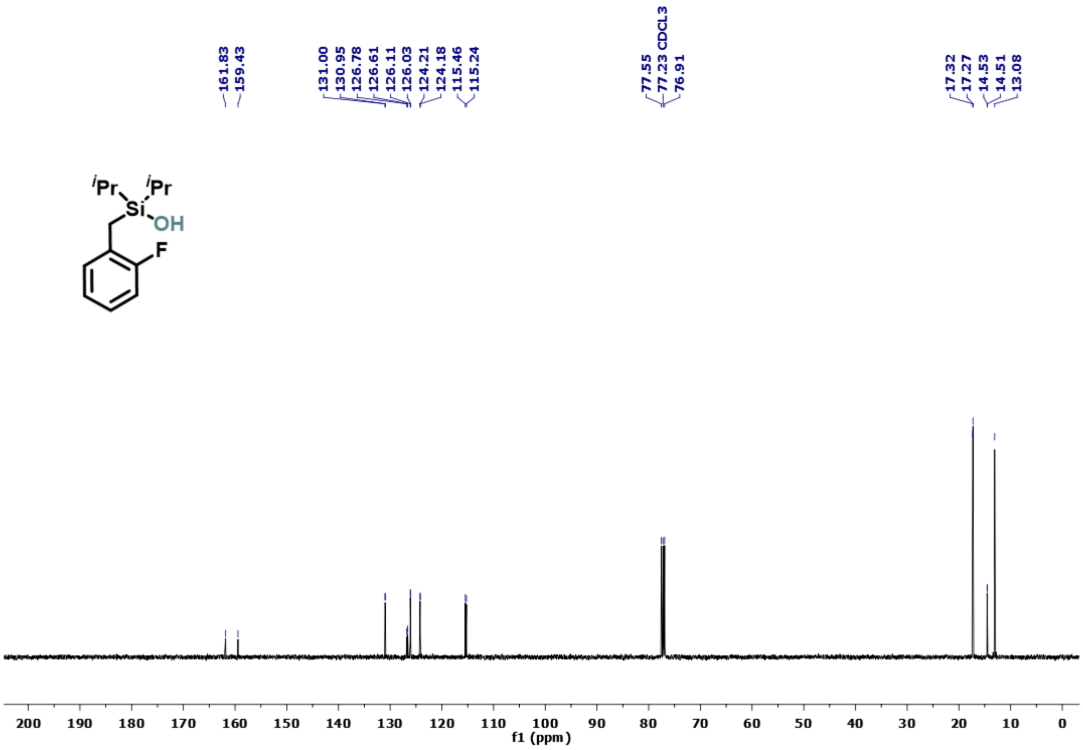

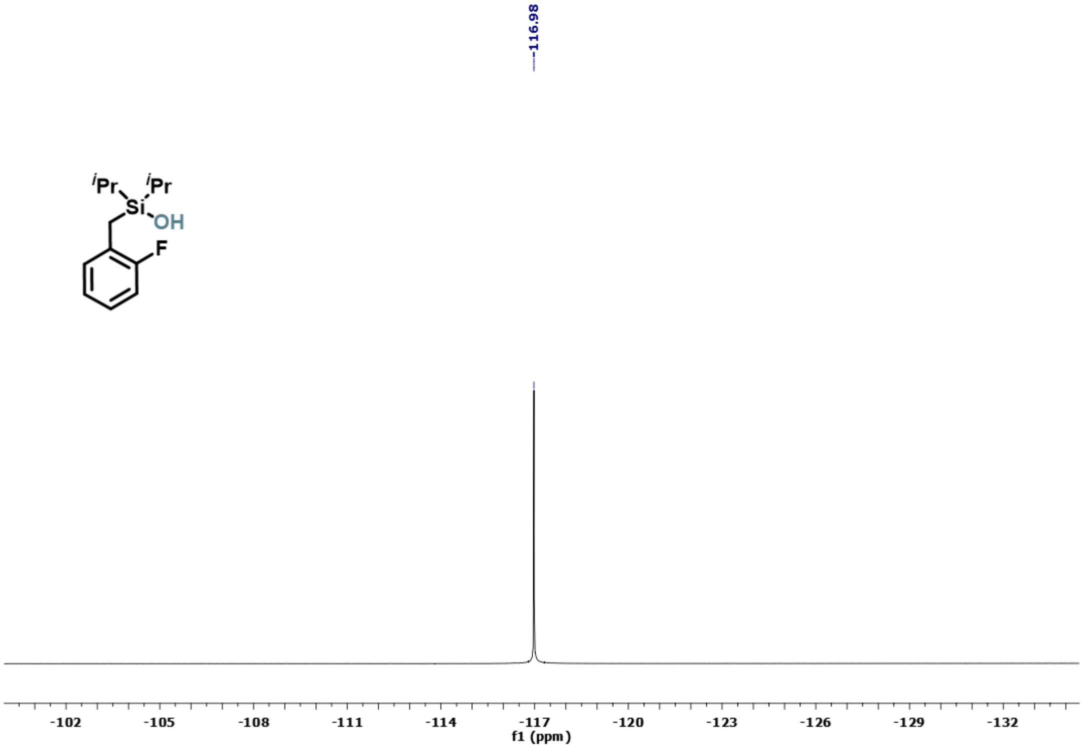
-116.98
iPr
iPr
Si
OH
F
-102
-105
-108
-111
-114
-117
-120
-123
-126
-129
-132
f1 (ppm)

Diisopropyl(3-(trifluoromethoxy)benzyl)silanol (18):

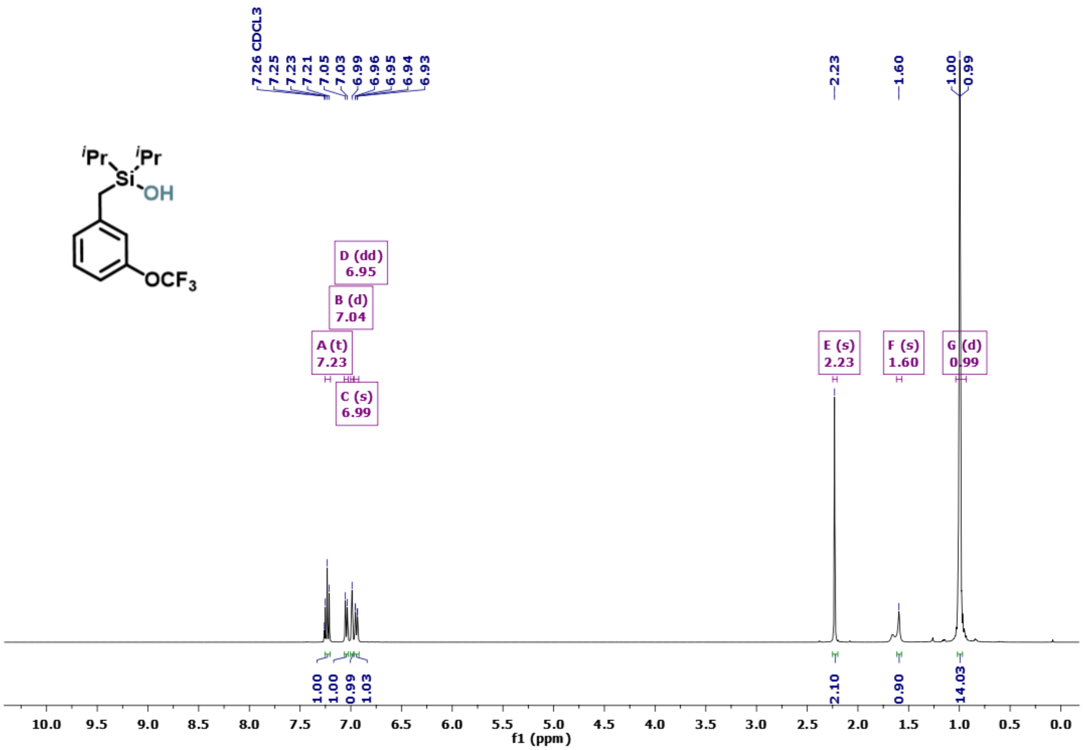

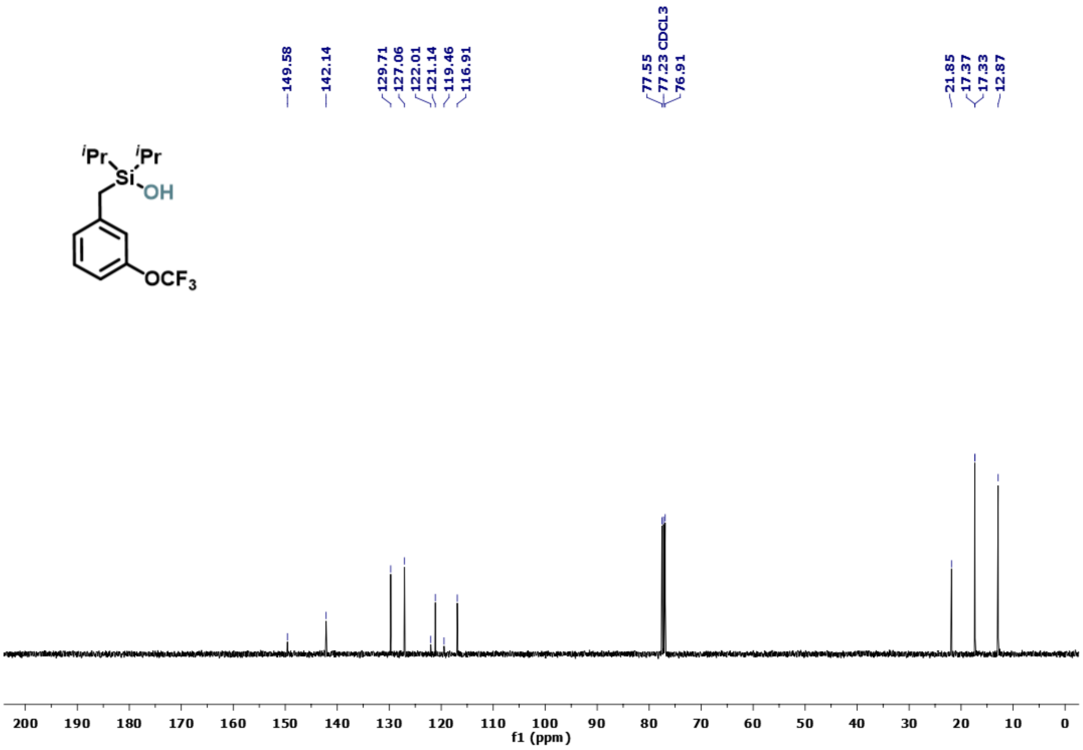

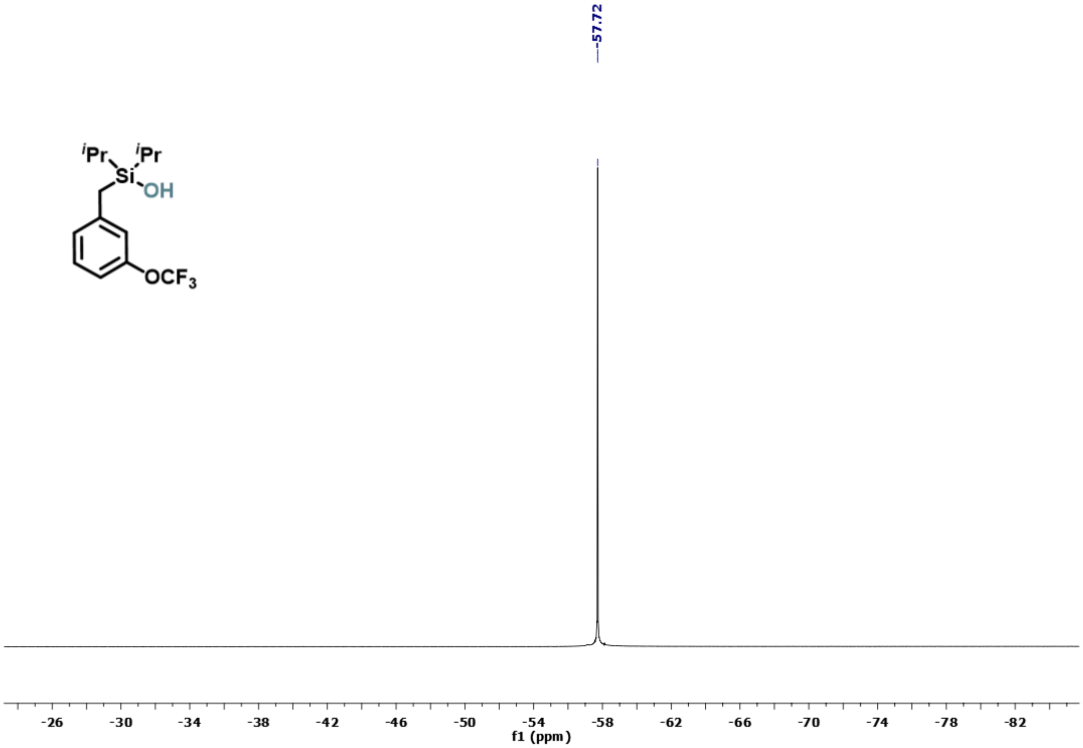

iPr
iPr
Si
OH
OCF3
-57.72
-26
-30
-34
-38
-42
-46
-50
-54
-58
-62
-66
-70
-74
-78
-82
f1 (ppm)

4-((hydroxydiisopropylsilyl)methyl)benzonitrile (19):

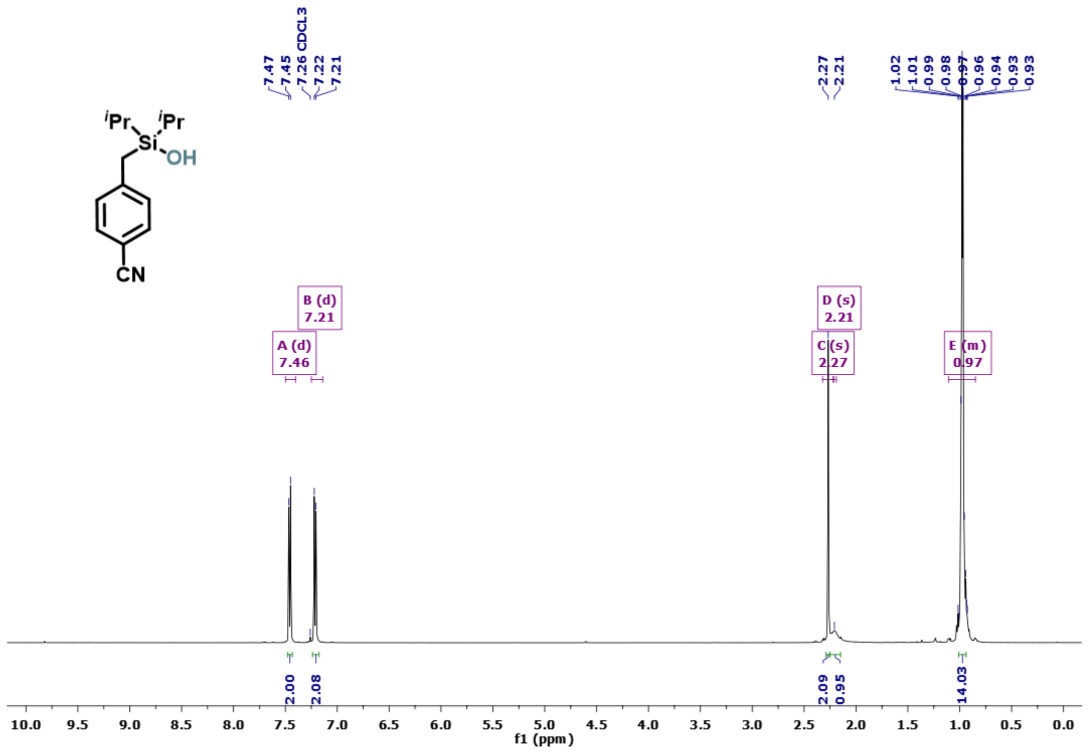

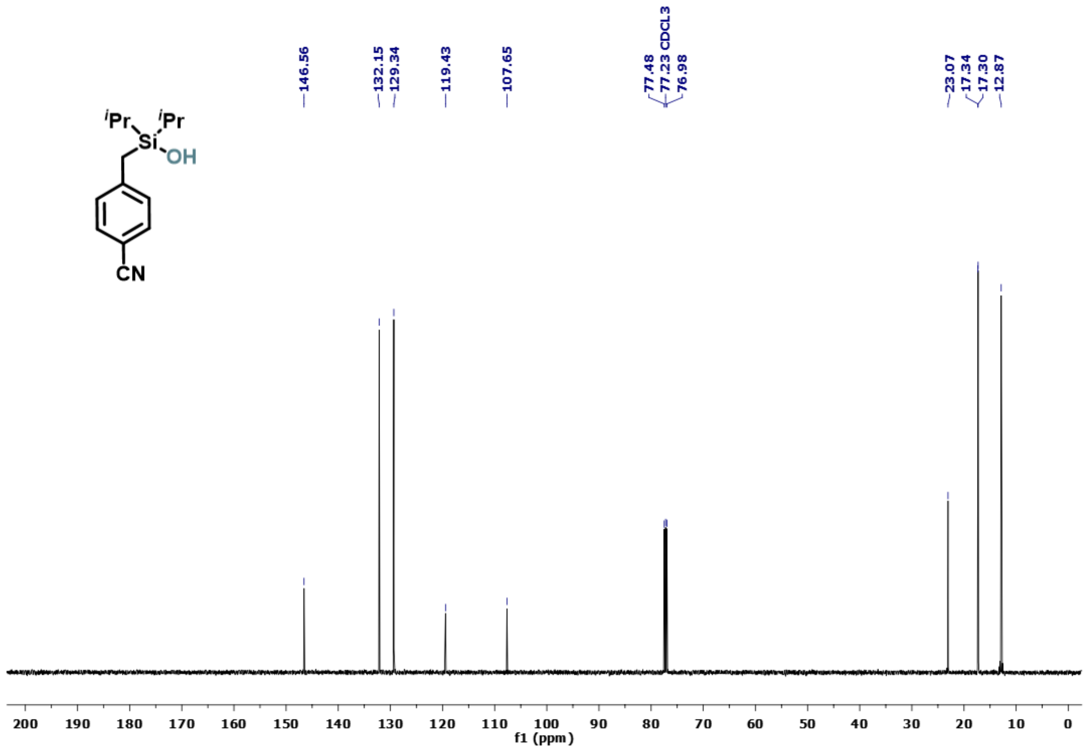

(2,3-dimethylbenzyl)diisopropylsilanol (20):

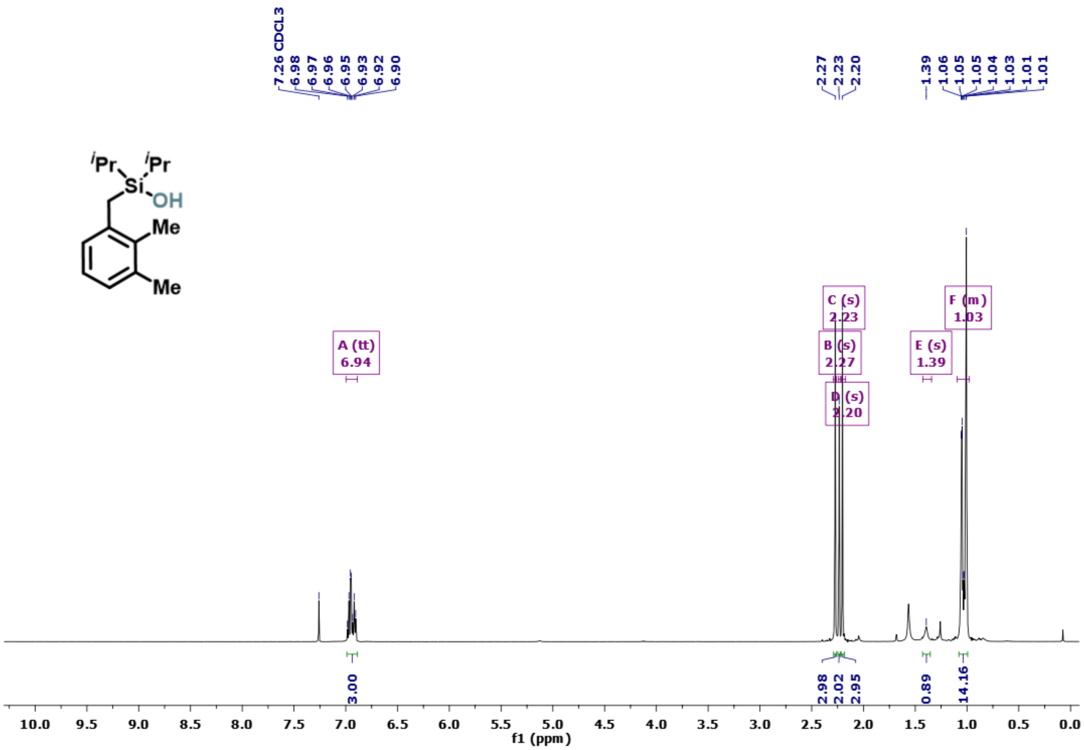

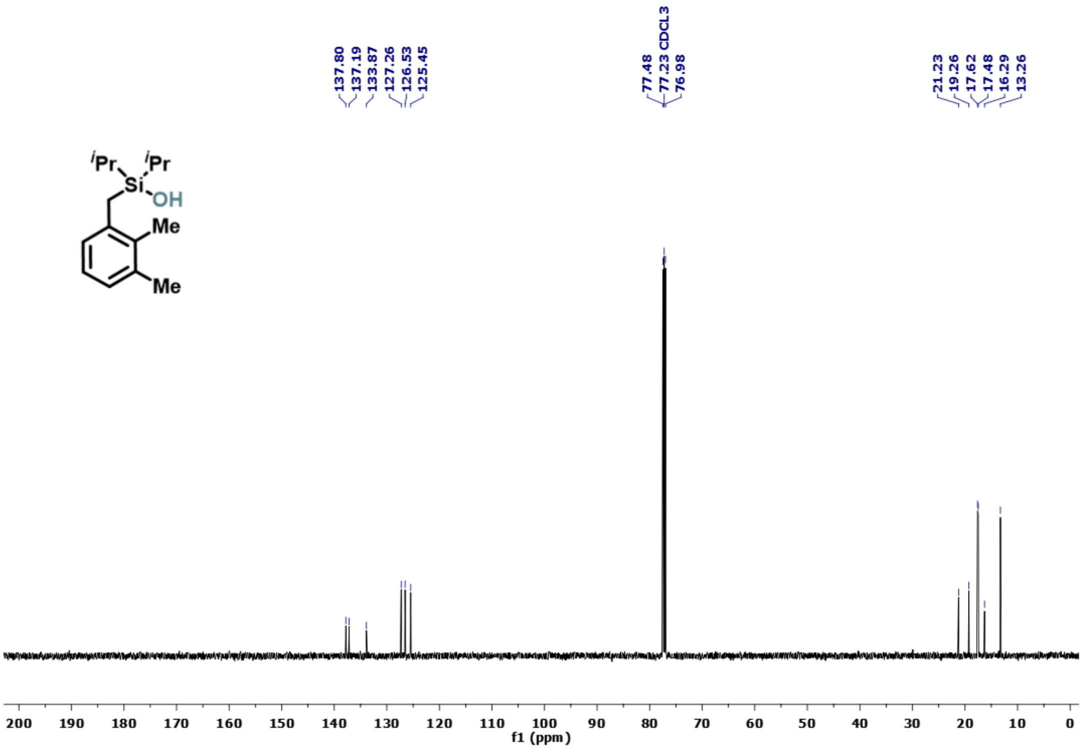

(2,3-Difluorobenzyl)diisopropylsilanol (21):

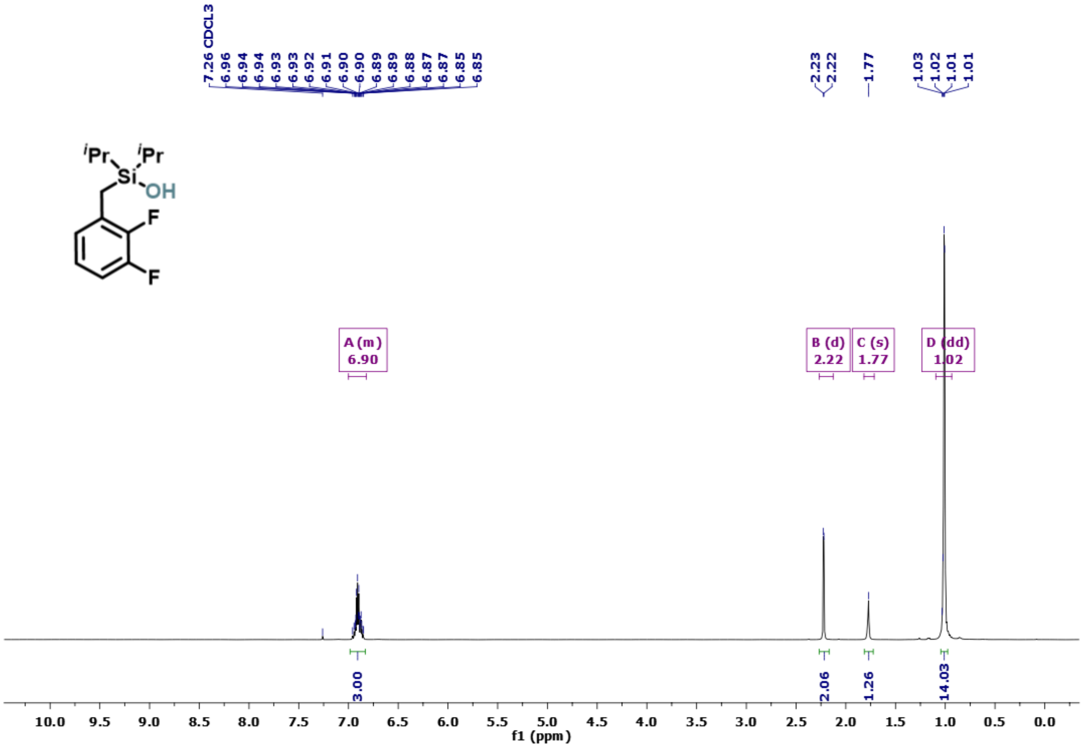

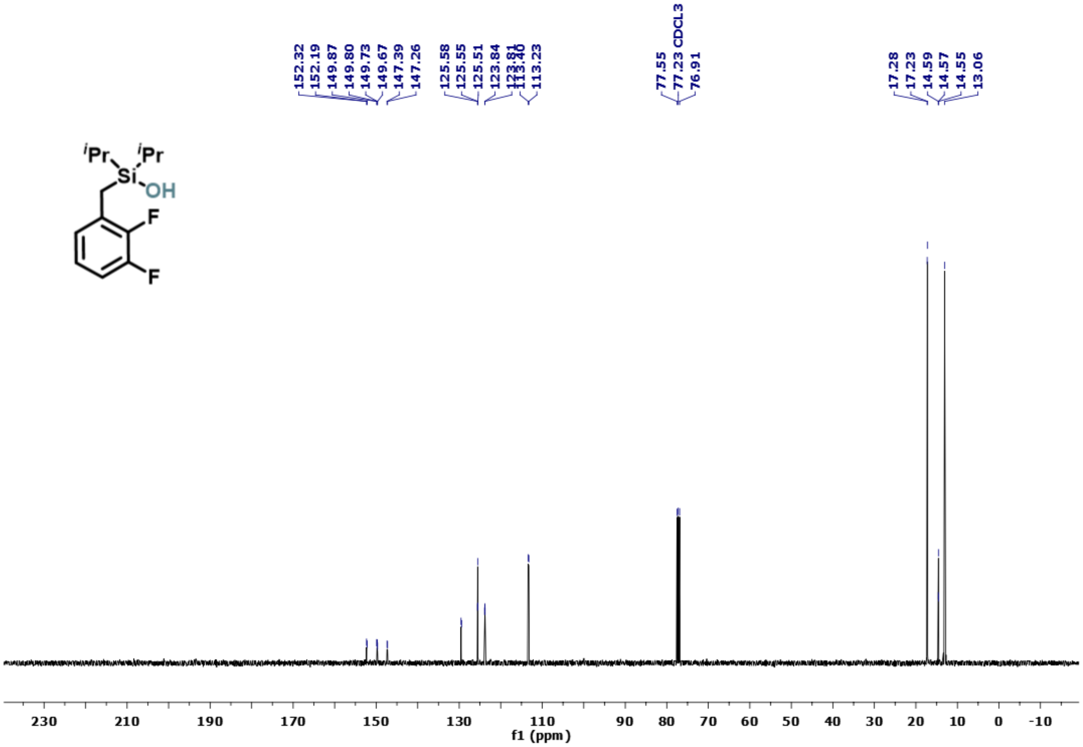

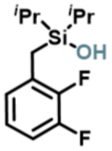

iPr
iPr
Si
OH
F
F

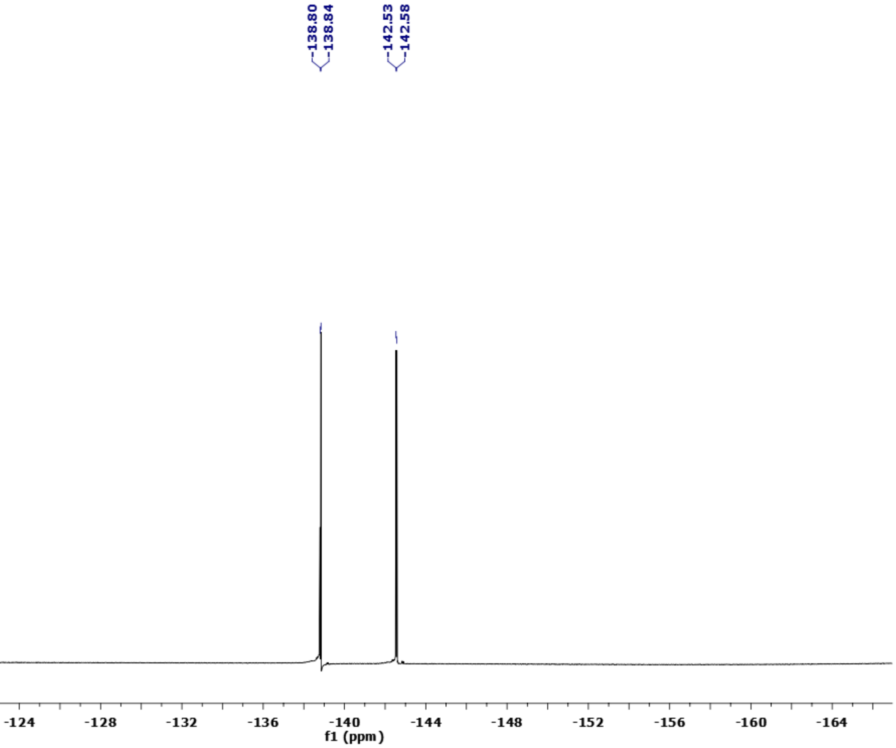

-138.80
-138.84
-142.53
-142.58
-116
-120
-124
-128
-132
-136
-140
-144
-148
-152
-156
-160
-164
f1 (ppm)

(5-fluoro-2-methoxybenzyl)dimethylsilanol (22):

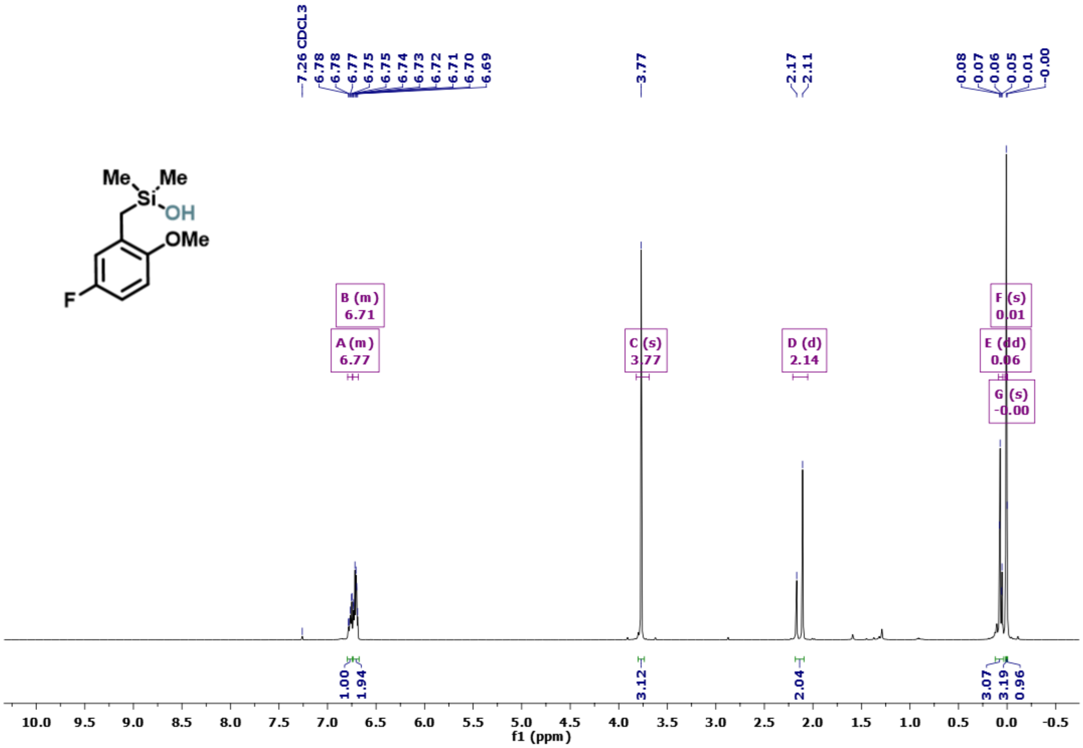

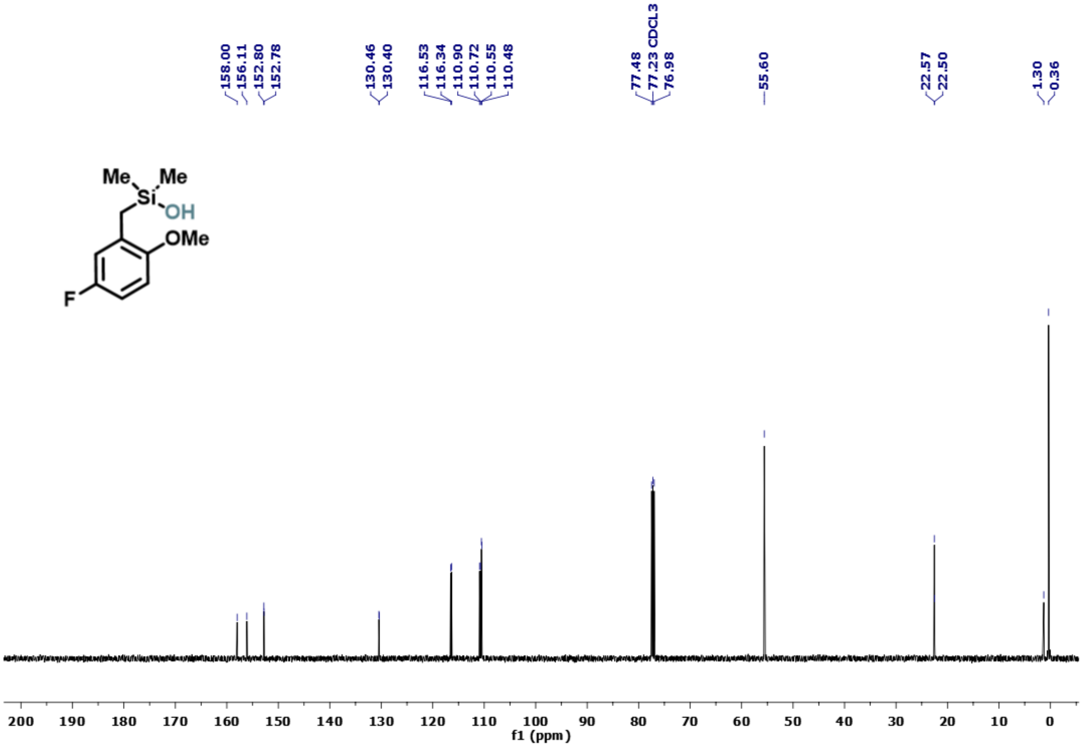

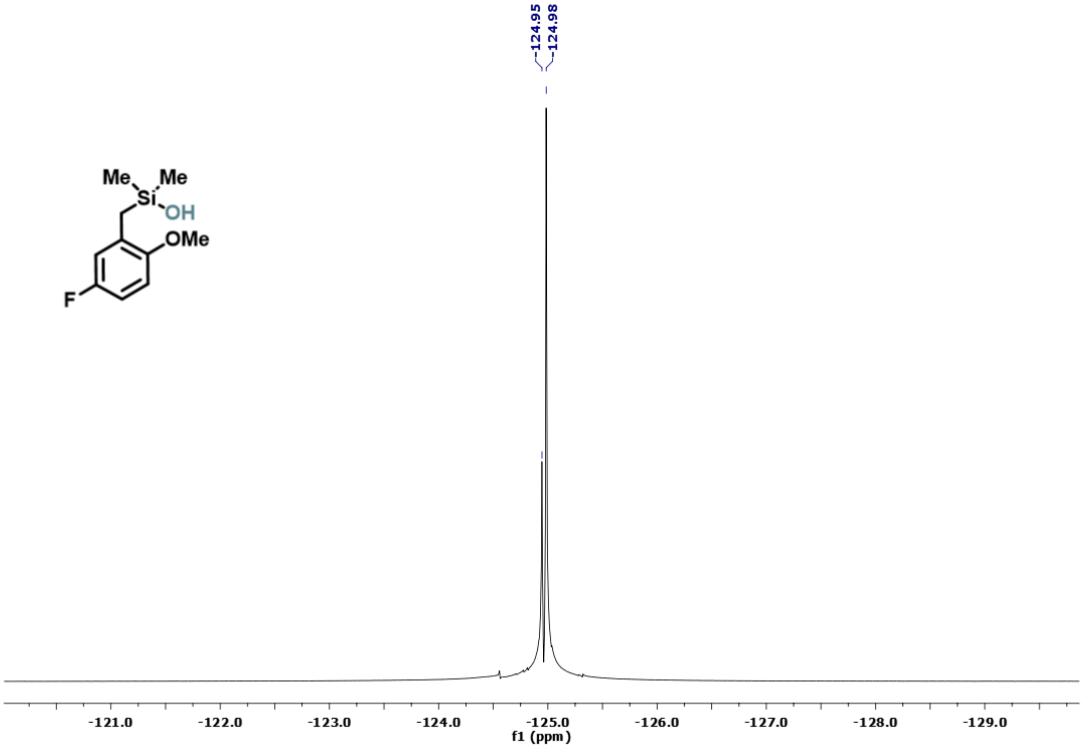

Me
Me
Si
OH
OMe
F
-124.95
-124.98
-121.0
-122.0
-123.0
-124.0
-125.0
-126.0
-127.0
-128.0
-129.0
f1 (ppm)

(2-Chloro-5-fluorobenzyl)dimethylsilanol (23):

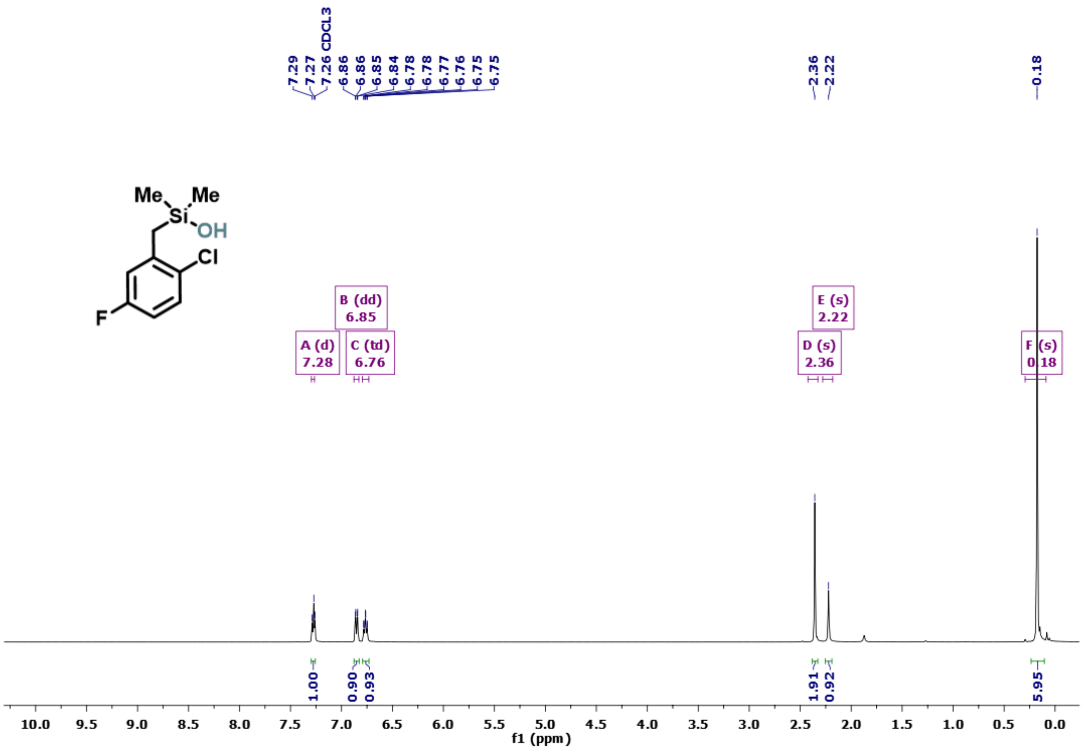

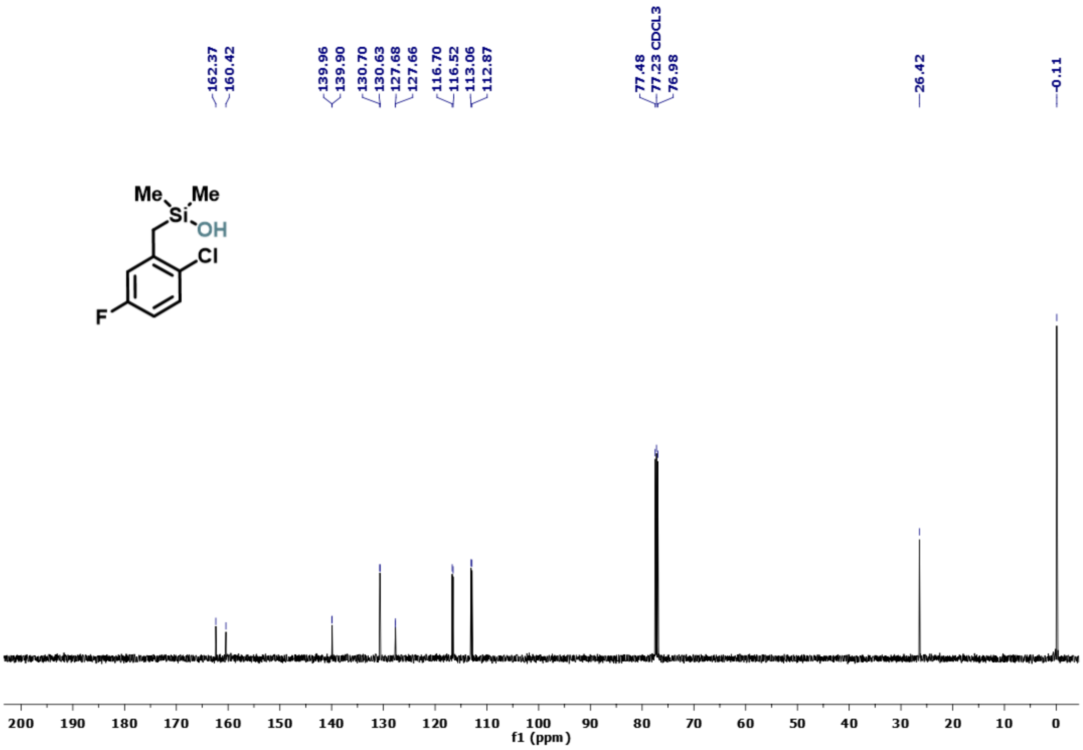

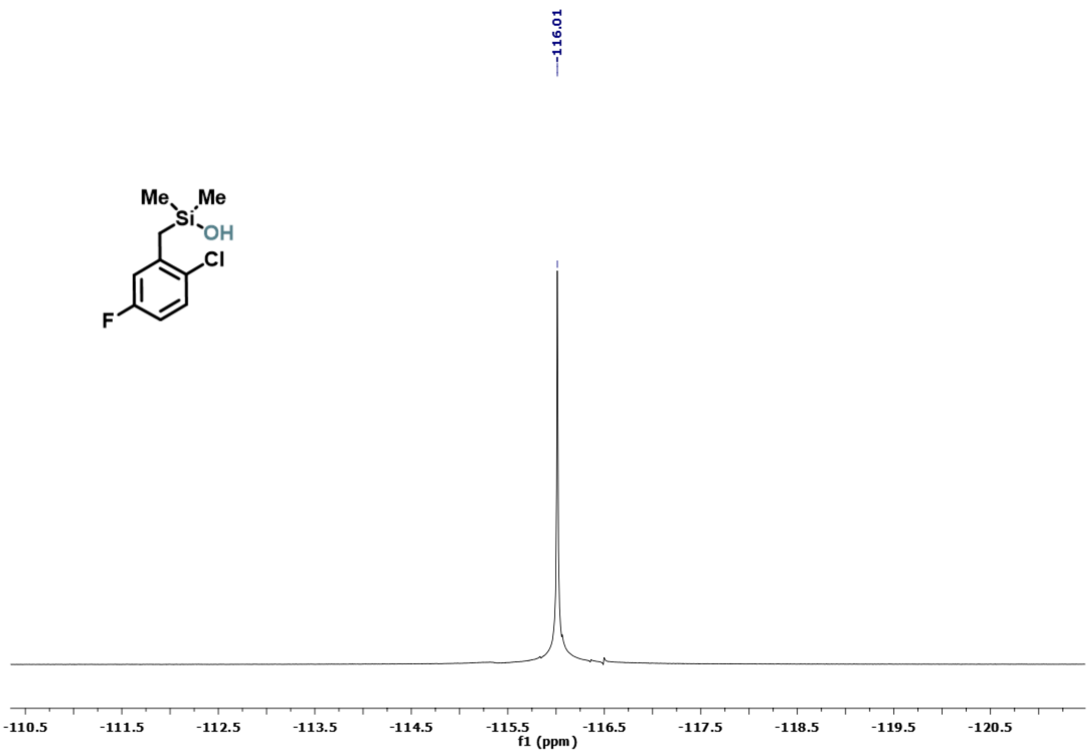

-116.01
Me
Me
Si
OH
Cl
F
-110.5
-111.5
-112.5
-113.5
-114.5
-115.5
-116.5
-117.5
-118.5
-119.5
-120.5
f1 (ppm)

(2,6-Dimethylbenzyl)diisopropylsilanol (24):

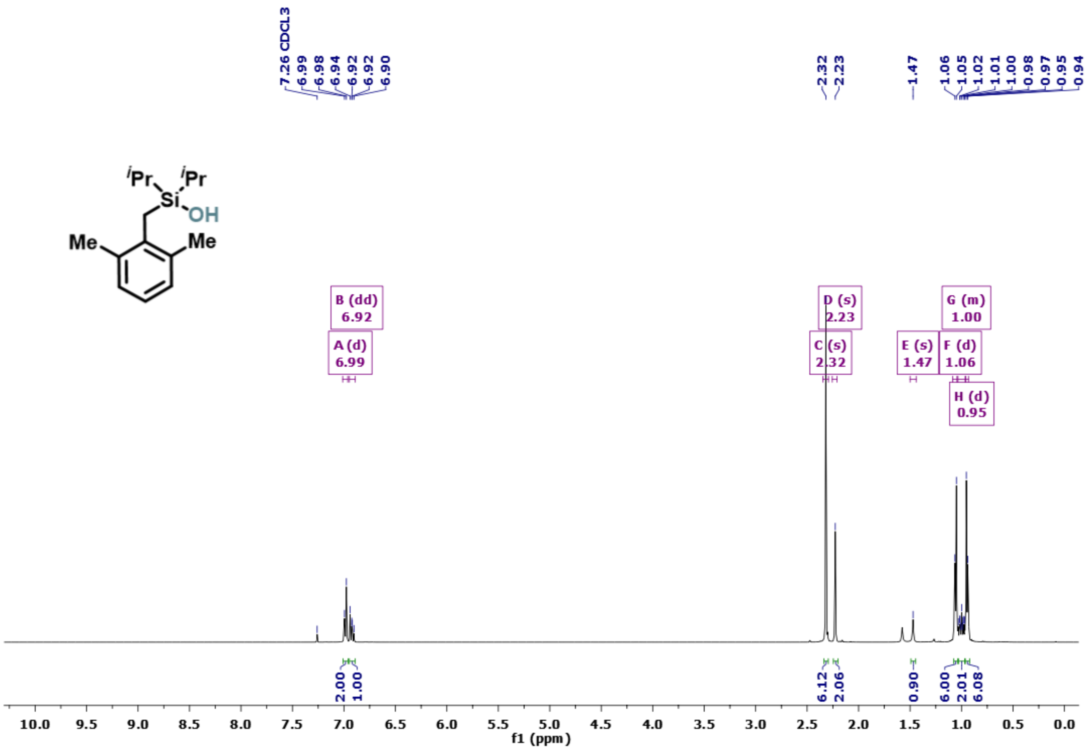

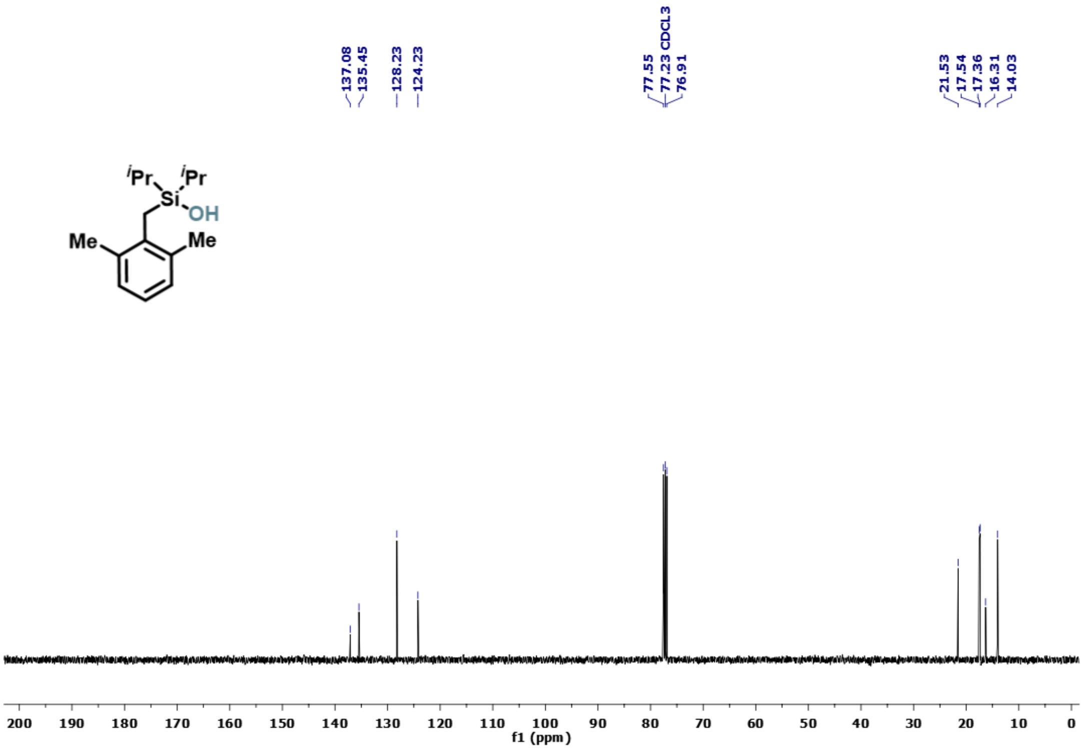

Benzhydryldiisopropylsilanol (25):

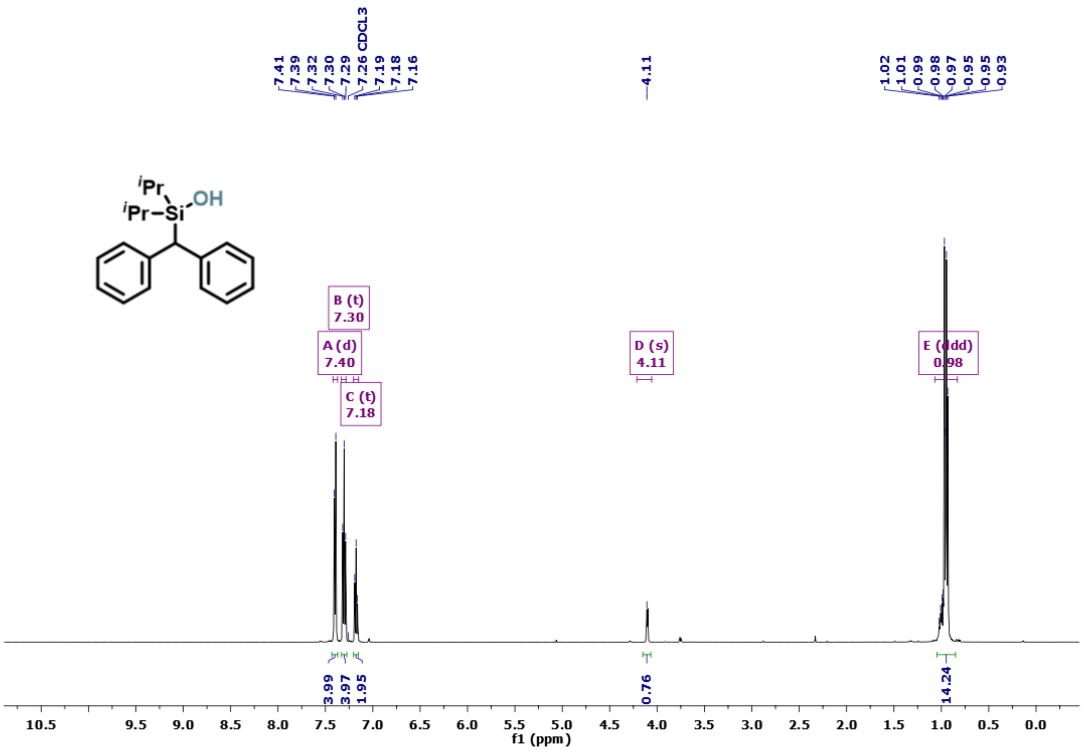

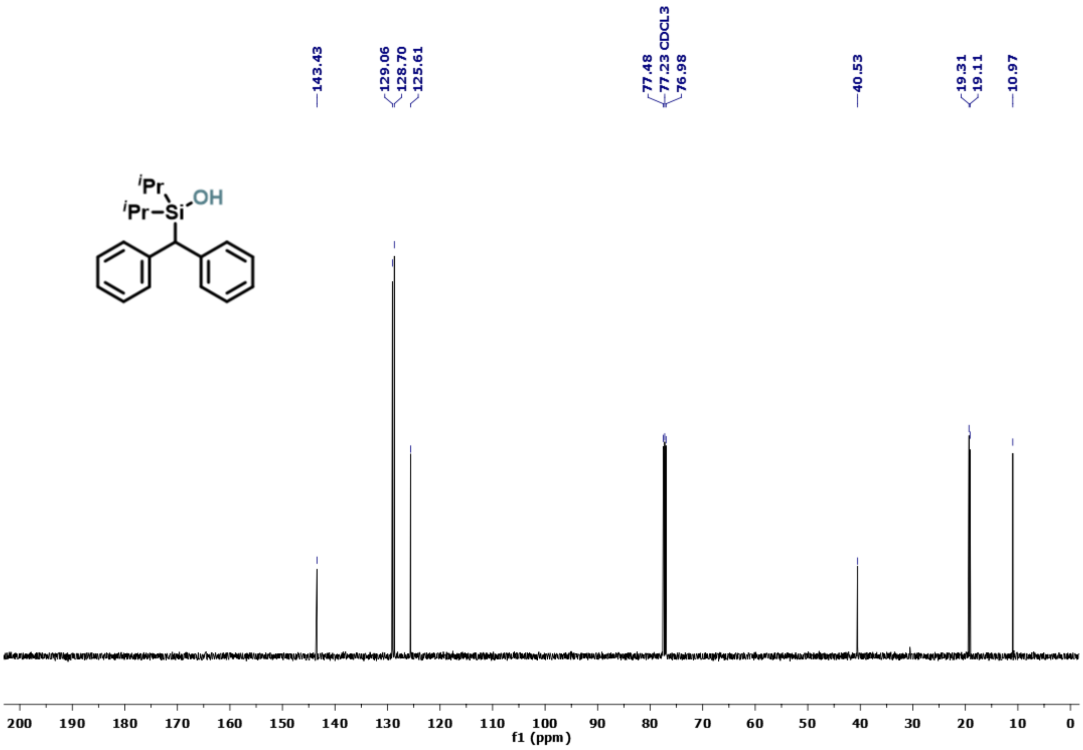

(9H-fluoren-9-yl)diisopropylsilanol (26):

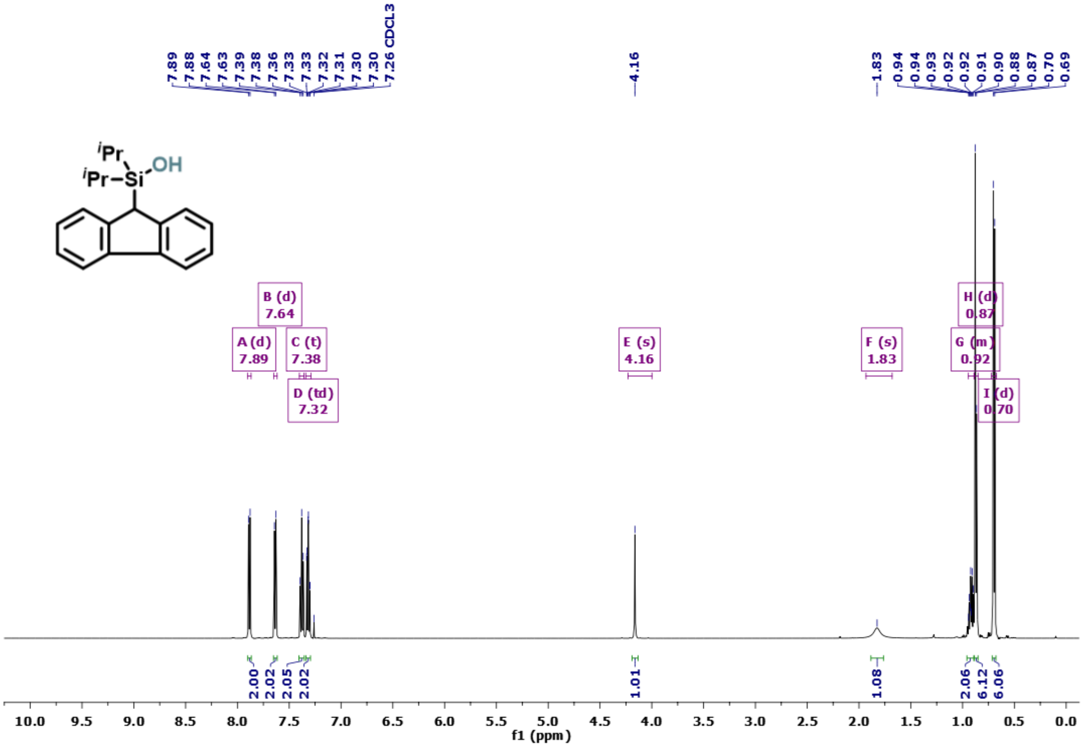

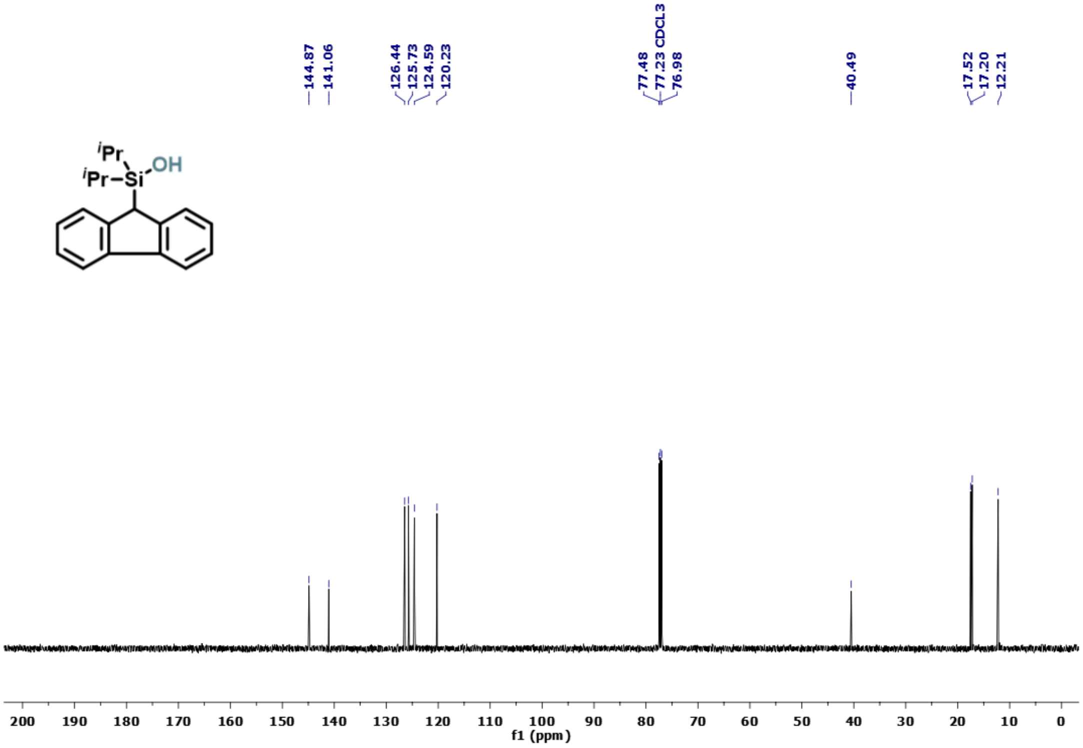

Tribenzylsilanol (27):

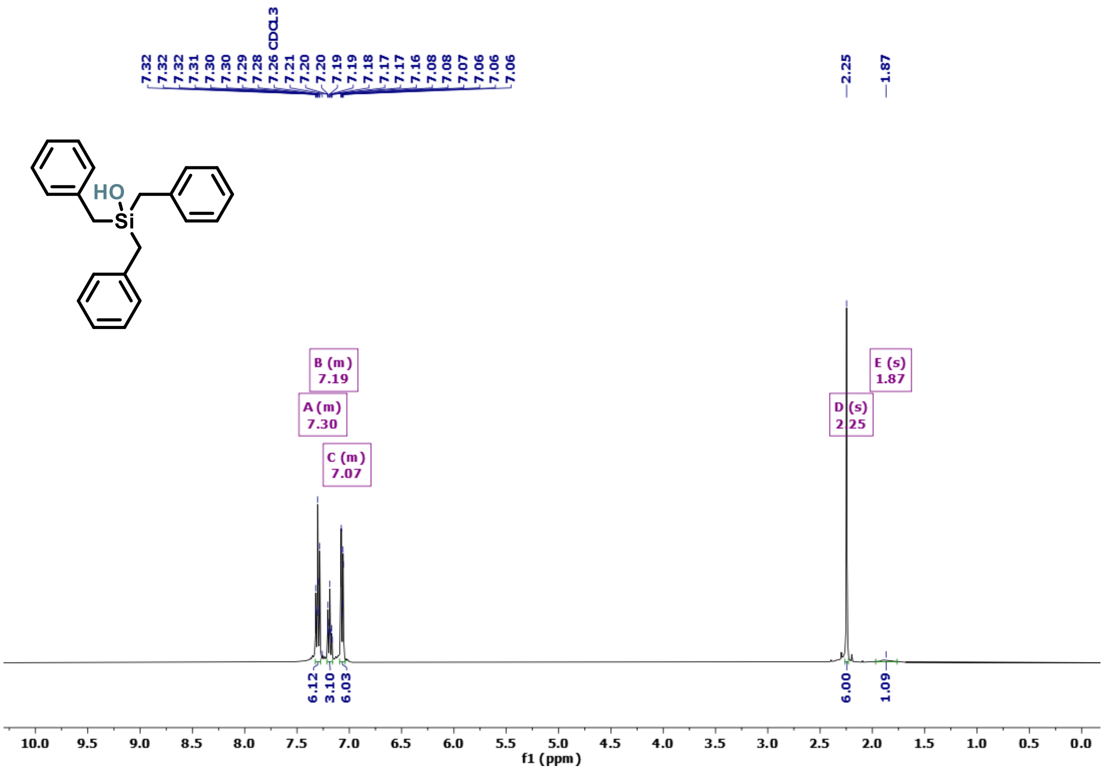

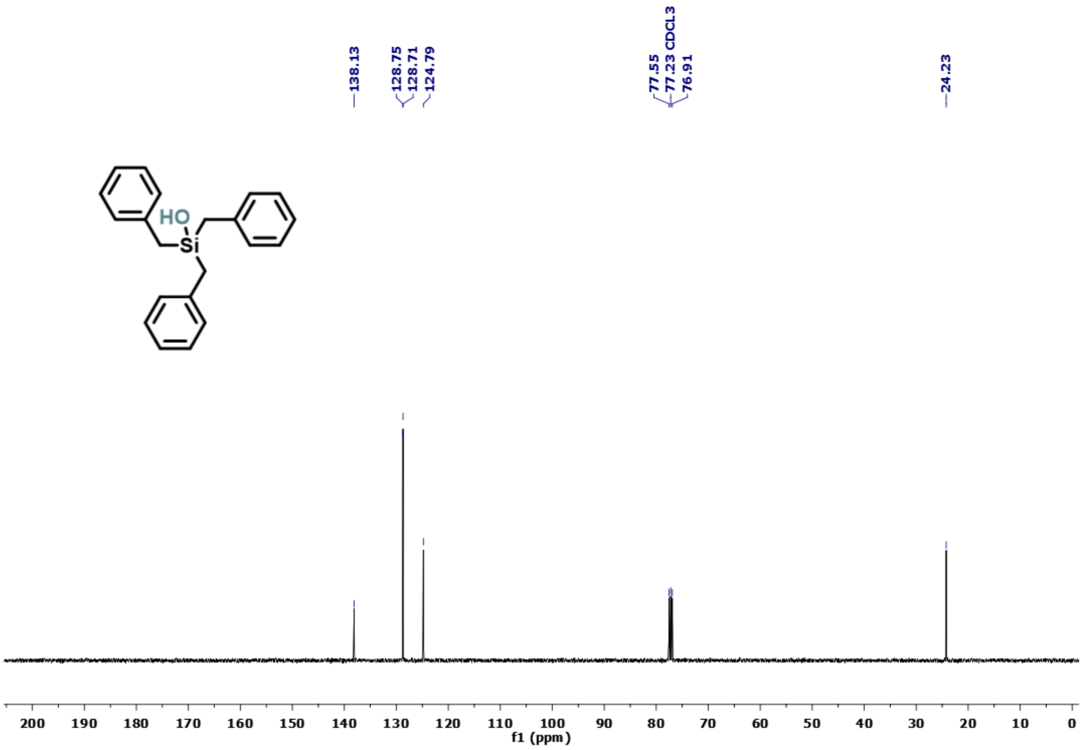

Dimethyl(2-methyl-2-phenylpropyl)silanol (28):

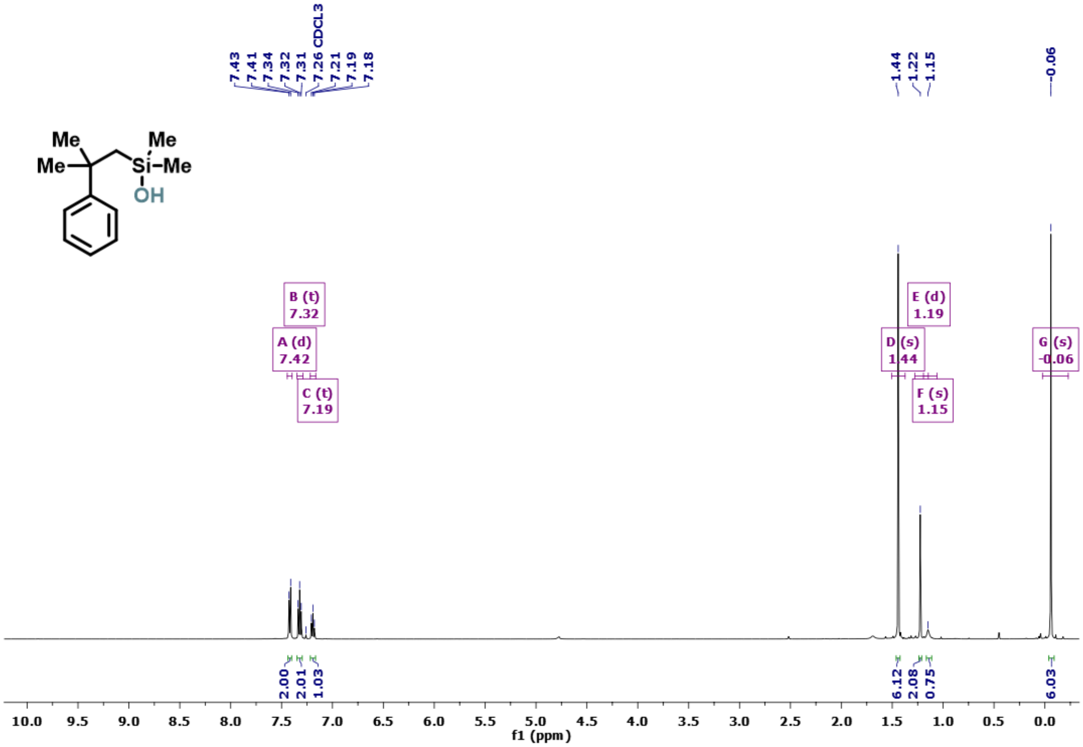

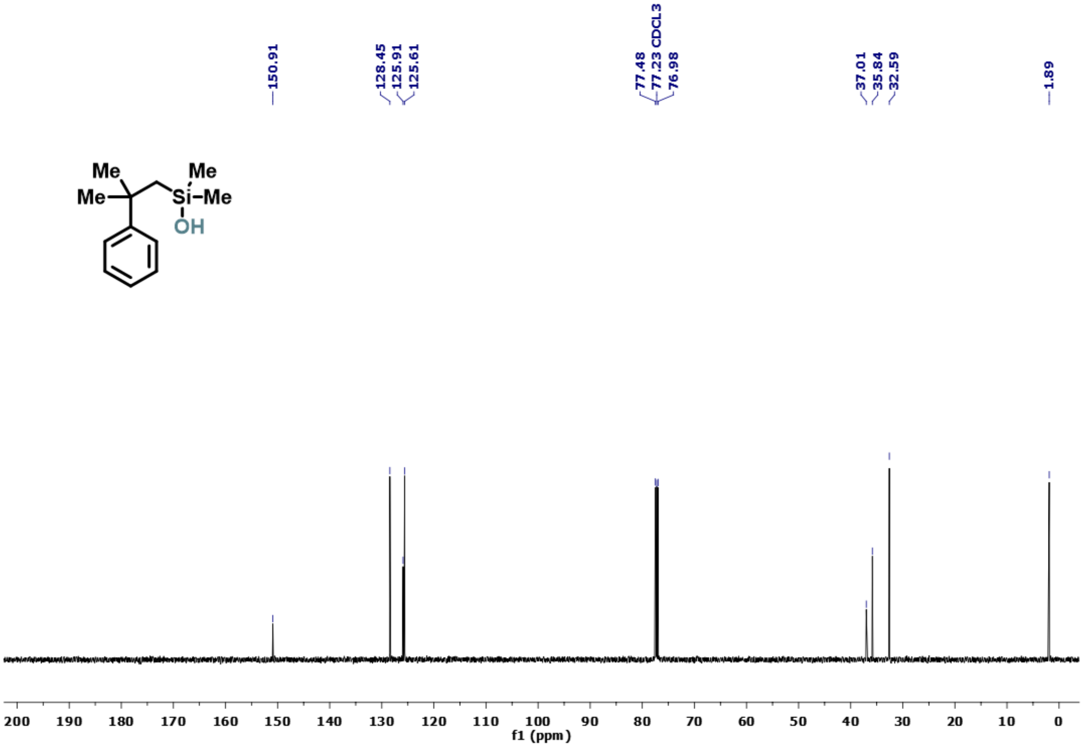

Dimethyl(4-vinylphenyl)silanol (29):

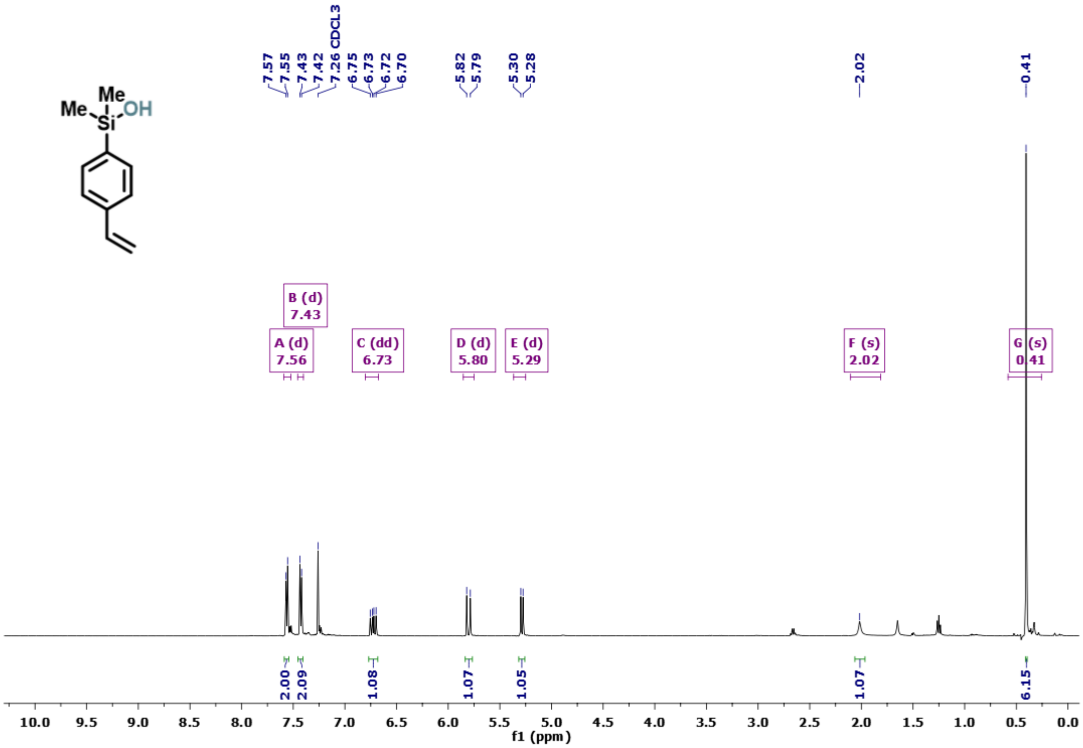

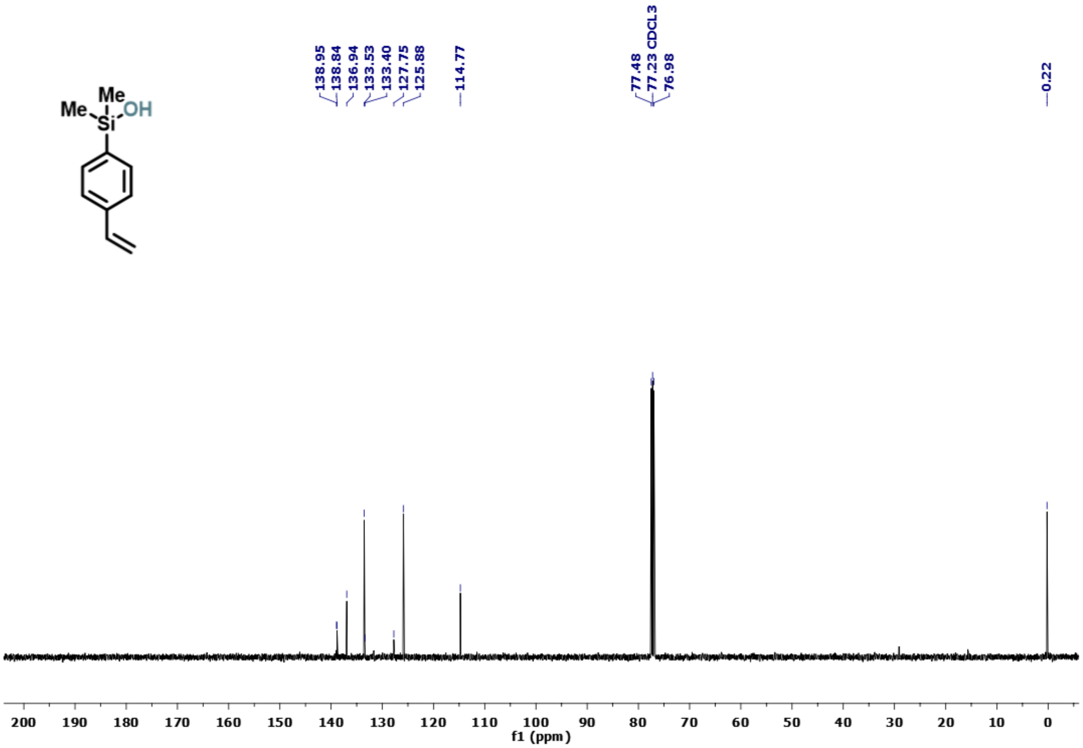

Benzo[b]thiophen-3-yldiisopropylsilanol (32):

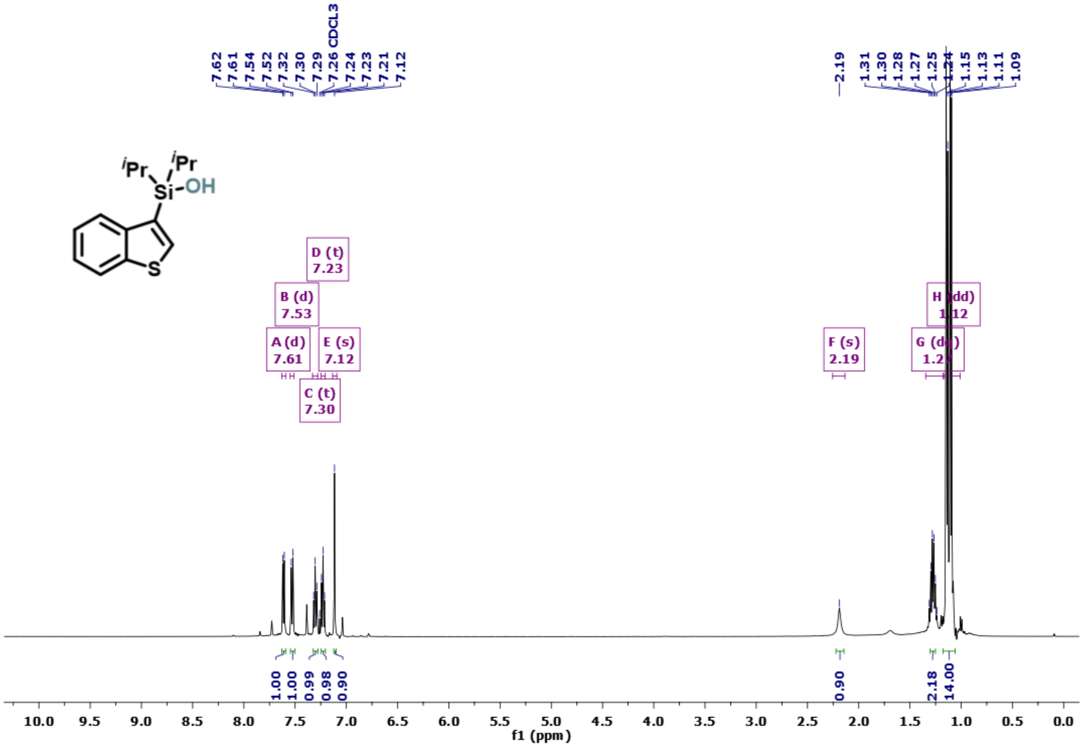

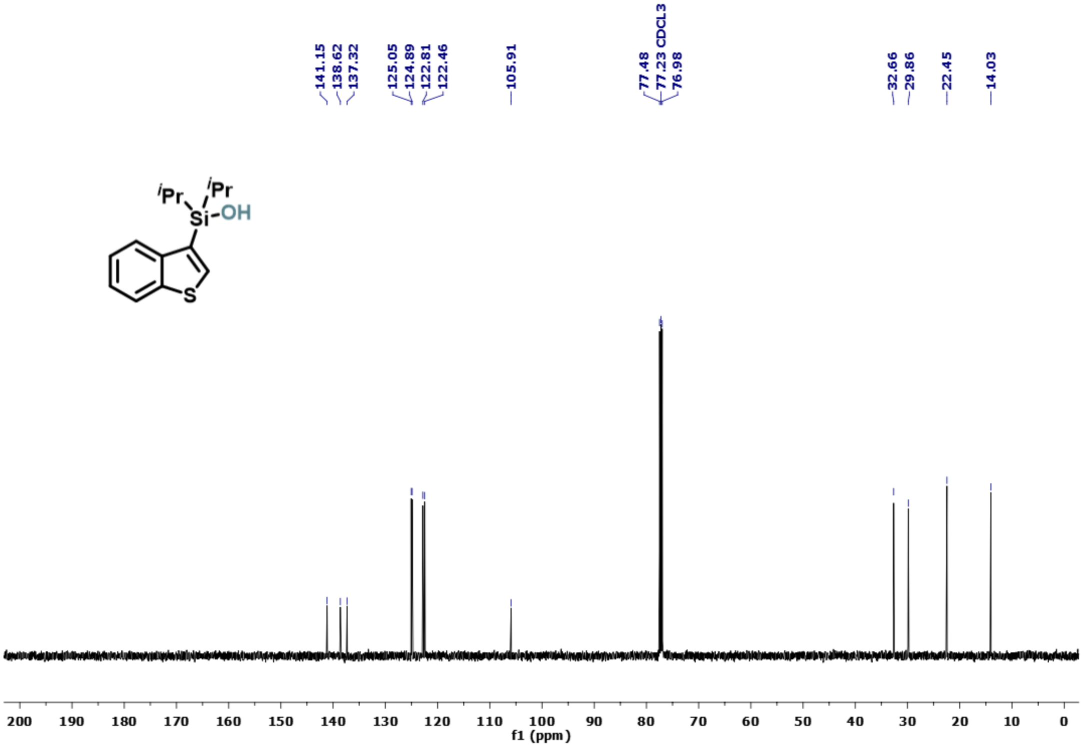

Benzofuran-5-yldiisopropylsilanol (33):

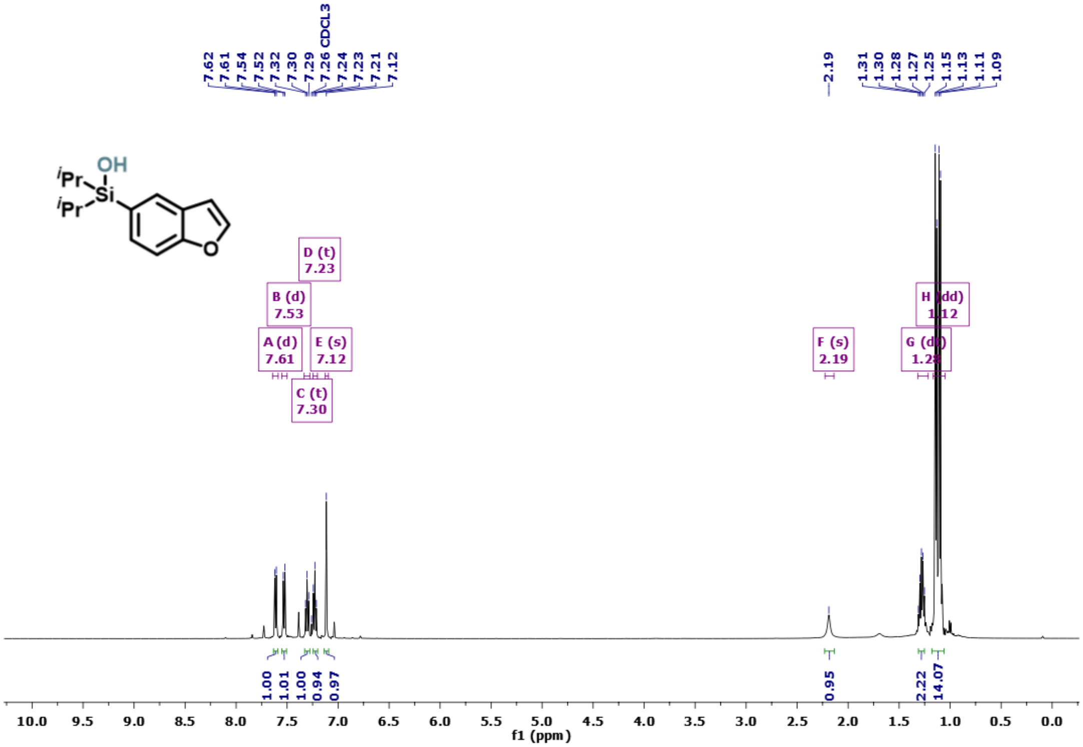

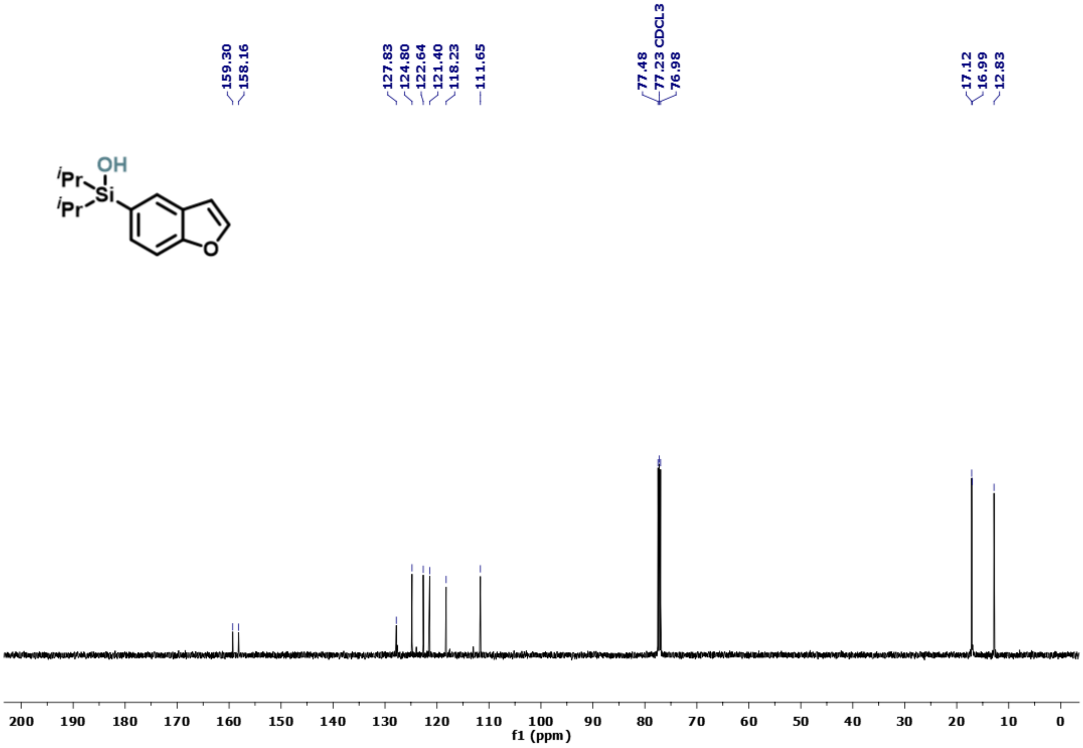

Tert-butyl 4-bromo-2-(hydroxydiisopropylsilyl)-1H-indole-1-carboxylate (34):

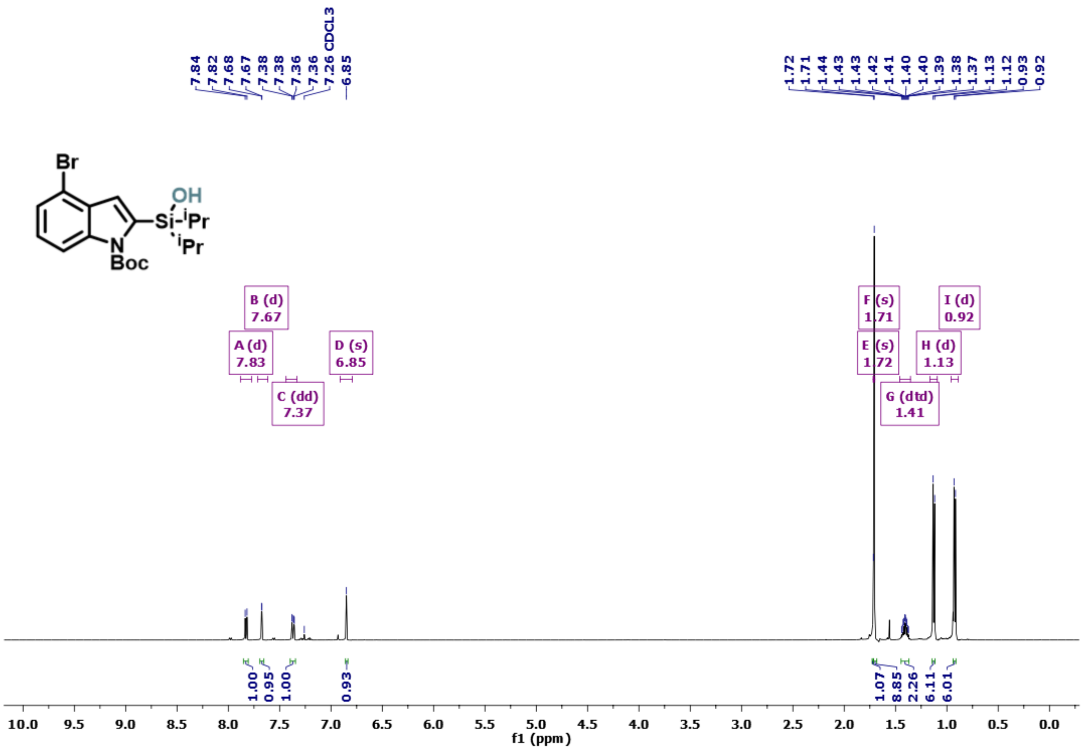

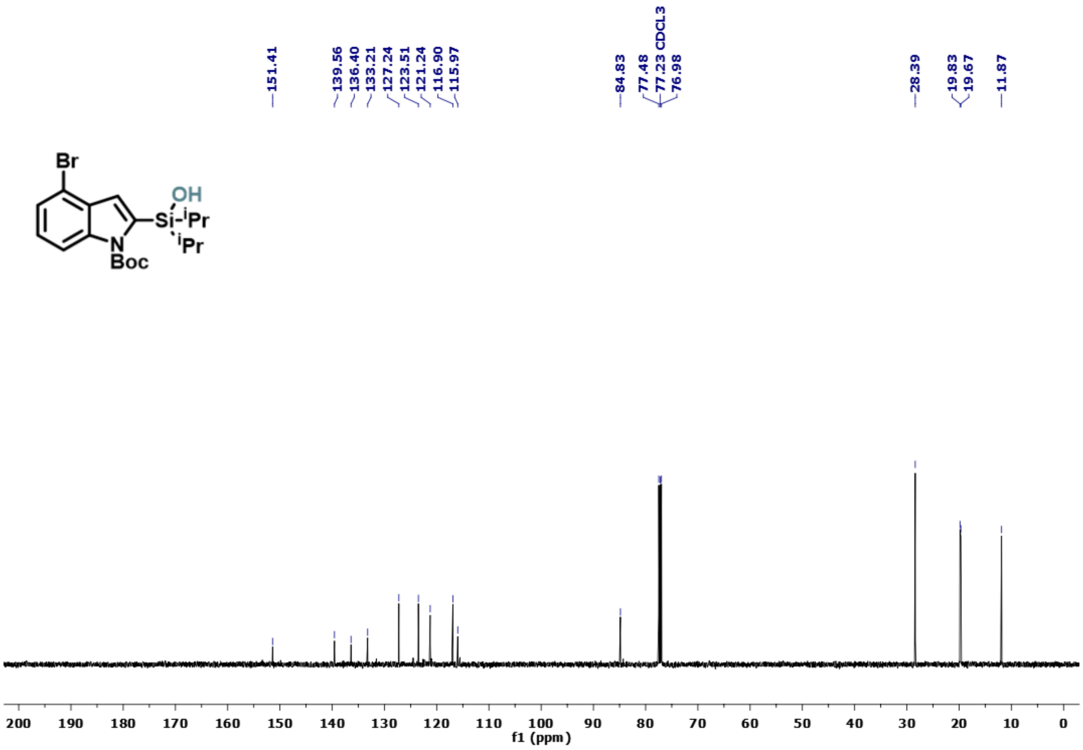

1,1,1,3,3,3-Hexamethyl-2-(trimethylsilyl)trisilan-2-ol (35):

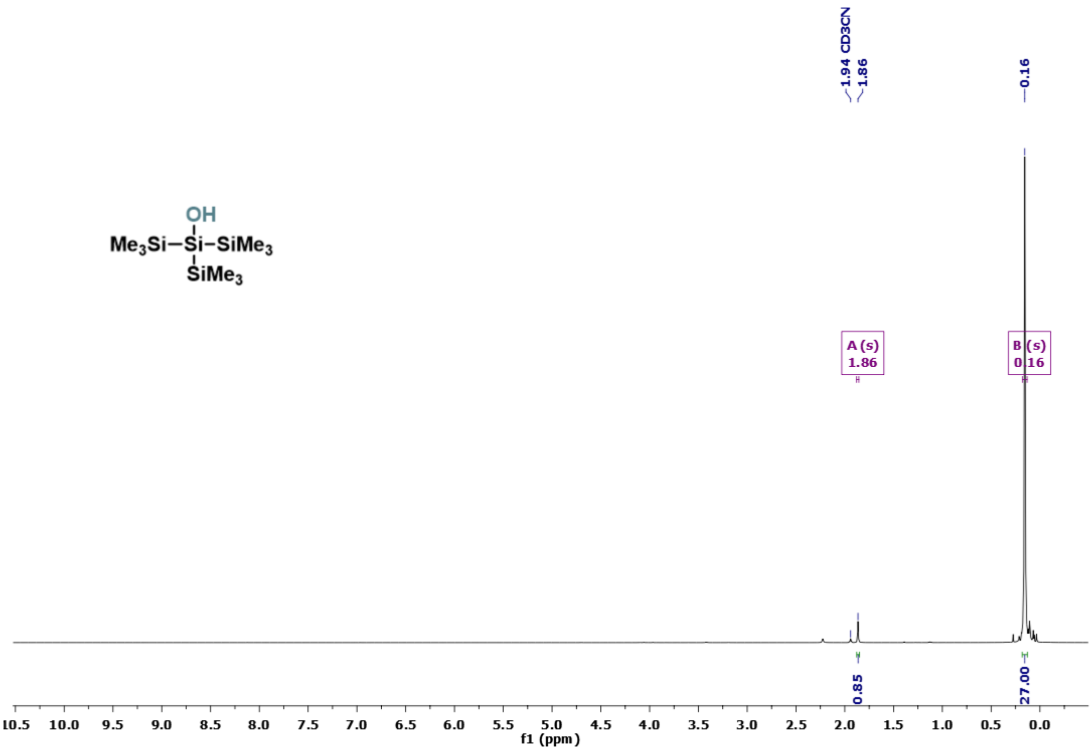

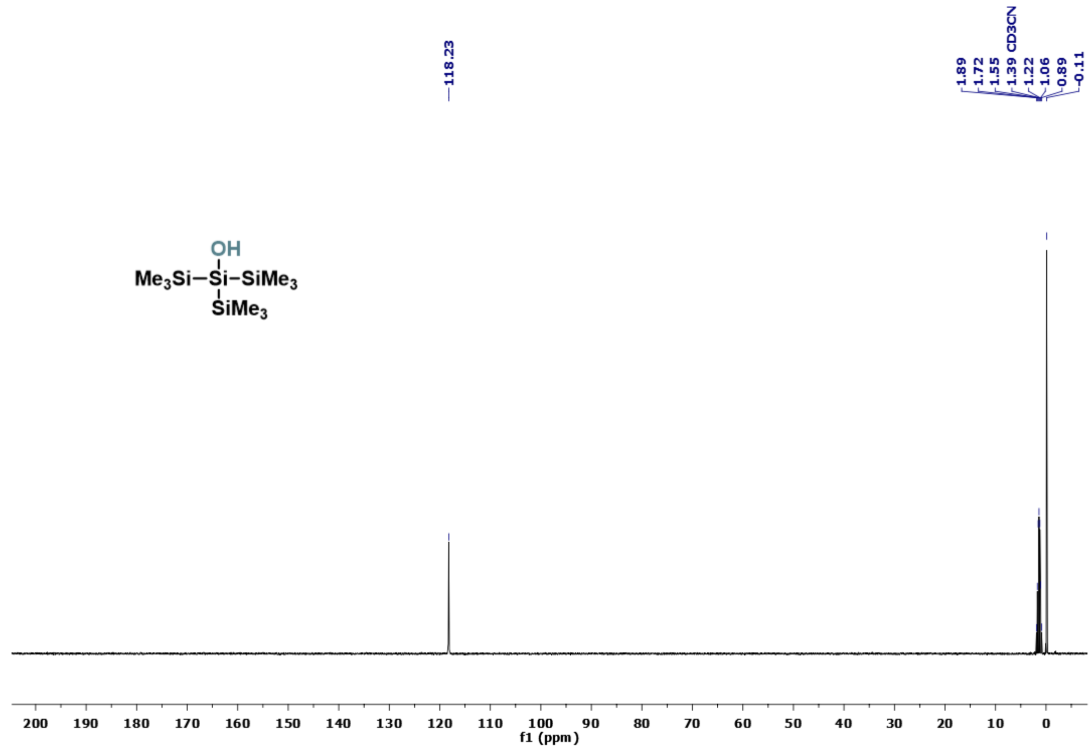

Triisopropylsilanol (36):

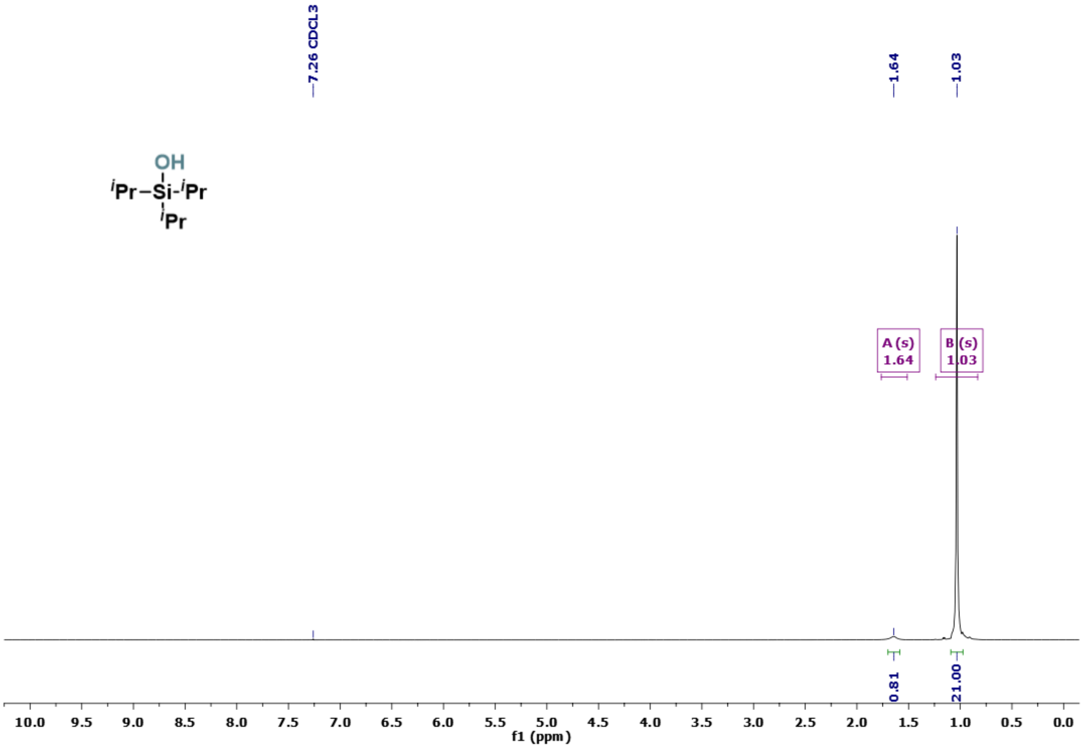

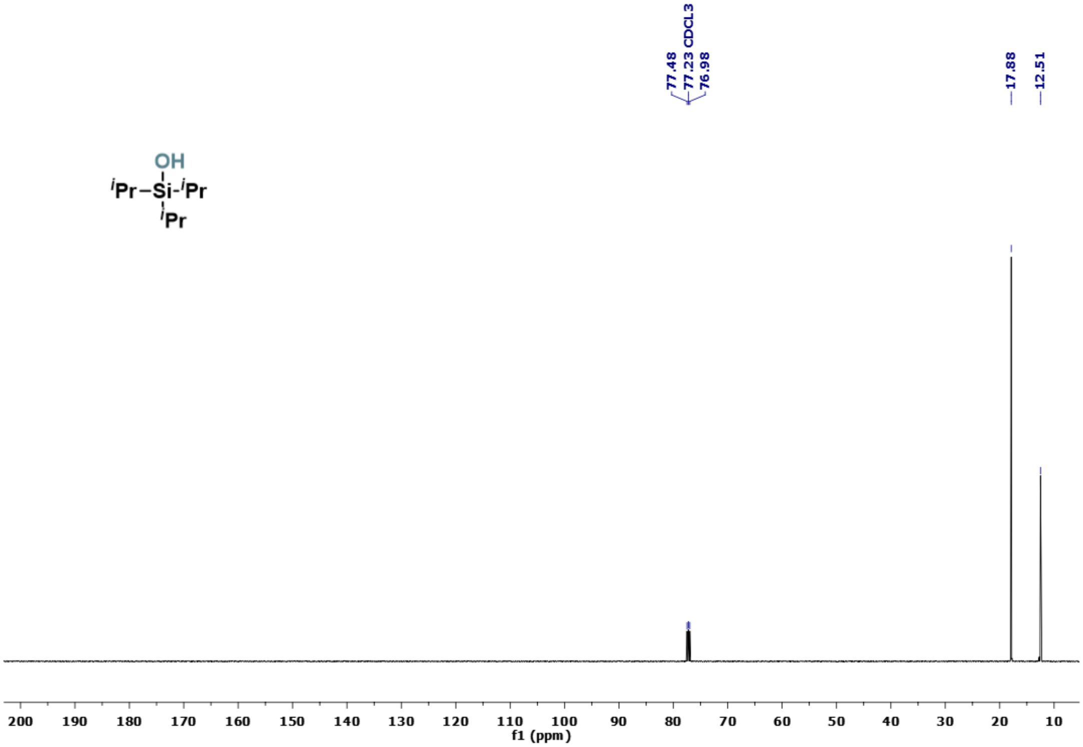

Chlorodiisopropylsilanol (37):

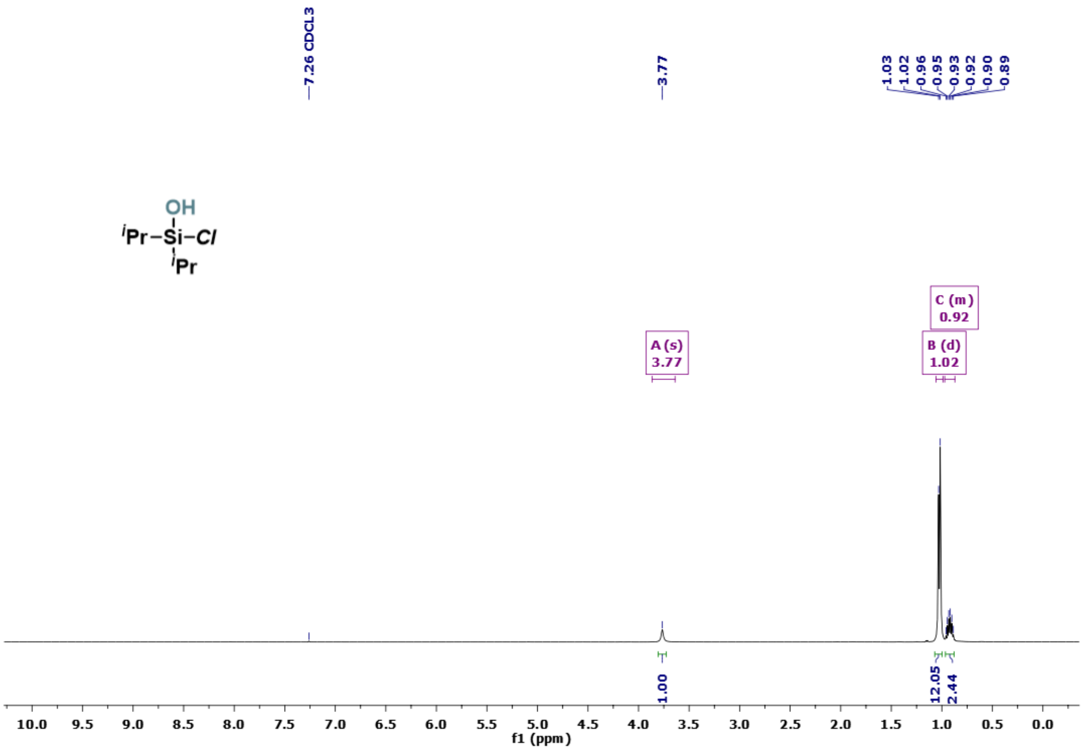

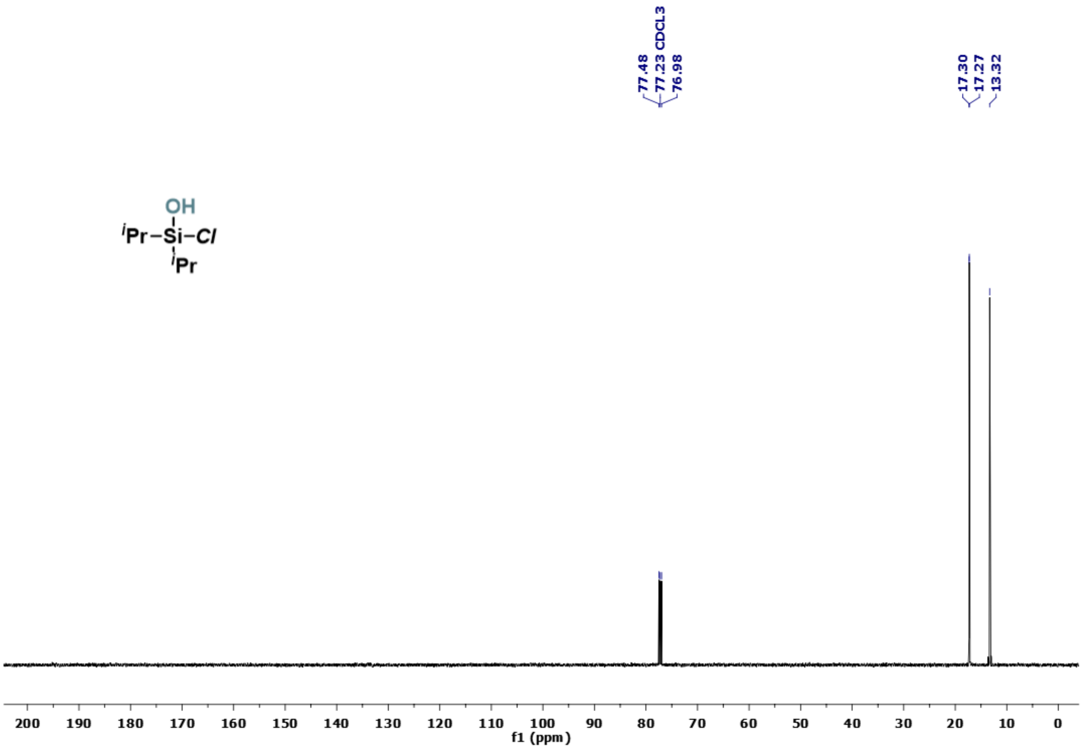

Tripropylsilanol (38):

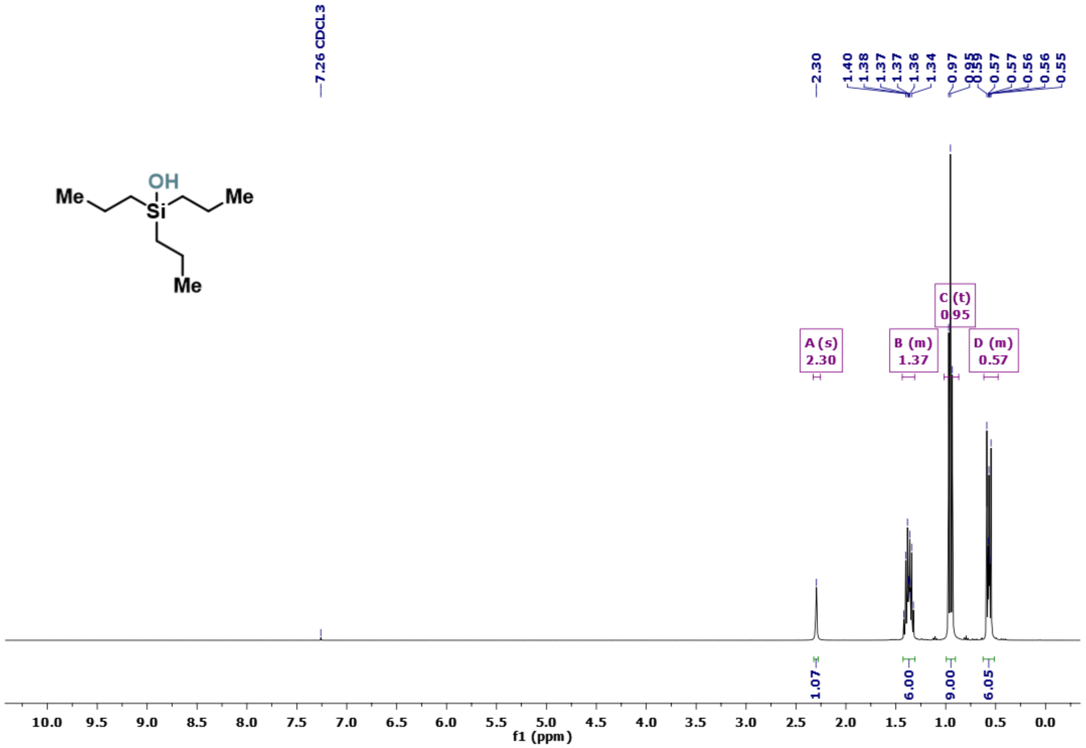

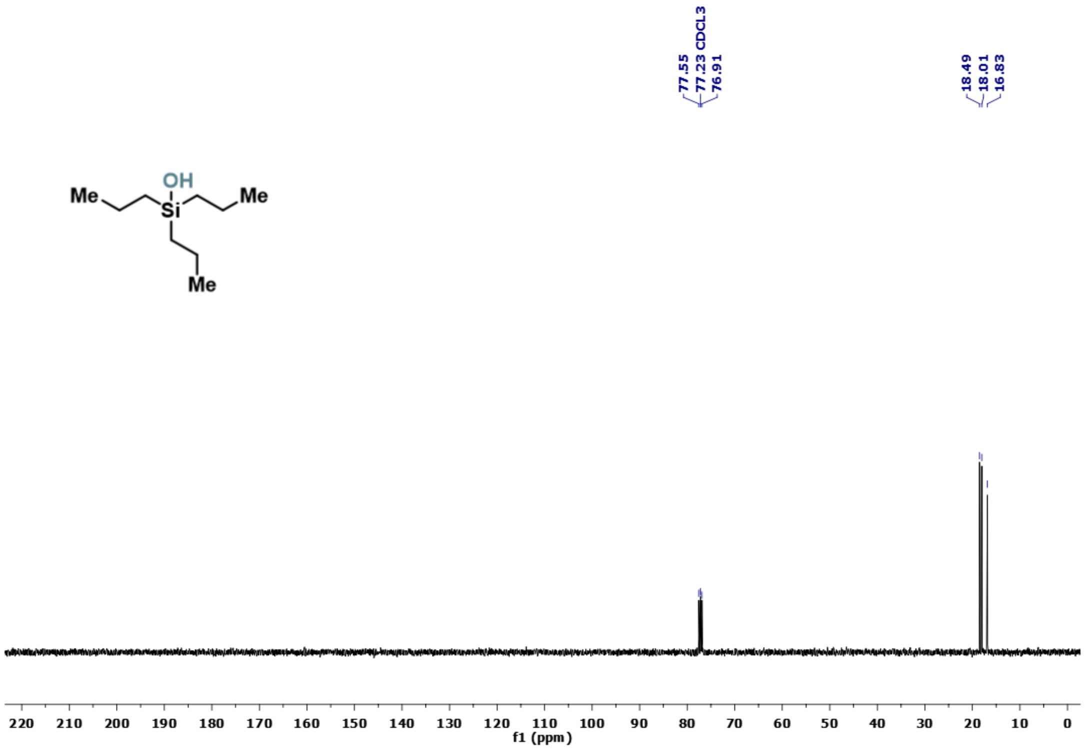

Tributylsilanol (39):

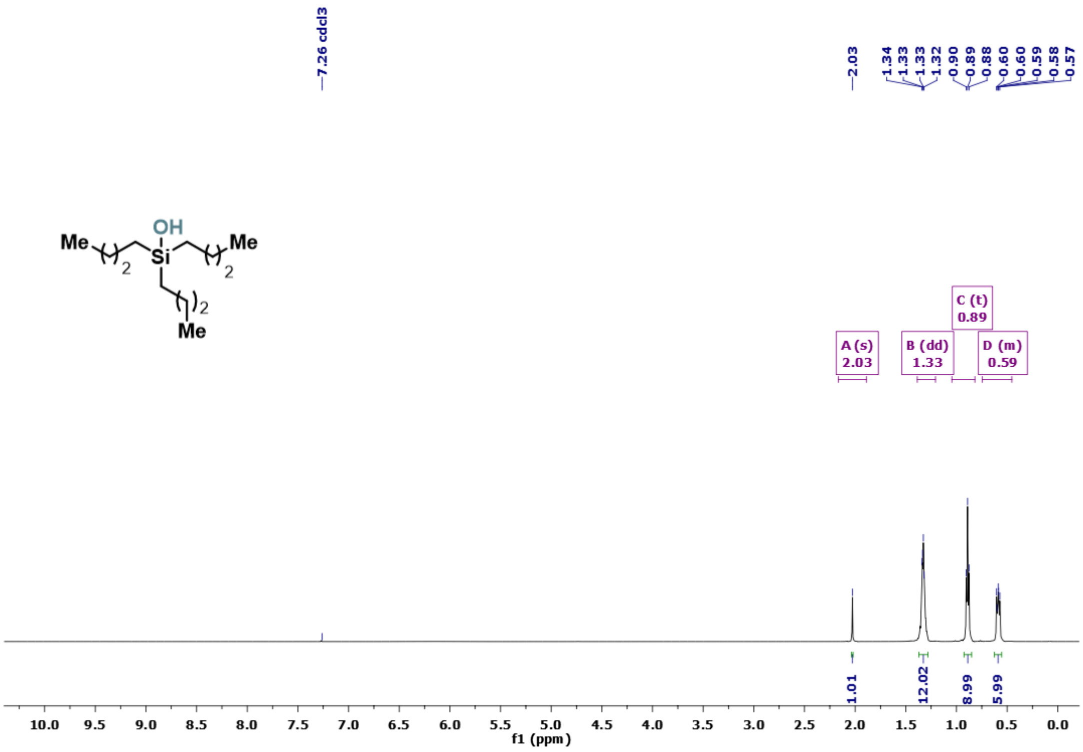

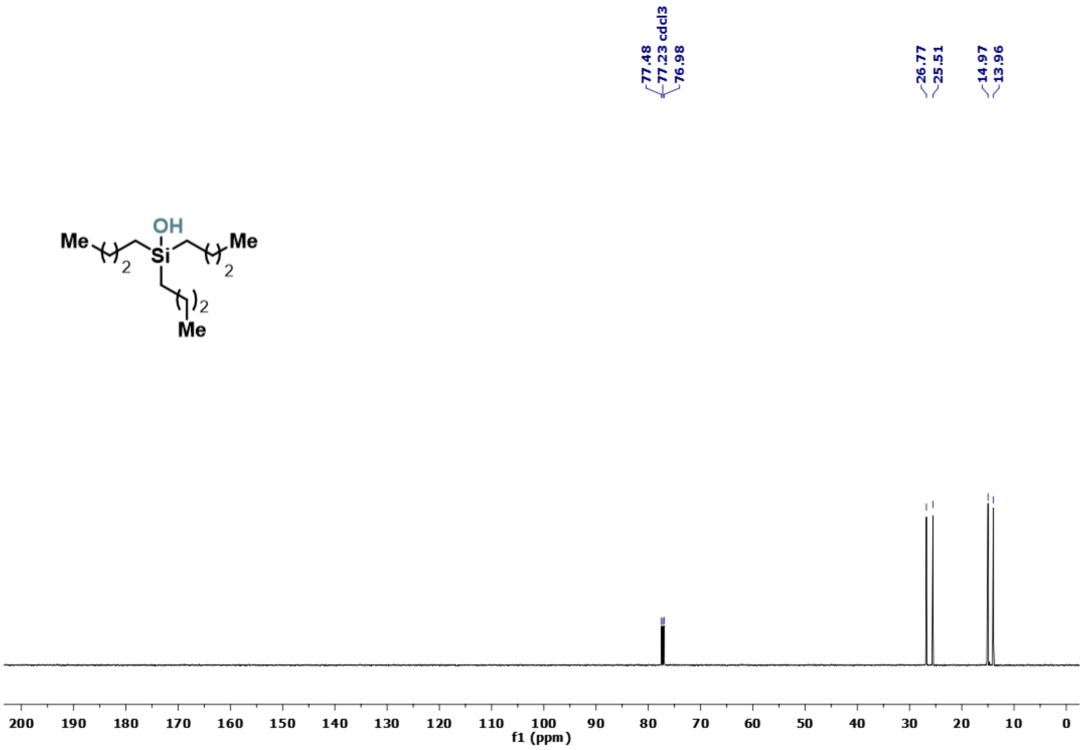

Trihexylsilanol (40):

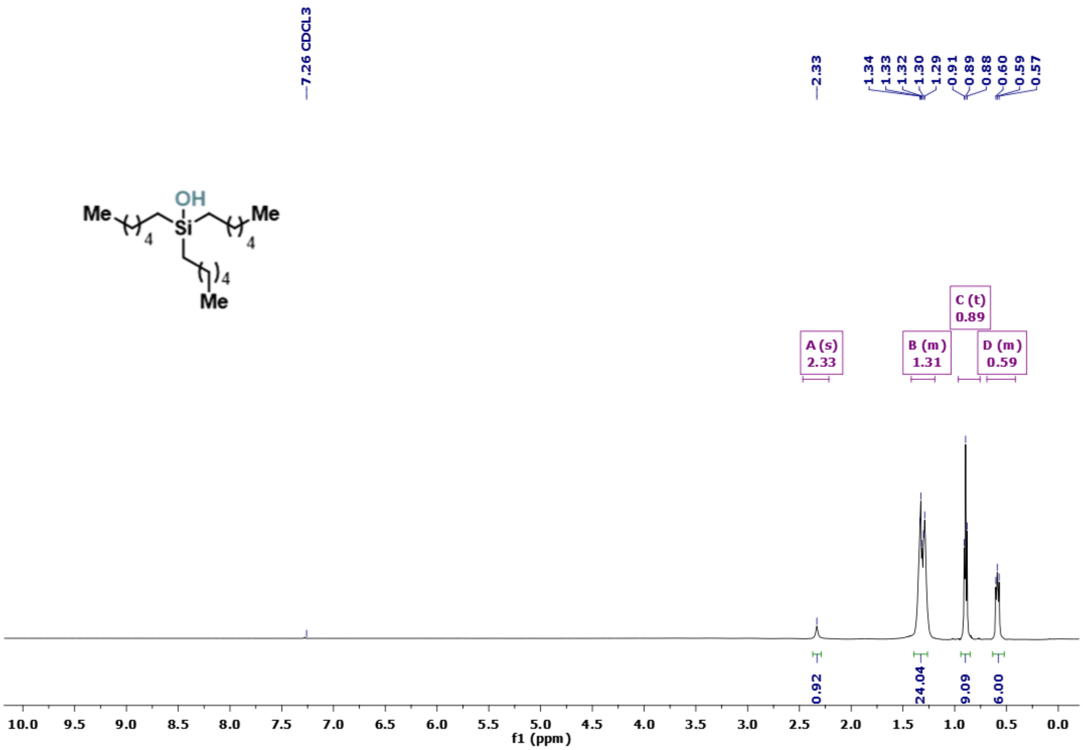

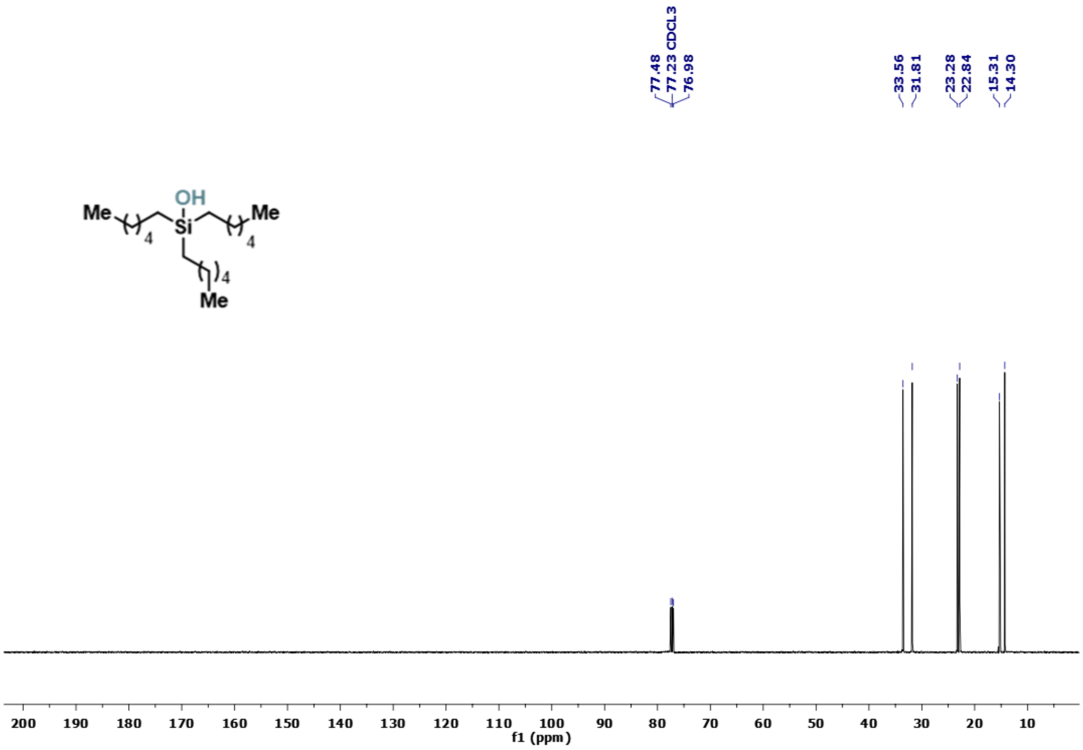

1,1,3,3,5,5,5-heptamethyltrisiloxan-1-ol (41):

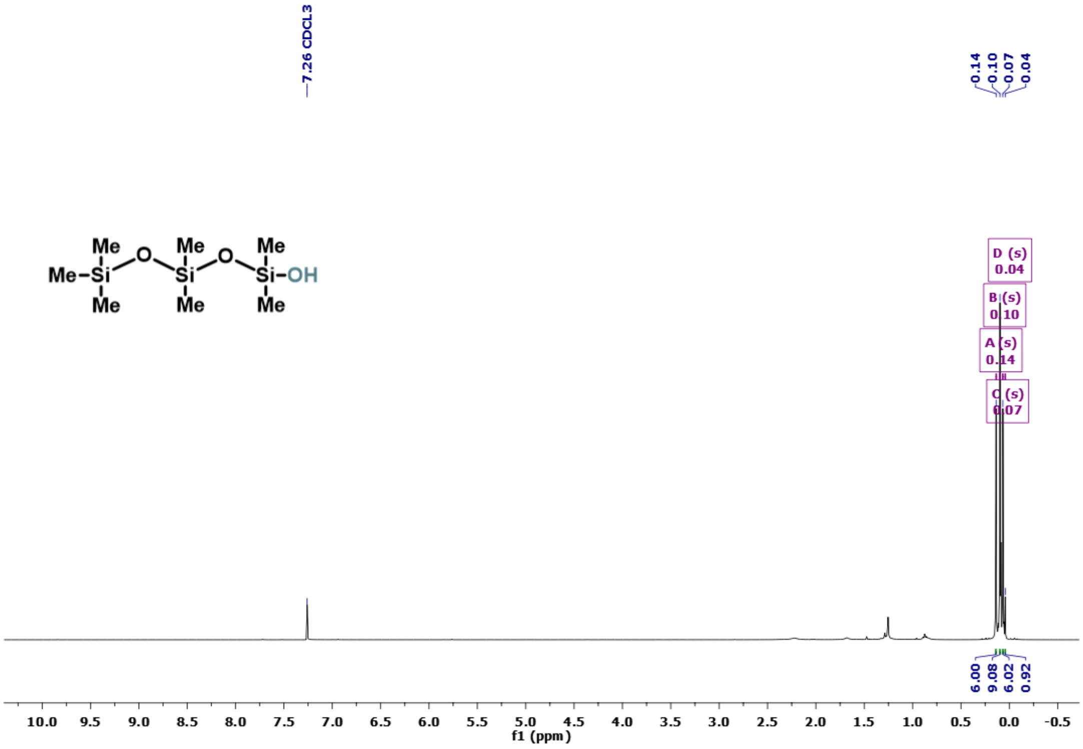

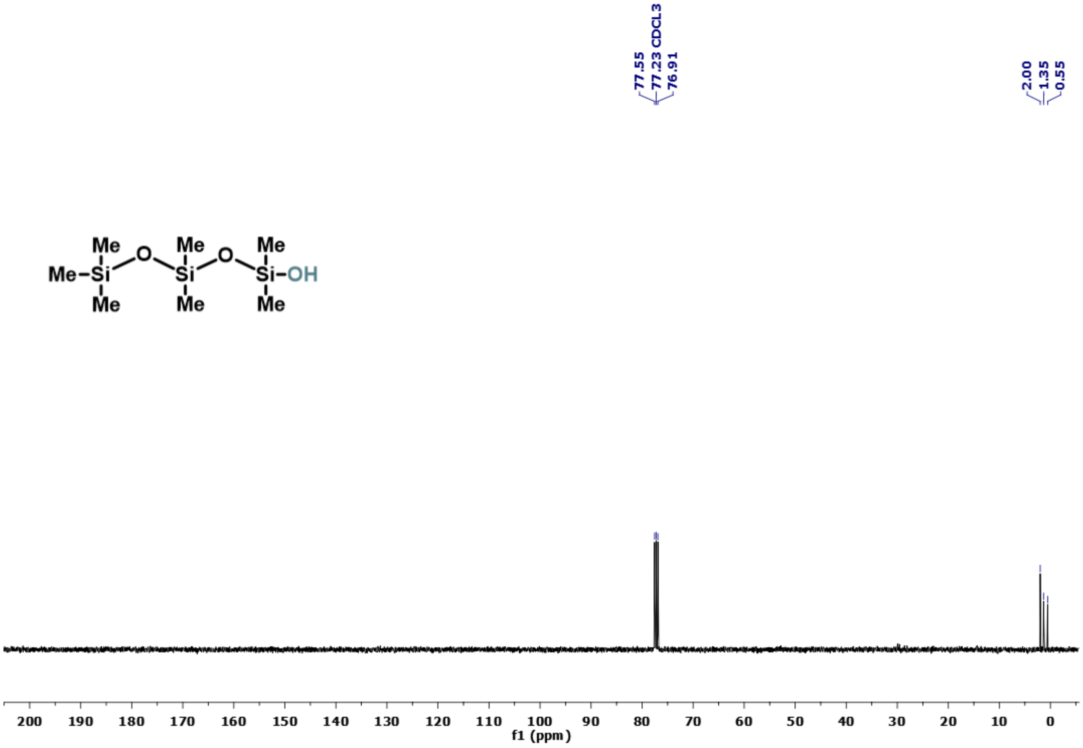

Di-tert-butylsilanediol (42):

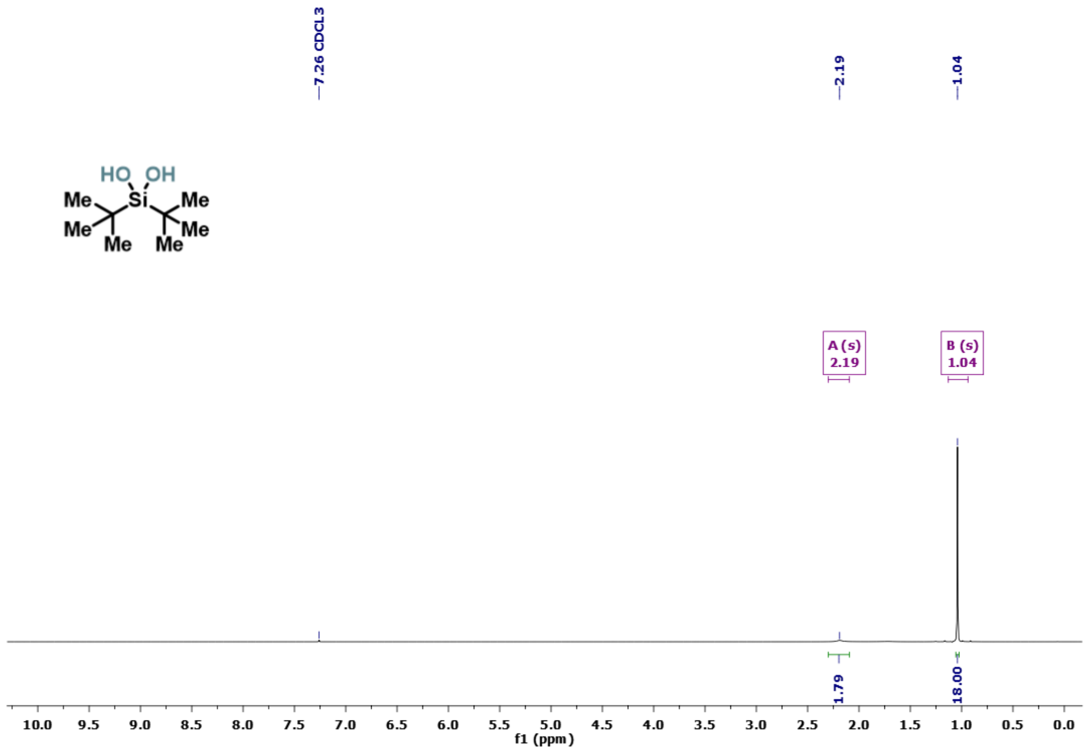

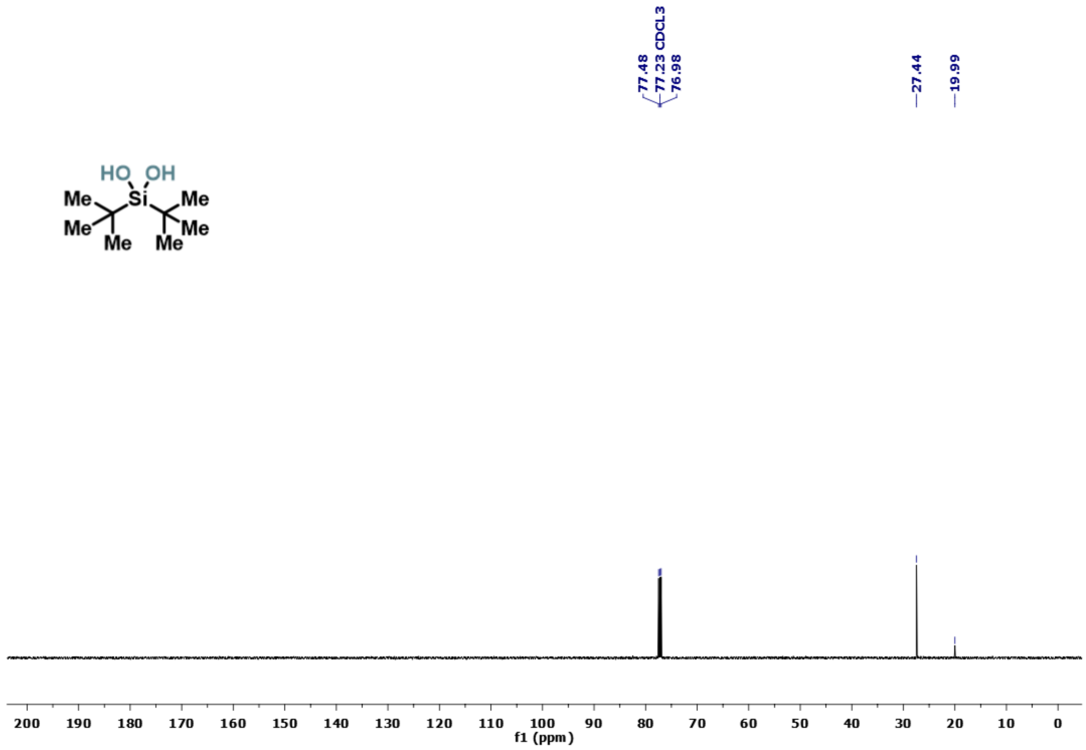

Diphenylsilanediol (43):

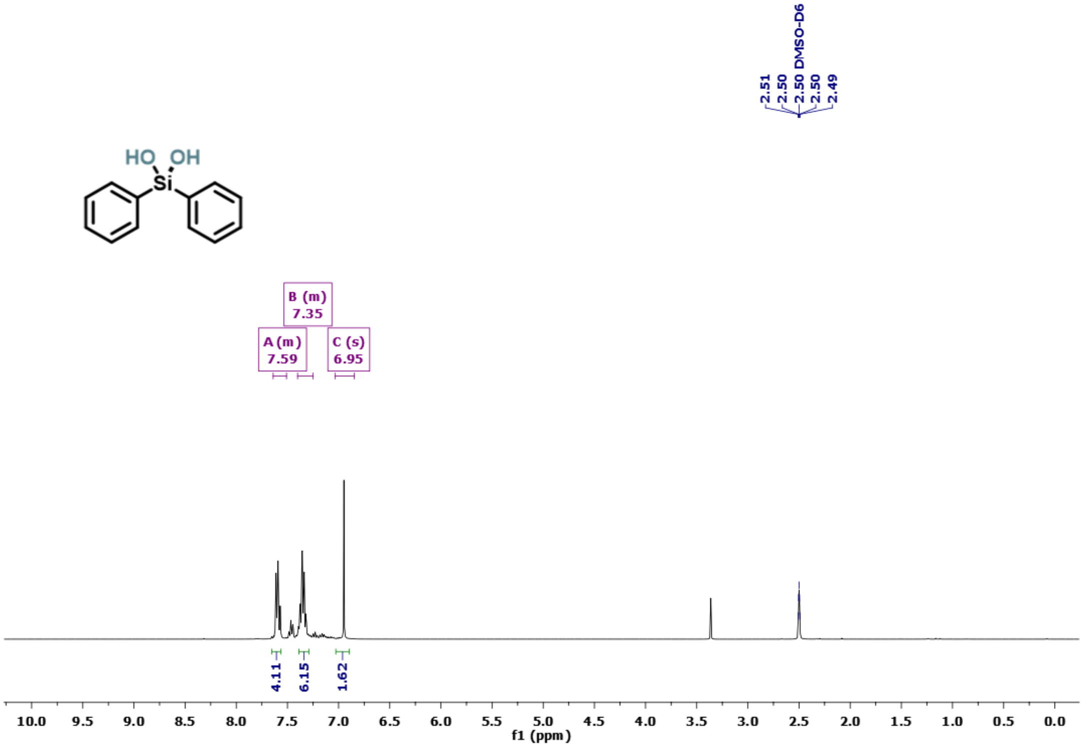

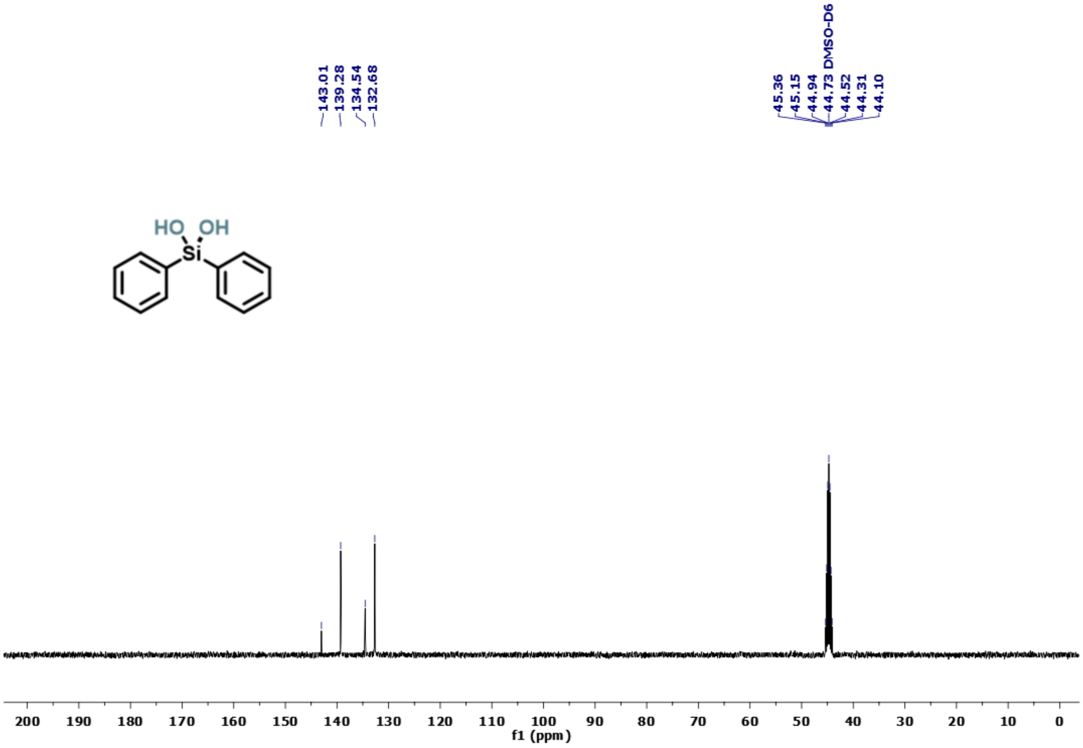

1,4-Phenylenebis(dimethylsilanol) (44):

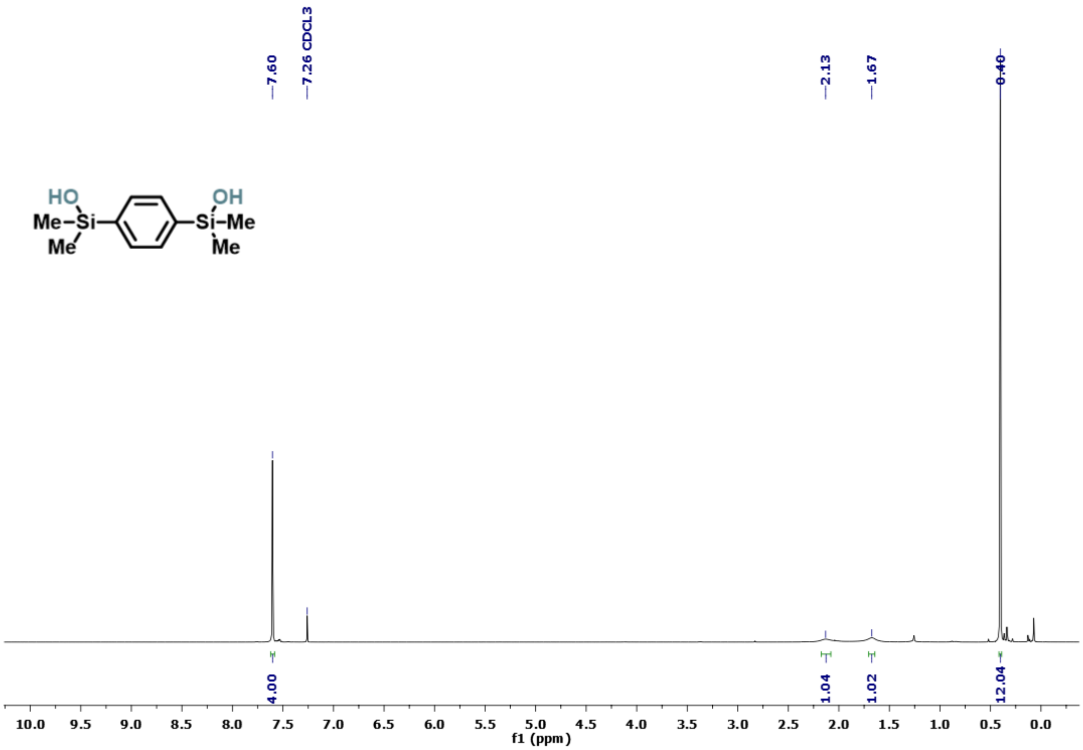

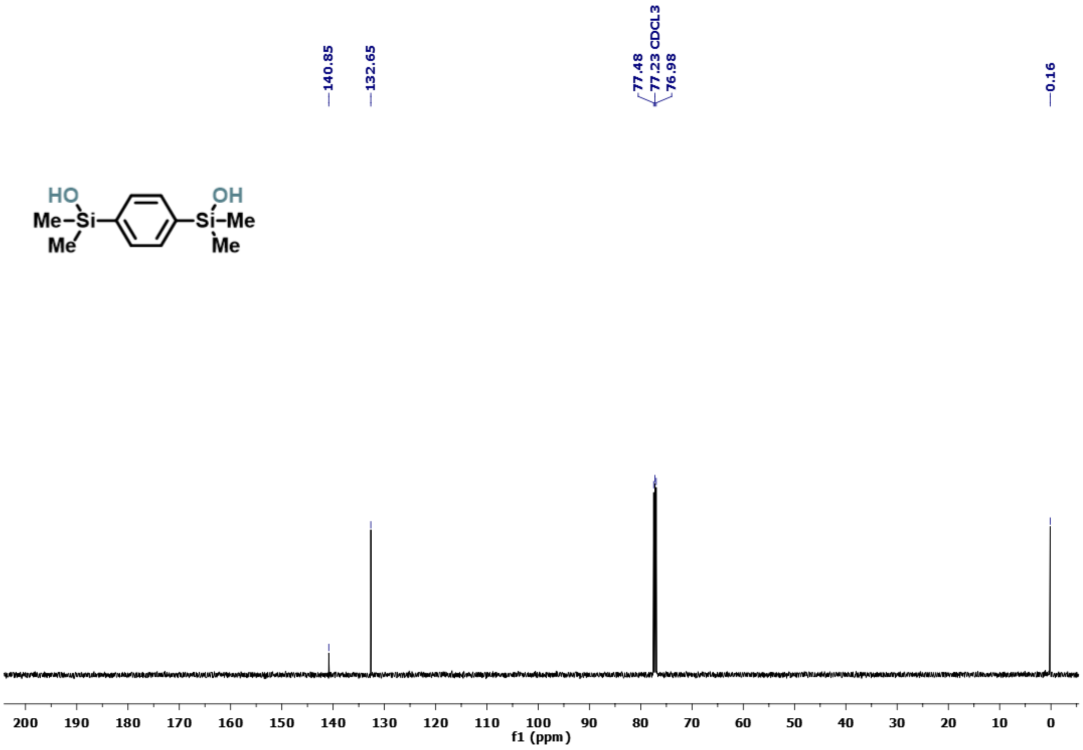

4'-((Hydroxydimethylsilyl)methyl)-[1,1'-biphenyl]-2-carbonitrile (45):

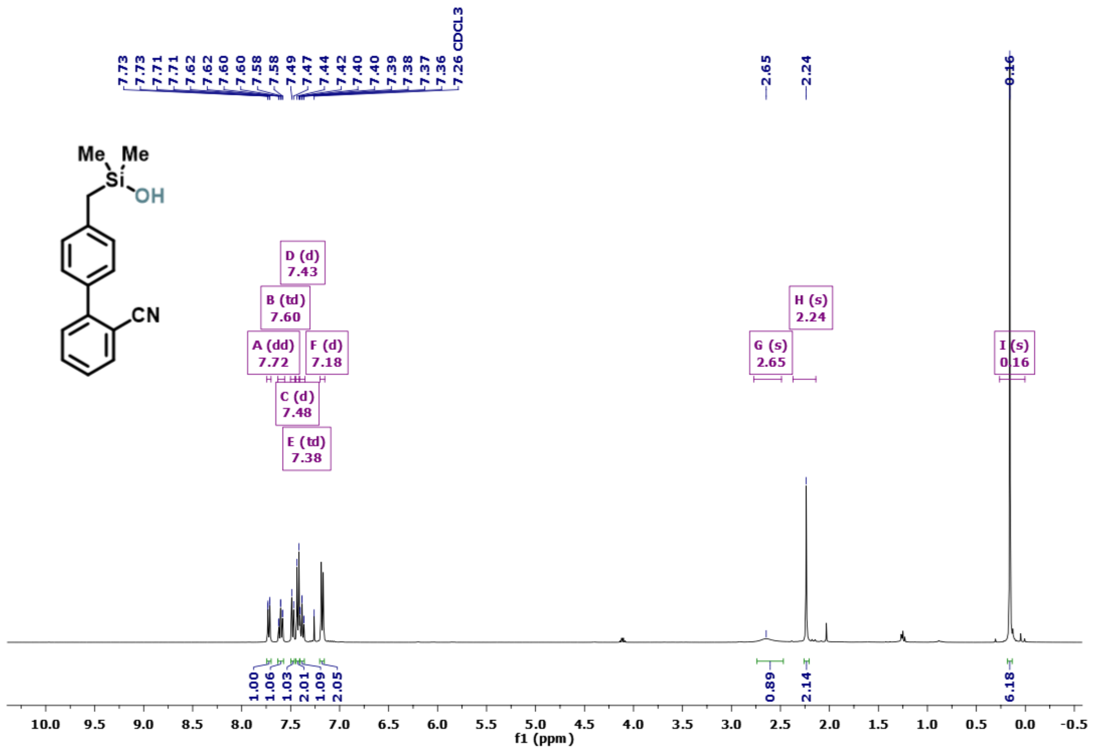

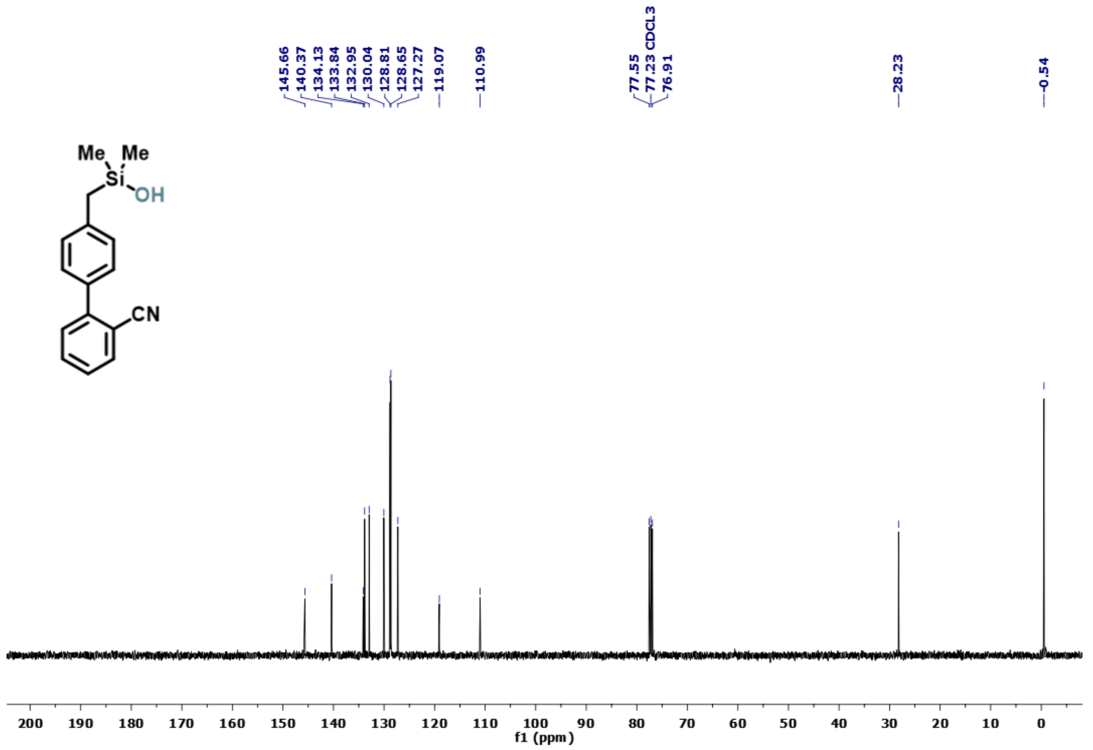

4-(Hydroxydimethylsilyl)phenyl 2-(4-((2-oxocyclopentyl)methyl)phenyl)propanoate (47):

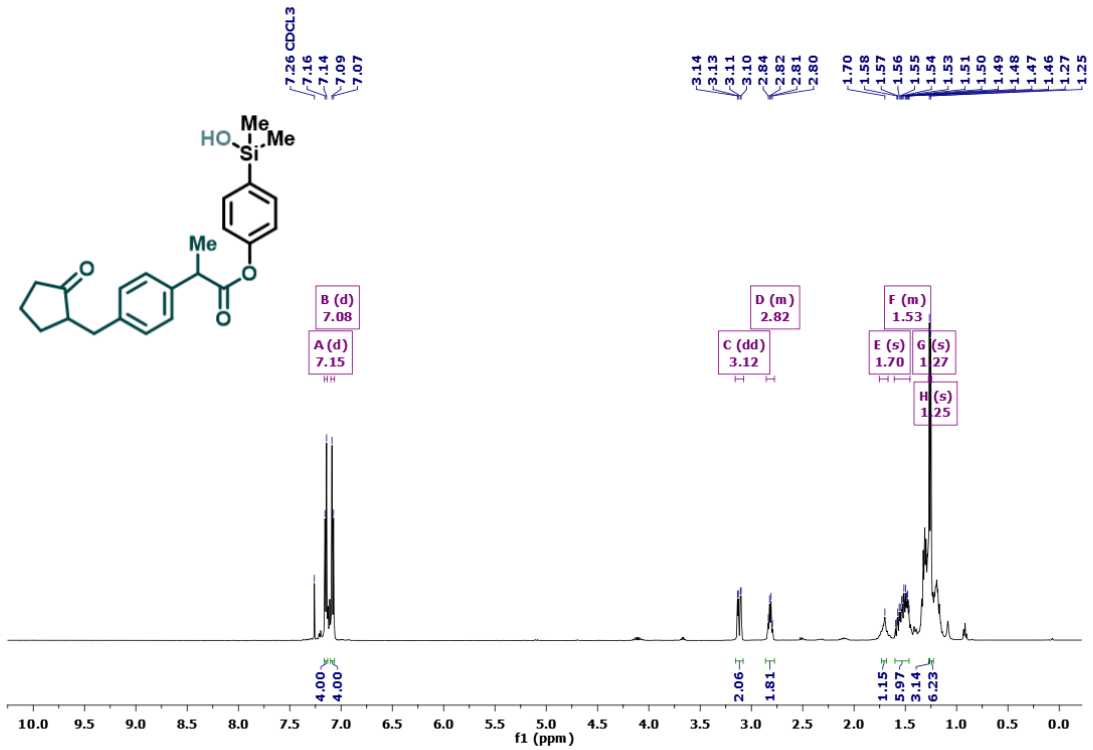

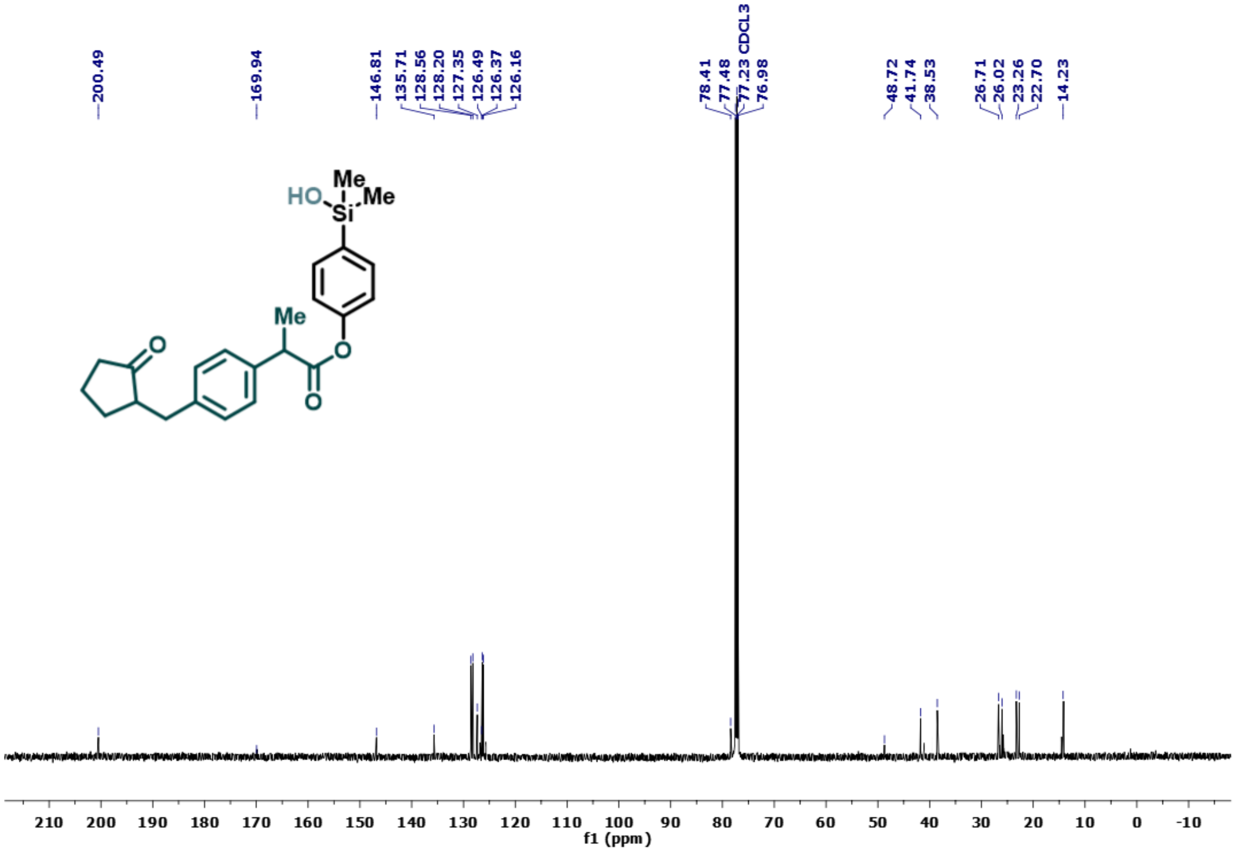

Methyl 2-(4-((hydroxydimethylsilyl)methyl)phenyl)propanoate (49):

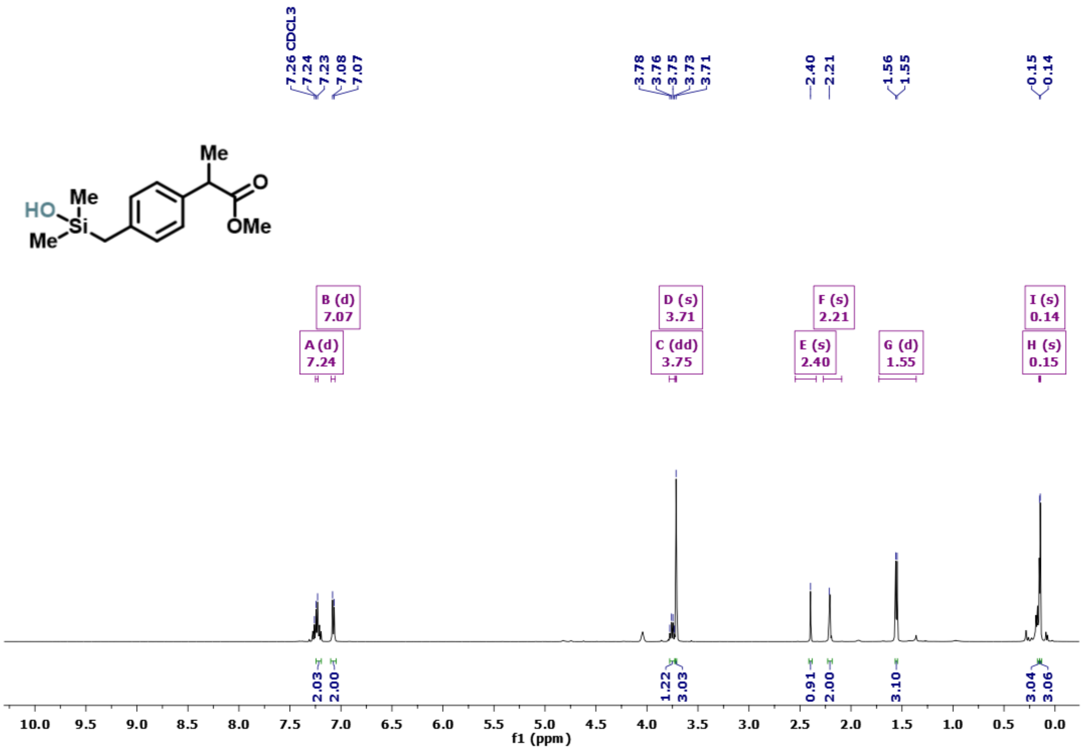

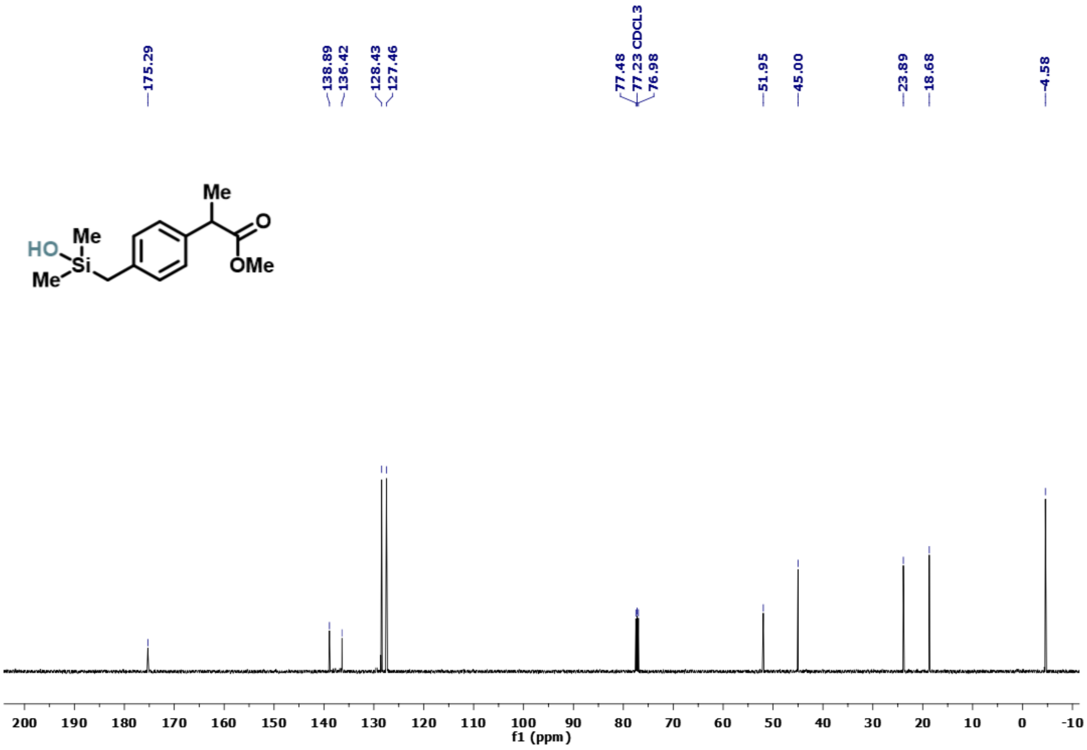

Diisopropyl(phenyl)silanol (51):

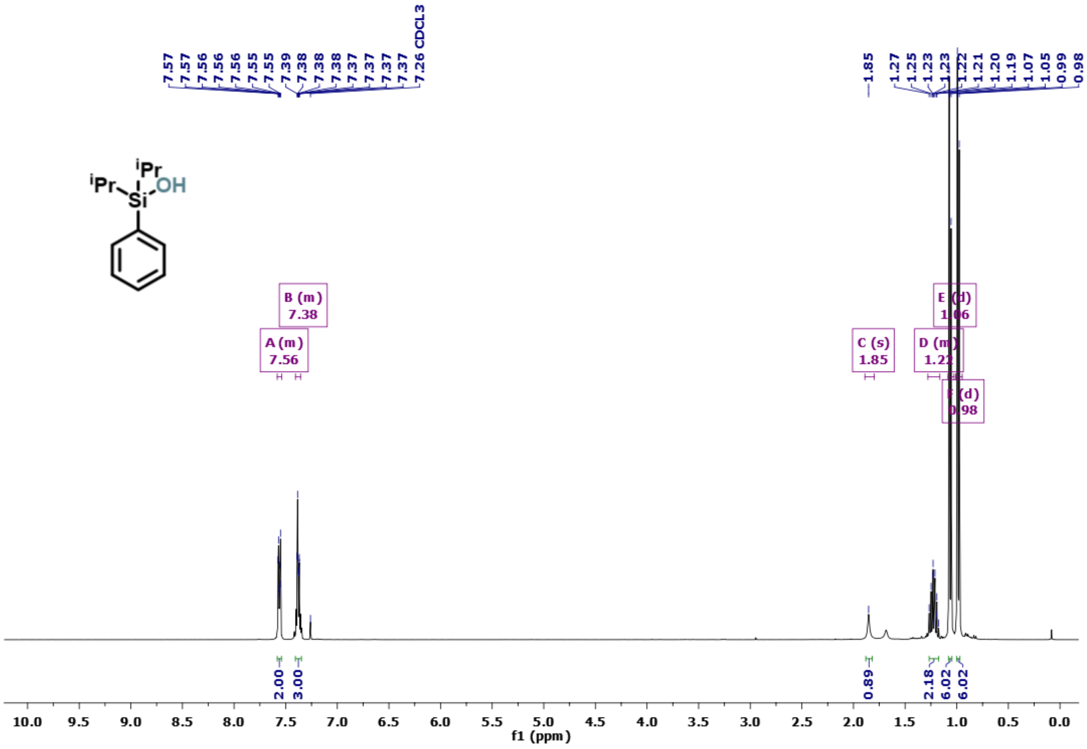

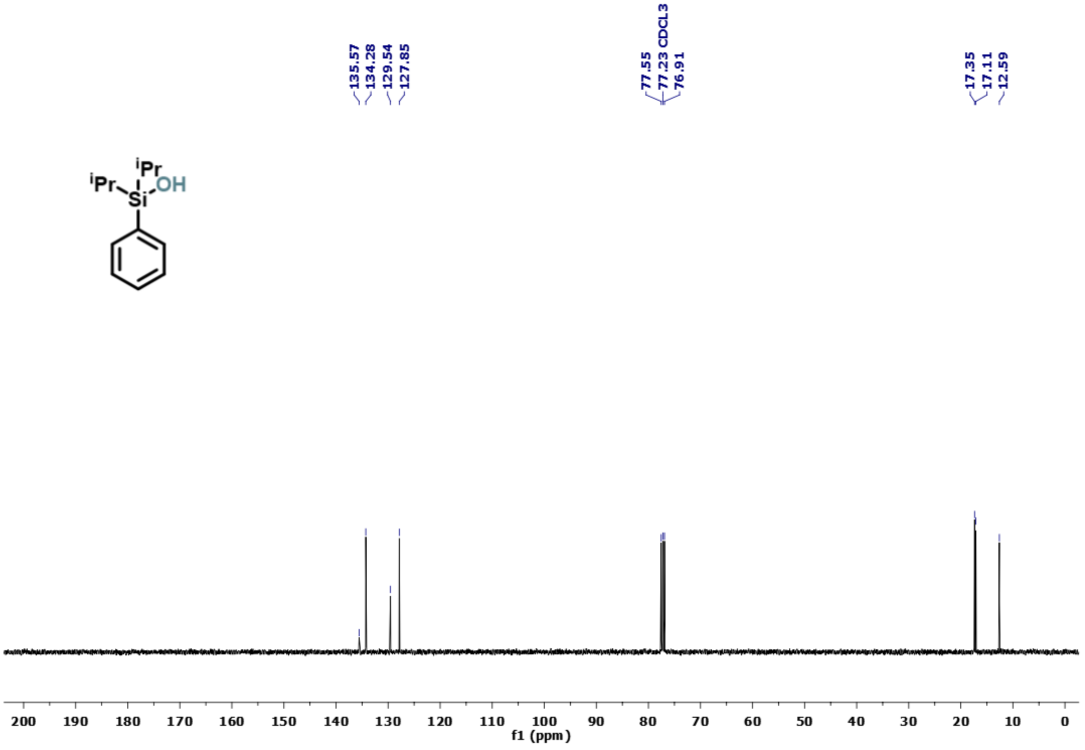

Diisopropyl(4-methoxyphenyl)silanol (52):

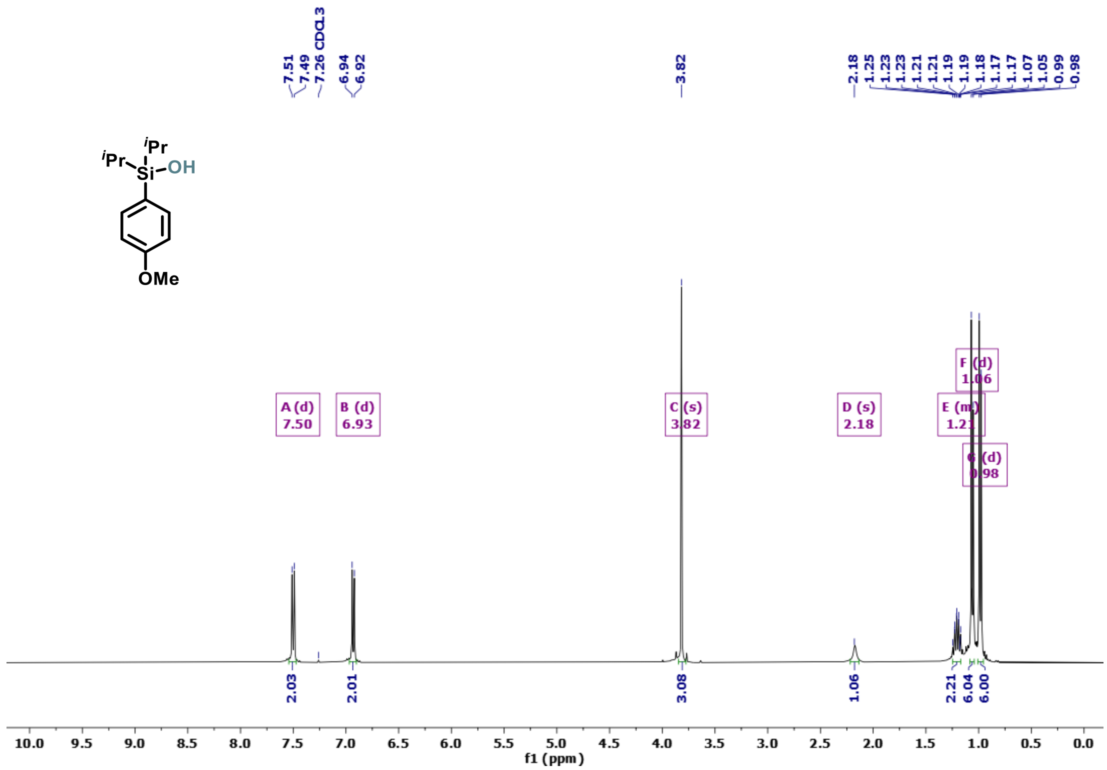

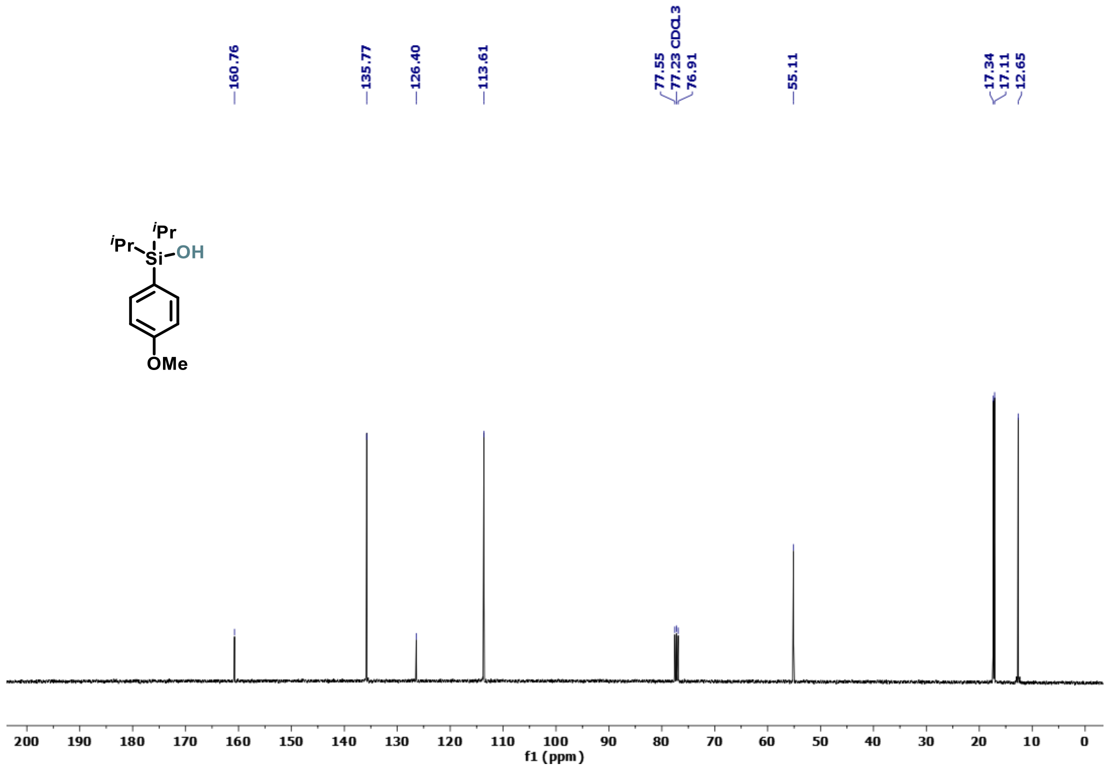

Tert-butyldiphenylsilanol (53):

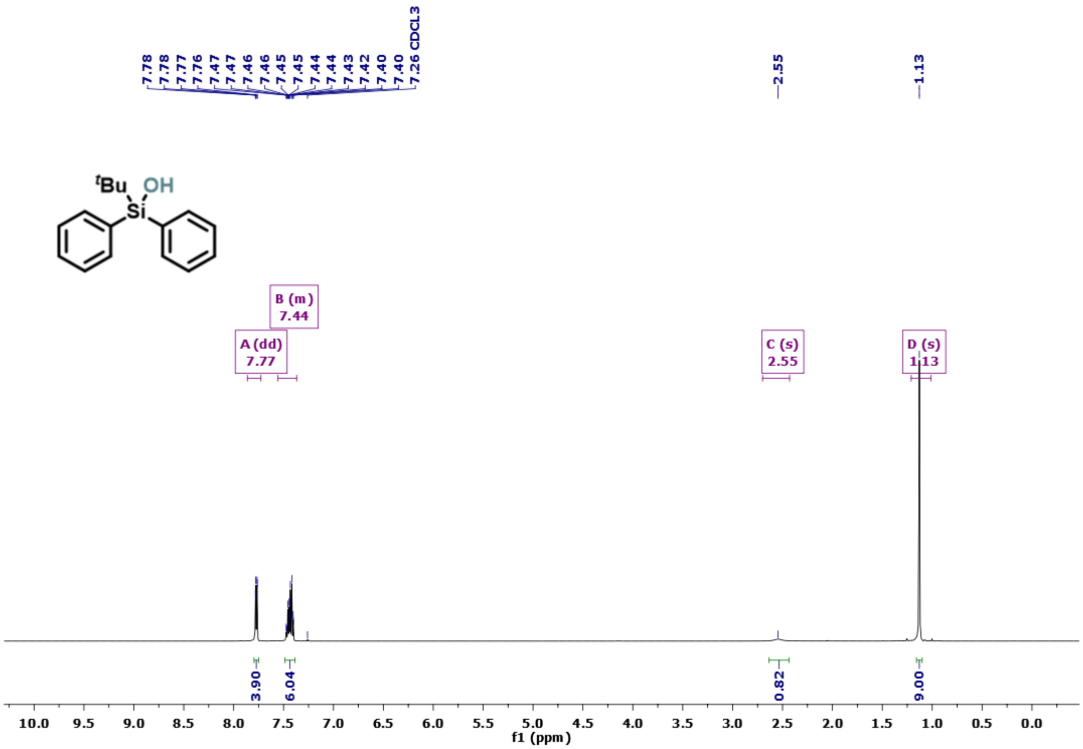

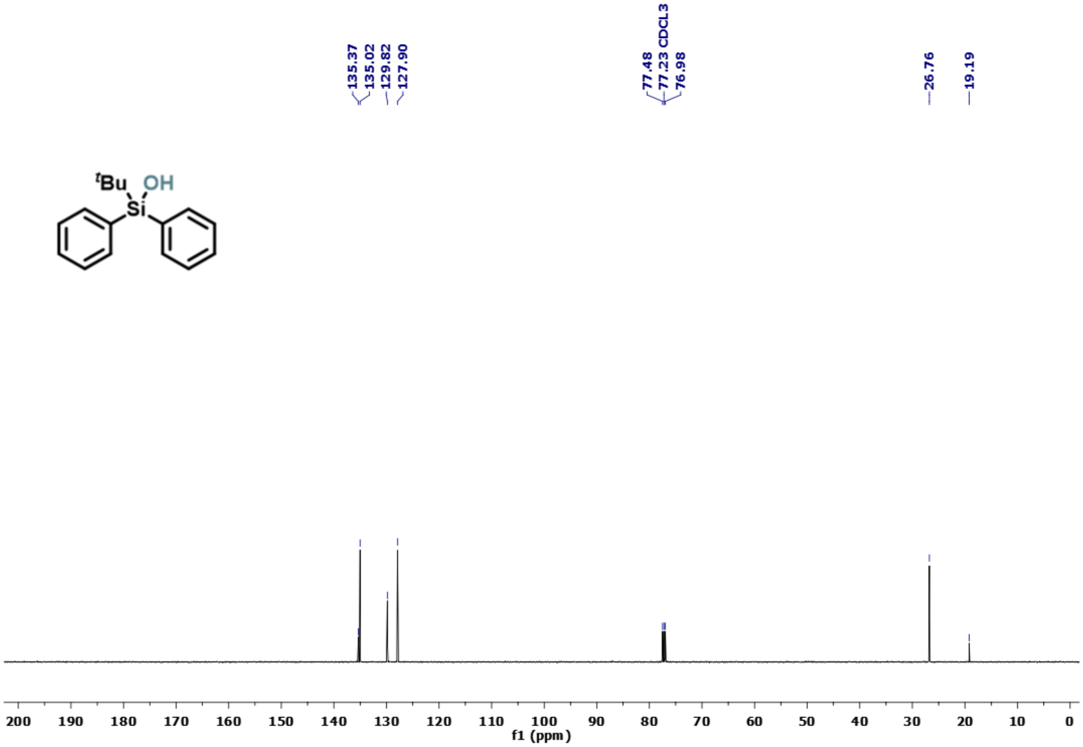

Benzyldimethylsilanol (54):

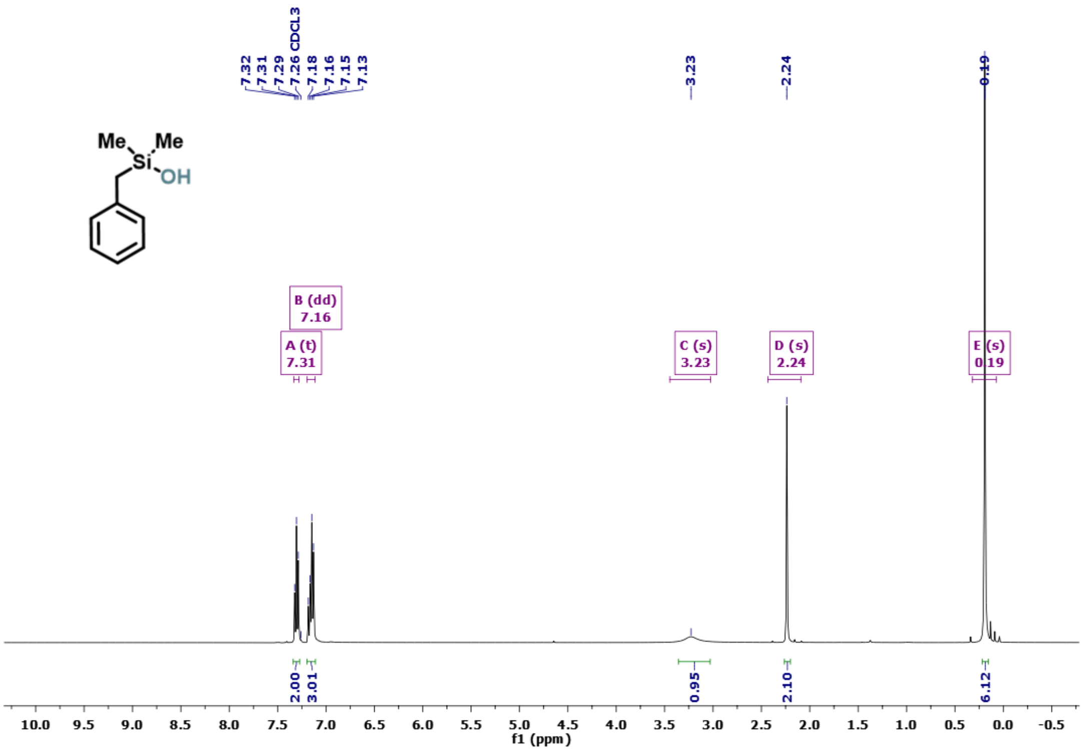

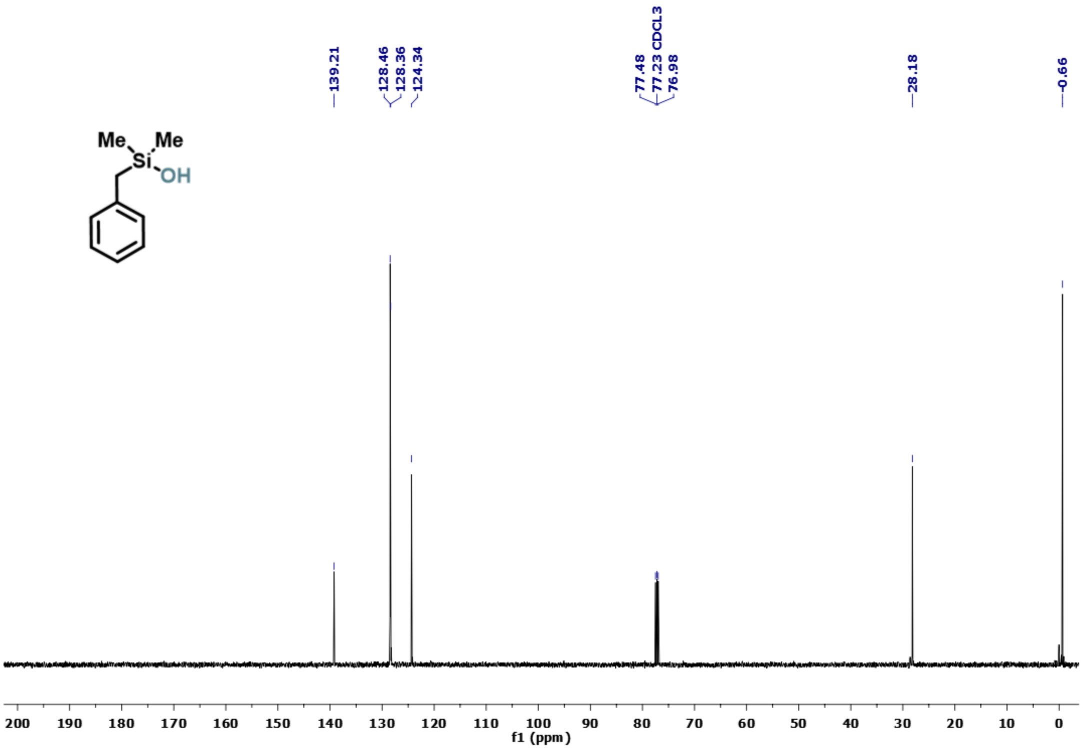

(2,3-dichlorobenzyl)diisopropylsilanol (55):

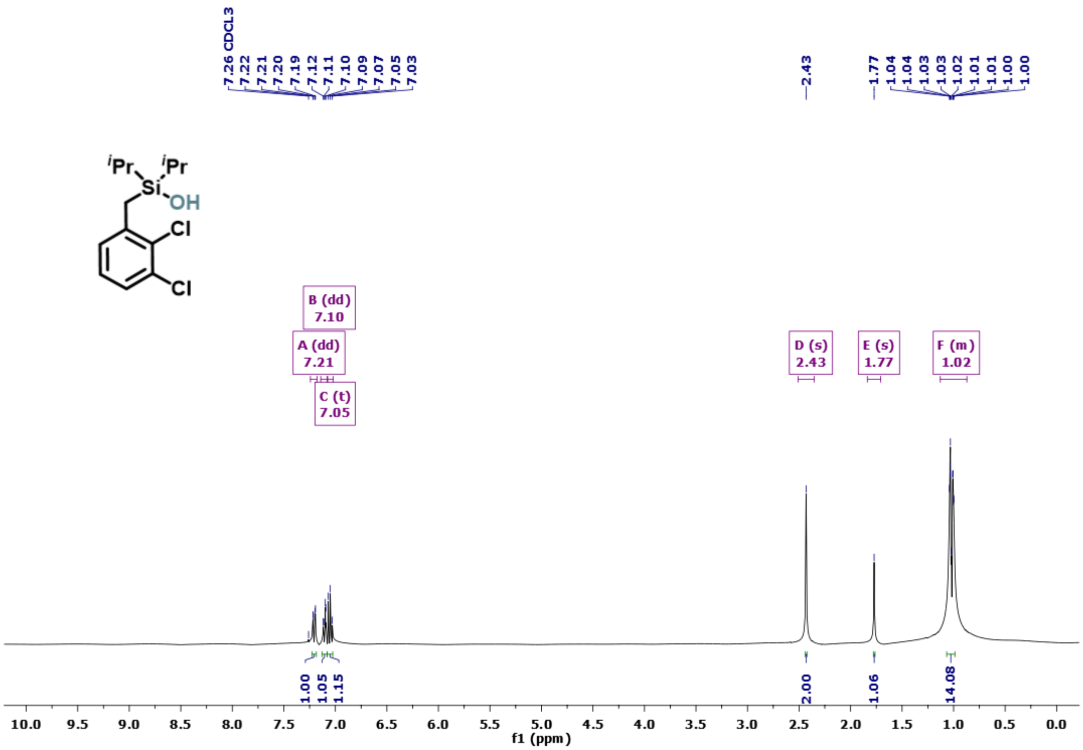

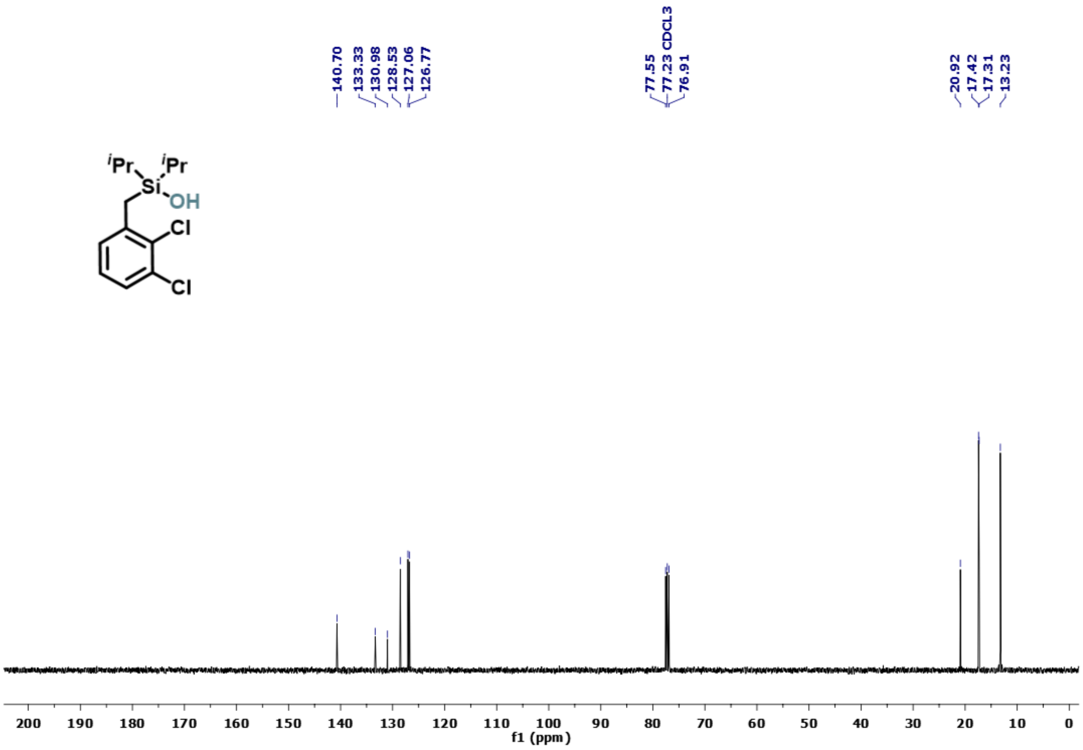

Diisopropyl(naphthalen-1-ylmethyl)silanol (56):

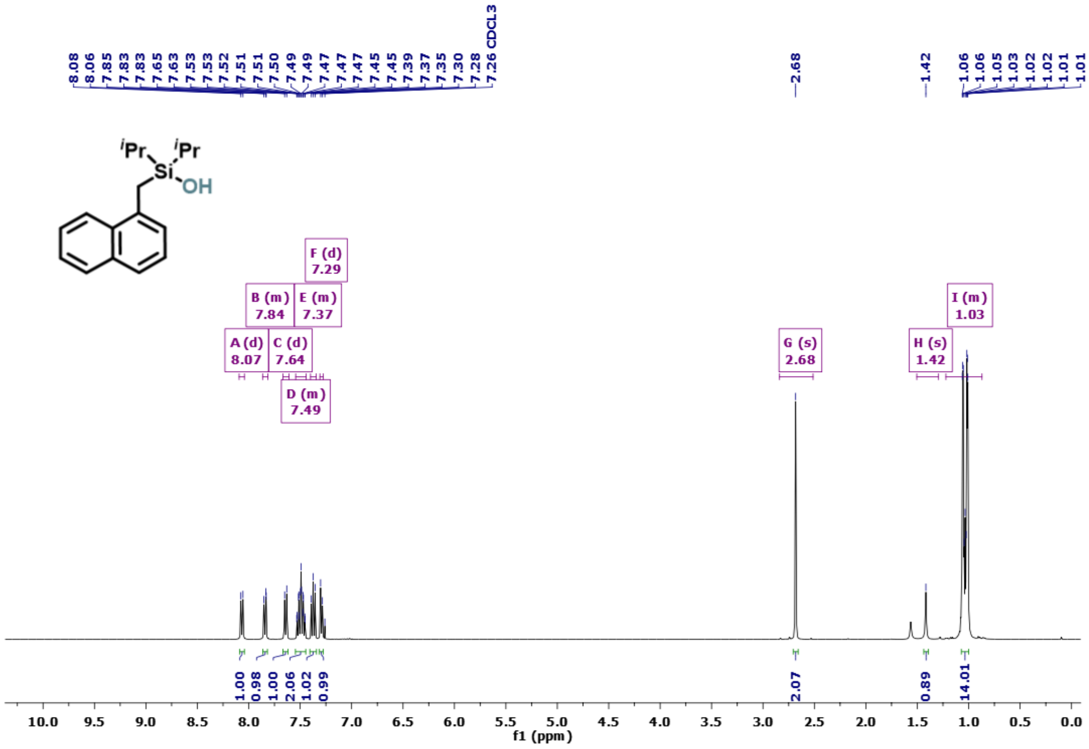

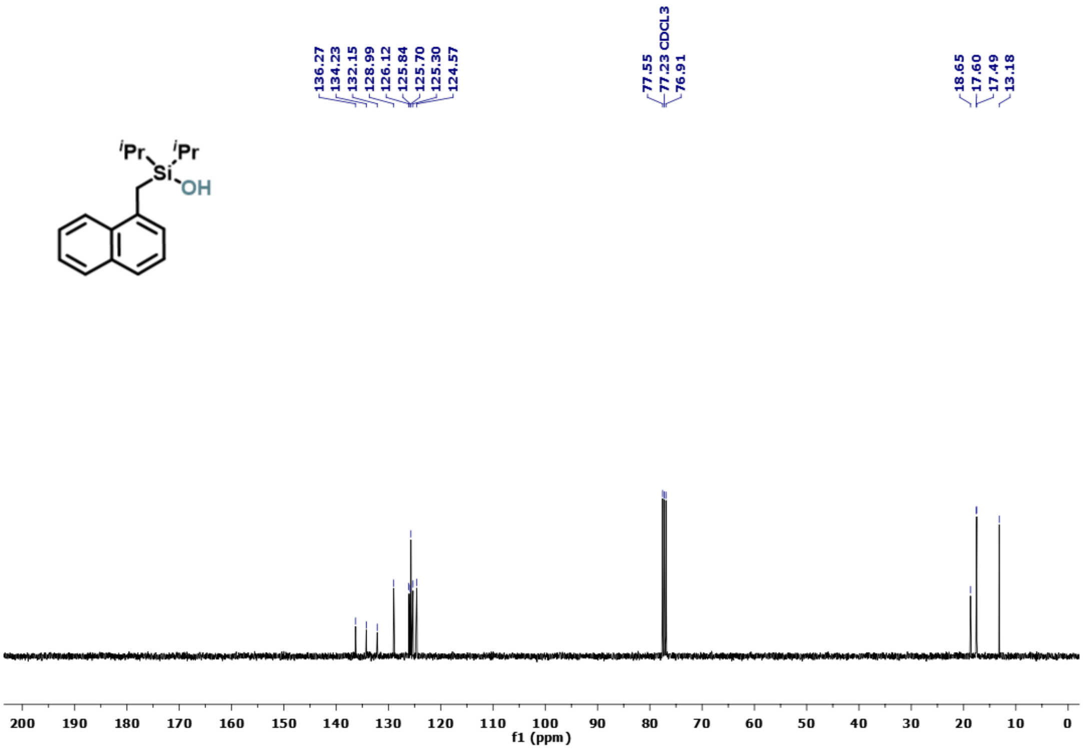

(10,11-Dihydro-5H-dibenzo[a,d][7]annulen-5-yl)dimethylsilanol (57):

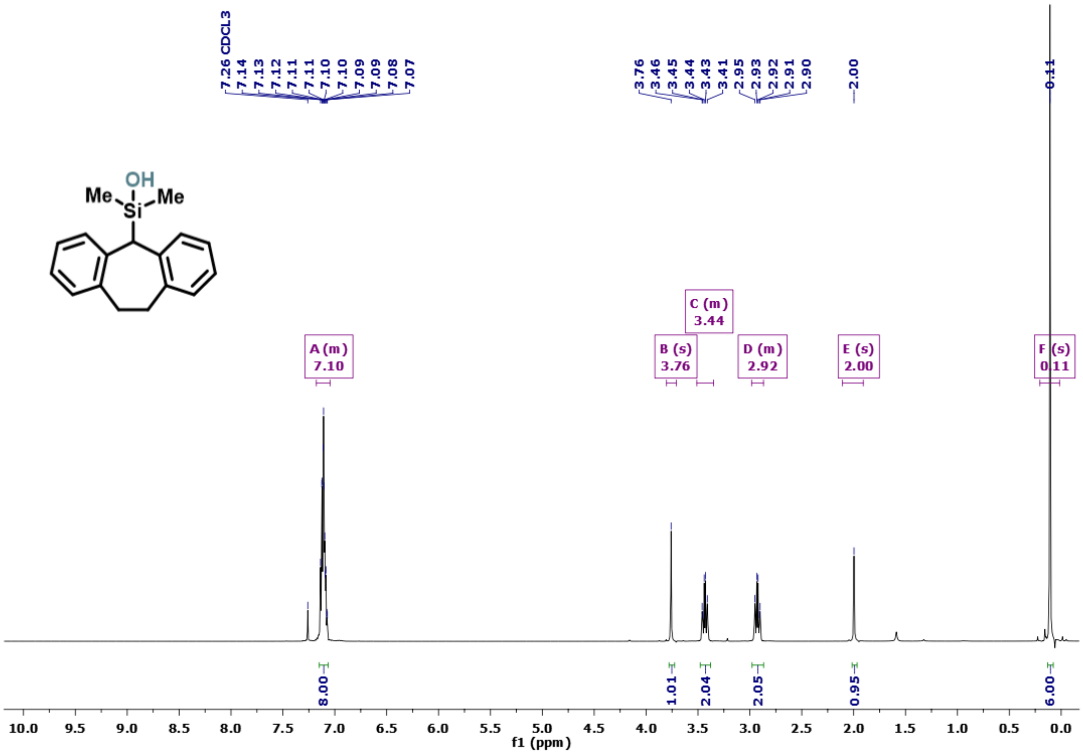

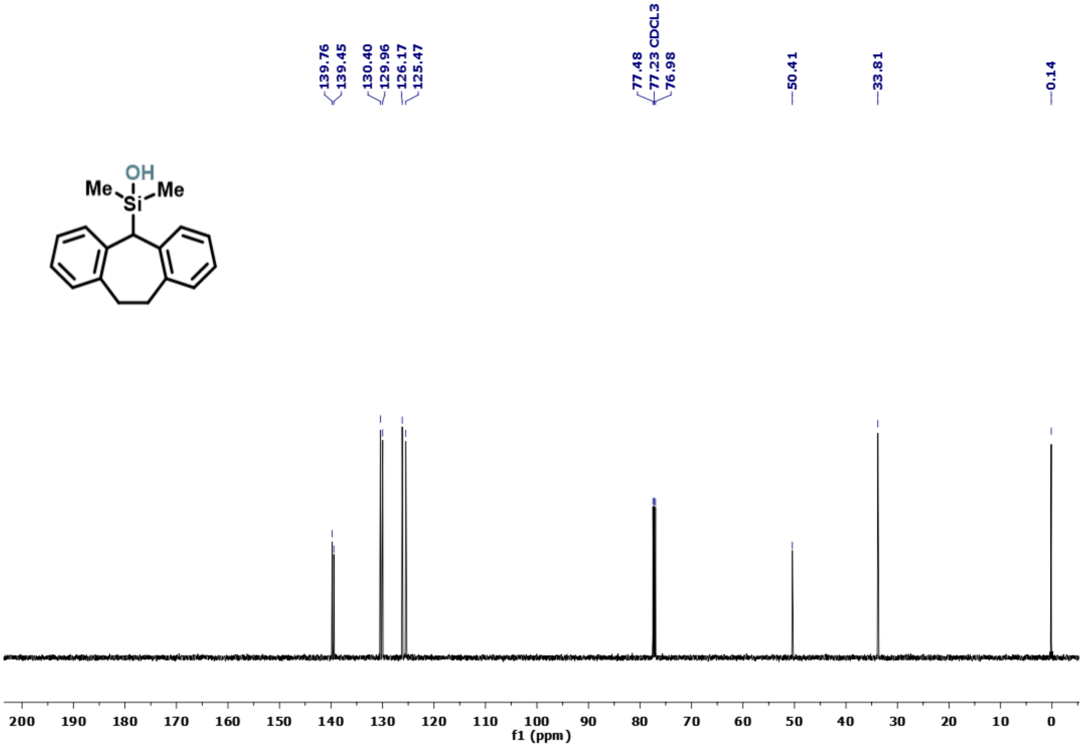

Dimethyl(1-phenylethyl)silanol (58):

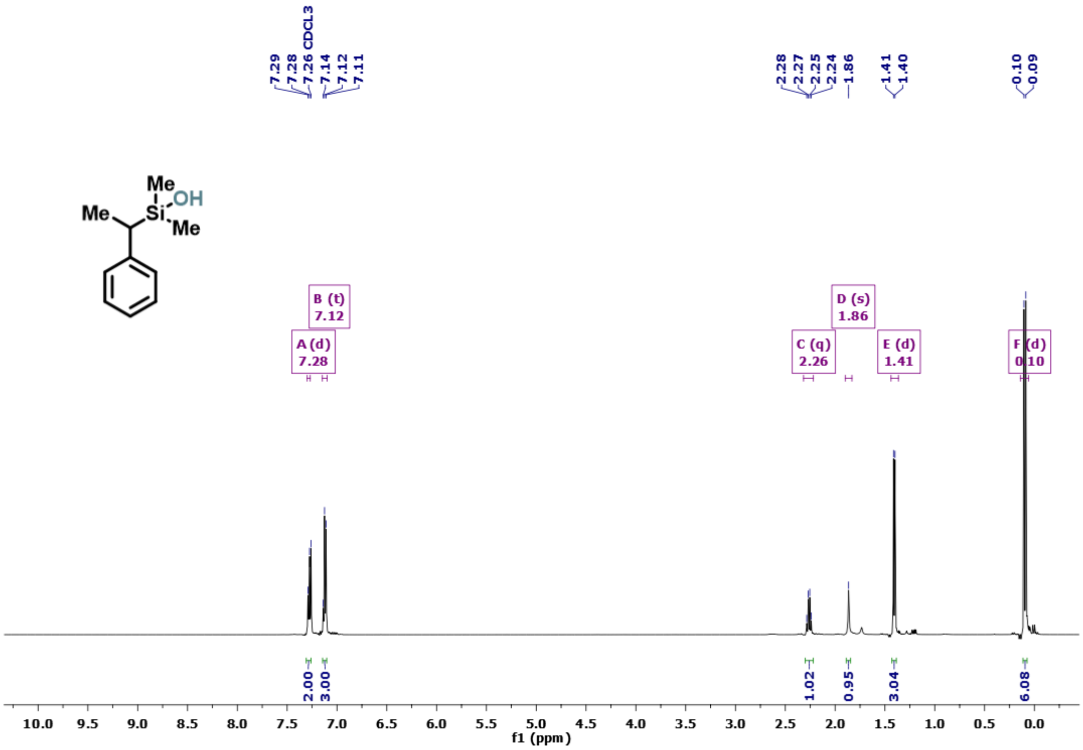

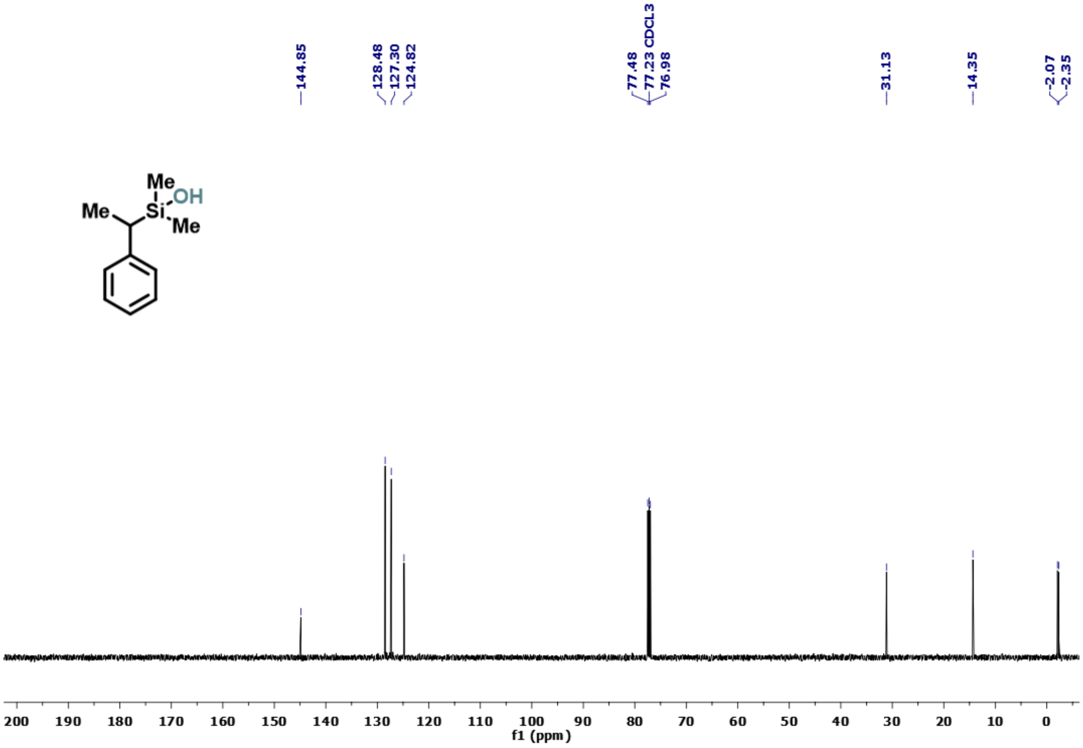

Triisobutylsilanol (59):

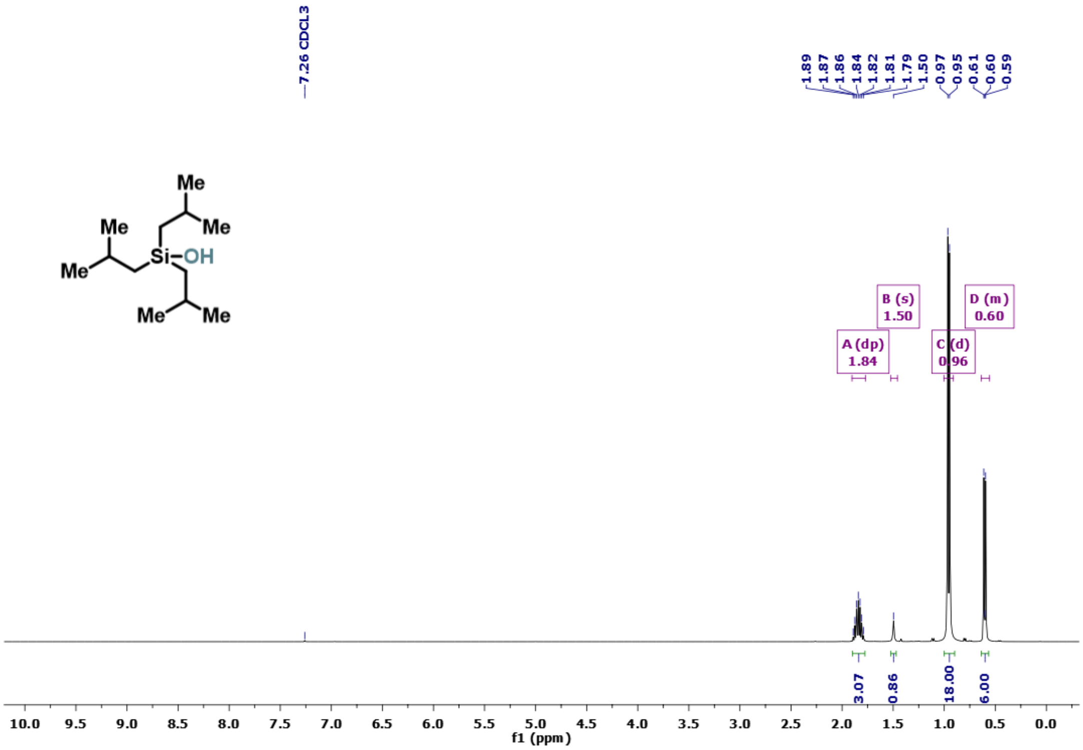

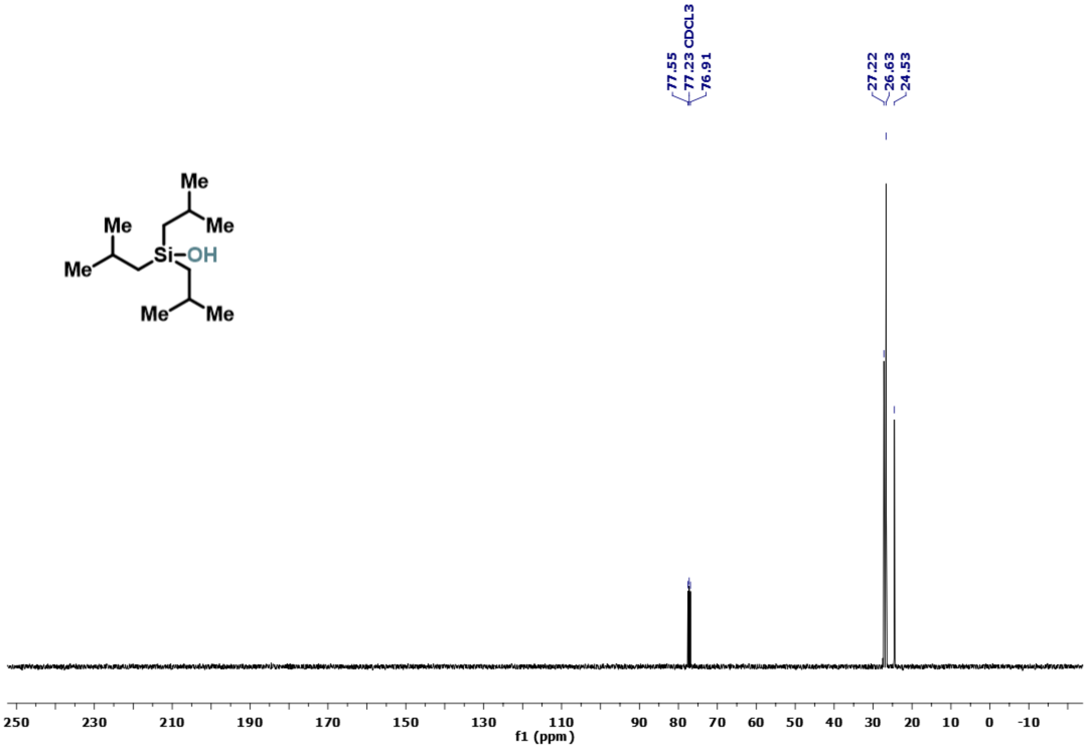

Methyl 4'-methoxy-[1,1'-biphenyl]-4-carboxylate (61):

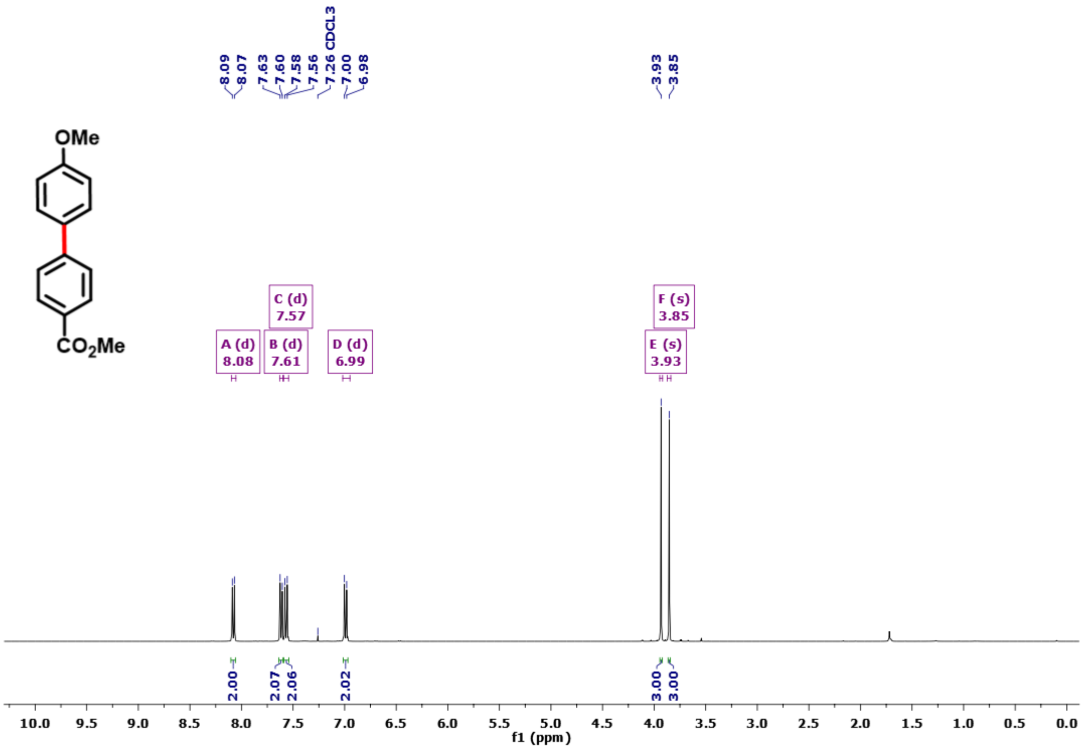

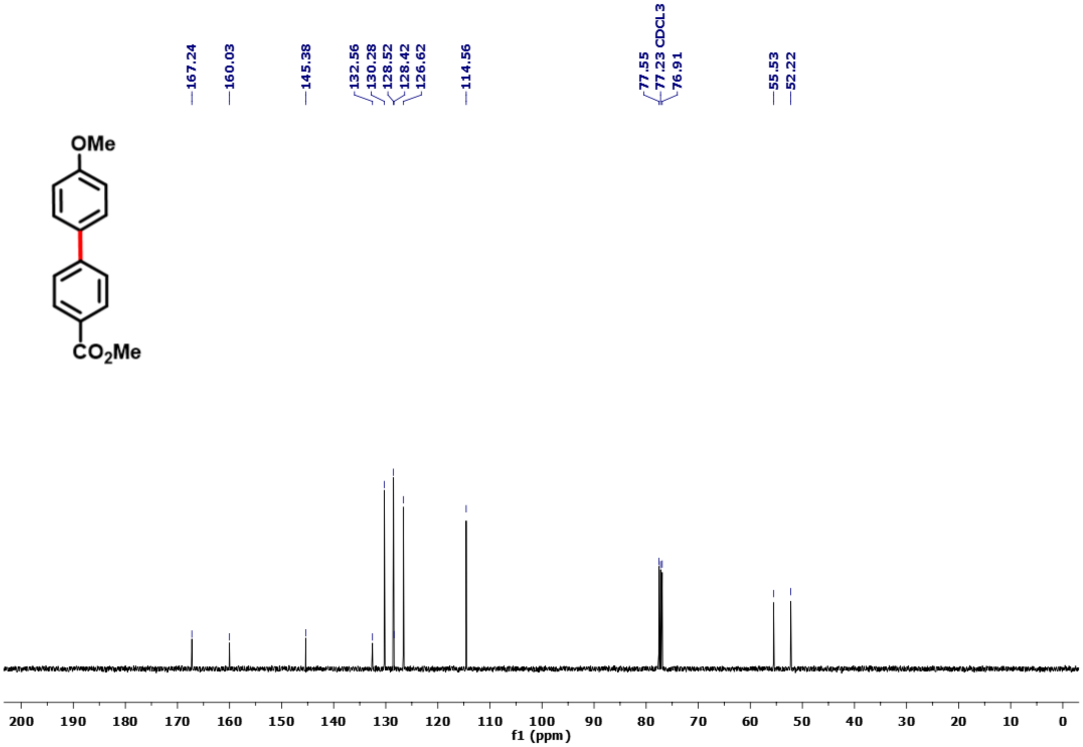

Methyl (*E*)-3-(2-((hydroxydiisopropylsilyl)methyl)phenyl)acrylate (62):

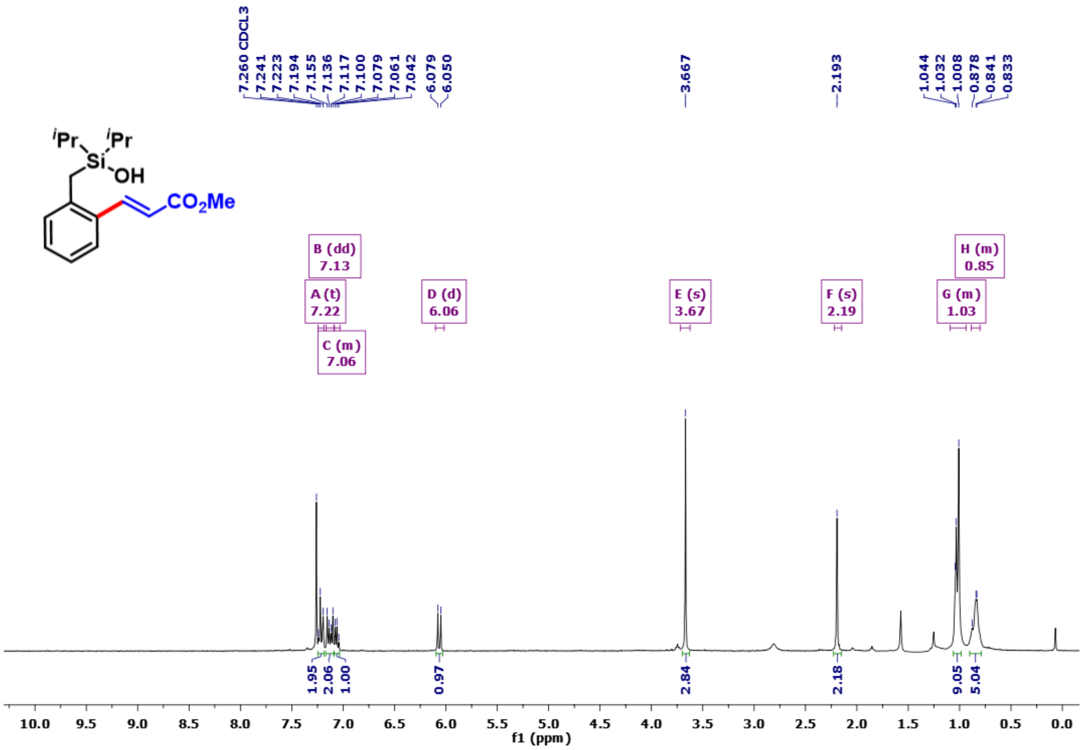

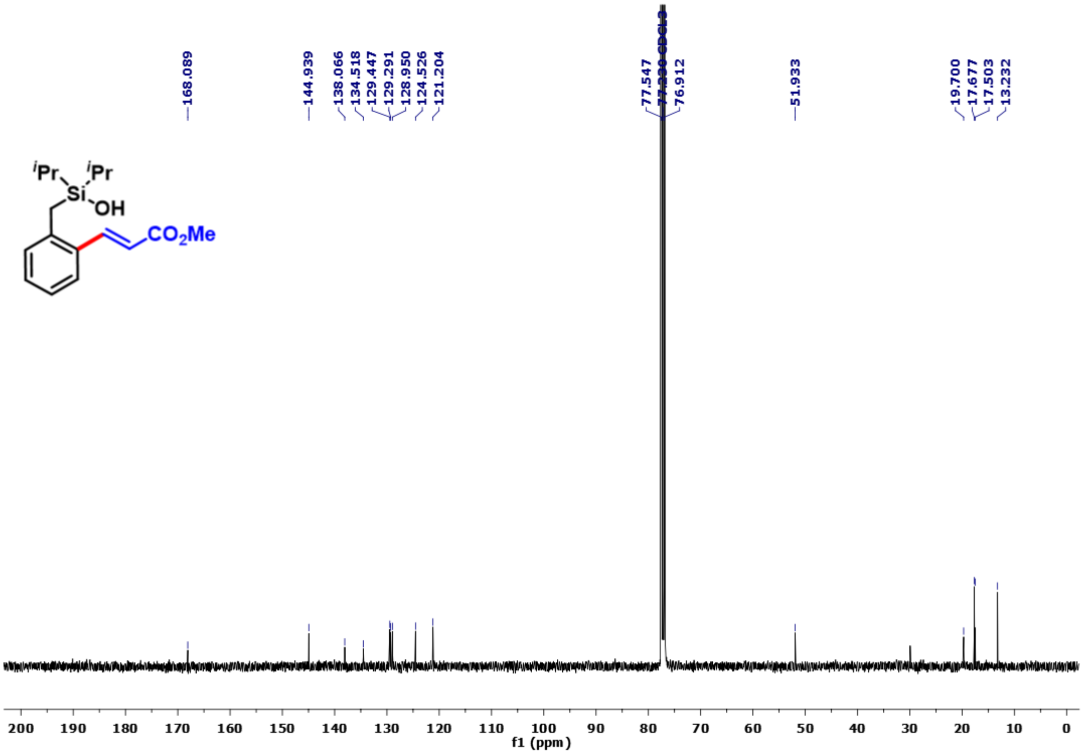

Methyldiphenylsilanol-O*D* (65):

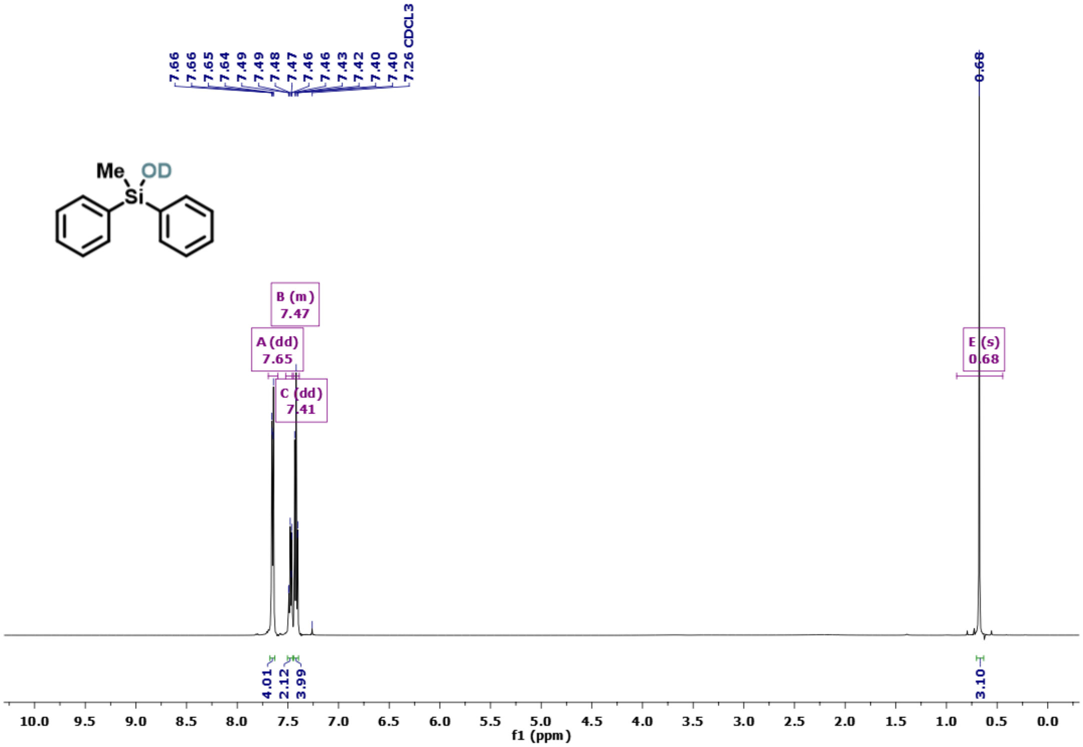

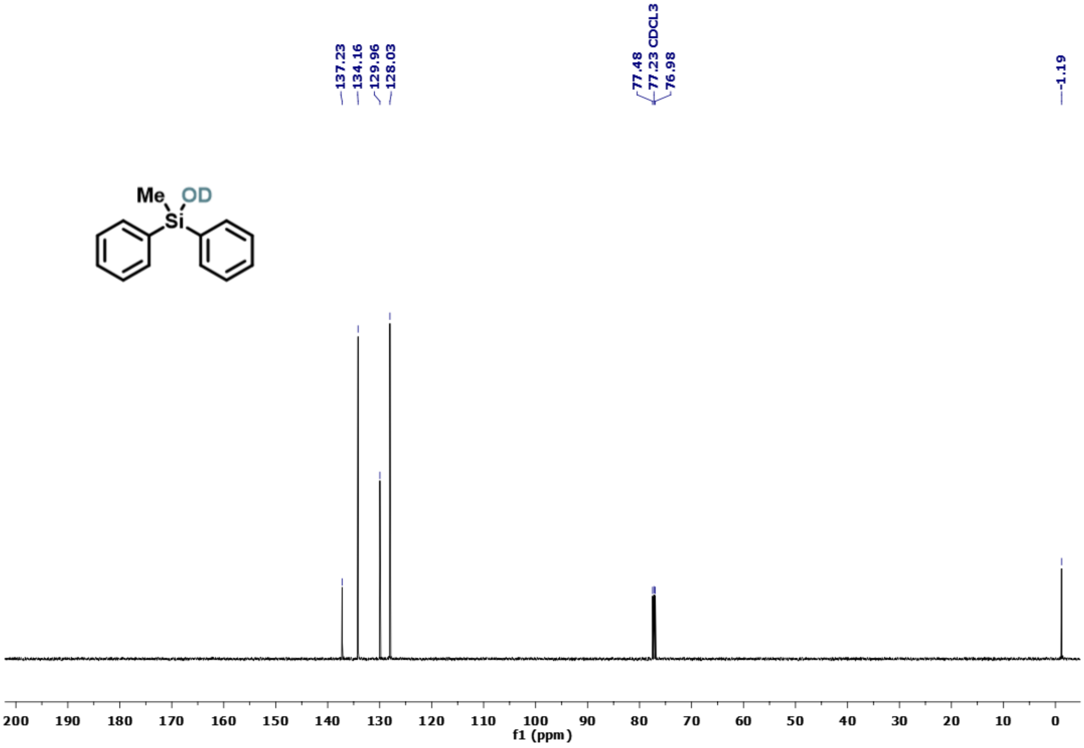

Trihexylsilanol-O*D* (66):

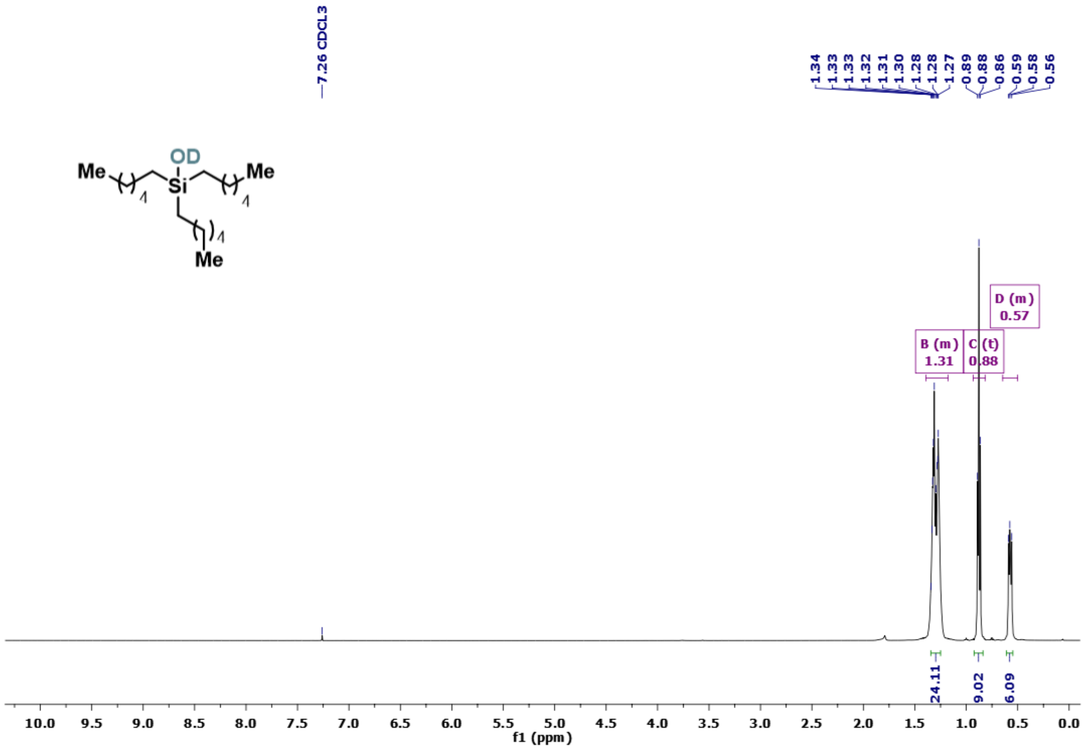

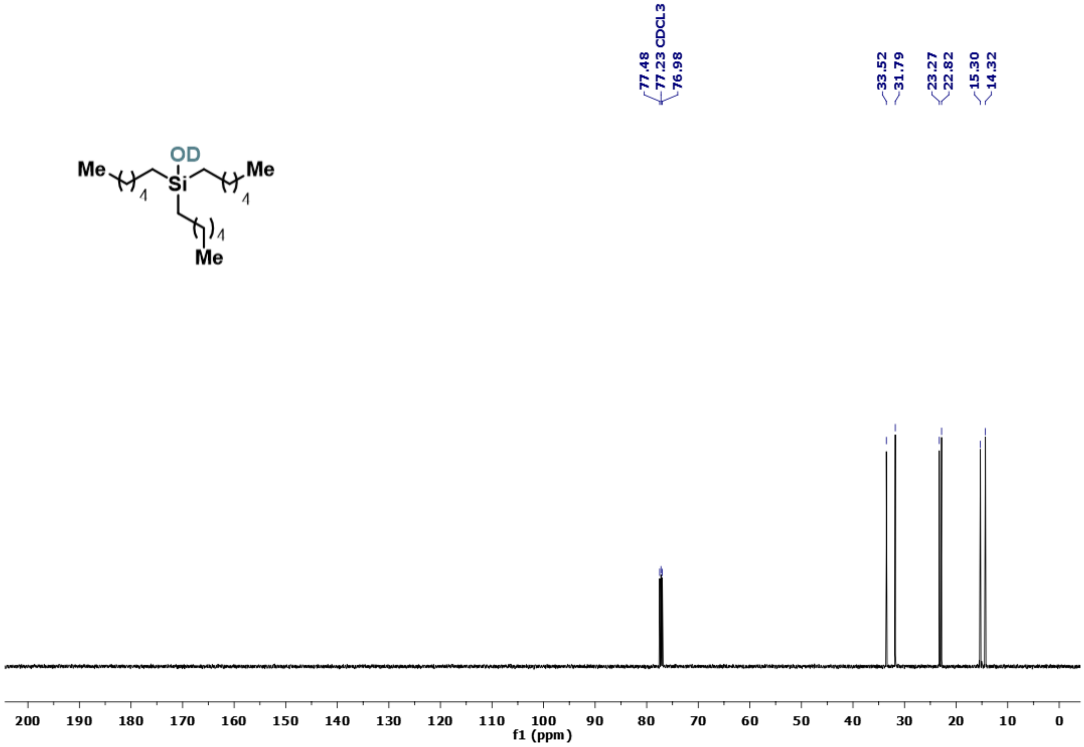

Methyldiphenylsilanol-^{18}O (67):

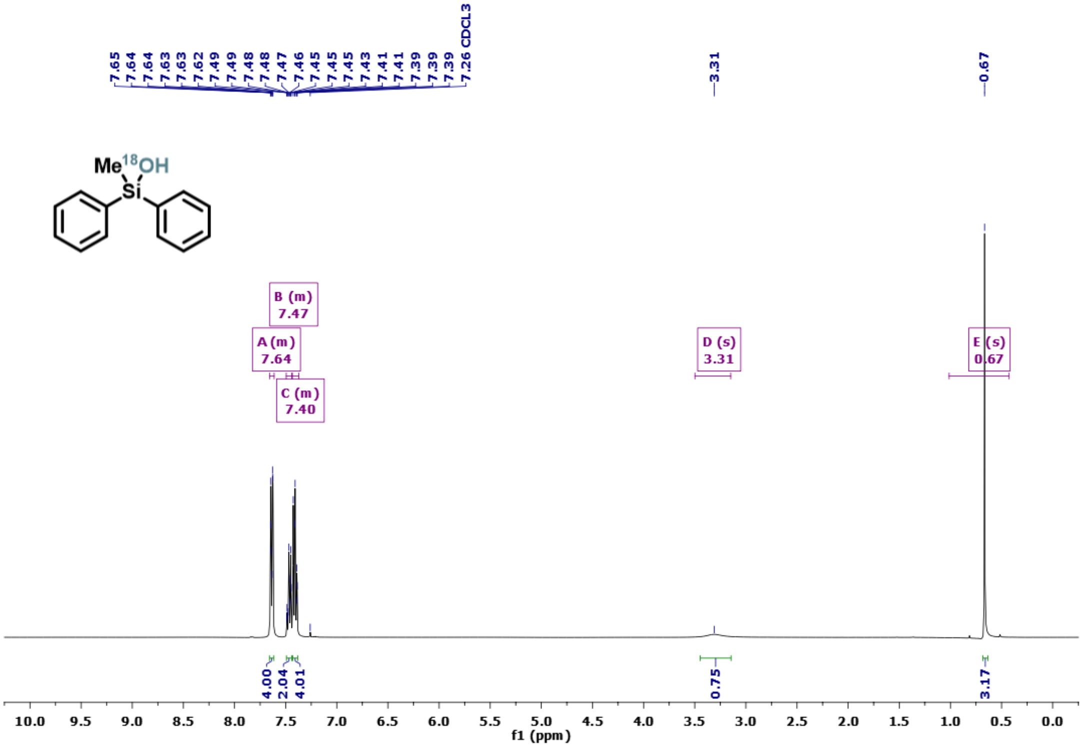

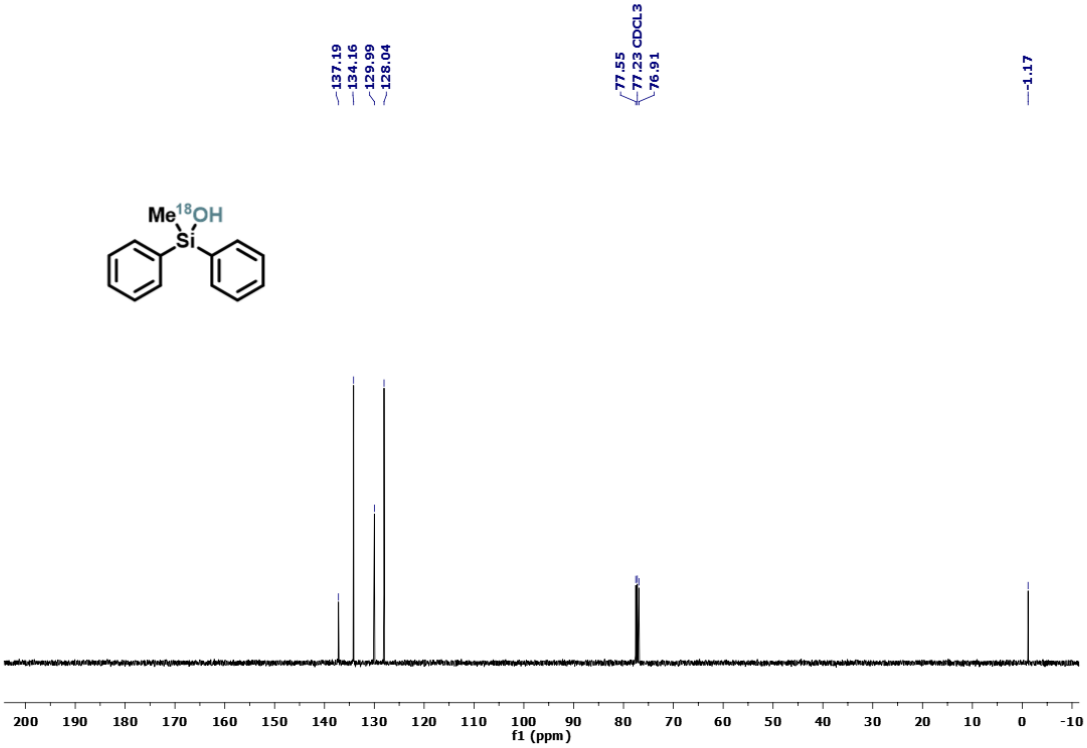

Methoxytriphenylsilane (68):

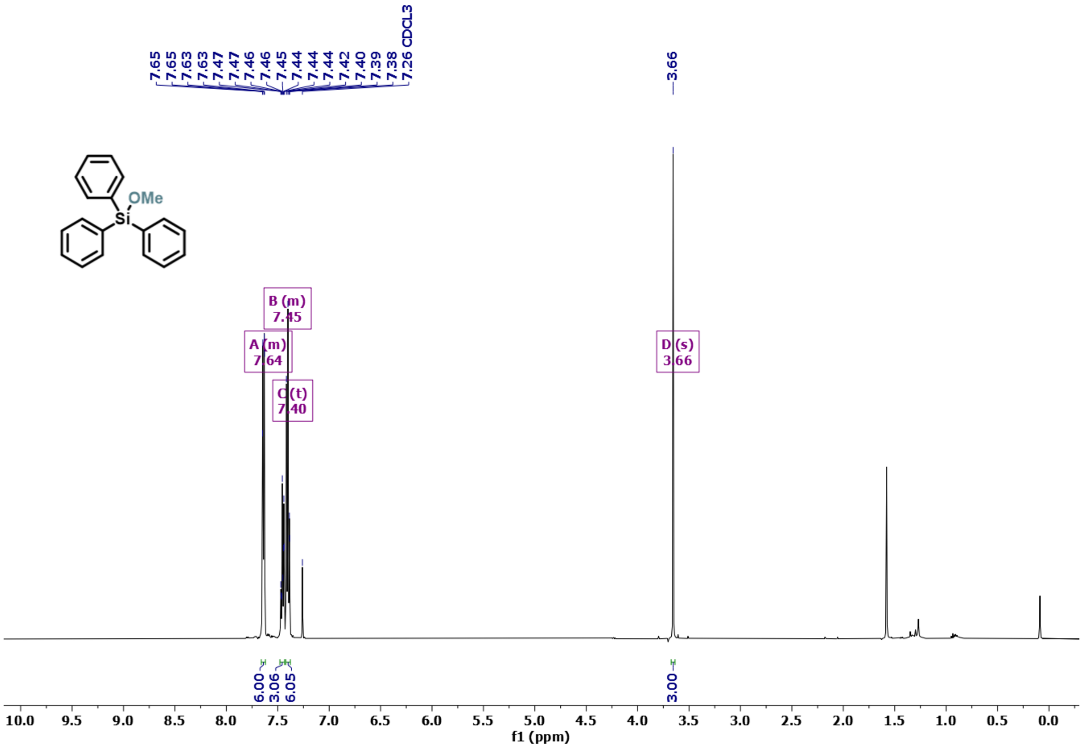

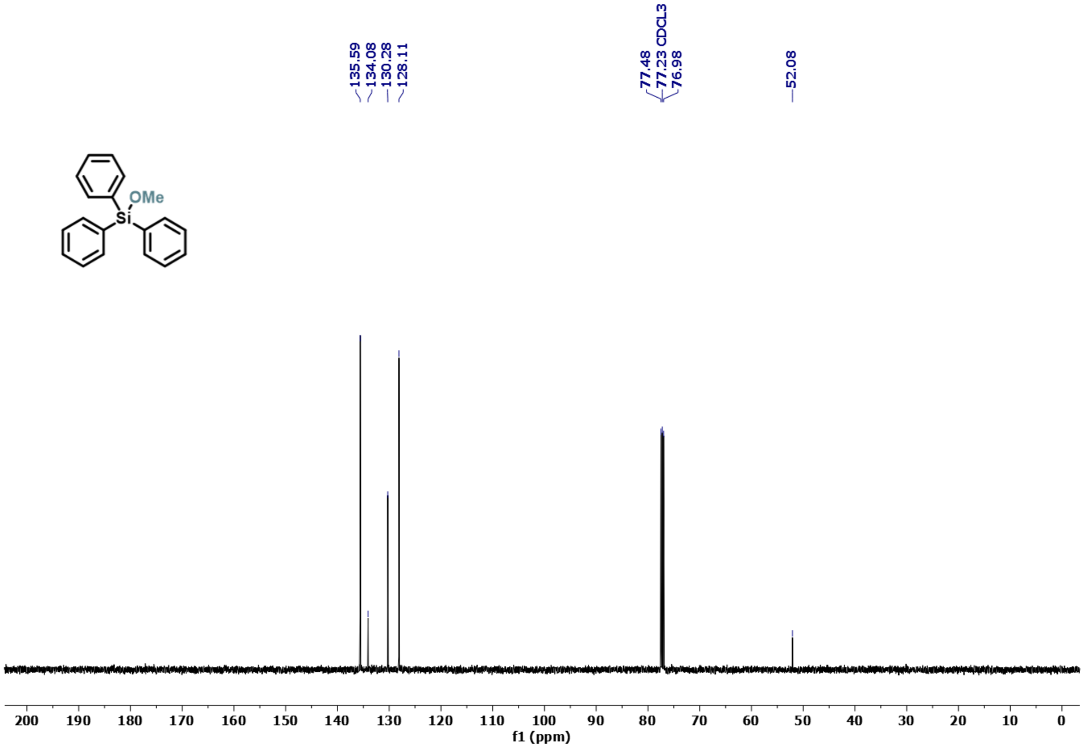

Ethoxytriphenylsilane (69):

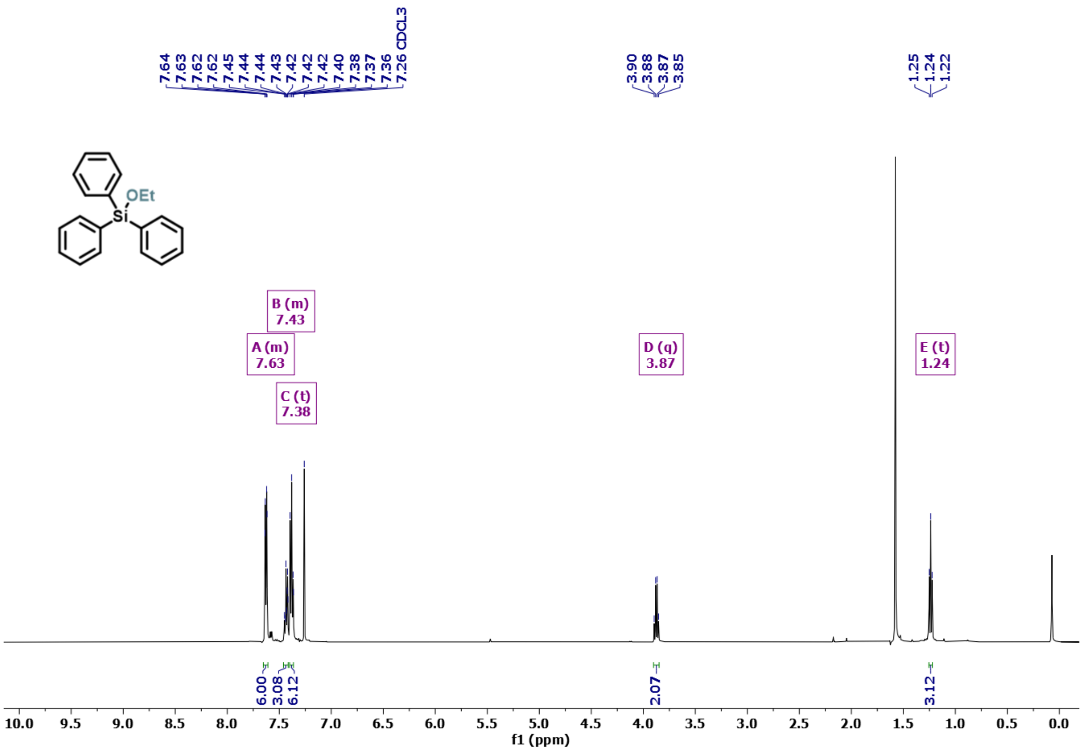

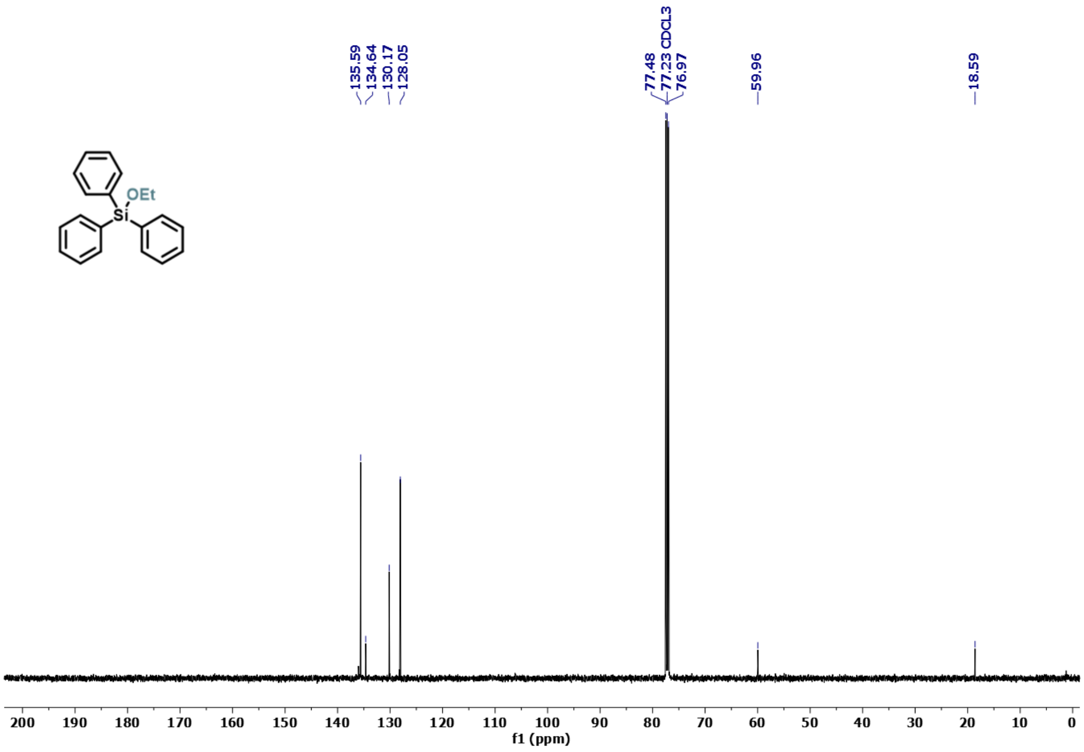

8. References:

1. Wang, K. K.; Zhou, J. M.; Jiang, Y. T.; Zhang, M. M.; Wang, C.; Xue, D.; Tang, W. J.; Sun, H. M.; Xiao, J. L.; Li, C. Q. Selective Manganese-catalyzed Oxidation of Hydrosilanes to Silanols under Neutral Reaction Conditions. *Angew. Chem. Int. Ed.* **58**, 6380–6384 (2019).

2. Lee, Y.; Seomoon, D.; Kim, S.; Han, H.; Chang, S.; Lee, P. H. Highly Efficient Iridium-catalyzed Oxidation of Organosilanes to Silanols. *J. Org. Chem,* **69**, 1741–1743 (2004).

3. Liang, H.; Wang, L.-J.; Ji, Y.-X.; Wang, H.; Zhang, B. Selective Electrochemical Hydrolysis of Hydrosilanes to Silanols via Anodically Generated Silyl Cations. *Angew. Chem., Int. Ed*. **60**, 1839−1844 (2021).

4. Zhang, Q., Peng, M., Gao, Z., Guo, W., Sun, Z., Zhao, Y., Zhou, W., Wang, M., Mei, B., Du, X. L., Jiang, Z., Sun, W., Liu, C., Zhu, Y., Liu, Y. M., He, H. Y., Li, Z. H., Ma, D., Cao, Y. Nitrogen-Neighbored Single-Cobalt Sites Enable Heterogeneous Oxidase-Type Catalysis. *J. Am. Chem. Soc.* **145**, 4166−4176 (2023).

5. Huang, C.; Ghavtadze, N.; Godoi, B.; Gevorgyan. V. Pd-Catalyzed Modifiable Silanol-Directed Aromatic C-H Oxygenation. *Chem. Eur. J.* **18**, 9789 – 9792 (2012).

6. Denmark, S. E.; Ober, M. H. Cross-Coupling Reactions of Aryl silanols with Substituted Aryl Halides. *Org. Lett.*, **5**, 1357–1360 (2003).

7. Saha, A., Guin, S., Ali, W., Bhattacharya, T., Sasmal, S., Goswami, N., Prakash, G., Sinha, S. K., Chandrashekar, H.B., Panda, S., Anjana, S. S., Maiti. D. Photoinduced Regioselective Olefination of Arenes at Proximal and Distal Sites. *J. Am. Chem. Soc.* **144**, 1929–1940 (2022).

8. Cismesia, M. A. & Yoon, T. P. Characterizing chain processes in visible light photoredox catalysis. *Chem. Sci.* **6**, 5426–5434 (2015).

9. A new sensitive chemical actinometer - II. Potassium ferrioxalate as a standard chemical actinometer. *Proc. R. Soc. London. Ser. A. Math. Phys. Sci.* **235**, 518–536 (1956).

10. Wegner, E. E. & Adamson, A. W. Photochemistry of Complex Ions. III. Absolute Quantum Yields for the Photolysis of Some Aqueous Chromium(III) Complexes. Chemical Actinometry in the Long Wavelength Visible Region. *J. Am. Chem. Soc.* **88**, 394–404 (1966).

11. Li, J., Dandan Xu, D., Shi, G., Liu, X., Zhang, J., Fan, B. Oxidation of Silanes to Silanols with Oxygen via Photoredox Catalysis. *Chemistry Select* **6**, 8345 –8348 (2021).

12. Li, S., Li, H., Tung, C. H., Liu, L. Practical and Selective Bio-Inspired Iron-Catalyzed Oxidation of Si−H Bonds to Diversely Functionalized Organosilanols. *ACS Catal.* **12**, 9143−9152 (2022).
